# Selecting medical research data platforms for translational biomedical research: a five-tier overview and requirement-weighted assessment framework

**DOI:** 10.3389/fdgth.2026.1814015

**Published:** 2026-06-17

**Authors:** Marc Jacobs, Samad Goudarzi, Jan Stücke, Robin Röhm, Timo Kanninen, Tero Oinonen, Petra Ritter, Michael Schirner, Fabian Prasser, Arman Bhagwagar, Bernd Matzenbach, Hartmut Schultze, Joachim Schultze, Mathias Göschl, Henrik Matthies, Kenneth R. Evans, Moyez Dharsee, Christian Stephan, Marvin Belz, Philip Gribbon, Katja Herzog, Chester Chen, David Ruau, Ittai Dayan, Adrish Sannyasi, Dieter Kranzlmüller, Naweiluo Zhou, Jens Warfsmann, Martin Hoffmann, Rudi Schmidt, Martin Hofmann-Apitius

**Affiliations:** 1 Bioinformatics Department, Fraunhofer-Institute for Algorithms and Scientific Computing (SCAI), Sankt Augustin, Germany; 2 Apheris AI GmbH, Berlin, Germany; 3 BC Platforms, Espoo, Finland; 4 Berlin Institute of Health at Charité — Universitätsmedizin Berlin, Charité, Berlin, Germany; 5 Center of Health Data Sciences, BIH @ Charité, Berlin, Germany; 6 DNANexus, Mountain View, CA, United States; 7 Deutsches Zentrum für Neurodegenerative Erkrankungen, Bonn, Germany; 8 42Hills GmbH, Königswinter, Germany; 9 HiGHmed e.V., Heidelberg, Germany; 10 HONIC Health Data Technologies GmbH, Neckarsulm, Germany; 11 Indoc Systems, Toronto, ON, Canada; 12 IQVIA, Bochum, Germany; 13 Fraunhofer Institute for Translational Medicine and Pharmacology ITMP, Discovery Research ScreeningPort, Hamburg, Germany; 14 NVIDIA, Santa Clara, CA, United States; 15 RHINO Federated Computing, Boston, MA, United States; 16 Leibniz Supercomputing Centre (LRZ) of the Bavarian Academy of Sciences and Humanities, Garching, Germany; 17 Division of Personalized Tumor Therapy, Fraunhofer Institute for Toxicology and Experimental Medicine (ITEM), Regensburg, Germany; 18 Bonn-Aachen International Center for Information Technology (B-IT), University of Bonn, Bonn, Germany

**Keywords:** medical research data platforms, translational biomedical research, equirement-weighted assessment, data privacy, FAIR data, iteroperability, federated learning, data harmonization

## Abstract

**Introduction:**

Translational biomedical research is increasingly collaborative and multimodal, making secure, high-quality data capture, curation, and analytics a major challenge. This work aims to provide an overview of existing medical research data platforms to support informed platform selection for translational biomedical research.

**Methods:**

As part of an ongoing Fraunhofer Request for Proposal (RFP) process, we developed a requirements assessment tool for users across the Fraunhofer ecosystem. In parallel, we compiled a structured overview of medical research data platforms through an open collaboration between academic and industry experts, who supplemented our market screening by identifying additional relevant platforms. Using a standardized questionnaire on key aspects of distributed data collaboration, we collected harmonized platform descriptions and organized them into a side-by-side overview with an accompanying feature weighting matrix.

**Results:**

The study yielded a structured, comparative characterization of medical research data platforms across five functional classes, highlighting common strengths in security, interoperability, data quality, and multimodal data support. We devised (developed) a platform feature-partner weight matrix that enables context-sensitive platform scoring without imposing a predefined global ranking. In this way, users can align platform scoring with their specific translational research requirements.

**Discussion:**

This structured, overview is intended to accelerate decision-making in the medical research community when choosing data platforms. By supporting context-sensitive, feature-weighted selection rather than one-size-fits-all comparisons, it acknowledges diversified research needs and can be updated as technologies and practices evolve.

## 1 Introduction

Translational biomedical research is increasingly collaborative, multidisciplinary, and multimodal. Beyond classical clinical studies and trials with highly stratified patient cohorts, there is a growing trend toward integrating observational and real-world data (RWD) from multiple sources with emerging data classes such as multi-omics. This integration is essential for both generating population-level evidence relevant to regulatory purposes, including drug approval, post-market surveillance, and drug repurposing, and for enabling *de novo* biomarker discovery.

At the same time, biomedical research data platforms are facing rapidly increasing demands. These arise not only from the introduction of new data classes, but also from the growing complexity within established modalities, for example in the transition from multi-omics to single-cell multi-omics. As a result, platform development is progressing rapidly to address the increasing dimensionality, heterogeneity, and analytical complexity of translational biomedical research.

Both the academic community and the commercial market respond to these new challenges by proposing and implementing new platforms supporting the management and analysis of clinical, omics, imaging and biomarker data from clinical trials and real-world data sources. These platforms support collaborative work, and they allow for harmonization and FAIRification of heterogeneous, multimodal data. Ultimately, they support advanced methods of modeling and data mining, usually involving machine learning (ML) and artificial intelligence (AI).

Given the complexity and rapid evolution of the medical research data platform landscape, users face a major challenge when selecting and implementing such systems: obtaining a sufficiently comprehensive market overview within a realistic timeframe, before new releases make the basis for procurement or tendering obsolete. What is additionally lacking is a clear, common understanding of strategies for a systematic platform choice assessment (SWOT-analysis).

By this structured overview, we aim to address both the lack of orientation in a rapidly evolving platform landscape and the absence of practically grounded assessment approaches. We present a comprehensive overview of medical research data platforms, describing their core functionalities and properties across a broad spectrum of systems designed for the management and analysis of translational biomedical data. In contrast to previous reviews, which typically analyse a limited number of academic translational platforms or focus on patient-data discovery and real-time analytics infrastructures, our work covers a broader spectrum of commercial and academic systems, spans five functional platform classes, and provides a feature-level matrix that supports context-specific, weighted platform selection (1–3).

A central contribution of this work is the introduction of a five-tier classification scheme that spans the full range of architectures, from local data hosting environments to distributed, federated, and decentralized learning systems (c.f. section 2.2). Building on this framework, we further provide a structured evaluation matrix derived from a real-world procurement (RFP) context at Fraunhofer, enabling requirement-driven assessment of platforms beyond purely theoretical considerations. The Fraunhofer-Gesellschaft is Germany's largest organization for applied research, dedicated to advancing technology and translating scientific knowledge into practical solutions in collaboration with industry, government, and academia.

Finally, we outline a path toward more systematic and comparable evaluation of platform capabilities and limitations through coordinated benchmarking efforts, including the use of standardized scenarios and datasets.

## 2 Methodology

The work presented here was motivated by the MED-connect project coordinated by the Fraunhofer Institute for Toxicology and Experimental Medicine (ITEM) and funded by Fraunhofeŕs internal research program, which aims at testing and implementing a solution for collaborative, data-driven, translational clinical research. MED-connect is a Fraunhofer platform for medical research data and data-driven research, currently being developed by a consortium of seven Fraunhofer Institutes. The platform addresses structural limitations in health research by providing a centralised infrastructure for data management and analysis. The focus is particularly on the fragmentation of study and patient data, as well as the lack of harmonisation and standardisation in data storage and curation.

The platform is designed to enable GDPR-compliant storage and management of pseudonymised medical research data, support multimodal data analysis including AI-based tools, and ensure secure data access with clearly defined data protection concepts. Its development is guided by specific clinical use cases, and the platform is to be strategically positioned to integrate Fraunhofer research into clinical trials and support translational research and development for both industry and clinical partners. Key to this project is a dedicated benchmarking effort that compares the utility and cost of a limited number of candidates for the collaborative medical research data platform. The Fraunhofer partners thus have worked out a systematic approach for the collection of relevant information on medical research data platforms for rational decision making - including technical benchmarking, legal compliance and total cost of operations.

In gathering information on medical research data platforms, we adopted a multi-source approach to ensure comprehensive analysis. Our first step involved conducting systematic searches via Google, targeting general information on widely used platforms. Keywords such as “medical research platforms,” “medical data platforms,” and “cloud-based healthcare platforms” were employed to identify key systems and trends in the field. To supplement this, we explored several academic databases including PubMed, Google Scholar, Scopus, and Web of Science, focusing on peer-reviewed articles and reviews discussing the capabilities, security, and scalability of specific platforms similar to a recent review (1). Search terms such as “i2b2 clinical research” or “I2B2 features” were utilized to extract scholarly insights. This internet search for platforms resulted in a long list of academic and commercial systems. The complete list is provided in supplementary S3 and described in the results section.

Furthermore, we manually reviewed the official websites of platforms to collect detailed technical specifications, case studies, and real-world implementations. It should be noted that not all platforms included in this overview were characterized with the same level of direct vendor input. For a subset of platforms, including for example Aridhia, Siemens Health, and i2b2, descriptions were derived solely from publicly available information, as active participation or direct feedback from the respective providers could not be obtained. While efforts were made to ensure accuracy through careful review of documentation and published sources, these entries may differ in depth and completeness compared to platforms for which primary input was available.

Pure Hospital Information Systems (HIS, like ORBIS^1^, CGM MEDICO^2^), Electronic Health Record Systems (EHR, like EPIC^3^, Oracle Cerner^4^), or Electronic Data Capture Systems (EDC, like Castor EDC^5^, REDCap^6^) were not considered, as these systems do not support the type of translational clinical research we intend to cover with our approach. Based on this initial screening of public information, we generated a short list that we wanted to study in detail (see section 2.3). Our focus was on platforms that have been in use by a broader European community over the past five years. Systems that are solely US-based without any available information on GDPR compliance, as well as academic prototypes lacking an active user community, were excluded for the next step.

The selection of platforms for detailed characterization was guided not only by the initial market and literature screening, but also by practical considerations regarding the feasible scope of such an assessment. Several co-authors affiliated with private-sector organizations, as well as additional stakeholders involved in procurement and implementation of data platforms, were consulted to define what constitutes a realistic and proportionate scope for a market screening exercise. These discussions informed the selection process by balancing the ambition of achieving broad coverage with the practical constraints of time, resources, and the level of engagement that could be expected from platform providers. As a result, the final set of platforms reflects both relevance to the intended application context and a scope that is considered manageable and representative from the perspective of real-world procurement processes in the biomedical research domain.

Following the initial narrowing down of platforms, key factors such as data security and privacy, compliance with healthcare regulations (e.g., HIPAA, GDPR), scalability, user interface, and integration with existing systems were systematically documented. By combining data from academic, web-based sources, and official platform documentation, we aim at delivering a robust evaluation of both commercial and academic platforms, ensuring a well-rounded perspective on their technical details and user experiences.

Information on key features of medical research data platforms was collected and assessed through a structured process designed to maximize transparency and consistency. We actively adhered to the principles of pre-competitive collaborative science as we have successfully practiced before in various public-private partnerships (e.g., Innovative Medicine Initiative, IMI and Innovative Health Initiative, IHI) before.

Some of the medical research data requirements were systematically acquired by colleagues of Fraunhofer ITEM. The academic lead authors of this overview process are also involved in several publicly funded translational medicine projects of which some are aimed at generating re-usable components for medical data management and platform technologies (e.g., IDERHA^7^, I2B2/tranSMART^8^). Additional expert opinion input for this overview came from academic consortia such as the SWARM learning consortium and the EBRAINS project^9^).

To acquire more detailed information and to enable interoperability of descriptions and platform features, a dedicated questionnaire was developed (see S2 in the supplementary material) and sent to the contributors of this review. The questionnaire was pre-annotated for I2B2 by our team to illustrate, how the leading open-source solution in this field looks like in the questionnaire context.

Each commercial and each academic partner was invited to become a co-author of this overview, with co-authorship limited to two persons per partner. All partners knew ahead about the other vendors that contributed; and each partner could retract their contribution during the final review of this manuscript. The entire writing process was open, transparent and the academic lead authors shared all relevant information with all partners to avoid any bias. We approached various vendors of medical research data platforms directly (some of them via their websites) and usually organized video conferencing calls to explain what we want to do and why. The same questionnaire was sent uniformly to all participants. Some of the vendors asked whether they could add other information that they deemed relevant for the assessment. This was allowed and welcomed.

To ensure transparency and avoid conflicts of interest, we informed every contributor about all other contributors. Academic scientists and scientists from commercial vendors provided in-depth information on their respective systems.

While preparing for this overview, we formalized the collection of information about platform features (see the supplementary material S1). Since different application scenarios require different services and strategies, we abstained from direct feature comparison of platforms. The questionnaire is a structured template sent to PIs or vendors to characterize a medical research data platform in a standardized way. It has three main parts:

- Introductory letter explaining the goal (systematic assessment of data sharing, virtual research, and federated learning platforms) and listing key evaluation criteria such as security, privacy, compliance, interoperability, data quality, usability, scalability, collaboration, cost, and ethics.
- A platform description section where contributors describe deployment and usage, give references, and provide an architecture diagram.
- Large matrices and tables where contributors fill in detailed information on platform features: security/privacy and regulatory compliance, interoperability and standards support, data quality, usability, scalability, supported data modalities, governance and collaboration tools, analytics and workflows (ETL, cohort discovery, CDM mapping, ontology services, built-in analysis tools, NLP, visualization, export/reporting), with space for references throughout.

While not listing discrete numbered questions, the template features expansive matrices with 10 + detailed tables prompting fillable responses on 50 + subtopics, plus spaces for references, diagrams, and examples like I2B2. This matrix-heavy format (e.g., rows for criteria like “Data Encryption” or “Genomic Data”) encourages comprehensive, referenced inputs rather than simple yes/no answers. A prefilled form for I2B2 has been handed out as a reference.

To support structured and application-driven assessment of medical research data platforms, we developed a spreadsheet-based decision-support tool that enables requirement-driven comparison of platform capabilities. The tool is based on a standardized feature matrix derived from platform questionnaires, in which relevant functionalities are systematically captured across multiple domains, including data integration, interoperability, governance, analytics, and machine learning.

Central to the tool is a flexible weighting mechanism that allows users to assign relative importance to individual features or feature groups, their specific research context and operational requirements. Platform scores are then calculated by aggregating feature-level information using these user-defined weights, thereby enabling a context-sensitive ranking of candidate systems. This approach explicitly avoids one-size-fits-all comparisons and instead reflects the heterogeneity of translational research use cases.

The tool was designed in the context of a real-world procurement (RFP) process within the Fraunhofer ecosystem and reflects practical constraints and decision criteria encountered in platform selection. It supports scenario-based evaluation, allowing different user groups (e.g., clinical researchers, data scientists, or IT stakeholders) to explore how varying priorities influence platform suitability. In addition, the structured representation of platform features enhances transparency and reproducibility of the assessment process.

Importantly, the tool is not intended to provide an objective benchmark of platform performance, but rather to facilitate informed decision-making by mapping platform characteristics to user-defined requirements. As such, it complements the descriptive overview presented in this study and provides a practical interface for translating qualitative platform features into quantitative, decision-relevant insights (see S3 feature descriptions, S18 weighting scheme).

The supplementary S3 is an aggregated factsheet matrix that compiles questionnaire responses for all characterized medical research data platforms. Columns list individual platforms, grouped by category (data integration, data sharing, data analysis, machine learning, federated learning), with an initial “Overview” block capturing contact persons for each vendor in a side-by-side format, with one column for definitions, and one prefilled example for i2b2. Rows are organized by 13 categories and 2 to 9 features or criteria per category (e.g., interoperability, standardized terminologies, RDF support, APIs, open-source status), each with a short definition plus per-platform entries coded as yes/no (0/1) and, where relevant, explanatory free text.

We leave it to readers of this overview to define their own priorities and assign weights to the various platform features. We suggest a simple scoring scheme on a 0–5 scale for each feature:

**Table:** 

Score	definition
0	don't care
1	low importance
2	moderate importance
3	important
4	very important
5	must have

If multiple people provide weights, these can be aggregated, for example using the minimum, maximum, mean, or median. An Excel worksheet (supplementary S18) is provided to support the creation of individual rankings. All features have been binary-annotated for the evaluated platforms (0: feature not present, 1: feature present). The score is calculated as the weighted (weights *w*) sum of all features (*f*): $\overset{n}{\underset{I=0}{\sum }}\;w_{i}f_{i}$. If necessary, absence of features can serve as an exclusion criterium. The next section 2.1 is included to provide a practical framework for translating broad platform concepts into decision-relevant requirements. For example, in the context of requests for proposals (RFPs), platform selection cannot rely on high-level labels alone, but requires a structured understanding of the functional differences. The definitions and distinctions introduced here therefore serve as an orientation tool for specifying requirements, comparing candidate systems, and aligning technical platform characteristics with the needs of concrete translational research use cases.

### 2.1 Definitions and main principles

Definition: A Medical Research Data Platform (MRDP) as discussed in this review is a digital infrastructure that facilitates the collection, storage, management and analysis of health-related data for research purposes. It enables researchers to access, share, and collaborate on data from various sources, enhancing the efficiency and effectiveness of translational medical studies and clinical trials.

Whilst this definition leaves room for a wide range of implementations, we further characterize MRDPs by the following features:

#### 2.1.1 Data collection and storage

Raw Data can be uploaded to the platform; data are versioned and secured (backup); full provenance is enabled. Connectors and importer tools exist for various types of data, including clinical, imaging and multi-omics data. Technical interoperability with other systems (e.g., by adhering to fundamental data exchange protocols) is enabled and “siloing” of data is actively avoided. The integration of various types of real-world data (including digital endpoints and measurements generated by digital devices) is made possible.

#### 2.1.2 Support of data governance and data security

Access to data is controlled by role-based access control (RBAC) and management of permissions to view, edit or process data is fully developed. Data usage is monitored, and a corresponding protocol is written (full provenance); the same is performed for data analytics workflows. Anonymization services are supported, and the anonymization status of data is made transparent. Compliance of data and analyses to HIPAA and/or GDPR is made explicit and ethics committee statements are linked to data sets. Data security and data protection measures are “features by design”; continuous intrusion detection is part of the security design.

#### 2.1.3 Data management, curation and harmonization

Data integrity checks and fundamental data statistics (e.g., data missingness) are built-in features. Compliance of data formats with widely accepted data exchange standards can be assessed. Data can be pre-processed, (re-)annotated and harmonized inside the platform (editing- and curation interfaces) along with continuous documentation of the applied changes. Metadata templates (controlled vocabularies; ontologies; terminologies) can be invoked (e.g., from resources taking care of shared semantics in the application field). Data Stewardship is supported and similarity assessment of data sets (“equivalence statements” through intelligent data stewardship tools) is enabled. Data can be mapped to Common Data Model (CDM)/public standard models for data persistence (e.g., OMOP, openEHR) or to data models for communication (e.g., FHIR, HL7, DICOM) with support by AI.

#### 2.1.4 Support for analytics

Advanced analytics is enabled through the integration of BioConductor/Python/R packages. Pre-trained models from other research labs can be imported and re-used. Analysis pipelines generate reports and write their own protocol. Functional interpretation of “signals” and “patterns” by dedicated algorithms (e.g., Gene Set Enrichment, Mechanism-enrichment) is enabled.

#### 2.1.5 Collaboration tools

The environment facilitates the collaboration between researchers (e.g., shared workspace; but also sharing of models and methods/workflows). Collaborative analytics is enabled through sharing analysis pipelines, functional interpretation services and pre-trained models. The latter belongs to the “federated learning” subsection of platforms and may not be applicable for all platforms. Discussion forums, internal communications of results (e.g., between collaboration partners); and data management in entire consortia are supported.

#### 2.1.6 Patient engagement, participatory research and representativeness

One of the goals of an advanced MRDP is to implement tools in the future that allow to involve patients in research and to support patient-oriented governance (e.g., by enabling deletion of data according to requests by study participants). Future patient engagement strategies will also see support for direct interaction with patients (e.g., lifestyle and behavioral coaching for prevention). Future visions also comprise services that allow for the integration of more diverse populations as well as of self-reported outcomes, ultimately leading to scenarios that allow study participants to identify “patients-like-me”. These strategies will require a different level of usability and UX for non-technical users (4–6).

#### 2.1.7 Interaction with healthcare professionals

One of the goals of an advanced MRDPs is enabling healthcare professionals to make use of modeling and mining output. Future implementations will most likely include recommender systems based on personalized risk models, allowing a medical practitioner to search for “patients-similar-to-this-one (my patient here)”. Ultimately, future-oriented platforms should support the guided interaction of healthcare professionals with data, knowledge, guidelines and evidence. We need to distinguish here between direct impact on clinical intervention (which would also require medical device certification on the data platform) or true research-only or clinical routine process support.

#### 2.1.8 Visualization

Advanced MRDPs will comprise Interactive Dashboards to support a “360 degree” visualization of data, trends, model output and the result of advanced analytics workflows. Visual methods may also enable the enactment of services to analytics workflows in an advanced analytics environment. Yet other visualization services will deal with mapping and curation tasks. Finally, visualization of longitudinal aspects (e.g., progression of a disease) is mandatory and will play a key role.

#### 2.1.9 Applied legal & ethics

Advanced and translational medical research data management is based on full compliance with data protection concepts for the platform. In addition, there are numerous examples where “public” study data sets are only shared based on agreements that require seamless documentation of data usage (and purpose) and full provenance of data analytics (see e.g., DPUK, Dementias Platform UK^10^). Furthermore, it requires dedicated management of approvals from ethics committees and documentation of technical organizational measures (TOMs) required by data protection legislation. Conventional models of informed consent, originally developed for traditional medical research such as clinical trials, may be inadequate for data platforms. These consent models often do not account for the dynamic nature, evolving applications, and novel research designs characteristic of platform/based research. Consequently, platform-based approaches necessitate a reimagining of informed consent practices. Alerting functions for regular training of researchers involved in patient-level data handling are not yet part of the systems on the market; nonetheless this functionality will need to be implemented rather soon as monitoring of regular ethics and legal training is increasingly regarded as an essential part of data governance today (7–9).

#### 2.1.10 Relevant data modalities

Multi-omics is a term coined for measurement of multiple modalities and endpoints in the same experiment. Initially branded for nucleotide analysis [DNA, (genetics, epigenetics)] and RNA (gene expression, miRNAs and other regulatory RNAs), this term is now also extending towards proteomics analyses, biomarker measurements, metabolomics and medical imaging (e.g., in the context of histology). These measurables are then correlated with clinical data such as diagnoses, clinical treatments and patient outcomes. For our systematic overview we present here, we consider a wide spectrum of modalities that have been summarized in Table 1. This modality list comprises clinical, genomics, imaging, phenotypic, medication and laboratory data. It also includes options for digital endpoints (e.g., from sensors; handhelds; smartphones).

**Table 1:** Data modalities to be considered along with relevant examples.

Group	Data Modality	Description	Example
Clinical Data	Clinical	All clinical data that we find in FHIR and/or OMOP core variables	Patient visits, diagnoses
	Medication	Prescriptions and compliance with prescriptions, known side effects or allergic reactions	Prescribed medications, adherence reports
	Laboratory	Typically blood analytics, but also special medical laboratory data	Blood test results, urine analysis
	Longitudinal	Visits, time courses; longitudinal studies; progression, trajectories	Patient follow-up visits over time
	Phenotypic	Clinical phenotypes, diagnoses linked to phenotypes, etc.	Diagnoses of diabetes, hypertension
	Family History	Inherited diseases and conditions	Family history of e.g., breast cancer
	Behavioural	Habits; sleep behaviour; psychiatric conditions; mental health	Smoking status, depression screenings
Molecular Data	Genomic	Exomes, whole genomes, cancer mutation panels, transcriptomes, proteomes, epigenomes	Whole genome sequencing results
	Biomarker	Dedicated biomarker information/measurements	PSA levels, BRCA1/2 mutation status
Imaging Data	Imaging	All types of imaging, including tomography, x-ray, histology, ultrasound	MRI scans, x-ray images
	Microscopic imaging	All types of imaging from microscopes, including histology arrays, video-imaging of live cells	Histology slides, video of live cell behavior
Electrophysiological Data	EEG, MEG	All types of electrophysiology data	EEG data coming from electrodes
Environmental Data	Environmental	Environmental factors including work conditions; stress	Air quality index, workplace stress surveys
Socioeconomic Data	Socioeconomic	Data on socioeconomic situation	Household income, education level
Survey Data	Survey/PROMs	Questionnaires of various types; interviews and patient reported outcomes	Patient satisfaction surveys, PROMIS questionnaires
Digital Data	Digital, wearables	Readouts from smart phones, smart glasses, sensors, Fitness Trackers, etc.	Activity levels from a Fitness Trackers, GPS data
Patient Path Data	Patient Path	Patient path; patient journeys; patient trajectories (time series)	Patient journey maps, care pathway analyses

In the following section we introduce definitions to distinguish platform types that address different technical and methodological problems within the biomedical data stack.

### 2.2 Definition of platform classes

Data warehouses, clinical data warehouses, and data lakes primarily concern the ingestion, storage, organization, and querying of heterogeneous data; data sharing platforms add the functions of governed discovery, access, and reuse; machine-learning platforms focus on the model lifecycle, including experiment tracking, training, deployment, and monitoring; and federated-learning platforms specifically support distributed model training across institutions while keeping raw data at the local site. The distinction is also justified by different data-movement logics: centralized hosting models bring data into a managed environment, whereas federated approaches move code or model updates to the data, which has direct implications for interoperability, privacy, governance, and legal compliance. Comparative work in life-science data sharing shows that centralized approaches tend to facilitate data linkage, harmonization, and interoperability, whereas federated approaches better preserve local control and can ease scaling and compliance. At the same time, these categories imply different evaluation criteria, which is a further methodological reason to keep them separate. However, the current taxonomy also combines two classification axes: some categories describe function (hosting, sharing, machine learning), whereas others describe topology (centralized vs. federated).

The five platform classes are conceptually stacked and build on each other from bottom to top: a pure *data hosting platform* (*Class 1*) primarily provides storage and access, while *data sharing platforms* (*Class 2*) additionally address interoperability and collaboration. *Data analysis platforms* (*Class 3*) extend this with integrated analysis and visualization capabilities; *machine learning platforms* (*Class 4*) add structured ML workflows and model management; and *federated learning platforms* (*Class 5*) finally complement this with distributed training and privacy-enhancing technologies. In practice, however, not all platforms in a higher class fulfill all characteristics of the respective “lower” classes—for example, there may be ML platforms that offer only minimal sharing functionality, or federated learning platforms whose data hosting capabilities are very limited. The boundaries between the classes are therefore fluid, and individual systems may combine features of several classes without fitting neatly into a single category.

#### 2.2.1 Class 1. Data hosting platform (data warehouse/data lake)

A data hosting platform refers to a service or infrastructure that integrates data from diverse sources and stores, manages, and provides access to it for users or applications. It can be cloud-based or on-premises and typically includes features such as data storage and backup, data security and access control, scalability (to accommodate growing data needs), search and retrieval functionalities, general data management support and special services for various data types and formats.

#### 2.2.2 Class 2. Data sharing platform

A data sharing platform is a system or service that enables the exchange and joint use of data among users, organizations, or applications in a secure and efficient manner. Data Integration allows for combining and harmonizing data from various sources for comprehensive sharing and analysis. Dedicated Interoperability Services support different data formats and standards to facilitate seamless data exchange. Advanced data sharing platforms comprise collaboration tools which allow users to cooperate on data-related projects or analyses. Finally, security- and compliance-services ensure that data sharing is compliant with regulations and maintains data privacy.

#### 2.2.3 Class 3. Data analysis platform

A data analysis platform is a software environment or toolset that enables users to collect, process, and analyse data to extract insights and make informed decisions. Key features of a data analysis platform include tools for cleaning, transforming, and other pre-processing of data for analysis. In addition, a data analysis platform comprises functions to perform statistical tests and statistical power estimates on data sets. Data analysis platforms typically have visualization options that allow to create graphs, charts, and dashboards for data representation. Advanced data analysis platforms support building and deploying machine learning models for predictive analysis. This can be done in collaboration, with options for sharing results and collaborating with others on data analysis projects.

#### 2.2.4 Class 4. Machine learning platform (local learning + central learning)

A machine learning platform is a software environment or framework that provides tools and services for building, training, deploying, and managing ML models. Key features of such a platform include tools and services for data cleaning, transformation, and feature engineering. Typically, these types of platforms support the application of various algorithms and libraries for creating ML models. Amongst the typical features are facilities for training models on large datasets and evaluating their performance. Advanced ML platforms provide tools for monitoring model performance and managing model lifecycle. There is a clear need for privacy preservation in training models from clinical data. That can be guaranteed on both ends: 1) the data providing system can exhibit (hardware-/software- based) secure compute environments, and/or 2) the ML/ deep learning (DL) method can employ source obfuscation techniques.

#### 2.2.5 Class 5. Federated learning platform

A federated learning (FL) platform is a ML framework that enables multiple decentralized devices or servers to collaboratively train models on their local data without sharing the actual data with a central server. Key features of this type of platform include enhanced data privacy through keeping user data on local servers, enhancing privacy and compliance with data protection regulations. Using a usually central custodian or server, collaborative learning aggregates model updates from various participants to build a global model while maintaining data locality. Federated learning platforms support training across numerous devices, making it suitable for applications with large-scale data. A key feature of federated learning platforms is communication efficiency, minimizing data transmission by sharing only model updates rather than raw data. For federated evaluation, the global model is distributed to clients and evaluated on their local test datasets, and the resulting metrics are then aggregated. One can use this approach to evaluate a third-party vendor's model on health systems’ local data- which is meaningful to test the generalizability of the commercial models. Privacy Enhancing Technologies: While training models on such confidential data can be highly beneficial, it also comes with huge privacy risks. The predictive models can establish a strong correlation between features and labels, which coincides exactly with what an adversary exploits to mount model inversion (MI) attacks. MI attacks aim to reconstruct sensitive features of training data by exploiting their correlation with the model output. One of the solutions that is studied in computer science and validated in many settings is Differential Privacy (DP), which carefully randomizes an algorithm so that its output does not depend too much on any individual's data. In the context of AI algorithms, DP guarantees protection against attempts to infer whether a data record is included in the training set from the trained model.

A SWARM learning platform is a specialized form of FL based on a decentralized approach to ML that allows multiple devices or nodes to collaboratively train models while sharing only model parameters and not the raw data. SWARM learning transforms the paradigm from data sharing to data visiting. It leverages principles from swarm intelligence, enabling collective learning from distributed data sources. Key features include decentralization which eliminates the need for a central server, allowing nodes to learn and improve independently. In contrast to many conventional federated learning platforms that rely on a central coordinator, Swarm Learning employs a blockchain-based, dynamically coordinated aggregation mechanism, while—like other federated approaches—keeping data local at each node and sharing only model parameters. SWARM learning establishes a sort of collective intelligence by utilizing the collective learning from various nodes to enhance model accuracy and performance.

### 2.3 Overview of selected platforms characterized for MED-connect

From the initial list of platforms found on the internet we selected a variety of solutions based on their significance for the intended objective of this overview (c.f. inclusion/exclusion criteria above). We are fully aware that we may have missed one or the other system that should have made it into the detailed characterization. However, we have endeavored to contact many providers to ensure an almost complete overview.

The selected solutions for detailed analysis are listed in Table 2. The table is a catalog of medical research data platforms, listed in alphabetical order and labeled with a “Supplementary” identifier (S4-S17) for the questionnaires filled in by the vendors. For each platform, it provides its name, a link to technical or user documentation (when available), and key publications that describe or use the platform.

**Table 2:** List of all considered platforms.

Supplementary number	Name of platform (alphabetic order)	URL to the documentation	publications
S4	42Hills Swarm Learning (42HSL)		(28–30)
S5	Apheris Compute Gateway	https://www.apheris.com/docs/	(31)
^a^	Aridhia		
S6	BC Platforms	https://www.bcplatforms.com/resource-center	
S7	DigiMed Secure Cloud	https://digimed.pages.gitlab.lrz.de/user-documentation/	(25, 32)
S8	DNAnexus	https://documentation.dnanexus.com/	(33–39)
S9	HiGHmed HIVE Suite, (openEHR-compatible CDR)	https://www.highmed.org/en/home	(40, 41)
S10	Honic	https://honic.eu/en/honics-core-pillars-data-privacy-and-data-security	
S11^b^	I2B2	https://www.i2b2.org/about/intro.html	(42–46)
S12	IDERHA	https://www.iderha.org/about	(47–50)
S13	Indoc Systems Pilot Data Management Platform	https://www.indocsystems.com/pilot	(51–54)
S14	IQVIA / KAIROS Health Data Research Platform	https://www.iqvia.com/locations/emea/iqvia-connected-healthcare-platform/iqvia-health-data-research-platform	(55, 56)
S15	NVIDIA FLARE	https://github.com/NVIDIA/NVFlare	(71)
S16	Rhino Federated Computing Platform (Rhino FCP)	https://docs.rhinohealth.com/hc/en-us	(57–62)
^a^	Siemens Health.		
S17	Virtual Research Environment (VRE)	https://vre-docs.charite.de/	(63–66)

a requested.

b annotated by public info, references have been extracted from the filled form sheets and imported into Zoter**o.**

**Figure 1:**
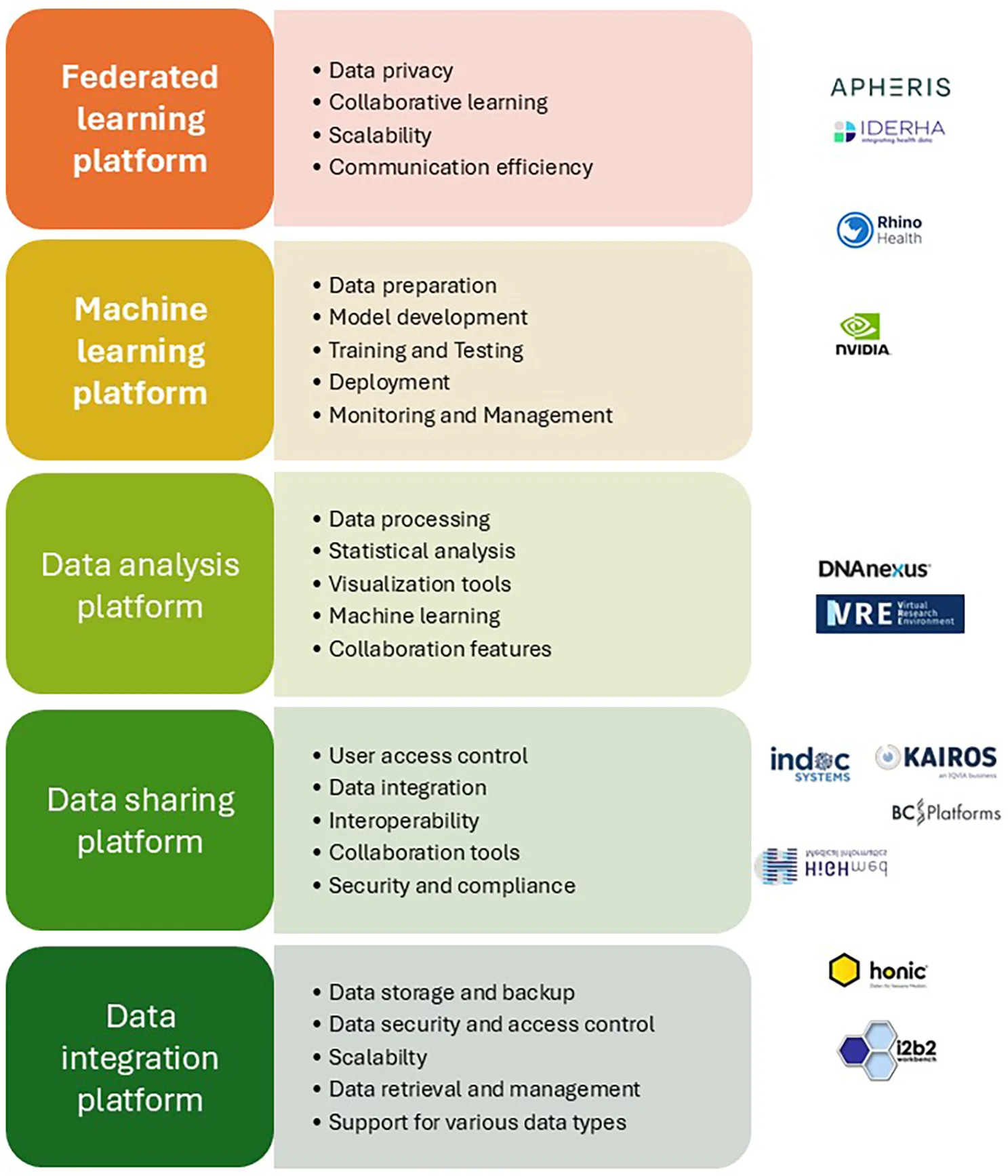
Shows the different levels of medical research data management platforms and maps individual vendors or platform.

A complete list of all platforms, their features and their functionalities is provided in a comprehensive table in the Supplementary Material S3 provided with this review. The different types of MRDP systems as defined in section 2.2 are shown below in Figure 1. The figure presents five hierarchical levels of medical research data management platforms, arranged from bottom to top. Each level lists key functions and shows example vendors mapped to that category. Each platform is assigned to a class based on which “layer” of functionality dominates its feature profile in the aggregated factsheets, not on whether it implements every possible feature of that class.

- Data integration/hosting platforms (e.g., HONIC, some data integration centers) primarily provide secure, scalable storage, pseudonymization and linkage, and standardized ingestion (OMOP, FHIR, openEHR), but have limited built-in analysis or ML functionality. Their strengths in encryption, RBAC, cloud/on-prem deployment, and handling very large datasets match the definition of a data hosting layer.
- Data sharing platforms (e.g., BC Platforms, HiGHmed, Pilot/INDOC) add strong interoperability, metadata/ontology support, cohort discovery, and shared workspaces on top of hosting. They emphasize federated cohort queries, multi-site governance, role-based permissions and collaboration tools, which are characteristic of platforms enabling controlled data exchange between institutions.
- Data analysis platforms (e.g., VRE, DNA Nexus, HDRP in some deployments) offer rich analysis workbenches: web UIs, scripting (R, Python, Jupyter), dashboards (Superset), cohort queries, and visualization, but may not enforce a specific ML lifecycle or federated training model. Their factsheets highlight HPC integration, GPU VMs, interactive notebooks, and BI tooling as core capabilities.
- Machine learning platforms (e.g., NVIDIA Clara, parts of DNA Nexus, some HIVE-based solutions) go beyond generic analysis by explicitly supporting model training, deployment and monitoring, GPU-accelerated workflows, and integration with ML frameworks. They provide MLOps-like components (model registries, harmonization copilot, GPU clouds) while still operating in a predominantly central-learning setup.
- Federated learning platforms (e.g., NVIDIA FLARE, Rhino FCP, Apheris, 42HSL, IDERHA) are characterized by decentralised training across multiple nodes, strong data sovereignty (data never leaves local sites), orchestration of jobs to edge/clients, and privacy-enhancing technologies such as differential privacy, homomorphic encryption and secure enclaves. Their factsheets repeatedly stress “data is only visited”, central orchestration with local execution, federated model aggregation, and support for large multi-institution networks.

Because the classes are conceptual layers, there are overlaps and grey zones: some FL platforms offer only minimal data-hosting functions, and some data sharing platforms already integrate substantial ML features. In such cases, the assignment in Figure 1 reflects the primary intended use and the strongest cluster of features in the matrix, rather than a strict, exclusive classification.

### 2.4 Referential implementations and utility

Most platforms listed here in this overview are part of referential installations at a global level. These reference implementations address different purposes and represent tailored solutions. Examples for referential installations are listed in Table 3. The table lists real-world reference installations for several medical research data platforms and briefly describes each use case. It has four columns: platform/vendor name, reference installation, a short description of that installation, and publication references.

**Table 3:** Examples for referential installations.

Vendor / System	Reference Installation	Description	Publication reference
Apheris Compute Gateway	INTONATE MS Consortium	INTONATE-MS is a public–private research consortium between Universitätsklinikum Münster, Penn Medicine, Unity Health Toronto, Erasmus MC and Roche.	(67)
	AISB Consortium	The AISB Consortium brings together biopharmaceutical companies and cutting-edge federated learning technology to harness the collective expertise and data of industry leaders to push the boundaries of AI-driven drug discovery.	(68)
DNAnexus	UK BioBank		
Honic	German RWD ecosystem platform	Harmonised, FHIR-structured, linkable data pools on digital sovereign, GDPR-compliant platform open for all researchers, first implementations as trusted research environment for third parties ongoing	(22)
IQVIA / KAIROS	German National Cohort	https://nako.de/en/	(69)
Indoc Systems / Pilot	Brain-CODE; Breakthrough Discoveries for thriving with Bipolar Disorder (BD2);	“These Pilot-based platforms enable secure management, integration, analysis, and sharing of multi-modal data collected across multiple research centres.”	(53, 54)
BCplatforms	UK Biobank	Installation to pharma company intranet+	
SWANSEA	DPUK	Dementias Platform UK	(70)
RHINO Federated Computing	EDRN Consortium	Data Farber Cancer Institute and its EDRN consortium members - Federated Learning for Healthcare NLP model development for predicting clinical events. By training models across multiple institutions without centralizing data, the framework enhances predictive performance while maintaining patient privacy.	
Virtual Research Environment (VRE)	Charité Virtual Research Environment^a^; EBRAINS Health Data Cloud^b^; VGREGION Göteborg, Sweden (on-premise, hospital internal)	General-purpose processing platform for personal data under the scope of GDPR	(71)

a https://hdc.ebrains.eu/

b https://hdc.humanbrainproject.eu/

## 3 Results

### 3.1 Literature search

Our search identified a broad and heterogeneous landscape of health data platforms (Supplementary S1). PubMed queries yielded 25 platforms, mainly academic initiatives with a strong focus on disease- or modality-specific research (e.g., REDCap for research data capture, TB Portals for global tuberculosis data, ImmPort for immunology, EMIF as a European federated data infrastructure). Google searches added 28 predominantly commercial platforms, many of them US-based cloud or interoperability offerings (e.g., Snowflake Health Data Platform, Datavant, Optum Data Exchange, Ambra Health for imaging). From Google Scholar, we retrieved 49 additional, mostly academic platforms, including numerous solutions dedicated to medical image sharing and management (e.g., XNAT, OHIF, Dicoogle, Orthanc, various PACS servers). Finally, using ChatGPT we compiled extended lists of vendor- and standard-driven ecosystems across several categories, including Electronic Health Records (8 major EHR vendors such as Epic and Cerner), Health Information Exchange networks (10), cloud services for healthcare (9), research-focused platforms (19), personal health record solutions (7), and generic data-sharing repositories (46).

### 3.2 Choosing a weighting scheme

**Figure 2:**
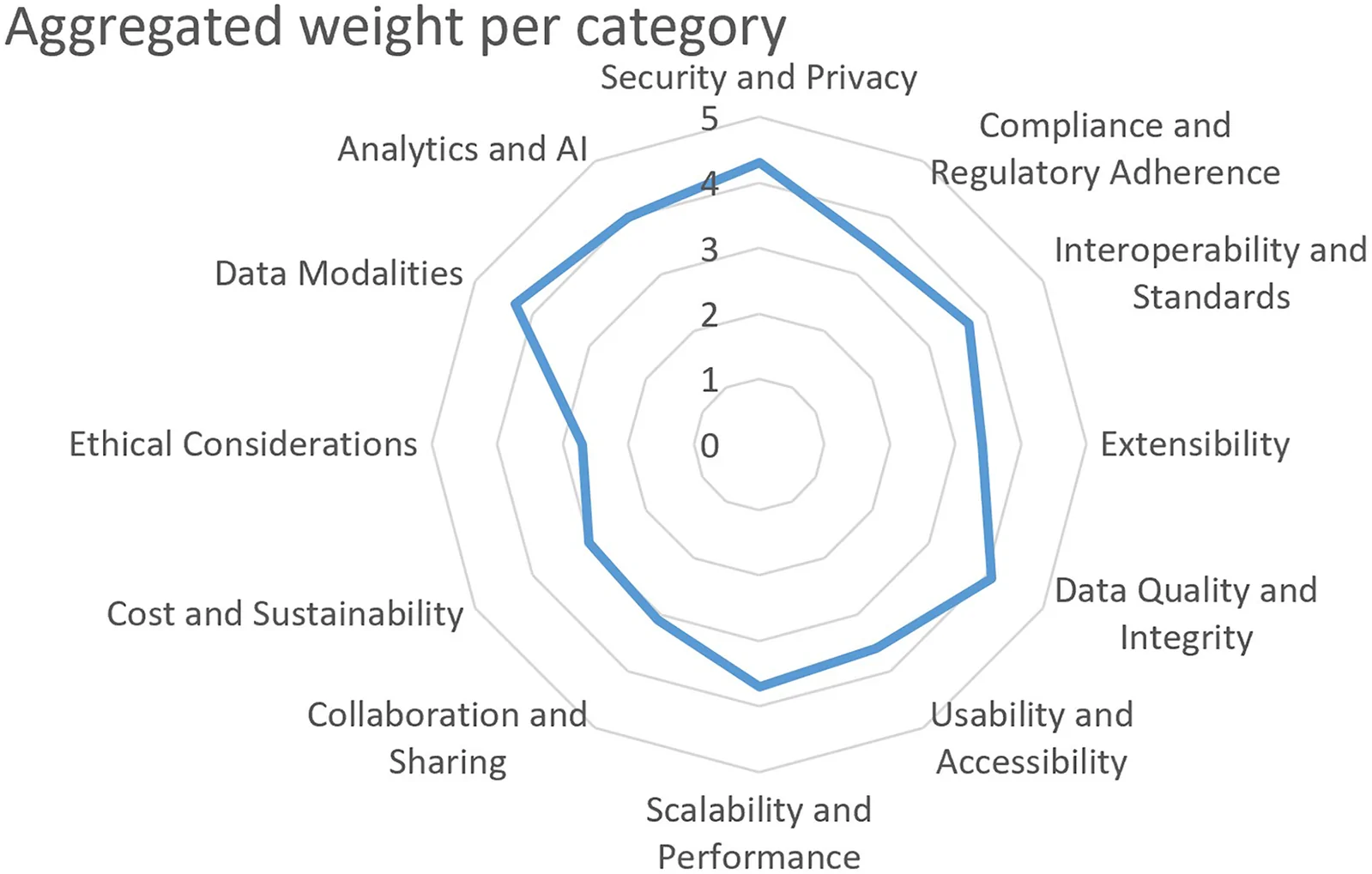
Aggregated importance weights for 12 platform selection categories derived from the Med-Connect partner survey, shown as a radar chart with highest weights for security, privacy, interoperability, data quality, multimodal data support, analytics and AI.

We asked the five MED-Connect partners to assign weights to platform selection features based on the scoring approach described in Section 2. The resulting aggregated importance weights, derived from the partner survey, are presented in Figure 2 as a radar chart spanning 12 categories. Each axis corresponds to a criteria block: Security and Privacy, Compliance and Regulatory Adherence, Interoperability and Standards, Extensibility, Data Quality and Integrity, Usability and Accessibility, Scalability and Performance, Collaboration and Sharing, Cost and Sustainability, Ethical Considerations, Data Modalities, and Analytics and AI. The plotted values correspond to the median feature weights per category (0–5 scale, 0 = “don’t care”, 5 = “must have”) aggregated across all five partners, illustrating a common emphasis on security, interoperability, data quality, multimodal data support, and analytics, with comparatively lower but still non-zero weights for cost, sustainability, and ethical governance.

To derive a final scoring scheme we tested the minimum, maximum, average and median on the weights of the partners. The partners show broadly aligned but not identical priorities across criteria, with security, interoperability, data quality, and multimodal data support consistently ranked highest, and aspects like configuration management, multiple instances, and advanced semantic search receiving lower weights. Overall scores are tightly clustered (total medians 205–295), so the choice of aggregation function (average vs. median vs. min vs. max) mainly shifts emphasis on outlier opinions rather than changing the qualitative picture.

Across partners, core security (encryption in transit/storage, RBAC) is rated uniformly very high (median 5; min–max 0/5 only where someone uses “disqualifies” or does not score), underlining that basic protection and access control are seen as non-negotiable baseline requirements. Features such as secure cloud deployment and on-premise operation show more spread (medians around 3–4, min as low as 2), suggesting different infrastructure preferences between partners (cloud-first vs. on-prem-oriented).

For regulatory aspects, GDPR compliance is weighted very strongly (median 5), whereas HIPAA shows lower and more variable scores (median 2, min 0, max 5), reflecting a predominantly European focus with only some partners considering US regulation critical.

Standard-based interoperability (FHIR, DICOM, OMOP, openEHR, standardized terminologies) is weighted systematically high with medians around 4–5 and small ranges, indicating strong consensus that standards support is central for platform selection. Extensibility features (APIs, data export workflows, 3rd-party integration) are also valued (medians 3–5), whereas open source and modular design have more heterogeneous importance (some partners score them 5, others only 2), so their overall medians settle around 2–3.

In data quality and integrity, data validation, audit trails, data provenance, FAIR support and CDM mapping all receive high median scores (mostly 4–5) with only modest variation, emphasizing a shared preference for platforms that support reproducible, well-governed data management. NLP and ontology support are seen as relevant but not essential for all use cases (medians 3, with min 2 and max 4), pointing to more specialized needs.

Usability aspects such as a web frontend, documentation, training, and support are consistently rated important (medians mostly 4–5), but customized forms/views and advanced search (faceted, semantic) show more mixed ratings (medians around 2–3), indicating that not every partner requires complex end-user interfaces from the core platform. Scalability for large data volumes and many concurrent users is considered important (medians 4–5), while monitoring receives slightly lower and more dispersed scores (median 3).

For collaboration and sharing, federated queries and shared data access have relatively high medians (3–4), but “multiple instances” has a very low median (0) and small average, reflecting that only one or two partners explicitly value multi-instance, loosely coupled deployments; most focus more on logical federation and access control within a single platform.

Long-term support and regular release cycles are clearly valued (medians 3–4), and “out-of-the-box installation” is considered moderately important, with one partner rating it very low and another very high, leading to a median of 3. Configuration management, in contrast, has low scores across partners (median 1), suggesting that sophisticated configuration tooling is seen as “nice to have” rather than a key discriminator.

Regarding data modalities, clinical routine data, imaging, phenotypic data, OMICs, and medication/lab data are consistently prioritized (medians 4–5), whereas lifestyle, sensor/wearable data and PROMs show more variability but still achieve mid-to-high medians (3–4). In analytics and AI, analysis workflows, visualization, reporting, scripting (R/Python) and application of ML models all have relatively high medians (4–5), while pure ML model building is slightly lower and more heterogeneous (median 3), reflecting that not all partners expect full ML development environments as part of the platform.

### 3.3 WE have studied the effect of aggregation method (avg, median, min, max)

Using the median for each feature, as done in the “Score (median)” column, dampens individual outliers and produces robust, mid-to-high scores for widely agreed-upon features (e.g., encryption, standards support, FAIR, key modalities), while down-weighting features that only one partner cares strongly about (e.g., multiple instances, strong emphasis on open source). A simple average leads to very similar rankings in this dataset because partner scores are fairly clustered, but slightly inflates the influence of extreme values (e.g., when one partner rates 0 and another 5).

By contrast, min and max emphasize disagreement: the min score highlights “deal-breaker” views (e.g., some partners scoring 0 for HIPAA, semantic search, multiple instances), which would heavily penalize platforms lacking any one partner's must-have feature; the max score, in turn, favors features that at least one partner sees as critical, even if others assign them low importance. In practice, using the median offers a balanced compromise between these perspectives, yielding a ranking that reflects shared priorities across the MED-Connect consortium rather than the preferences of any single institution.

The weighting profiles of the five MED-Connect partners are best described in aggregated, anonymous terms, because their preferences are strongly overlapping and differ mainly in emphasis rather than in fundamentally opposed priorities. Overall, partners converge on a common core: high importance for security, standard-based interoperability, data quality, rich multimodal data support, and integrated analytics, while factors like configuration comfort, multiple deployment instances, or advanced semantic search are seen as secondary.

Across all criteria, median scores are typically in the mid-to-upper range (3–5), and the total per-partner scores (not shown individually) occupy a relatively narrow band, indicating that no partner systematically departs from the consortium consensus. The main differences arise in a few specialized areas (e.g., HIPAA vs. GDPR relevance, open-source and modularity, multi-instance deployment, configuration management), where some partners assign very high weights and others low ones; these appear as wider min–max ranges while the median stays moderate.

Using the median as the aggregation function therefore produces a robust “consensus profile” that highlights broadly shared priorities and avoids over-emphasizing outlier views. Averages yield a very similar picture but are slightly more sensitive to extreme ratings, whereas min and max would shift the selection logic towards “veto power” (min) or “at least one strong advocate” (max), which is less suitable for representing a joint consortium perspective.

### 3.4 Scoring and ranking of platforms

**Figure 3:**
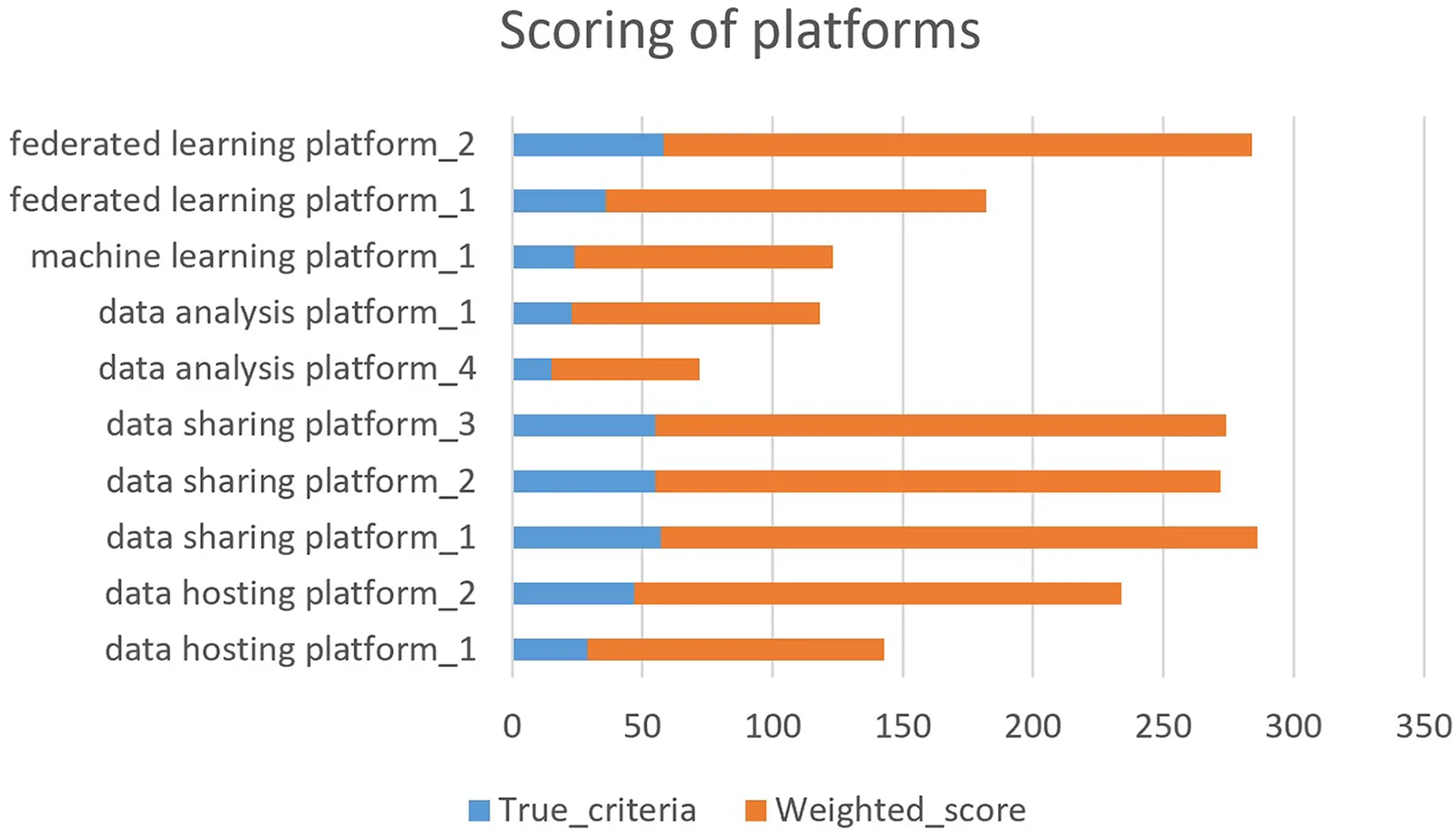
Scoring of selected medical research data platforms across five functional classes, displaying the number of fulfilled criteria and the corresponding requirement-weighted scores for each anonymized platform instance.

Based on the questionnaire results, nine commercial platforms and one open-source solution currently deployed in Europe were selected for scoring and ranking. The resulting ranking reveals a clear progression from general-purpose data hosting and integration platforms to more advanced (federated) learning platforms, both in terms of the number of criteria fulfilled and the overall weighted scores. Broadly, three tiers can be distinguished. Figure 3 presents the scoring of selected medical research data platforms across five functional classes. For each anonymized platform instance—data hosting, data sharing, data analysis, machine learning, and federated learning—the horizontal bars indicate the number of fulfilled criteria (“True_criteria,” shown in blue) alongside the corresponding weighted score derived from the MED-Connect consortium's feature weights (“Weighted_score,” shown in orange).

Platforms in higher-level functional classes (data analysis, machine learning, and federated learning) generally meet a larger number of criteria and achieve higher weighted scores. In contrast, lower-level hosting and sharing platforms address a more limited subset of requirements, highlighting how increasing functional scope and sophistication are reflected in the aggregated scoring.

#### 3.4.1 Top tier: most complete, feature-rich platforms

The top group comprises platforms that satisfy a very high number of criteria (around 55–58 “true” items) and at the same time achieve the highest weighted scores (around 217–229 points). These systems belong to the “data analysis/machine learning” or “federated learning” classes and combine broad interoperability, extensive workflows, multimodal data support, and advanced analytics/ML/FL capabilities. They are therefore closest to an “all-in-one” approach for distributed medical data analysis.

#### 3.4.2 Middle tier: strong but partly specialised platforms

A second group achieves 36–47 satisfied criteria with weighted scores in the range of 146–219 points. Functionally, these platforms lie between classic data integration/sharing solutions and fully developed analysis or FL stacks and are typically classified as “data sharing/data analysis/ML platforms.” They offer solid security, interoperability, and analysis features but are either less broad in overall functionality or more strongly focused on specific application scenarios.

#### 3.4.3 Lower tier: focused, often domain-specific platforms

The lower group satisfies clearly fewer criteria (15–29 “true” items) and shows correspondingly lower weighted scores (57–114 points). These solutions belong mainly to the “data hosting” or narrowly focused “data sharing” categories and usually address particular data types, clinical domains, or infrastructure aspects without covering the full spectrum of interoperability, workflow, and ML features.

Taken together, the ranking indicates that higher-level classes (analysis, ML, federated learning platforms) tend to meet more criteria and score better overall, whereas lower-level classes selectively cover only parts of the requirement catalogue.

## 4 Discussion

Digital infrastructures supporting translational medical research are evolving at a breathtaking pace. MRDPs form the core of data and innovation ecosystems and need to keep up with the ever-growing capacity and performance of laboratory technologies.

Given the heterogeneity observed in the domain of medical research data and the dynamics of multimodal data generation, we need to acknowledge that there is a significant number of remaining challenges for innovative data management and advanced analytics in translational medicine:

### 4.1 Ongoing challenges in the field

Although MRDPs have evolved from centralized data repositories toward distributed cloud-based and federated learning environments, several core challenges remain relevant across all platform types. One of the most fundamental issues is the establishment of shared semantics. Productive data ecosystems depend on the consistent use of terminologies, ontologies, metadata standards, and common data models so that heterogeneous datasets can be interpreted, harmonized, and reused across projects and institutions. Closely related to this is the need for intelligent data stewardship, including data cleansing, quality assessment, mapping to standards such as OMOP, openEHR, or disease-specific common data models, and the support of curation workflows by semi-automated or AI-assisted services. In this context, FAIRification should not be treated as an optional add-on, but as a design principle built into the platform architecture, ensuring that both internal and externally integrated data resources remain findable, accessible, interoperable, and reusable. Another central requirement is the management of primary data together with their derivatives, for example when imaging features, transformed omics profiles, or analysis outputs must remain linked to the original source data with complete provenance tracking.

Beyond data integration itself, major challenges arise from the workflows that transform raw data into curated and analysis-ready research assets. Platforms need to support interoperability assessment, harmonization, ETL processes, annotation, and provenance-aware curation in a way that is transparent, reproducible, and auditable. This also includes support for audit compliance, certification, and productive use in regulated environments. The way information can be queried, exported, and represented is equally important, especially where data should become directly usable for advanced analytics or AI-driven workflows (10, 11). Many use cases further require secure on-platform processing environments in which sensitive data can be analysed without leaving the protected environment. In addition, platforms differ substantially in the extent to which they support modeling and mining workflows, integrate external analysis pipelines, or enable the inclusion of prior biomedical knowledge such as pathway databases, disease maps, or graph-based representations for functional interpretation of results.

Operational and organizational challenges are just as important as technical ones. The practical value of a platform depends on how easily it can be deployed, maintained, and ported across different hosting environments, including on-premises, cloud, and hybrid settings. Questions of staffing required human intervention, consistency management, and total cost of ownership therefore remain central selection criteria. Trust in such platforms also depends on secure deployment, intrusion detection, and robust safeguarding procedures, including regular vulnerability testing. Privacy remains a particularly critical issue in the era of AI, since even anonymized or pseudonymized data may become vulnerable through model inversion, reconstruction, or other forms of privacy leakage. Regulatory developments add another layer of complexity. In Europe, the EHDS framework is likely to reshape how health data can be requested, accessed, and reused for research, creating both new opportunities and substantial adaptation pressures for existing platforms (12, 13). None of the platforms we analyze here in this overview has fully adopted EHDS regulation, which is not surprising, as the regulation passed the EU parliament one year ago and is now on its way to be implemented in national law. The Sphin-X initiative (https://sphin-x.de/en/) provides an entire ecosystem to enable active networking and trusting cooperation across all areas of the healthcare system in Europe. The process of involving stakeholders across the entire healthcare system has just started and it remains to be shown that this initiative really catalyzes the discourse and best practice that is needed to make the EHDS an innovation-fostering framework. Finally, long-term adoption will depend not only on technical sophistication, but also on usability for non-technical stakeholders such as clinicians, patients, and patient organizations (14–17), as well as on the ability of platform-based AI tools to support clinical validation, regulatory readiness, compliance monitoring, and trustworthy deployment in real-world biomedical research settings.

### 4.2 Emerging technology trends

Beyond organizational and regulatory aspects, emerging technologies are reshaping the design space for medical research data platforms. Among these, blockchain-based approaches have attracted particular attention as a potential response to long-standing challenges of centralised architectures such as data silos, tampering risks, and limited transparency. Their core properties —immutability, decentralization, transparency, and auditability—enable secure, patient-centric control over sensitive research data while supporting compliance with regulations like HIPAA and GDPR (18).

Key applications of blockchain technology span from clinical trial data management (where verifiable audit trails are in the center of blockchain application strategies) via data sharing platforms (where blockchain enables fine-grained sharing amongst researchers, hospitals and sponsors while preserving privacy) to the management of Multi-Omics Data (where traceable provenance and compliance with data protection regulation are central features enabled through blockchain technology) (19).

For all these key application areas of blockchain technology, dedicated frameworks have been developed (e.g., BlockHealth (20), a hybrid framework using Ethereum, NFTs and Cassandra for off-chain storage).

Main benefits of adopting blockchain are enhanced data integrity and provenance tracking, patient – empowerment through consent-based sharing (and the ability of undoing enrollment in trials based on individual decisions by patients) (21). Reduced costs in audits are expected, but not yet systematically investigated. At the same time, blockchain is not a universal solution and introduces its own trade-offs in terms of scalability, latency, energy use, and integration with existing clinical infrastructures. More broadly, biomedical research is undergoing a shift towards multimodal, systems-medicine-oriented data ecosystems in which heterogeneity is the norm rather than an exception. In this setting, clear provenance of data processing and model use is increasingly a legal requirement, and professional handling of legal and ethical aspects of data analytics becomes a first-order design constraint rather than an afterthought. These trends underscore the need for rigorous, context-sensitive benchmarking and critical assessment, which we discuss in the following `.

### 4.3 Benchmarking and critical assessments in biomedicine

Benchmarking has become an important approach for the critical assessment of biomedical IT systems, ranging from data infrastructures to AI models in federated environments (22). While initial work has proposed benchmark datasets and scenarios for cross-silo federated learning in healthcare (23), existing efforts remain limited in scope and usually address only selected modalities or technical functions. They therefore do not reflect the full complexity of translational medical research. Because platform-selection decisions have substantial strategic and financial consequences, benchmarking of medical research data platforms should be based on tailored scenarios that match specific research contexts and requirements (24). One major challenge is the restricted availability of patient-level data for benchmarking (25). Synthetic clinical datasets (26, 27) provide a promising way forward by enabling realistic and privacy-preserving benchmark scenarios. We therefore propose the development of a repository of synthetic, high-dimensional translational research datasets that can support the evaluation of multimodal data handling, longitudinal data integration, interoperability, curation workflows, federated learning performance, and access to analytical tools and pipelines.

### 4.4 Limitations

A key limitation of this overview is that several platform descriptions were provided or reviewed by authors affiliated with the respective platforms. While this approach enabled access to detailed and up-to-date technical information, it also introduces a risk of self-reporting and presentation bias. The overview should therefore not be interpreted as an independent comparative assessment or formal benchmark of platform performance. In addition, platform inclusion was influenced by vendor responsiveness and willingness to participate, which may have favored more visible, established, or collaborative providers. Where possible, platform information was checked against publicly available documentation and publications, but independent technical verification and head-to-head benchmarking were beyond the scope of this work. The findings should therefore be read as a structured descriptive overview intended to support orientation and decision preparation, rather than as a definitive evaluation of comparative suitability or performance.

## 5 Conclusion

Major insights generated by this work on Medical Research Data Platforms.

The overview we present here has generated a couple of major insights that we would like to summarize here:

- **Multi-Stakeholder Approach:** the collaboration between academic and industrial stakeholders resulted in a systematic approach towards a comprehensive overview of Medical Research Data Platforms that support collaborative, data-driven, translational research.
- **Realism and pragmatism:** the questionnaire we generated pre-defined categories that were motivated by the aims of a real project aimed at setting up a collaborative platform within the Fraunhofer translational life sciences ecosystem (which comprises 7 Fraunhofer institutes in this particular case)
- **Public – private collaboration:** we generated an open “information partnership” that invited public and private stakeholders to share information about platforms; we managed to generate the trust in fairness and openness of the overall approach that convinced a diverse set of stakeholders to contribute and share relevant information
- **A classification system of types of platforms:** we came up with a classification schema plus definitions and utility profile of the various types of platforms that we could integrate in our overview. This greatly helps mapping requirements to platform features and integrated features.
- **Decision-support for third parties:** we generated a simple tool that enables other scientists or other organizations to assign weights to various aspects relevant for their usage scenario. This provides an unbiased way to identify platforms that display a good fit between the application use case motivating the individual lab or network and the features of the platforms.

## Data Availability

The original contributions presented in the study are included in the article/Supplementary Material, further inquiries can be directed to the corresponding author/s.

## Footnotes

^1^ https://www.dedalus.com/de/de/solutions/orbis/

^2^ https://www.cgm.com

^3^ https://www.epic.com/

^4^ https://www.oracle.com/health/

^5^ https://www.castoredc.com

^6^ https://www.project-redcap.org

^7^ https://www.iderha.org/

^8^ https://i2b2transmart.org/

^9^ https://ebrains.eu/

^10^ https://www.dementiasplatform.uk/
