# Correction: Selecting medical research data platforms for translational biomedical research: a five-tier overview and requirement-weighted assessment framework

**DOI:** 10.3389/fdgth.2026.2006683

**Published:** 2026-10-05

**Authors:** Marc Jacobs, Samad Goudarzi, Jan Stücke, Robin Röhm, Timo Kanninen, Tero Oinonen, Petra Ritter, Michael Schirner, Fabian Prasser, Arman Bhagwagar, Bernd Matzenbach, Hartmut Schultze, Joachim Schultze, Mathias Göschl, Henrik Matthies, Kenneth R. Evans, Moyez Dharsee, Christian Stephan, Marvin Belz, Philip Gribbon, Katja Herzog, Chester Chen, David Ruau, Ittai Dayan, Adrish Sannyasi, Dieter Kranzlmüller, Naweiluo Zhou, Jens Warfsmann, Martin Hoffmann, Rudi Schmidt, Martin Hofmann-Apitius

**Affiliations:** 1 Bioinformatics Department, Fraunhofer-Institute for Algorithms and Scientific Computing (SCAI), Sankt Augustin, Germany; 2 Apheris AI GmbH, Berlin, Germany; 3 BC Platforms, Espoo, Finland; 4 Berlin Institute of Health at Charité — Universitätsmedizin Berlin, Charité, Berlin, Germany; 5 Center of Health Data Sciences, BIH @ Charité, Berlin, Germany; 6 DNANexus, Mountain View, CA, United States; 7 Deutsches Zentrum für Neurodegenerative Erkrankungen, Bonn, Germany; 8 42Hills GmbH, Königswinter, Germany; 9 HiGHmed e.V., Heidelberg, Germany; 10 HONIC Health Data Technologies GmbH, Neckarsulm, Germany; 11 Indoc Systems, Toronto, ON, Canada; 12 IQVIA, Bochum, Germany; 13 Fraunhofer Institute for Translational Medicine and Pharmacology ITMP, Discovery Research ScreeningPort, Hamburg, Germany; 14 NVIDIA, Santa Clara, CA, United States; 15 RHINO Federated Computing, Boston, MA, United States; 16 Leibniz Supercomputing Centre (LRZ) of the Bavarian Academy of Sciences and Humanities, Garching, Germany; 17 Division of Personalized Tumor Therapy, Fraunhofer Institute for Toxicology and Experimental Medicine (ITEM), Regensburg, Germany; 18 Bonn-Aachen International Center for Information Technology (B-IT), University of Bonn, Bonn, Germany

**Keywords:** medical research data platforms, translational biomedical research, equirement-weighted assessment, data privacy, FAIR data, iteroperability, federated learning, data harmonization

The funders the EU Horizon Europe Programme through BRIDGE (101219311), EBRAINS2.0 (101147319), Virtual Brain Twin (101137289), EBRAINS-PREP (101079717), AISN (101057655), EBRAIN-Health (101058516), and EIC grant PHRASE (101058240); the Digital Europe Programme through TEF-Health (101100700), SHAIPED (101195135), and CoordinaTEF (101168074); the German Research Foundation (DFG) through SFB 1436 (Project ID 425899996), SFB 1315 (Project ID 327654276), SFB 936 (Project ID 178316478), SPP Computational Connectomics (RI 2073/6-1, RI 2073/10-2, and RI 2073/9-1), DFG Clinical Research Group BECAUSE-Y (504745852), the DFG German-Spanish project BrainsAge (RI 2073/14-1), and DFG Project ID 424778381 – TRR 295 Retune; ERA PerMed JTC2021; the German Federal Ministry for Health through PatternCog (ZMI5-2522FSB904); Berlin University Alliance OpenMake; and the Berlin Institute of Health and Foundation Charité to Petra Ritter were erroneously omitted.

The original version of this article has been updated.

