# Supplementary material for: Selecting medical research data platforms for translational biomedical research: a five-tier overview and requirement-weighted assessment framework
Source: Front Digit Health. 2026 Jun 17;8:1814015. doi: 10.3389/fdgth.2026.1814015 (PMC13319098; doi:10.3389/fdgth.2026.1814015)
Supplement: Supplementary file 2 [file Supplementaryfile2.docx]

***Rhino Federated Computing Platform***

***Deployment and Usage****:*

*The Rhino Federated Computing Platform (Rhino FCP) enables cross-silo data collaboration using federated learning, edge computing, and generative AI, eliminating the need for data transfers by allowing users to 'bring code to the data'. This code can range from queries to transformations to federated machine learning to actually running custom code on a set of decentralized data.*

***References:***

[***https://www.rhinohealth.com/resources#learn-publications***](https://www.rhinohealth.com/resources#learn-publications)

## Federated learning for predicting clinical outcomes in patients with COVID-19

Authors: Dayan I, et al.

Journal: Nature Medicine (2021)

Highlight: Largest federated learning study to date, conducted across 20 institutions worldwide.

Link: <https://www.nature.com/articles/s41591-021-01506-3>

## Multi-center Study of a Deep Learning Model for Intracranial Aneurysm Detection in Computed Tomography Angiography

Authors: Wu D, Orru’ E, Ferraciolli S, et al.

Journal: Journal of NeuroInterventional Surgery, 2022; 14(Suppl 1):A134-A135

Highlight: Multi-institutional evaluation of AI for intracranial aneurysm detection in CT angiography.

Link: <https://jnis.bmj.com/content/14/Suppl_1/A134>

## CAFES: Chest X-ray Analysis Using Federated Self-Supervised Learning for Pediatric COVID-19 Detection

Authors: Parida A, et al.

Journal: Proceedings of SPIE Medical Imaging: Computer-Aided Diagnosis, 2024

Highlight: Federated self-supervised learning (FSSL) improved Vision Transformer-based pediatric COVID-19 diagnosis.

Link: <https://www.spiedigitallibrary.org/conference-proceedings-of-spie/12927/3008757/CAFES--chest-x-ray-analysis-using-federated-self-supervised/10.1117/12.3008757.short>

## Accelerating Artificial Intelligence: How Federated Learning Can Protect Privacy, Facilitate Collaboration, and Improve Outcomes

Authors: Patel M, Dayan I, Fishman EK, et al.

Journal: Health Informatics Journal, 2023; 29(4)

Highlight: Demonstrates how federated learning enhances AI while safeguarding privacy in collaborative settings.

Link: <https://journals.sagepub.com/doi/10.1177/14604582231207744>

## Multi-institutional Federated Image Quality Assessment of Prostate MRI Scans

Authors: Hariri A, et al.

Journal: AACR Annual Meeting, 2023

Highlight: Federated learning framework for assessing prostate MRI quality across multiple centers.

Link: <https://aacrjournals.org/cancerres/article/84/6_Supplement/2344/738823>

## Federated Training of Deep Learning Models for Prostate Cancer Segmentation on MRI: A Simulation Study

Authors: Wang S, et al.

Journal: Accepted for ISMRM 2024

Highlight: Demonstrated federated learning for robust segmentation models in prostate cancer MRI imaging.

## Collaborative Multisite Pediatric COVID-19 Detection Using Federated Learning on Chest X-rays

Authors: Parida A, et al.

Journal: Submitted for CNH REI Week 2024

Highlight: Explores federated learning for collaborative pediatric COVID-19 diagnostic studies.

## Federated Learning Framework for NLP in Healthcare: Assessing Hospital Readmission Using Electronic Health Records

Authors: Nalawade P, et al.

Journal: Submitted for Bio-IT World 2023

Highlight: Federated learning for natural language processing applications in hospital readmission prediction.

Link: <https://dspace.mit.edu/handle/1721.1/151725>

## Federated Training of Deep Learning Models Improves Prostate Gland Segmentation on MRI: A Simulation Study

Authors: Sompalle P, et al.

Journal: Accepted for SIIM 2024

Highlight: Validated federated learning for segmentation of prostate glands in MRI data.

Link: <https://annualmeeting.siim.org/wp-content/uploads/2024/05/2005_Shiradkar_Federated-Training-of-Deep-Learning-Models-Improves-Prostate.pdf>

## Platform components

Rhino FCP consists of two main components:

1. **Rhino Client:** Software installed on a virtual machine or physical server behind the data custodian’s firewall, whether on-prem in a data center or on a Virtual Private Cloud (VPC). The Rhino Client can securely access local datasets and has access to GPUs/CPUs in order to securely run computational workloads (e.g., AI-model training) without moving sensitive data outside the firewall.
2. **Rhino Cloud:** Software that orchestrates tasks across Rhino Clients (e.g., FL), and is the access point for all user interactions with Rhino FCP. It is hosted on AWS. Patient data is not transferred to the Rhino Cloud, thus enabling collaborations with data custodians who are restrictive in their data-sharing requirements.

The Rhino Cloud exposes three types of interfaces to users:

1. **Web UI:** A graphical web-based user interface (GUI) accessible via browsers at <https://dashboard.rhinohealth.com>.
2. **Python SDK:** A Python library accessible via browsers at <https://pypi.org/project/rhino-health/> for interacting with Rhino FCP from Jupyter Notebooks or any other Python-based system.
3. **REST API:** An HTTPS-based REST API that interacts with Rhino FCP. Both the Web UI and the Python SDK use this REST API under the hood.

The approach is “centralized control with decentralized execution,” allowing users to manage federated AI projects using cloud interfaces to oversee compute processes run at the edge. Rhino FCP manages the interactions between the Rhino Clients and Rhino Cloud so that the data on the Rhino Clients never leaves the data custodian’s network. This is accomplished via a secure interface between the Rhino Clients and the Rhino Cloud that only supports specific actions, not allowing patient data to be transferred from the Rhino Client.

Rhino FCP is HIPAA and GDPR-compliant and is ISO 27001 and SOC 2 Type II certified. Additional information regarding Rhino FCP’s different security features is outside the scope of this white paper, and can be provided on a case-by-case basis.

### Rhino FCP Architecture


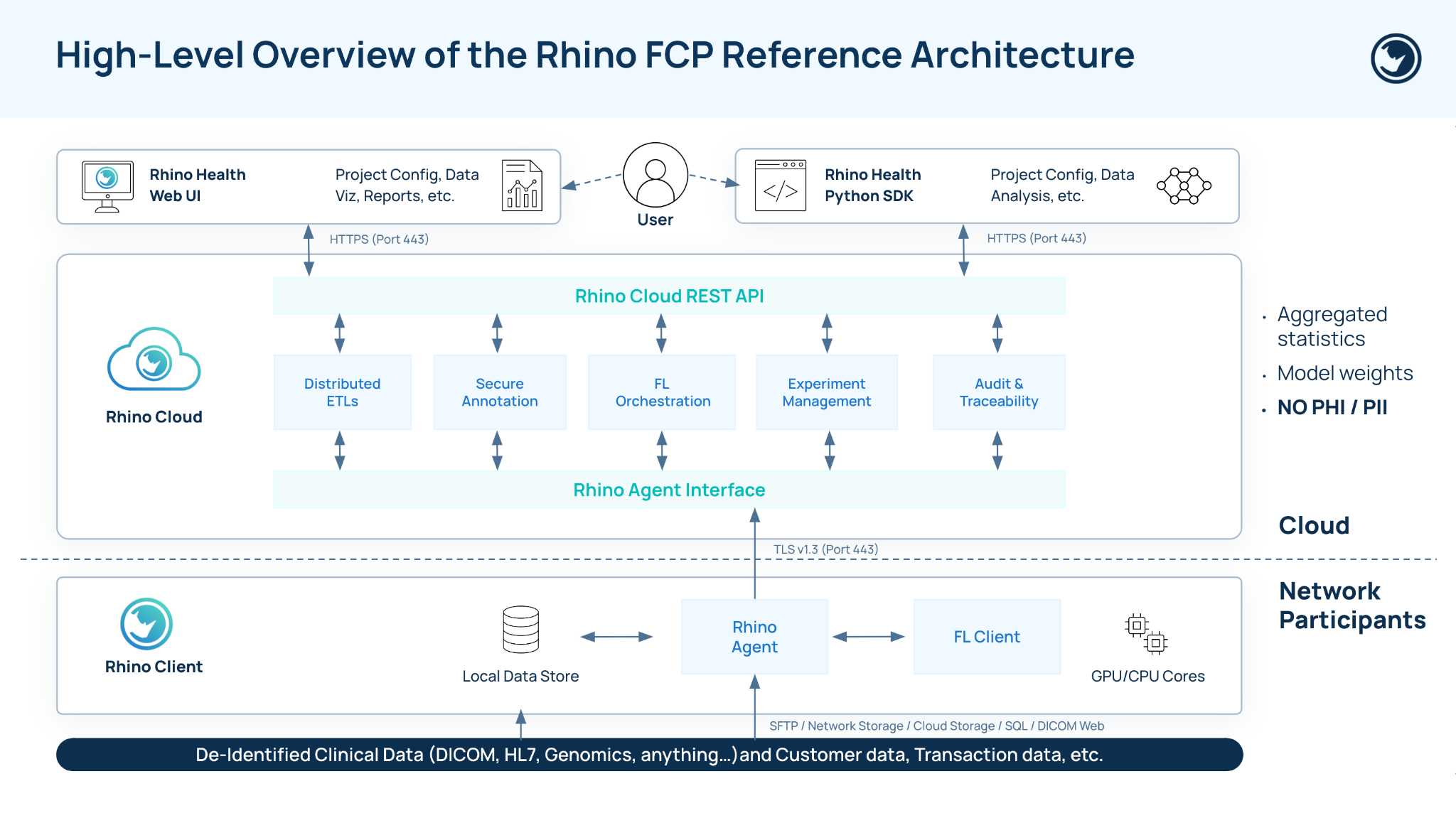
Rhino FCP consists of four secure layers. Users interact via the Rhino Web UI, Python SDK, or API. The Rhino Cloud environment, accessed through HTTPS, manages components like distributed ETLs, secure annotation, and FL orchestration without transferring row-level data. The Rhino Client, operating within the data custodian’s network, handles storage and computing, communicating securely with the Cloud. This architecture ensures row-level data remains within the data custodian’s network, enabling secure collaboration for model training and privacy-preserving analysis, with encryption at rest, in transit, and during processing.


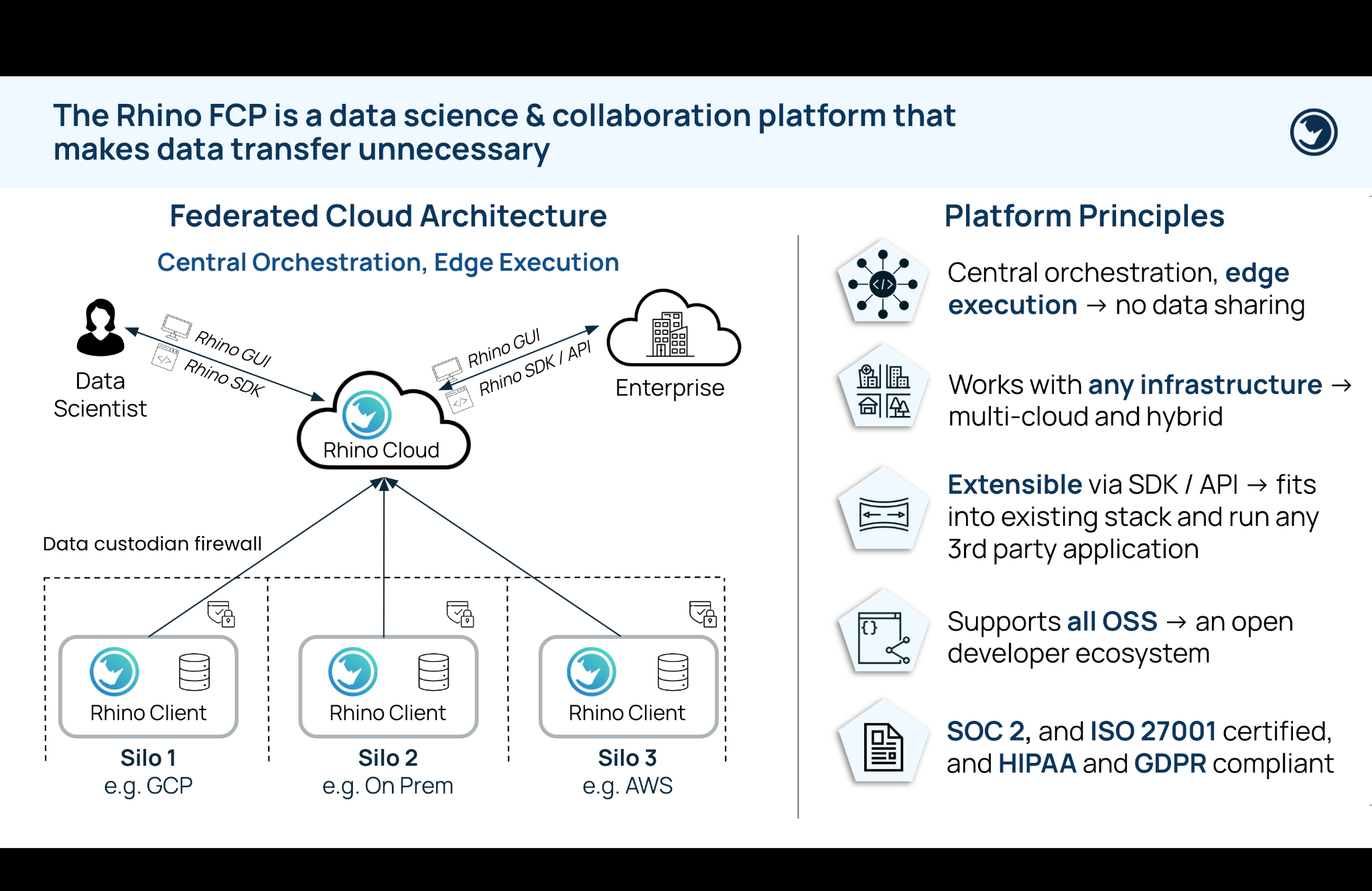


Rhino FCP leverages a “Central Orchestration, Edge Execution” model to create a Federated Cloud Network. This network consists of interconnected yet autonomous Rhino Clients operating across various environments, including on-premises data centers, virtual private clouds (VPCs), and public cloud services. Rhino FCP enables secure, efficient, and scalable data collaboration across multiple sites without transferring sensitive data outside local environments by federating these clients.

Rhino FCP is a comprehensive data science and collaboration platform that eliminates the need for data transfer. It employs a “Central Orchestration, Edge Execution” model, where users interact with the Rhino Cloud through the Rhino GUI and Python SDK. At the same time, enterprises connect via the Rhino Cloud GUI and SDK/API. This setup supports multi-cloud and hybrid environments, is extensible via SDK/API, and is compatible with all open-source software, fitting seamlessly into existing software stacks.

Rhino FCP’s Federated Computing approach offers several advantages:

- **Enhanced Data Privacy and Security:** Rhino FCP minimizes the risk of data breaches and unauthorized access by keeping data on Rhino Clients for processing purposes only. This local data storage supports privacy and security, reducing the potential for data compromise.
- **Cost Efficiency and Resource Optimization:** By processing data locally, edge computing significantly reduces the need to transmit large amounts of data, lowering data transfer expenses. Additionally, this approach avoids duplicate storage, further optimizing resource utilization and cost.
- **Improved Scalability:** Rhino FCP’s architecture allows for easy integration of new Rhino Clients and handles increasing data volumes efficiently without requiring significant infrastructure changes.
- **Reduced Latency:** Processing data locally on Rhino Clients minimizes latency compared to scenarios where data needs to be sent to a central location for processing.

***Reference:***

[*Rhino Trust Center Papers*](https://app.vanta.com/rhinohealth/trust/b6eq2ndtqlgh7mzp55a3e)

### Rhino FCP platform features

| *Criteria* | *Details* |
| --- | --- |
| ***Security and Privacy*** | **Trusted Execution Environment:**  The code is executed in a tightly controlled trusted execution environment to ensure security and prevent data exfiltration. In addition, the execution environment controls (e.g. absence of network access, no human user access) and granular permissions management have all been put in place to ensure that containerized code can be run in a secure enclave after the code execution approval process.  **Least privilege with user identity and authorization controls**:  Users are assigned specific roles in specific workgroups to configure project settings, build, test, and run code such as model training and evaluations and can only see model metrics and aggregated model parameters.  **Data Encryption at all three modes**  The server on which the Rhino Client is installed has hard-drive encryption such that all data is encrypted at rest. All communication between the Rhino Client and the Rhino Cloud is encrypted using strong industry standard TLS based encryption methods. Rhino Clients can be configured to run in Confidential VMs or other Trusted Execution Environments (TEEs), in which case all code will be executed in the TEE ensuring encryption during processing and code execution.  **Audit Trails and Continuous Security Monitoring** - User activity is logged within the system and major user actions like creating a new project, adding a collaborator to a project, importing datasets, etc. are displayed in a visual log.  **Data Tokenization:**  Rhino FCP can selectively tokenize the input PII fields such as HIPAA safe harbor fields and make the tokenized version of the data available for federated training. |
| ***Compliance and Regulatory Adherence*** | The FCP is HIPAA and GDPR compliant and is SOC-2 type 2 and ISO-27001 certified.  For more information, please visit our trust center. |
| **Interoperability and Extensibility** | We support integration with both structured EHR data models, standard data models such as HL7 FHIR and DICOM. In addition, unstructured clinical notes, non-DICOM images (pathology), protein, and genomics data formats.  **Data Harmonization:**  Rhino FCP offers the Harmonization Copilot application, which leverages cutting-edge Generative AI capabilities and Federated Learning (FL) to streamline this transformation. The Harmonization Copilot application simplifies and accelerates data harmonization by standardizing clinical data to fit Common Data Models and standard vocabularies. It makes the adoption of industry data models and vocabularies easier. |
| ***Data Quality and Integrity*** | **Data Validation**: We provide out of the box Data Analytics on data completeness and data distributions. Using Rhino SDK, one can customize additional data validation metrics and visualize using open source libraries and visualization tools.  **Data Version control and Provenance Tracking:** Datasets are version controlled and all the activities on the datasets are logged. In addition, we maintain all versions of transformed data and track the intermediate datasets. |
| ***Usability and Accessibility*** | We provide extensive [documentation](https://docs.rhinohealth.com/hc/en-us) and [open source github](https://github.com/RhinoHealth/user-resources) examples. In addition, each customer goes through 1:1 onboarding by our Forward Deployed Engineers and we assist most customers execute the first project. In addition, customers have access to Rhino support for any product related assistance.  In November 2024, we conducted a Web Accessibility Conformance Report (VPAT) against the WCAG 2.2 standard and have a roadmap to address the gaps to compliance up to AAA criteria. |
| ***Scalability and Performance*** | Rhino FCP Orchestrator monitors the performance of jobs in all Rhino Clients and provides access to client site logs through a secure interfece. Depending on the needs of the jobs, it can execute on large clusters and provide dynamic scalability configurations.  Our customers executed custom medical imaging deep learning segmentation and classification model training, large language model fine tuning, clinical natural language processing.  Given that the compute on the Rhino FCP is distributed among different institutions, the scalability to 10s of thousands (or more) of parallel users is simple both for different users working in parallel on different projects, as well as for different users working together on the same project to perform federated training across many institutions. |
| ***Collaboration and Sharing Capabilities*** | **Collaboration Setup:** Rhino has an application level permission and policy enforcement module, which helps in granting least privilege necessary to human users or machine accounts for a given task and provides configuration options for data custodians and consortium operators. See [Rhino documentation](https://docs.rhinohealth.com/hc/en-us/categories/21376674931101-Setting-Up-Projects-and-Collaboration) for detailed permission management settings.  In Rhino FCP, the [collaboration](https://docs.rhinohealth.com/hc/en-us/articles/12384883575837-What-is-a-Collaborator) is organized around “Organization” and “Workgroup”. Each Organization can have multiple workgroups. A [**Project**](https://docs.rhinohealth.com/hc/en-us/articles/12384887634333-What-is-a-Project) forms the core organizational unit that encapsulates a distinct data analysis or research initiative. The assets such as Datasets and Code Object are key components of a project.  **Collaboration Tools:** Rhino provides GUI and Notebook based approaches to interact with the platform and develop and test models in collaborative settings. In addition, certain groups of users may use annotation and interactive data analysis tools without any data exfiltration risks.  See Rhino Documentation on various [code collaboration](https://docs.rhinohealth.com/hc/en-us/categories/21376714625053-Creating-and-Running-Code) options. |
| ***Cost and Sustainability*** | Cost Effectiveness: Since Rhino FCP does not require the data to move to another cloud /network, customers save significant network and personnel cost of data centralization effort. Second, we productized many AI pipeline components which were previously required for large scale consulting projects - such as security and collaboration setup, data harmonization, MLOps setup, and container orchestration across a large number of federated sites.  Sustanability: The Rhino FCP is supporting a [large number of institutions](https://www.rhinohealth.com/federated-network#federated-network-map) from end-to-end: from consortium agreements to writing IRB proposals to completing infosec / legal / compliance / procurement reviews to then guiding IT teams on hardware provisioning & custom configurations to finally supporting data engineering & data science. In addition, Rhino FCP is getting used beyond academic medical centers - by Life science companies building consortiums among themselves and with the data collaborators.  Furthermore, we built relationships with the AI and Data technology ecosystem - Rhino is the strategic federated computing partner of NVIDIA, Google Cloud, and AWS. |
| ***Ethical Considerations*** | We're in the Ethical AI Database for our ability to promote data diversity, respect patient privacy, and preserve data sovereignty: [link](https://www.linkedin.com/posts/rhinohealth_ai-healthcare-lifesciences-activity-7150157740202496002-BS1e/)  Recently, Rhino received a Health Equity [award](https://www.digitalhealthhub.org/awards/2024/2024-winners#health-equity)  because of data diversity, sovereignty, and privacy. We are completing a certification for the NIST AI Risk Management Framework.  Furthermore, we're also members of [CHAI](https://chai.org/), which aims to promote responsible AI development.  Lastly, we have users who attest that they have appropriate consent / right to process any data they use on Rhino FCP and have a DSAR process in place per GDPR requirement. |
| ***Innovation and Adaptability*** | Rhino FCP follows microservices REST API driven architecture approach. This means it is very easy for us to adopt new innovations, integrate with the ecosystem without disrupting the core features. For example, [NVIDIA FLARE](https://developer.nvidia.com/flare) (NVFlare) is an open source SDK for federated learning that has been integrated into the Rhino Health Platform (RHP). In addition, we integrate with enterprise identity management tools, model registry such as Hugging Face, FHIR/DICOM APIs, and cloud based data sources.  Recently, Rhino FCP launched the Harmonization Copilot application, which leverages cutting-edge Generative AI capabilities and Federated Learning (FL) to streamline this transformation. The Harmonization Copilot application simplifies and accelerates data harmonization by standardizing clinical data to fit Common Data Models. |

***References***

[*Rhino Trust Center Papers*](https://app.vanta.com/rhinohealth/trust/b6eq2ndtqlgh7mzp55a3e)

## Matrix Rhino FCP common challenges

| **Category** | **Description** |
| --- | --- |
| **Consortium Setup Agreement and Operational Coordination** | To efficiently implement FL, we need business, resesarch, and commercial agreements among the collaborators. The process may take several months.  While Rhino is not engaged in this process directly, but we help in the following ways:   1. We connect the new participants to our federated data network and provide a data catalog for them to do feasibility analysis and make the decisions on collaborators. 2. We provide product demonstrations and walk through technical papers to educate the collaborators that their data is safe and their intellectual property is protected. And they can get started without building the federated infrastructure themselves. |
| **Federated Computing Technical Skills** | Many researchers and engineers are new to deep learning and federated learning. Oftentimes, they experience a steep learning curve. Rhino provides tutorials and onboarding to alleviate this challenge and we are working on FCP academy and structured certification programs. |
| **Cloud/On-prem Foundation Network and Compute Hardware and availability of GPUs** | Federated learning participants are responsible for training the global model on their local data in their compute infrastructure. Many customers in healthcare have not fully embraced public or private cloud infrastructure. Oftentimes, it takes time to install required hardware and they may not have the right personnel to administer the system. In addition, allocating and paying for GPUs is a challenge for many organizations.  Rhino is working on a “Managed Compute” solution where we will provide options to run compute as a managed service - which will provide cost effective dynamically scaled infrastructure. |

## Data Modalities Supported by Rhino FCP

Usually, clinical research data platforms are designed to integrate and manage a wide range of data modalities to support biomedical research. The primary data modalities used so far in

Rhino FCP includes:

| **Category** | **Data Modality** | **Description** |
| --- | --- | --- |
| **Clinical Data** | Electronic Health Records (EHRs) | Structured data from US and International EHR systems and unstructured data - clinical notes. |
| Claims | Hospital Administrative Data | Admissions, discharges, transfers, billing codes, and insurance information. |
| **Genomic Data** | Genomic Sequences | Whole genome, exome sequencing, targeted sequencing from common sequencing devices - FASTA, FASTQ , SAM/BAM, VCF formats. |
| **Imaging Data** | Radiology Images | MRI, CT, X-ray, OCT. |
|  | Pathology Images | Digital pathology slides, histology images and their metadata. |
| **Phenotypic Data** | Disease Phenotypes | Disease characteristics, symptom severity, progression. Use of HPO or other controlled vocabularies for annotation. |
|  | Clinical Outcomes | Treatment responses, survival rates, recurrence. |
| **Medication Data** | Prescription Records | (Co-)Medication names, dosages, administration routes, duration. |
|  | Medication Adherence / Compliance | Refill records, patient self-reports. |
| **Laboratory Data** | Lab Test Results | Blood tests, urine tests, microbiological cultures, Biomarker measurements. |
| **Survey Data** | Questionnaires and Surveys | Patient health questionnaires, lifestyle surveys, mental health assessments. |
|  | Patient-Reported Outcomes | Pain scales, quality of life measures, functional status. |
| **Biomarker Data** | Proteomics | Rhino FCP can support this data type. We do not have a current project. |
|  | Metabolomics | Rhino FCP can support this data type. We do not have a current project. |
| **Environmental Data** | Lifestyle Factors | Diet, physical activity, workout schemata, smoking, alcohol consumption, substance (ab)use. |
|  | Environmental Exposures | Air quality, water quality, exposure to toxins, occupational hazards. |
| **Socioeconomic Data** | Social Determinants of Health | Education, income, employment status, housing, neighborhood characteristics. |
| **Family History Data** | Genetic Risk Factors | Family history of diseases, pedigree analysis. Risk alleles, tumor gene panels etc. |
| **Longitudinal Data** | Time-Series Data | Repeated measures over time, disease progression, treatment responses over time.  Rhino FCP uses standard data models like OMOP and FHIR as target data models. These data models provide many timestamp columns where we may map timestamp fields from source systems. In addition, Rhino FCP provides support for large historical data loading and incremental data loading so that we can maintain the patient events over time. |
| **Behavioral Data** | Behavioral Assessments | Cognitive tests, psychological assessments, behavioral interventions. |
|  | Transcriptomics | mRNA levels, non-coding RNAs, alternative splicing events.  Rhino FCP can support this data type. We do not have a current project. |
| **Pathway Data** | Biological Pathways | Signaling pathways, metabolic pathways. Mechanism graphs. Pathophysiology graphs (disease maps)  Rhino FCP can support this data type. We do not have a current project. |
|  | Interaction Networks | Protein-protein interaction networks, gene regulatory networks. Co-expression networks  Rhino FCP can support this data type. We do not have a current project. |

***References :***

<https://www.rhinohealth.com/post/automating-clinical-data-standardization-for-arc-at-sheba-with-rhino-health-harmonization-copilot>

<https://www.rhinohealth.com/federated-network#federated-network-map>

<https://www.rhinohealth.com/federated-network#discover-federated-datasets>

<https://www.ichom.org/>

## Built-in Workflows and Analysis Tools

Does Rhino FCP contain built-in workflows and analysis tools that facilitate clinical and translational research?

### Workflow

| **Feature** | **Description** |
| --- | --- |
| Patient Cohort Discovery | Rhino FCP provides comprehensive [federated statistics](https://www.rhinohealth.com/platform#use-cases-federated-statistical-methods-and-analytics) and analysis methods and UI/SDK options to discover patient cohorts. In addition, we have a pre-built container available for the OHDSI Healthcare Analytics suite, which includes a cohort tool. These analytics could be executed either in a batch processing mode or interactive processing mode. |
| Data Integration and Management | Rhino FCP provides private connectors to object storage or network file systems or SQL database systems. Integrate heterogeneous data sources into a common data model (CDM), including clinical, genomic, and other research data. Rhino FCP supports mapping to common CDMs such as OMOP and FHIR. |
| Ontology Management | Create and manage ontologies for organizing and categorizing and making it easier to search and use.  Rhino FCP provides vocabulary service for common healthcare ontologies such as LOINC, SNOMED, RXNORM. In addition, customers may bring in their own vocabulary and ingest in the platform- which can be used in data transformation work. |
| Data Extraction and Transformation | Using the Rhino UI or Python SDK, the users may execute pre-built container images within the Rhino Client edge on local datasets. One can build ETL pipelines step-by-step and easily connect those steps into a single repeatable pipeline from ingestion to transformation. In addition, for clinical data, use Harmonization Copilot to automatically map and execute ETLs. FCP’s Harmonization Copilot allows one to quickly produce a semantic mapping or custom vocabulary mapping between a subset of the data in your Electronic Health Records (EHR) or Data Warehouse (DW) to OMOP or a custom vocabulary. This allows you to transform your data’s locally-defined names for procedures, conditions, and other items into supported clinical data standard names.  Rhino [Data Harmonization documentation](https://docs.rhinohealth.com/hc/en-us/categories/21376685700125-Importing-and-Harmonizing-Data-at-the-Edge) |
| Security and Privacy Management | **User Identity and Access Management:**  Users are assigned specific roles in specific workgroups based on a list of users/roles provided by the institution. Role based permissions are applied in each project in which the user participates. User activity is logged within the system and major user actions like creating a new project, adding a collaborator to a project, importing datasets, etc. are displayed in a visual log. It is also possible to use SSO to authenticate users with 3rd party authentication providers such as Google and Azure AD.  **User Authentication Policies:** Rhino FCP implemented several best practices such as strict password policies, multi-factor authentication, and monitoring unsuccessful attempts.  **Role Based Access Control (RBAC):**  Each project in the FCP defines a set of role based user permissions, usually defined by the project lead and agreed to by all participants. For example - which users are allowed to train a model, which users are allowed to view the results of model training, etc. These permissions are  enforced in the Rhino Cloud such that each user only has access to data and actions that are allowed by the permissions. Users do not have any access to projects in which they are not a participant.  **Data, Model, and Code Encryption Key Management support:**  Data Encrypted at Rest: The server on which the Rhino Client is installed has hard-drive encryption such that all data is  encrypted at rest.  **Data Encrypted in Transit**: All communication between the Rhino Client and the Rhino Cloud is encrypted using strong industry standard TLS based encryption methods.  **Code and Model Protection:** The code (e.g. models or Generalized Compute code) run in the Rhino Client are run as docker  containers that are tightly locked down. No communication is allowed, neither inbound or outbound, except for communication with the Federated Server for FL. This Federated Server is provisioned by Rhino Health for each FL training run and provided with SSL certificates to securely authorize the Federated Clients that are allowed to connect to it.  In addition, model parameters and code can be encrypted and only allow the code run time to access the decryption keys.  **Data de-identification tools:**  Data ingested by the Rhino Client is intended to be de-identified prior to ingestion using one of the HIPAA-compliant methods provided by the U.S. Department of Health and Human Services. The data used in a FL Project could be de-identified prior to making it available for Rhino Client.  Aggregation of de-identified patient data uses mechanisms to ensure that no information about an individual patient can be sent and persisted in the Rhino Cloud. These techniques include:  ● K-anonymization - ensuring that there are at least K data points in each piece of aggregated data. K is configurable per project.  ● Differential Privacy - adding a small amount of random noise to the data to ensure that the original data cannot be directly accessed. The level of enforcement of differential privacy is configurable per project. |

**References:**

<https://docs.rhinohealth.com/hc/en-us>

[Whitepapers](https://app.vanta.com/rhinohealth/trust/b6eq2ndtqlgh7mzp55a3e)

[Data deidentification](https://docs.rhinohealth.com/hc/en-us/articles/13136734951837-Dataset-De-identification)

Collaboration security setup [documentation](https://docs.rhinohealth.com/hc/en-us/categories/21376674931101-Setting-Up-Projects-and-Collaboration)

### Analysis Tools

| Query Interface | Rhino FCP has three query interfaces:   1. **Web UI** - a graphical web-based user interface (“GUI”) accessible via browsers. 2. **Python SDK** - a Python library accessible via browsers for interacting with the Rhino FCP from Jupyter Notebooks or any other Python-based system. 3. **REST API** - an HTTPS-based REST API that can be used to interact with the Federated Computing Platform. This REST API is used under the hood by both the Web UI and the Python SDK.   In all these interfaces, users can specify filter criteria and metrics they want to see in the query across federated data networks. |
| --- | --- |
| Timeline Viewer | Visualize individual patient timelines, displaying events such as diagnoses, treatments, and lab results over time.  The users of federated computing platforms, generally, do not have access to row level patient data. However, data owners can selectively provide access to collaborating members for patient level data visualization. Rhino FCP [Interactive Containers](https://docs.rhinohealth.com/hc/en-us/articles/12385209693981-What-is-an-Interactive-Container-Code-Object) allow users to connect to applications running at the edge, akin to having a remote desktop session. This enables direct interaction with the data, making it easier to visualize patient timeline directly within the secure confines of the local environment. |
| Statistics and Analytics | Basic statistical tools to analyze query results, including counts, distributions, and summary statistics.  Rhino FCP provides an Analytics page and SDK functions to query metrics from the individual sites and then combine the results from viewing. The customer can use any 3P visualization tool that can take the SDK output and format in a way suitable for the use case.  Rhino FCP includes a large set of privacy-preserving federated metrics, including Basic Statistics (Mean, Standard deviation, Median, and Percentiles), Common Statistical Methods [t-test, ANOVA (Analysis of Variance), Chi-square test, Correlation analysis (e.g., Pearson, Spearman), Regression analysis (e.g., linear regression, logistic regression)], and Epidemiology Metrics [Risk (e.g., relative risk, odds ratio), Prevalence, Incidence, Kaplan-Meier survival analysis, Cox proportional hazards (PH) model]. Only aggregate data are shared, and a privacy filter is applied to ensure that data extracted via these metrics cannot be used for re-identification, including k-anonymization, differential privacy, and field-level permissions. |
| Plugin Framework | Integrate external analysis tools and custom plugins to extend the platform's capabilities. Please provide references, examples and documentation of plugin architecture.  **FL Framework integration:** The RHP expands on the capabilities of NVFLARE, and enables easy and reusable FL orchestration without the hassle of IT provisioning and setup, as well as end-to-end support for the entire AI project lifecycle. We also plan to support the Flower framework early 2025.  **Federated TensorBoard:** Use TensorBoard to see real-time results from model runs, halt model runs that aren’t converging, and compare model runs across different experiments. All this while preserving the TensorBoard logs at the edge.  **3P tools integration:** Rhino FCP’s zero-footprint visualization technology enables providing and receiving permissions to view specific data points (e.g., tabular data, imaging studies) in a dynamic and audited way while enforcing that data never persists outside its local environment (i.e., the local Rhino Client). Rhino FCP has a fully integrated tabular data viewer, textual log viewer, image viewer (e.g., .png, .jpg), and DICOM viewer, Genomics Viewer, Protein Viewer. Rhino FCP supports various applications, from custom data visualization and annotation tools (e.g., 3D Slicer) to interactive data analysis environments like Jupyter Notebooks, and BI tools such as Streamlit. |
| Natural Language Processing (NLP) | Does Rhino FCP comprise already integrated NLP services? Are they open source?  Rhino FCP provides containerized edge NLP models based on open weight large language models - small to large sizes, including [Hugging Face pubmed models](https://huggingface.co/models?other=pubmed), [NVIDIA nemo NLP mode](https://catalog.ngc.nvidia.com/orgs/nvidia/collections/nemo_nlp)l, and [BioMistral](https://huggingface.co/BioMistral/BioMistral-7B). It enables users to efficiently extract and classify entities without sending the private data to LLM public API endpoints. In addition, Rhino FCP provides pre-built evaluation and fine tuning containers and example cookbooks for model customizations. |
| Genomic Data Analysis | Integrate and analyze genomic data alongside clinical data, often requiring additional modules or plugins.  [Nextflow](https://www.nextflow.io/) enables scalable and reproducible genomics workflows using containers. In Rhino FCP, users containerize their Nextflow genomics workflows and run on top of federated data networks in GPU or CPU only systems. In addition, Rhino supports joint analysis of clinical data and genomics data through containers that support multiple datasets as data input.  Furthermore, Rhino could work with other existing linux based orchestration tools and make the run efficiently across federated networks. |
| Temporal Querying | Perform queries that consider the temporal sequence of events, such as identifying patients who had a particular treatment before a specific diagnosis.  Rhino FCP provides tools to transform data from multiple tables into a single patient journey table with time windowing. Then, users can encapsulate their temporal queries in a container and dispatch the same to multiple sites. The result of that query is a dataset that could be visualized using Rhino GUI Analytics screen and augmented by a 3P visualization tool. |
| Data Visualization | Basic tools for visualizing data distributions and query results, extendable with additional plugins for advanced visualization.  Rhino FCP provides a python SDK with many analytical functions , so output of the SDK could be visualized using any python package such as matplotlib, streamlit app, or a business intelligence tool. |
| Export and Reporting | Does Rhino FCP allow for export of query results for further analysis or reporting purposes in formats compatible with other statistical and data analysis software.  Rhino FCP supports export of data in CSV and JSON format and can write to an external database or network storage systems. |

| **Integration with Other Tools** | R / BioConductor and Python Integration | Use R and Python scripts for advanced statistical analysis and machine learning workflows.  Rhino is flexible to integrate with most 3P analytics tools - we have containerized R and have pre-built containers available to use. |
| --- | --- | --- |
|  | Integration with Clinical Trial Management Systems (CTMS) | Is Rhino FCP integrated with CTMS for managing clinical trial data and workflows?.  Using Rhino REST API or Python SDK, we integrate with many cloud based systems. In addition, we may use data integration methods to bi-directionally connect with CTMS |
|  | Integration with Electronic Health Records (EHR) | Does Rhino FCP allow for seamless integration with EHR systems to pull in clinical data for analysis?  Yes, Rhino FCP connects to EHRs or EHR based data warehouse systems through HL7 V2, HL7 FHIR, or custom data file based methods. |

## Support for Semantic Integration

Does Rhino FCP support semantic integration through the use of terminologies, ontologies, and common data models?

1. **Terminologies and Ontologies**: Can Rhino FCP integrate with standard medical terminologies and ontologies such as ICD, SNOMED CT, LOINC, and others. This ensures consistent data representation and facilitates interoperability.? **Yes, as part of the Harmonization Copilot application.**
2. **Common Data Models (CDMs)**: Can Rhino FCP work with various common data models like the Observational Medical Outcomes Partnership (OMOP) CDM, enabling data standardization and easier data sharing across institutions.? **Yes, as part of the Harmonization Copilot application.**
3. **Ontology Management**: Does the platform include tools for ontology management, allowing users to customize and extend the ontologies as needed to fit their specific research requirements​? **We support all OMOP domains out of the box. In addition, users may add custom terminologies as well. FHIR can be supported via services.**

**References** : <https://docs.rhinohealth.com/hc/en-us/categories/21376685700125-Importing-and-Harmonizing-Data-at-the-Edge>
