# Supplementary material for: Selecting medical research data platforms for translational biomedical research: a five-tier overview and requirement-weighted assessment framework
Source: Front Digit Health. 2026 Jun 17;8:1814015. doi: 10.3389/fdgth.2026.1814015 (PMC13319098; doi:10.3389/fdgth.2026.1814015)
Supplement: Supplementary file 3 [file Supplementaryfile3.docx]

**VRE Data Platform with a history of the BIH**

*A****Virtual Research Environment (VRE)****which allows multiple researchers in different locations to work together in real time without restrictions was firstly described by UK’s****Joint Information Systems Committee (JISC)****VRE Collaborative Landscape Study in 2010 as “a platform, to help researchers from all disciplines to work collaboratively by managing the increasingly complex range of tasks involved in carrying out research on both small and large scales” (1)*

*In 2009, Federal Research Minister Annette Schavan founded the German Centers for Health Research to integrate and advance research on major diseases. This initiative led to the creation of the Berlin Institute of Health (BIH) in 2013, supported by Johanna Quandt's donation, to enhance collaboration between university and non-university research, with the Charité and the MDC as key partners.(2)*

Reference

1. <https://link.springer.com/chapter/10.1007/978-3-319-50070-6_1>
2. https://www.bihealth.org/en/translation/innovation-enabler/quest-center/calls-and-awards/quest-calls-and-awards/quest-open-data-reuse-award

**Matrix table for the VRE platform’ features (the BIH)**

| **Aspect** | **Description** |
| --- | --- |
| **Security and Privacy(1,10)** | Security levels that enable work with open as well as with confidential data. All data derived from electronic health records (EHRs), picture archiving and communication systems (PACS), laboratories, and other sources, can be de-identified, transformed and prepared in the VRE Green Room, an isolated staging area, prior to being transferred to the Core Zone where is made available to the entire Project team for research use |
| **Compliance and Regulatory(2)** | complies with various regulations and standards such as GDPR |
| **Interoperability (2)** | interoperability with international data commons like EBRAINS and Virtual Brain Cloud, as well as support for common data standards such as the Brain Imaging Data Structure (BIDS) |
| **Data Quality and Integrity(10,11)** | The VRE offers platform integrations into a central portal to provide researchers with the resources they need to securely collect, analyse, catalogue, and share their research data. Provenance tracking creates a lineage of transformation for each file to support high data quality and integrity. |
| **Usability and Accessibility(11,10)** | Ease of use for researchers, easy access to discovery, access and re-use of data. including support for diverse user needs and compliance with accessibility standards. Workspaces are flexible and interactive environments in which users can access, visualise and analyse their data with a range of analysis and visualization tools. |
| **Scalability and Performance(3,13)** | Capacity to handle increasing amounts of data and users efficiently without performance degradation. the VRE has been designed and implemented using a microservices architecture, which enables the platform to be reliable, resilient, scalable, and highly adaptable to evolving research requirements and to changes to the underlying IT infrastructure. |
| **Collaboration and Sharing Capabilities(5 ,10)** | As a general purpose data platform, VRE offers secured storage spaces that can be operated via graphical and command line interfaces for easy exchange of data and metadata, and allows the VRE to be interoperable with other data platforms, data sources and systems. |
| **Cost and Sustainability(14)** | The BIH/Charité Virtual Research Environment is fully open-source (https://github.com/vre-charite), and the VRE is open also for external users who do not belong to Charite |
| **Ethical Considerations(4)** | VRE creates Projects for new studies only upon positive votes of the involved ethics committees. As an open source community, working with standard interoperable file systems and formats, VRE supports FAIR and open source data exchange and linkage. |
| **Innovation and Adaptability(6,7,13)** | VRE is based on state-of-the-art containerized microservice architecture that can be flexibly extended and adapted while providing built-in scalability and fault tolerance. |

**References :**

1. <https://unece.org/fileadmin/DAM/stats/documents/ece/ces/ge.46/20150/Paper_6_Session_4_-_Germany__Schiller_.pdf>
2. <https://vre.charite.de/vre/pages/about>
3. <https://www.utwente.nl/en/bms/datalab/collaborationinresearch/Virtual-Research-Environment(VRE)/>
4. <https://www.sciencedirect.com/science/article/pii/S1877050911001761>
5. <https://link.springer.com/chapter/10.1007/978-3-030-52829-4_15>
6. <https://journal.mostwiedzy.pl/TASKQuarterly/article/view/1788/1696>
7. <https://www.bihealth.org/en/translation/network/digital-medicine/bihcharite-virtual-research-environment/virtual-research-environment-architecture>
8. <https://www.bihealth.org/en/translation/network/digital-medicine/bihcharite-virtual-research-environment/virtual-research-environment-architecture>
9. <https://cordis.europa.eu/programme/id/H2020_EINFRA-9-2015>
10. <https://www.bihealth.org/en/translation/network/digital-medicine/bihcharite-virtual-research-environment/virtual-research-environment-architecture>
11. https://vre.charite.de/vre
12. <https://www.bihealth.org/en/about-us/history>
13. <https://www.bihealth.org/en/translation/network/digital-medicine/bihcharite-virtual-research-environment/virtual-research-environment-architecture>

**VRE Architecture( the BIH) (1)**


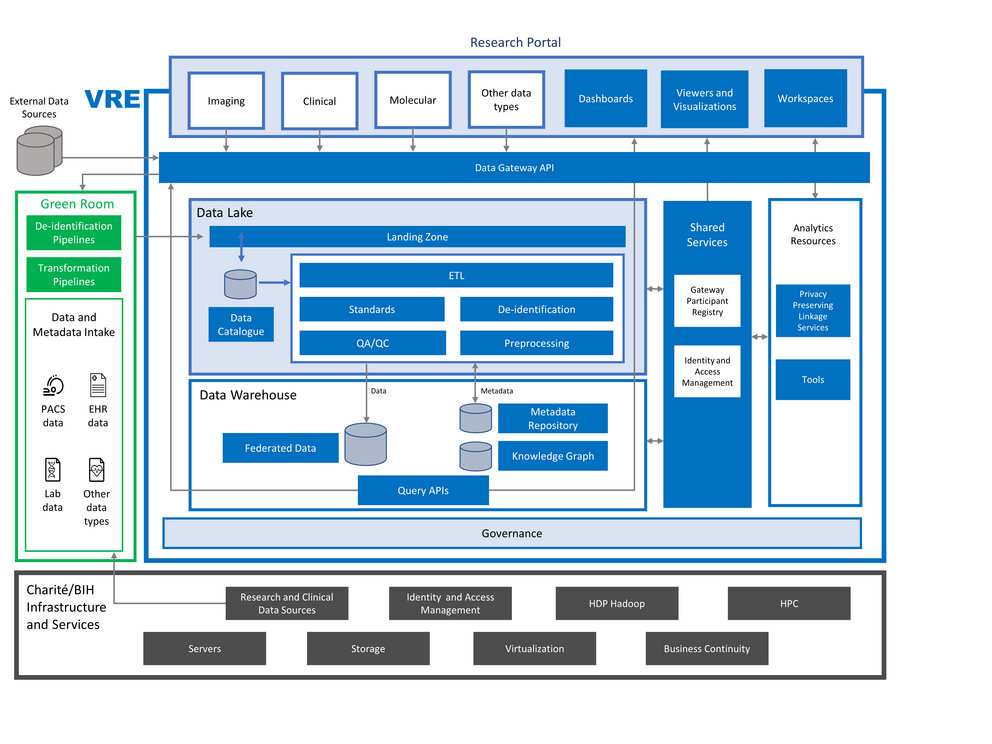


**Reference**

***1.*** ***https://www.bihealth.org/en/translation/network/digital-medicine/bihcharite-virtual-research-environment/virtual-research-environment-architecture***

**VRE Platforms Challenges**

| **Challenge** | **Description** |
| --- | --- |
| **Small Commercial Market** | The availability of trusted VREs is poor, with most developments originating from academic and research communities, posing sustainability challenges. |
| **Time-consuming Development** | Robust platform development is time-consuming and often customized to specific domains or computing infrastructures, creating gaps between the available software for researchers and the needs of daily scientific practices. |
| **Openness and Collaboration** | VREs need to be open, extendable and based on widely used standards to connect with new infrastructures and communities, expanding collaboration across scientific domains. |
| **Standardized Metadata and APIs** | Low interoperability among current research infrastructures complicates the creation of standardized interfaces for data access and computing tasks. |
| **Sustainable Cataloguing Solutions** | Challenges include integrating new RI resources, handling standards updates, scaling with data volumes, and ensuring proper data attribution. |
| **Steep Learning Curve** | For processing of data the VRE makes Virtual Machines available to its users that are set up like standard personal computers, including the typical file and operating systems (Linux, Windows), making it easy to use the system. |
| **Data Heterogeneity** | Heterogeneous data sets make linking similar data types difficult and lack of operational functions for using semantics to link data sets. |
| **Sustainability Commitment** | Without assurances of medium and long-term sustainability, e.g. via long-term governmental funding, it's challenging to get researchers to commit time and effort to VREs. |
| **Scoping International Landscape** | Difficulties in identifying and defining VREs across different countries and disciplines, as well as locating on-premise or early-stage VRE projects. |
| **Comprehensive Global Picture** | Impossible to obtain an exhaustive global picture of VREs due to their recent development and varying stages, leading to a focus on identifying trends and issues rather than comprehensive details. |

**References**

1. https://onlinelibrary.wiley.com/doi/full/10.1002/spe.3098

2.https://www.researchgate.net/profile/Stefan-Buddenbohm/publication/283182060_Success_Criteria_for_the_Development_and_Sustainable_Operation_of_Virtual_Research_Environments/links/56cc354608aee3cee54360e8/Success-Criteria-for-the-Development-and-Sustainable-Operation-of-Virtual-Research-Environments.pdf

3. <https://www.sciencedirect.com/science/article/abs/pii/S0167739X19302699>

4.https://www.sciencedirect.com/science/article/pii/S0308521X23001117?ssrnid=4357575&dgcid=SSRN_redirect_SD

5.https://citeseerx.ist.psu.edu/document?repid=rep1&type=pdf&doi=213482d2e583be8c348c9bd583eb1a8c9c2d93a8

**VRE platforms Data Modality**

| **Data Modality** | **Description** | **Examples** |
| --- | --- | --- |
| **Clinical Data(1)** | Written or printed data in text form | Lab measurements, clinical trial data |
| **Numerical Data** | Quantitative data that can be expressed in numbers | Survey results, statistical data, financial data |
| **Geospatial Data(2)** | Data related to geographic locations | Maps, GIS data, spatial coordinates |
| **Sensor Data(3)** | Data collected from various sensors | Temperature readings, accelerometer data, IoT data |
| **Genomic Data(1)** | Data related to genetic sequences | DNA sequences, RNA sequences, gene expression profiles |
| **Metadata(1,2)** | Data that describes other data | Metadata on these datasets are also captured centrally within the Knowledge Graph and the Metadata Repository. |

**References :**

1. [***https://www.bihealth.org/en/translation/network/digital-medicine/bihcharite-virtual-research-environment/virtual-research-environment-architecture***](https://www.bihealth.org/en/translation/network/digital-medicine/bihcharite-virtual-research-environment/virtual-research-environment-architecture)
2. [***https://link.springer.com/chapter/10.1007/978-3-030-94219-9_22***](https://link.springer.com/chapter/10.1007/978-3-030-94219-9_22)
3. ***https://link.springer.com/chapter/10.1007/978-3-030-39815-6_11***

**VRE platform (the BIH) Data Modality**

| **Data Modality** | **Description** |
| --- | --- |
| **Clinical Data** | Electronic Health Records (EHR), Patient demographics, Medical history, Diagnosis codes (e.g., ICD-10), Procedure codes (e.g., CPT), Lab test results, Medication records |
| **Imaging Data** | Radiology images (e.g., X-rays, MRI, CT scans), Pathology images, Other diagnostic imaging data |
| **Genomic Data** | DNA sequences, RNA sequences (transcriptomics), Genotype data, Variant data (e.g., SNPs, CNVs), Epigenetic data |
| **Biospecimen Data** | Sample information (e.g., blood, tissue), Biobanking data, Biomarker data |
| **Phenotypic Data** | Clinical phenotypes, Disease characteristics, Patient-reported outcomes |
| **Omics Data** | Proteomics, Metabolomics, Lipidomics |
| **Wearable and Sensor Data** | Data from wearable devices (e.g., fitness trackers), Continuous monitoring data (e.g., heart rate, glucose levels) |
| **Environmental and Lifestyle Data** | Environmental exposures (e.g., pollution levels), Lifestyle factors (e.g., diet, physical activity) |
| **Administrative and Claims Data** | Health insurance claims, Billing records, Resource utilization |
| **Research Study Data** | Clinical trial data, Study protocols, Participant information |
| **Text Data** | Clinical notes, Pathology reports, Radiology reports |
| **Public Health Data** | Epidemiological data, Population health statistics |

**Reference :**

***https://www.bihealth.org/de/translation/netzwerk/digitale-medizin/bihcharite-virtual-research-environment***

**VRE Semantic features**

| **Component** | **Definition** | **Role in VRE Data Platform** |
| --- | --- | --- |
| **Terminologies** | Standardized vocabulary and terms used within a specific domain. | As a general-purpose data platform, the VRE is open to all vocabularies. Metadata can be annotated via a graphical interface, or by providing JSON files. |
| **Ontologies(1)** | Structured frameworks that define the relationships between concepts in a specific domain. | As a general-purpose data platform, the VRE is open to all ontologies. Metadata can be annotated via a graphical interface, or by providing JSON files. |
| **Common Data Models** | Standardized schemas that define how data is structured and formatted across different systems. | As a general-purpose data platform, the VRE is open to all common data models. Metadata can be annotated via a graphical interface, or by providing JSON files. |
| **Metadata Standards(2)** | Guidelines for describing data, making it easier to discover, access, and use. | As a general-purpose data platform, the VRE is open to all metadata standards. Metadata can be annotated via a graphical interface, or by providing JSON files. Metadata from datasets are also captured centrally within the Knowledge Graph and the Metadata Repository |
| **Data Integration Tools(3)** | Tools and platforms that support the integration of data from diverse sources using the above components. | As a general-purpose data platform, VRE is open to the installation of any software that support the integration of data from diverse sources. |
| **Interoperability Frameworks(1)** | Standards and protocols that facilitate seamless data exchange and integration across systems. | Data can be exchanged via the upload and download functions which exist in the form of a web interface as well as client for download to local machines. Interoperability with international data commons like those developed under the [European Open Science cloud](https://ec.europa.eu/research/openscience/index.cfm?pg=open-science-cloud), for example the [Virtual Brain Cloud](https://virtualbraincloud-2020.eu/tvb-cloud-main.html) or the [Human Brain Project](https://www.humanbrainproject.eu/en/). |

**References :**

1. <https://cordis.europa.eu/programme/id/H2020_EINFRA-9-2015>
2. <https://www.bihealth.org/en/translation/network/digital-medicine/bihcharite-virtual-research-environment/virtual-research-environment-architecture>
3. <https://www.bihealth.org/en/research/scientific-infrastructure/core-units/interoperability/home/projects/wearables-telemedicine/integration-hub>

**Other features**

1. workflows for radiologic imaging data
2. a model for cataloguing data and making it easier to find
3. workbenches for modelling, simulating and analyzing data

**Workbench tools(1)**

| **Tool Name** | **Explanation** |
| --- | --- |
| Apache Guacamole | A built-in remote desktop gateway, for accessing Virtual Machines and facilitating workbench analyses. |
| Jupyterhub | A multi-user version of Jupyter Notebook that provides access to custom and pre-configured data science computational environments. |
| Superset | A data exploration and visualization platform, designed for integrating modern databases and building visualization dashboards. |
| Command line Tool | A binary executable program that provides a command-line client for managing data within the VRE platform. |
| HPC gateway | The High Performance Computing (HPC) cluster hosted by the Charité that is integrated directly with the VRE workbench. |
| WIKI | A collaborative content management tool for project documentation and learning resources. |
| Datalad | A data management multitool that can assist you in handling the entire life cycle of digital objects. (coming soon) |
| Marmotgraph | A Knowledge Graph. (coming soon) |

**References:**

1. <https://vre.charite.de/vre/pages/resources>
