# Supplementary material for: Selecting medical research data platforms for translational biomedical research: a five-tier overview and requirement-weighted assessment framework
Source: Front Digit Health. 2026 Jun 17;8:1814015. doi: 10.3389/fdgth.2026.1814015 (PMC13319098; doi:10.3389/fdgth.2026.1814015)
Supplement: Supplementary file 4 [file Supplementaryfile4.docx]

*Here comes the “empty” form to add your information. All my comments are labeled in blue. They can be removed. Would be good if you chose another color for your input.*

***42HSL platform***

***Deployment and Usage****:*

***42HSL*** is a decentralized machine learning approach that uses **blockchain** technology and **peer-to-peer** networking to enable **collaborative** **model training** across distributed datasets while preserving **data privacy** and **security** thereby going **beyond classical federated learning** approaches, the **42Hills Swarm Learning (42HSL)** allowing Swarm Learning applications across **heterogenous hardware** and software architectures in healthcare organizations globally.

***References for the platform:***

1. N/A – under active development

***42HSL* components**

42HSL is based on federated secure meshes using open-source standards, for securely identifying systems in dynamic and heterogeneous environments for mutual authentication. It leverages zero trust attestation in micro-segmented mesh networks to minimize attack vectors for all SL network participants. Participants are authenticated and authorized leveraging existing Identity Providers (IdP), and services authentication is built into service meshes. 42HSL uses CNCF cloud-native technologies that leverage well-established Git repositories alongside foundational orchestration platforms like Kubernetes with applying GitOps principles in the decentral setup.

*
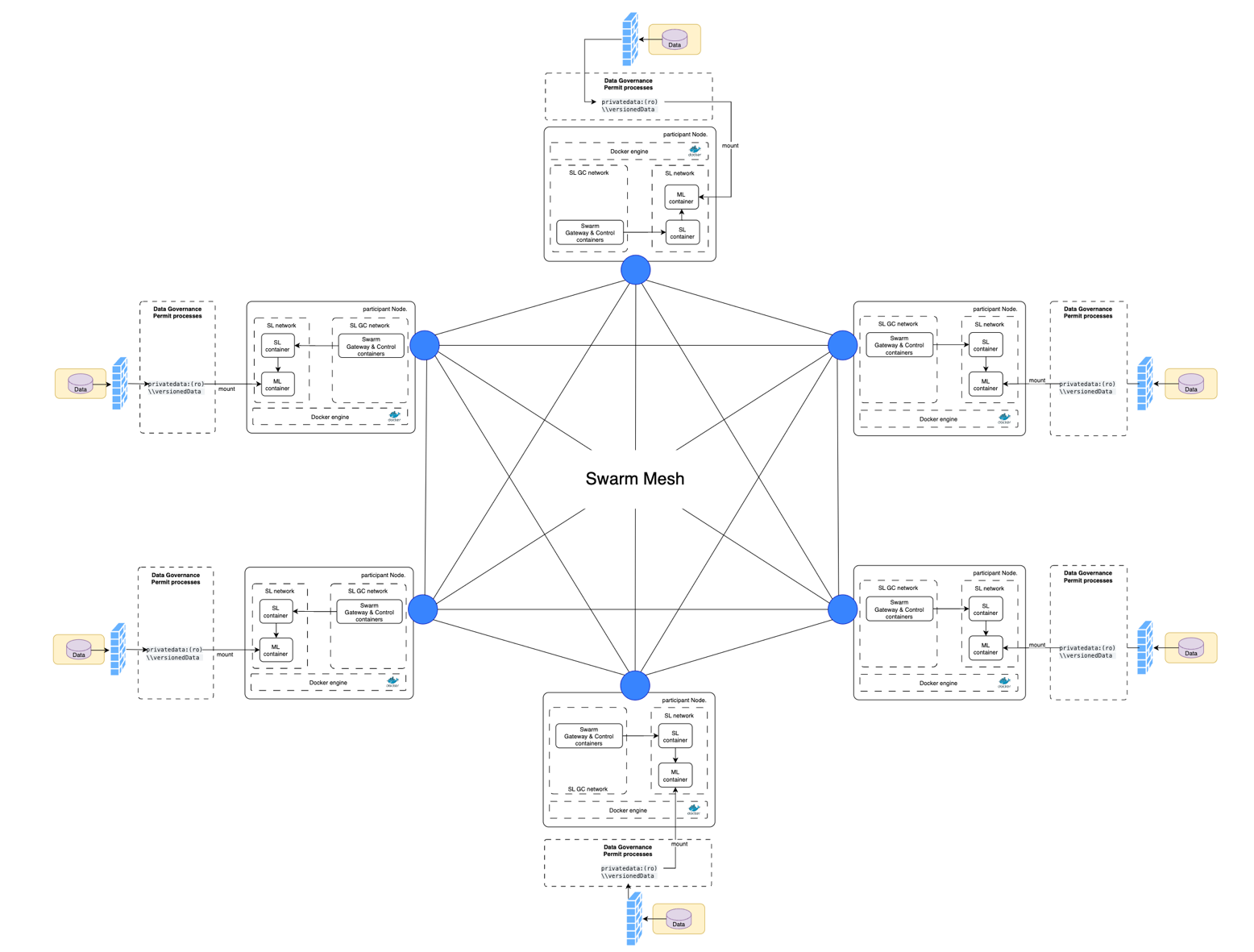
*

***Matrix 42HSL features***

| *Criteria* | *Details* |
| --- | --- |
| ***Security and Privacy*** | 42HSL deploys different levels of security, access control (authorization and authentication) on network and also on workload level with mTLS short-lived certificates. Within 42HSL, data is encrypted. Data is visited only at the sources from the swarm participant |
| ***Compliance and Regulatory Adherence*** | 42HSL is compliant to GDPR, HIPAA |
| **Interoperability and Extensibility** | **42HSL** concentrates on decentralized AI training. It is extensible for various types of standard connectors for heterogeneous data sources with various data types (including HL7, FHIR, OMOP). Per use case the appropriate open-standards data connector is deployed at the data source to allow integration with existing clinical systems. |
| ***Data Quality and Integrity*** | **42HSL** leverages systems such as lamindb (<https://github.com/laminlabs/lamindb>, data framework for biology) for data lineage and integration in upstream data processing pipelines. |
| ***Usability and Accessibility*** | **42HSL** deploys automated workflows and data pipelining with minimal requirements for user interaction. Dashboards are provided for the trainings and system state. |
| ***Scalability and Performance*** | **42HSL** by its architecture is a system leveraging its decentralized nature. Datasets are segmented and sharded by design to minimized potential system bottlenecks.  Performance metrics across the deployed swarm nodes are in place to continuously monitor performance. |
| ***Collaboration and Sharing Capabilities*** | **42HSL** provides a Metadata Hub for detection of potential analysis and training targets in the collaborative training using swarm principles. Data is by design not shared, only visited throughout the collaborative training process. Access to data is completely under control of the data provider at the source of the data. |
| ***Cost and Sustainability*** | Cost of operations not available yet. **42HSL**, is an automated system which is designed for large scale deployment for lowest operations cost possible. It is leveraging existing open-standards and open-source for long-term sustainability in a variety of applications. |
| ***Ethical Considerations*** | Implementation has been successfully audited and certified within the finnish clinical system. |
| ***Innovation and Adaptability*** | **42HSL** is designed to be open to include emerging technologies. With the architecture based on common implementation schemes it can be adapted to requirements as they arise. |

***References***

*1 your references for relevant literature go here*

***Matrix 42HSL common challenges and mitigation***

| **Category** | **Description** |
| --- | --- |
| **Federated Queries Challenges** | The 42HSL platform focuses on ML/AI applications. An additional product for direct queries is currently under development. Within 42HSL, queries on individuals are *not* the target for the current platform. According to benchmarking experiments, datasets with sufficient dataset size will mitigate the issue of ‘same-patient’ data across nodes and have a negligible impact. |
| **Patient Privacy and Data Protection** | A common, upfront agreed upon data protection scheme is applied before training is started. Data is never shared but visited by the training algorithm. |
| **Organizational Policies** | The 42HSL platform is operated under a contractual agreement between all members of the decentralized SL network. The contract contains also the organizational policies concerning all aspects of the medical data, the algorithms and the network organization. Different maturity levels of applying technologies are overcome during the onboarding process. |
| **Data Transformation requirements** | Requirements for data harmonization, transformation, other steps of data pre-processing at the source is a prerequisite for decentralized ML/AI also within the 42HSL platform These requirements are done directly at the source and by the data holder/provider. |
| **Installation and Maintenance** | **42HSL** has an automated installation for cloud-native environments and is designed to be deployed in different settings in cloud, and on premise. |
| **Secure Deployment** | **42HSL** by design applies security measures for mTLS on workload base.  Deployed in finnish Hospital system on MS Azure. |
| **Understanding User Queries** | Researcher query Metadata catalogues to identify targets for collaborative training. As stated above, for exploratory data analytics (user queries), a complementary product is currently under development. |
| **Informatics and User Experience** | The 42HSL platform is a decentralized ML/AI platform with a metadata hub (availability of data at SL network member nodes) and a dashboard for community building of decentralized ML/AI applications within SL communities. Users are fully shielded from the informatics core as they would provide, preferably de-identified data using automated data preparation pipelines locally to each node within the SL network of the 42HSL platform. The platform is intended to run decentralized ML/AI learning and the automated integration into the training process for joint results. The results from such decentralized ML/AI learning are directly shared to all SL experiment member nodes. |
| **Complexity of 42HSL Software** | **42HSL** is designed in a micro-services cloud-native scheme. Integrations are done by APIs and leverage open standards. For the user, ID-secured access to the system is guaranteed via the local SL node platform entry, which connects to the metadata hub with the dashboard of the respective SL network community. The platform is agnostic to hardware environments as it requires only a virtualization environment. |
| **Incremental Updating Limitations** | **42HSL** aggregates data in the learning process and only shares the learnings, never source PII data. De-identification is done on the data plane layer at the source by the data owner and is the preferred scheme before data can be accessed by the **42HSL** platform. As such, source data is never exposed to the platform. |
| **Standardized Vocabularies and Flexibility** | The 42HSL platform utilizes standardized vocabularies via tools such as lamindb within the metadata hub, which can also provide data pre-processing prior to decentralized ML/SL training. Data quality and integration are also controlled on this local level. This part is not exposed to any other member within the 42HSL network. Moreover, metrics are introduced in the data pipelining to confirm the usage of standards. Non-compliance to the agreed standards within the collaborative learning may lead to exclusion of a participant. This is contractually guaranteed prior building SL networks, and all participants agree to this upfront. |

- ***does your community organize data challenges / platform challenges? Is there any benchmarking available?***

***References :***

1. please add references here or directly in the fields of the matrix

[*Building Trust in Medical Use of Artificial Intelligence - The****Swarm*** ***Learning****Principle.*](https://pubmed.ncbi.nlm.nih.gov/36969482/)

*Schultze JL.J CME. 2023 Jan 10;12(1):2162202. doi: 10.1080/28338073.2022.2162202. eCollection 2023.*

[***Swarm****immunology: harnessing blockchain technology and artificial intelligence in human immunology.*](https://pubmed.ncbi.nlm.nih.gov/35624333/)

*Schultze JL, Büttner M, Becker M.Nat Rev Immunol. 2022 Jul;22(7):401-403. doi: 10.1038/s41577-022-00740-1.*

[***Swarm*** ***Learning****for decentralized and confidential clinical machine****learning****.*](https://pubmed.ncbi.nlm.nih.gov/34040261/)

*Warnat-Herresthal S, Schultze H, Shastry KL, Manamohan S, Mukherjee S, Garg V, Sarveswara R, Händler K, Pickkers P, Aziz NA, Ktena S, Tran F, Bitzer M, Ossowski S, Casadei N, Herr C, Petersheim D, Behrends U, Kern F, Fehlmann T, Schommers P, Lehmann C, Augustin M, Rybniker J, Altmüller J, Mishra N, Bernardes JP, Krämer B, Bonaguro L, Schulte-Schrepping J, De Domenico E, Siever C, Kraut M, Desai M, Monnet B, Saridaki M, Siegel CM, Drews A, Nuesch-Germano M, Theis H, Heyckendorf J, Schreiber S, Kim-Hellmuth S; COVID-19 Aachen Study (COVAS); Nattermann J, Skowasch D, Kurth I, Keller A, Bals R, Nürnberg P, Rieß O, Rosenstiel P, Netea MG, Theis F, Mukherjee S, Backes M, Aschenbrenner AC, Ulas T; Deutsche COVID-19 Omics Initiative (DeCOI); Breteler MMB, Giamarellos-Bourboulis EJ, Kox M, Becker M, Cheran S, Woodacre MS, Goh EL, Schultze JL.Nature. 2021 Jun;594(7862):265-270. doi: 10.1038/s41586-021-03583-3. Epub 2021 May 26.*

***Data Modalities Supported by 42HSL***

*Usually, clinical research data platforms are designed to integrate and manage a wide range of data modalities to support biomedical research.*

42HSL is agnostic to ontologies and data sources. Per Use case the respective data is connected and used within the model training. The initial integration is done for scRNAsec and OMOP leveraging lamindb.

| **Category** | **Data Modality** | **Description** |
| --- | --- | --- |
| **Clinical Data** | Electronic Health Records (EHRs) | All, as long as these are meaningfully pre-processed for ML/AI applications:  *Structured data (please specify) and unstructured data (please add information on indexing / information extraction possible).* |
|  | Hospital Administrative Data | All, as long as these are meaningfully pre-processed for ML/AI applications:  *Admissions, discharges, transfers, billing codes, and insurance information.* |
| **Genomic Data** | Genomic Sequences | All, as long as these are meaningfully pre-processed for ML/AI applications:  *Whole genome, exome sequencing, targeted sequencing (please specify and provide references).* |
|  | Genotype Data | All, as long as these are meaningfully pre-processed for ML/AI applications:  *Single nucleotide polymorphisms (SNPs), copy number variations (CNVs). Please specify and provide references.* |
|  | Gene Expression Data | All, as long as these are meaningfully pre-processed for ML/AI applications:  *What types of transcriptomics data?* |
| **Imaging Data** | Radiology Images | All, as long as these are meaningfully pre-processed for ML/AI applications:  *MRI, CT, X-ray, ultrasound. Please specify and provide references if possible* |
|  | Pathology Images | All, as long as these are meaningfully pre-processed for ML/AI applications:  *Digital pathology slides, histology images. Please specify and provide references if possible* |
| **Phenotypic Data** | Disease Phenotypes | All, as long as these are meaningfully pre-processed for ML/AI applications:  *Disease characteristics, symptom severity, progression. Use of HPO or other controlled vocabularies for annotation? Please provide references if possible.* |
|  | Clinical Outcomes | All, as long as these are meaningfully pre-processed for ML/AI applications:  *Treatment responses, survival rates, recurrence. Please specify and provide references if possible.* |
| **Medication Data** | Prescription Records | All, as long as these are meaningfully pre-processed for ML/AI applications:  *(Co-)Medication names, dosages, administration routes, duration. Please add references if possible.* |
|  | Medication Adherence / Compliance | All, as long as these are meaningfully pre-processed for ML/AI applications:  *e.g. Refill records, patient self-reports.* |
| **Laboratory Data** | Lab Test Results | All, as long as these are meaningfully pre-processed for ML/AI applications:  *Blood tests, urine tests, microbiological cultures, biochemical assays. Biomarker measurements. Please provide references if possible.* |
| **Survey Data** | Questionnaires and Surveys | All, as long as these are meaningfully pre-processed for ML/AI applications:  *Patient health questionnaires, lifestyle surveys, mental health assessments. Please provide references if possible.* |
|  | Patient-Reported Outcomes | All, as long as these are meaningfully pre-processed for ML/AI applications:  *Pain scales, quality of life measures, functional status. Please provide references if possible.* |
| **Biomarker Data** | Proteomics | All, as long as these are meaningfully pre-processed for ML/AI applications:  *Protein expression, protein-protein interactions, post-translational modifications. Mass-Spec, MALDI whatsoever. Blood and urine proteomics? References ??* |
|  | Metabolomics | All, as long as these are meaningfully pre-processed for ML/AI applications:  *Metabolite profiles, metabolic pathways, lipidomics.* |
| **Environmental Data** | Lifestyle Factors | All, as long as these are meaningfully pre-processed for ML/AI applications:  *Diet, physical activity, workout schemata, smoking, alcohol consumption, substance (ab)use.* |
|  | Environmental Exposures | All, as long as these are meaningfully pre-processed for ML/AI applications:  *Air quality, water quality, exposure to toxins, occupational hazards.* |
| **Socioeconomic Data** | Social Determinants of Health | All, as long as these are meaningfully pre-processed for ML/AI applications:  *Education, income, employment status, housing, neighborhood characteristics.* |
| **Family History Data** | Genetic Risk Factors | All, as long as these are meaningfully pre-processed for ML/AI applications:  *Family history of diseases, pedigree analysis. Risk alleles, tumor gene panels etc.* |
| **Longitudinal Data** | Time-Series Data | All, as long as these are meaningfully pre-processed for ML/AI applications:  *Repeated measures over time, disease progression, treatment responses over time. Please provide information on how you organize information in time (from time stamp to longitudinal representation of patients).* |
| **Behavioral Data** | Behavioral Assessments | All, as long as these are meaningfully pre-processed for ML/AI applications:  *Cognitive tests, psychological assessments, behavioral interventions. Nutrition coaching? Please specify and provide references, if possible.* |
|  | Transcriptomics | All, as long as these are meaningfully pre-processed for ML/AI applications:  *mRNA levels, non-coding RNAs, alternative splicing events.* |
| **Pathway Data** | Biological Pathways | All, as long as these are meaningfully pre-processed for ML/AI applications:  *Signaling pathways, metabolic pathways. Mechanism graphs. Pathophysiology graphs (disease maps) ?* |
|  | Interaction Networks | All, as long as these are meaningfully pre-processed for ML/AI applications:  *Protein-protein interaction networks, gene regulatory networks. Co-expression networks ?* |

***References :***

1. please provide references to relevant publications / documentation here

**Built-in Workflows and Analysis Tools**

Does 42HSL contain built-in workflows and analysis tools that facilitate clinical and translational research?

**Workflow**

| **Feature** | **Description** |
| --- | --- |
| Patient Cohort Discovery | **42HSL** leverages systems such as lamindb (<https://github.com/laminlabs/lamindb>, data framework for biology) and similar tools for developing metadata catalogues including patient cohort data, which allow patient cohort discovery within established SL networks |
| Data Integration and Management | **42HSL** leverages systems such as lamindb (<https://github.com/laminlabs/lamindb>, data framework for biology) for local data integration and management. Data Pipelines are build per use case on CDMs, i.e. OMOP |
| Ontology Management | **42HSL** leverages systems such as lamindb (<https://github.com/laminlabs/lamindb>, data framework for biology) for ontology management |
| Data Extraction and Transformation | **42HSL** leverages systems such as lamindb (<https://github.com/laminlabs/lamindb>, data framework for biology) for data extraction and transformation as part of the data pre-processing pipelines at each SL node locally. |
| Security and Privacy Management | Policy based access control is included for user and services authentication and authorization. Source Data is by design only accessible to the data holder/provider. Data de-identification is always done locally and part of data pre-processing. Primary data are never exposed to the network. |

**References:**

1. references go here

**Analysis Tools**

| Query Interface | The 42HSL platform is a decentralized ML/AI training platform. It includes a meta data hub of all datasets (cohorts and alike) available within an SL network for querying for partners and datasets available. Exploratory data analytics is currently being developed as an additional product |
| --- | --- |
| Timeline Viewer | The 42HSL platform is a decentralized ML/AI training platform. It includes a meta data hub of all datasets (cohorts and alike) available within an SL network for querying for partners and datasets available. Exploratory data analytics is currently being developed as an additional product, time line viewing is seen as exploratory data analytics and is not part of the ML/AI engine of the 42HSL platform. |
| Statistics and Analytics | The 42HSL platform is a decentralized ML/AI training platform. It includes a meta data hub of all datasets (cohorts and alike) available within an SL network for querying for partners and datasets available. Exploratory data analytics is currently being developed as an additional product, statistics and analytics is seen as exploratory data analytics and is not part of the ML/AI engine of the 42HSL platform. |
| Plugin Framework | The 42HSL platform is a decentralized ML/AI training platform. It includes a repository for ML / AI algorithms (MLOps), which will be continuously extended. |
| Natural Language Processing (NLP) | Training utilizing NLP is currently performed in projects as part of the roadmap |
| Genomic Data Analysis | **42HSL** leverages systems such as lamindb (<https://github.com/laminlabs/lamindb>, data framework for biology) for data preprocessing. Genomic data analysis prior use for decentralized ML / AI applications is seen as preparatory explorative data analytics. This is performed only locally. |
| Temporal Querying | **42HSL** leverages systems such as lamindb (<https://github.com/laminlabs/lamindb>, data framework for biology) for data preprocessing. Temporal questing on local data prior use for decentralized ML / AI applications is seen as preparatory explorative data analytics. This is performed only locally. |
| Data Visualization | **42HSL** contains a dashboard, which includes visualization of ML /AI training results . Classical exploratory data analytics tools for visualization are seen as part of the data pre-processing procedures. |
| Export and Reporting | All ML / AI training results are available for all SL nodes being part of the AI / ML training network. The 42HSL platform allows for export of AI / ML results for further analysis or reporting purposes in formats compatible with other statistical and data analysis software. |

***References***

*1.*references go here

| **Integration with Other Tools** | R / BioConductor and Python Integration | **42HSL** leverages systems such as lamindb (<https://github.com/laminlabs/lamindb>, data framework for biology) for data preprocessing. R / BioConductor and Python applications can be done locally during local exploratory data analytics prior to use for decentralized ML / AI applications |
| --- | --- | --- |
|  | Integration with Clinical Trial Management Systems (CTMS) | The integration into CTMS is part of the roadmap of extensions of 42HSL |
|  | Integration with Electronic Health Records (EHR) | Local data pre-processing is intended to increase data quality. Currently, the 42HSL platform utilizes highly standardized CDMs for ingestion into decentralized AI / ML applications. Direct integration with often low quality EHRs is currently under evaluation |

References:

1. references go here

**Support for Semantic Integration**

Does 42HSL support semantic integration through the use of terminologies, ontologies, and common data models? Such as:

1. **Terminologies and Ontologies**: Can 42HSL integrate with standard medical terminologies and ontologies such as ICD, SNOMED CT, LOINC, and others. This ensures consistent data representation and facilitates interoperability.?

See answers above. **42HSL** leverages systems such as lamindb (<https://github.com/laminlabs/lamindb>, data framework for biology) for integration of terminologies and ontologies. Current use cases include different data spaces with different terminologies and ontologies. New ontologies are currently developed for immune assays including FACS and scRNA-seq

1. **Common Data Models (CDMs)**: Can 42HSL work with various common data models like the Observational Medical Outcomes Partnership (OMOP) CDM, enabling data standardization and easier data sharing across institutions.?

Currently, the 42HSL platform utilizes highly standardized CDMs for ingestion into decentralized AI / ML applications. OMOP models are already in use Any other CDM is easily implementable, since the platform is agnostic to the data space

1. **Ontology Management**: Does the platform include tools for ontology management, allowing users to customize and extend the ontologies as needed to fit their specific research requirements​?

Schemas can be loaded from available public ontologies for use within the platform. Here the 42HSL platform leverages systems such as lamindb (<https://github.com/laminlabs/lamindb>, data framework for biology) for integration and management of terminologies and ontologies.

**References** :

1. References go here
