# Supplementary material for: Selecting medical research data platforms for translational biomedical research: a five-tier overview and requirement-weighted assessment framework
Source: Front Digit Health. 2026 Jun 17;8:1814015. doi: 10.3389/fdgth.2026.1814015 (PMC13319098; doi:10.3389/fdgth.2026.1814015)
Supplement: Supplementary file 5 [file Supplementaryfile5.docx]

Apheris Compute Gateway

# Deployment and usage

The Apheris product is a scalable, secure and governed federation solution for connecting and building data networks. Apheris was founded in 2019 by Robin Röhm and Michael Höh and is funded by various leading investors including LocalGlobe, Dig Ventures, Octopus Ventures and Heal Capital.

Apheris' core product is the Compute Gateway, which is currently deployed in various European and North American hospitals as well as in top 10 pharmaceutical companies. The Compute Gateway is primarily used in collaborative use cases to build data networks, collaboratively train machine learning models, or enable model benchmarking in an IP-preserving manner. Apheris provides the technology infrastructure for the AI Structural Biology Consortia, one of the largest collaborative model training projects in drug discovery today.

References:

1. <https://www.crunchbase.com/organization/apheris-ai/company_financials>
2. <https://medically.gene.com/global/en/unrestricted/neuroscience/ECTRIMS-2023/ectrims-2023-poster-oh-utility-and-implementation.html%e2%80%8b.html>
3. <https://www.apheris.com/industries/aisb>

# Apheris Components


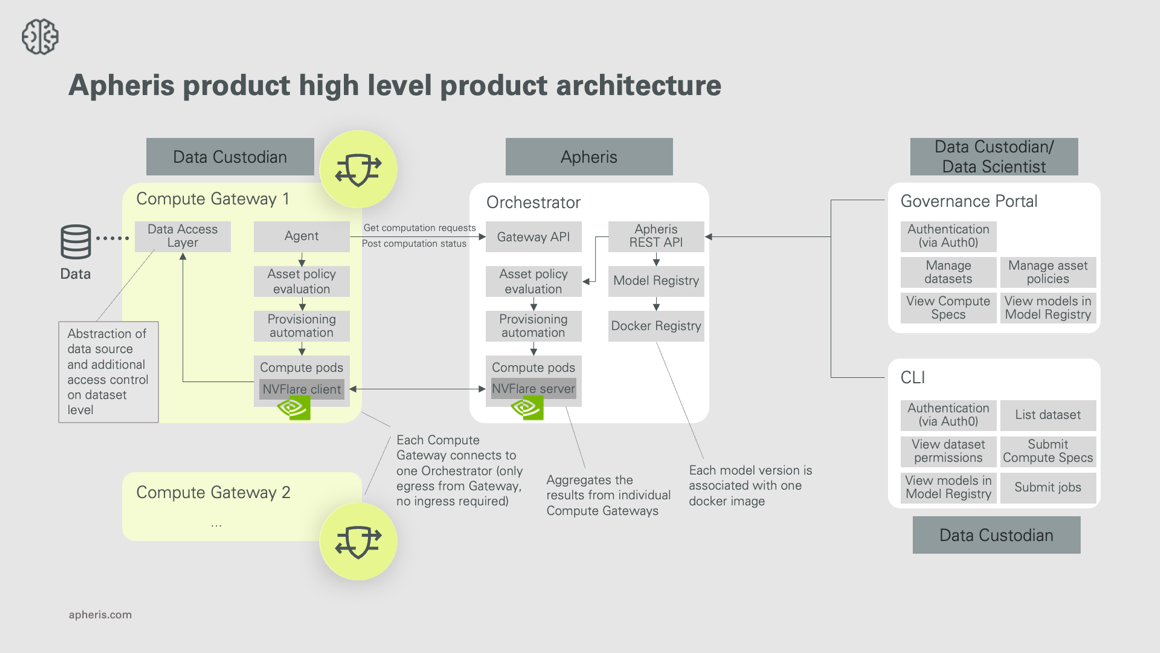


Reference:

<https://www.apheris.com/docs/general/architecture.html>

# Matrix table for the Apheris platform features

| **Criteria** | **Details** |
| --- | --- |
| Security and Privacy  (1, 2, 3, 4, 5, 6, 7, 8,9) | - Data Encryption: Apheris ensures data is encrypted at rest (AES-256) and in transit (TLS 1.2). - User Access Control: The product employs robust role-based access control and supports enterprise-grade single sign-on (SSO). Identity management for users managed by Apheris is provided by Auth0. - Data Privacy: Only a data provider with specific user permissions has access to raw data. External users will never have direct access to or see raw data. For privacy-preserving preparation and local simulations of computations, Apheris supports the upload of synthetic data (Dummy Data) for each registered dataset that can be downloaded by data scientists to perform data discovery, experimentation and simulation tasks while preserving privacy. - Data Access Control: In addition to role-based access control for users, Apheris provides so-called Asset Policies to supervise and control access to data. Such policies allow for granular control of who has access to which data with what model and additional requirements such as model permissions and optional privacy controls. - Platform Security: Apheris follows several security-by-design principles. For example: - **Egress-only architecture: A** data provider never has to allow inbound connections to their environment. The same goes for a data scientist connecting to the network via the Apheris CLI. - **Credentials & Cryptography:** Apheris uses AWS KMS on the Orchestrator to generate and rotate the keys used to encrypt various elements, such as S3 objects, EKS secrets, and EBS volumes - Software development processes follow industry best practices and standards - Cryptographic Signatures: Apheris supports the cryptographic signing of user-generated data access controls, providing an additional layer of security to prevent the possibility of tampering with Asset Policies - Privacy: Apheris supports various privacy-enhancing technology (PET) plug-ins to protect sensitive data, including Differential Privacy, Homomorphic Encryption and various other forms. Apheris ensures computations required to incorporate such PETs are only executed when compliant. |
| Compliance and Regulatory Adherence  (10, 11, 12) | - Apheris enables users to collaborate in compliance with various privacy regulation such as HIPAA, GDPR or local regulations such as CCPA by various features and measures such as role and purpose-based access control, data locality preservation, model-specific privacy-enhancing technology (PET) plug-ins. - The product enables data-centric collaboration in a zero-trust environment by allowing a data provider to control computations at the algorithmic level. In addition. It also allows a data provider and a data consumer to protect themselves against multiple attack vectors - Apheris provides the means to achieve compliant collaboration but is not responsible for the actions of individual collaborators. A data provider remains the data custodian. |
| Interoperability and Extensibility  (13, 14, 15) | - The Compute Gateway is data and model agnostic, any type of data, regardless of format or standard, can be registered in the Gateway and analyzed with any data-driven algorithm - Communicating with and running in the Compute Gateway can be thought of as Docker execution with an API-based approach. If it can run in a Docker container, it can run in Apheris - Analytics or ML capabilities can be extended/customized to any data-driven algorithm using the Apheris Model Registry - The product can be integrated into any workflow and setup (PyTorch, Python, etc.) via a programmatic interface (Apheris CLI, Python CLI). |
| *Data Quality and Integrity*  *(16)* | - Data providers can register synthetic data, enabling privacy-preserving data discovery, experimentation, and preprocessing by a data scientist. - All interactions with raw data are logged to provide audit trails and to monitor and demonstrate compliance. - Data scientists can use the Compute Gateway to define and run custom computations to verify data integrity and improve data quality. |
| *Usability and Accessibility*  (17, 18) | - Apheris provides optimized interfaces for all user groups - Data custodians can control all collaboration through a GUI called Governance Portal. All daily activities, tasks and configurations can be performed there - Data Scientists can use the Apheris CLI or Python CLI to interact with the data network: simulate & test computations, prepare computations, receive results and logs. Data scientists can easily integrate with Jupyter or Deepnote notebooks, for example, to stay in their preferred environment and workflow. - Apheris complies with a11y standards for optimal accessibility |
| *Scalability and Performance*  (19, 20) | - The Compute Gateway is optimized to run in resource-constrained on-premises environments as well as modern, high-performance GPU cloud environments. Apheris is based on Docker and Kubernetes and is therefore infrastructure agnostic. For low resource environments, a lightweight Kubernetes distribution (K3s.io) is provided. - The Compute Gateway can run any type of analytic and ML workload, and there is no performance penalty for actually running a compute job in a data provider environment. Performance is, of course, dependent on the infrastructure resources and compute capabilities provided at that location. As with most federated learning solutions, compute orchestration (distribution, aggregation) and on-premises or cloud resource provisioning can add overhead to the execution of individual compute jobs. |
| *Collaboration and Sharing Capabilities*  *(21, 22, 23, 24, 25, 26)* | - The Apheris product supports any analytical workflow across any number of sites for collaborative, secure research on e.g. protein, omics or patient-level data. - Asset Policies allow the data provider to define the requirements for compliant computations for a user on a specific dataset and with a specific model (purpose-based access control). In addition, the data provider can control models at the algorithmic level and require the addition of privacy-enhancing technologies such as differential privacy or homomorphic encryption. - A data scientist can configure a computation to meet Asset Policy requirements through compute specifications, assess the status of individual compute jobs, and obtain results through compute logs. - For data discovery, compute preparation and local simulation, Apheris provides a solution for data providers to upload a non-sensitive synthetic version of a dataset for data scientists to download. - The Compute Gateway ensures that only computations that comply with established asset policies can be executed. - The product provides fine-grained access control, multi-factor authentication, and integration with single sign-on solutions. |
| *Cost and Sustainability*  *(27,28)* | - Cost-Effectiveness: Apheris offers a broad and continually improving feature set covered under the same customer agreement. - Apheris is flexible in finding the optimal solution with the overarching goal of making collaboration happen. - Sustainability: Apheris is funded by leading healthcare and AI investors, has market-leading pharmaceutical companies and data aggregators as customers, is active in public funding projects, and provides technology to one of the largest drug discovery consortia. |
| *Ethical Considerations*  *(29)* | - With the Compute Gateway, the data provider retains full control of the data. A change in patient consent status can be reflected in records at any time. - Ethical Review: Apheris already provides processes for ethical review (Asset Policies, Audit Logs) and more features are on the roadmap. |
| *Innovation and Adaptability*  *(30, 31* | - Apheris is docker-based and can therefore quickly adapt to new data standards, ML models and analytical needs. The product is API-based and uses a modular approach to quickly adapt to customer needs and new project requirements. - Users can already quickly adapt to various local regulatory needs. If other needs should arise which are currently not being covered, the modular approach to Computational Governance and algorithmic control can be easily extended or changed. - Apheris integrates with open-source and strives to be federation engine agnostic. Today, Apheris integrates with NVIDIA FLARE and Flower – two very popular federation engines. |

References

1. <https://www.apheris.com/docs/how-to/manage-users.html>
2. <https://www.apheris.com/docs/how-to/account-setup-and-login.html>
3. <https://www.apheris.com/docs/data-custodian/dummy-data.html>
4. <https://www.apheris.com/docs/data-custodian/asset-policies-overview.html>
5. <https://www.apheris.com/docs/general/architecture.html#security-by-design>
6. <https://www.apheris.com/resources/blog/a-devsecops-journey-to-secure-and-standardize-github-repositories>
7. <https://www.apheris.com/docs/data-custodian/sign-asset-policies.html>
8. <https://www.apheris.com/docs/data-custodian/controls-for-apheris-stats.html>
9. <https://www.apheris.com/docs/data-custodian/privacy-controls.html>
10. <https://www.apheris.com/docs/general/computational-governance.html>
11. <https://medically.gene.com/content/dam/pdmahub/restricted/neurology/ectrims-2023/ECTRIMS-2023-poster-oh-utility-and-implementation.pdf>
12. <https://www.bsi.bund.de/SharedDocs/Downloads/EN/BSI/Publications/Studies/KI/P464_Provision_use_external_data_trained_models.pdf?__blob=publicationFile&v=7>
13. <https://www.apheris.com/docs/how-to/python-api-reference.html>
14. <https://www.apheris.com/docs/general/custom-models.html>
15. <https://www.apheris.com/docs/general/introduction.html>
16. <https://www.apheris.com/docs/data-custodian/dummy-data.html>
17. <https://www.apheris.com/docs/general/concepts-and-features.html#governance-portal>
18. <https://www.a11yproject.com/>
19. <https://www.apheris.com/docs/gateway/endpoints.html?h=k3s#gateway-installer>
20. <https://medically.gene.com/content/dam/pdmahub/restricted/neurology/ectrims-2023/ECTRIMS-2023-poster-oh-utility-and-implementation.pdf>
21. <https://www.apheris.com/docs/data-custodian/asset-policies-overview.html>
22. <https://www.apheris.com/docs/data-custodian/privacy-controls.html>
23. <https://www.apheris.com/docs/data-custodian/dummy-data.html>
24. <https://www.apheris.com/docs/data-science-and-ml/simulating-and-running-ml-workloads.html>
25. <https://www.apheris.com/docs/how-to/manage-users.html#roles-and-permissions>
26. <https://www.apheris.com/docs/how-to/manage-users.html#enforcing-multi-factor-authentication-mfa-for-your-organization>
27. <https://www.apheris.com/resources/blog/apheris-3-3-improved-scalability-federated-logistic-regression>
28. <https://www.apheris.com/industries/consortia>
29. <https://www-preview.apheris.com/docs/how-to/work-with-compute-gateway-logs.html>
30. <https://www.apheris.com/docs/general/architecture.html#running-computations-on-the-gateway>
31. <https://arxiv.org/html/2407.00031v2>

# Matrix table for the Apheris Compute Gateway’s common challenges

| **Category** | **Description** |
| --- | --- |
| **Federated Queries Challenges**  **(1)** | By definition, federation applies to use cases where it is not possible to centralize and view the raw data. This can be particularly challenging for data exploration and analytics dominated workloads where insights are required at the raw data level, including personally identifiable information. |
| **Patient Privacy and Data Protection**  **(2)** | Our software provides privacy controls that can be configured and set by the data custodian to suit their particular data, use case, and workload.  Guaranteed privacy for LLM is still an open research topic. We provide expert guidance on how best to deploy and use these algorithms while still maintaining privacy, but security is not 100% guaranteed, and while it can be minimized, there is always some inherent risk in using these types of complex technologies. |
| **Organizational Policies**  **(3,4)** | The Apheris team has extensive knowledge in deploying our product in high security environments such as pharmaceutical IT infrastructure and EU as well as American hospitals, while meeting information security, data management and privacy requirements. Due to our modular API approach and other product features, we can usually easily map to internal requirements. However, coordination and technical implementation can still be time consuming due to the extremely stringent security requirements in such environments. |
| **Data Transformation requirements** | We do not require the data provider to harmonize or transform data in any particular manner. A data consumer might want to harmonize data across sites and can use various approaches supported by the Apheris product, such as a programmatic custom interface, to achieve harmonized datasets across sites. |
| **Installation and Maintenance**  **(5)** | We do not require the data provider to harmonize or transform the data in any particular way. A data consumer may wish to harmonize data across locations and may use various approaches supported by the Apheris product, such as a custom programmatic interface, to achieve harmonized datasets across locations. |
| **Secure Deployment**  **(4,5)** | Deploying software in on-premises environments is not trivial, as security requirements, network and firewall configurations vary from site to site. However, Apheris has achieved a number of successful on-premises deployments because our security architecture strives to minimize these efforts by following a security-by-design architecture, which can be found in more detail in our documentation. |
| **Understanding User Queries** | While our product UX strives for simplicity, data scientists should at least be familiar with command line interfaces and/or scripting in a Pythonic stack. |
| **Informatics and User Experience** | We abstract the informatics core away from the user. Data providers can use the Governance Portal (web-based UI) and data consumers can use the Apheris CLI or Python CLI. Our software takes care of orchestration, resource provisioning, audit logging, and secure aggregation. |
| **Complexity of Apheris Software** | Our software abstracts away most of the complexity associated with a federated infrastructure. Adapting to new data types and analytical algorithms is fairly straightforward, especially if such models are already available in the Model Registry. We allow users to federate their own algorithms from scratch if they wish, using our custom code functionality, although it should be noted that this may require a fairly deep technical understanding of federation techniques. |
| **Incremental Updating Limitations**  **(6)** | Datasets and Asset Policies can be updated by the user at any time and on their own terms. Because data does not have to move, custodians can update, modify, or deregister records at any time. The Compute Gateway itself can be updated without problems such as data re-identification, database fragmentation or exposure, or temporary data exposure. |
| **Standardized Vocabularies and Flexibility** | Our goal is to make the product as flexible as the user needs it to be. Data scientists can define their own custom computational jobs that allow them to map data to standardized vocabularies. |

References

1. <https://www.bsi.bund.de/SharedDocs/Downloads/EN/BSI/Publications/Studies/KI/P464_Provision_use_external_data_trained_models.pdf?__blob=publicationFile&v=7>
2. <https://www.apheris.com/docs/data-custodian/controls-for-apheris-stats.html>
3. <https://medically.gene.com/content/dam/pdmahub/restricted/neurology/ectrims-2023/ECTRIMS-2023-poster-oh-utility-and-implementation.pdf>
4. <https://www.apheris.com/docs/general/architecture.html#security-by-design>
5. <https://www.apheris.com/docs/gateway/deploy-on-a-single-instance.html>
6. <https://www.apheris.com/docs/data-custodian/managing-datasets.html>

# Data Modalities Supported by Apheris

Apheris is data model agnostic, and both analytical and machine learning workloads can be run against any data type and modality. The primary data modalities used in Apheris to date include: tabular (patient-level records), text, images, omics.

| **Category** | **Data Modality** | **Description** |
| --- | --- | --- |
| **Clinical Data** | Electronic Health Records (EHRs) | Supported |
|  | Hospital Administrative Data | Supported |
| **Genomic Data** | Genomic Sequences | Supported |
|  | Genotype Data | Supported |
|  | Gene Expression Data | Supported |
| **Imaging Data** | Radiology Images | Supported |
|  | Pathology Images | Supported |
| **Phenotypic Data** | Disease Phenotypes | Supported |
|  | Clinical Outcomes | Supported |
| **Medication Data** | Prescription Records | Supported |
|  | Medication Adherence / Compliance | Supported |
| **Laboratory Data** | Lab Test Results | Supported |
| **Survey Data** | Questionnaires and Surveys | Supported |
|  | Patient-Reported Outcomes | Supported |
| **Biomarker Data** | Proteomics | Supported |
|  | Metabolomics | Supported |
| **Environmental Data** | Lifestyle Factors | Supported |
|  | Environmental Exposures | Supported |
| **Socioeconomic Data** | Social Determinants of Health | Supported |
| **Family History Data** | Genetic Risk Factors | Supported |
| **Longitudinal Data** | Time-Series Data | Supported |
| **Behavioral Data** | Behavioral Assessments | Supported |
|  | Transcriptomics | Supported |
| **Pathway Data** | Biological Pathways | Supported |
|  | Interaction Networks | Supported |

***References :***

1. [***https://www.apheris.com/docs/data-custodian/managing-datasets.html#managing-datasets***](https://www.apheris.com/docs/data-custodian/managing-datasets.html#managing-datasets)

# Built-in Workflows and Analysis Tools

Apheris has a modular approach and provides the building blocks for creating customized workflows and data analysis tasks.

**Workflow**

| **Feature** | **Description** |
| --- | --- |
| Patient Cohort Discovery  (1) | Users can define custom jobs to perform tasks relevant to patient cohort discovery when relevant data is registered with the Compute Gateway, leveraging our ability to run analytics workloads on unstructured and structured data. |
| Data Integration and Management  (2,3,4,5) | Apheris supports three options for data harmonization on the foundation of the programmatic interface for data scientists, ability to persist intermediate results on the Gateway and run both federated jobs across sites or custom jobs on one site. The options are   - Harmonization using Apheris Statistics library - Define own custom workflows - Integrate with 3rd party custom tools   Preprocessed datasets can be persisted via the Derived Datasets feature to be readily available for further analysis tasks. |
| Ontology Management | Apheris does not provide ontology management out-of-the-box |
| Data Extraction and Transformation  (2,3,4,5) | Apheris supports three options for data harmonization based on the programmatic interface for data scientists, the ability to persist intermediate results on the Compute Gateway, and the ability to run both federated jobs across sites or custom jobs on a single site. The options are   - Harmonization using the Apheris statistics library - Define your own custom workflows - Integrate with custom 3rd party tools   Preprocessed datasets can be persisted using the Derived Datasets feature to be readily available for further analysis tasks. |
| Security and Privacy Management  (6,7,8,9,10,11,12, 13, 14) | Security for the Compute Gateway and protection of the Apheris platform   - Role-based access control based on the principle of least access necessary - Purpose-based access control: Data providers can specify on a per-use basis who can perform which computations on a dataset and whether additional privacy controls must be met. - Cryptographic signatures: Apheris provides cryptographic signatures to ensure that asset policies controlling data access are tamper-proof. - Identity management can be provided by Apheris or Apheris can interface with existing single sign-on systems. - Support for multi-factor authentication - Egress-only architecture: The Compute Gateway and Apheris CLI only allow outbound connections, not inbound, to ensure that direct access to the data custodian and data scientist environment is not possible. - Data is encrypted in transit (TLS 1.2+) and at rest (AES-256) - Access is monitored via AWS Cloudtrail logs, VPC flow logs, DNS logs, S3 object logs, Kubernetes logs, and system events. - Additional internal security measures taken by the Apheris team via AWS Guard Duty and GuardDuty malware protection, EKS Runtime Monitoring, and IAM Access Analyzer. - Apheris is ISO 27001 certified and SOC 2 Type 1 audited (Apheris is currently undergoing SOC 2 Type 2)   Privacy Measures   - Data Locality: Data always stays within the data provider's environment - Federated computation: Computations are sent to the data and only results (aggregates, model weights) are returned - Asset Policies: Computations can be controlled at the algorithmic level - Privacy-enhancing technology (PET) add-ons: Data providers can require the use of privacy-enhancing technologies for each user and model. - Data control: Data providers maintain full control of their data and can adjust the data set and access to it at any time. This includes updating patient consent to comply with GDPR requirements. - Apheris ensures that only compliant computations can be performed, which the data provider has specified as privacy-preserving. - Synthetic Data: Apheris supports the upload of a non-sensitive version of a given dataset to enable privacy-preserving preparation and simulation of federated computations prior to computation runs on the real data. |

**References:**

1. <https://www.apheris.com/docs/general/custom-models.html>
2. <https://www.apheris.com/docs/how-to/statistics-reference.html>
3. <https://www.apheris.com/docs/general/custom-models.html>
4. <https://www.apheris.com/docs/how-to/python-api-reference.html>
5. <https://www.apheris.com/docs/data-custodian/derived-datasets.html>
6. <https://trust.apheris.com/>
7. <https://www.apheris.com/docs/how-to/account-setup-and-login.html>
8. <https://www.apheris.com/docs/how-to/manage-users.html>
9. <https://www.apheris.com/docs/data-custodian/sign-asset-policies.html>
10. <https://www.apheris.com/docs/general/architecture.html#security-by-design>
11. <https://trust.apheris.com/?itemUid=92ff33fd-666e-49f9-8cc7-c8a3ba9fd4d7&source=search>
12. <https://www.apheris.com/docs/general/concepts-and-features.html#federated-learning>
13. <https://www.apheris.com/docs/data-custodian/asset-policies-overview.html>
14. <https://www.apheris.com/docs/general/concepts-and-features.html#dataset>

## Analysis Tools

| Query Interface  (1, 2, 3) | Researchers and data scientists use a programmatic interface to interact with Apheris (Apheris CLI, Python API). To prepare a computation, users can select a model from the Apheris Model Registry, a repository of pre-ported and federation-ready models. Custom models and workflows can also be added to an organization's Model Registry. Apheris will continue to add models to the general Model Registry to further extend out-of-the-box query capabilities. |
| --- | --- |
| Timeline Viewer | No specific or out-of-the-box capabilities |
| Statistics and Analytics  (4, 5) | Apheris provides an Apheris Statistics package that includes a complete set of statistical functions and function groups for basic and advanced analysis, as well as out-of-the-box multiple regression models. |
| Plugin Framework  (6) | Apheris is Docker-based, so it can run anything that can run a Docker image, including external plug-ins and analysis tools. |
| Natural Language Processing (NLP)  (7) | Apheris can run any machine learning model through custom models. Apheris uses NVIDIA Flare (and soon Flower) as its federation engine, so it easily integrates with Hugging Face, BioNeMo, and other model libraries. |
| Genomic Data Analysis  (7) | Users can easily select any GNN, RNN, XGBoost or generative model from Hugging Face, BioNeMo or use their own models to run on the data. Apheris is model agnostic and can be extended to meet individual needs (see Custom Models). |
| Temporal Querying  (7) | This is something a user could do with custom code functionality. |
| Data Visualization  (8) | Our tool consists of a modular, programmatic access. 3^rd^ party visualization tools can usually be easily integrated with our APIs. |
| Export and Reporting  (8,9,10) | After a computation, a data scientist can download the results and associated logs to their local environment. For the data custodian, Apheris provides audit logs that can be persisted to a data custodian's preferred login setup. All interactions with the data are logged for the data custodian to monitor and demonstrate compliance. |

***References***

1. <https://www.apheris.com/docs/data-science-and-ml/apheris-cli-hello-world.html>
2. [*https://www.apheris.com/docs/general/model-registry.html*](https://www.apheris.com/docs/general/model-registry.html)
3. [*https://www.apheris.com/docs/general/compute-specs.html*](https://www.apheris.com/docs/general/compute-specs.html)
4. [*https://www.apheris.com/docs/how-to/statistics-reference.html*](https://www.apheris.com/docs/how-to/statistics-reference.html)
5. [*https://www.apheris.com/docs/data-science-and-ml/model-registry/regression-models.html*](https://www.apheris.com/docs/data-science-and-ml/model-registry/regression-models.html)
6. [*https://www.apheris.com/docs/general/custom-models.html*](https://www.apheris.com/docs/general/custom-models.html)
7. <https://www.apheris.com/docs/how-to/python-api-reference.html>
8. [*https://www.apheris.com/docs/data-science-and-ml/simulating-and-running-ml-workloads.html#downloading-results*](https://www.apheris.com/docs/data-science-and-ml/simulating-and-running-ml-workloads.html#downloading-results)
9. [*https://www.apheris.com/docs/data-science-and-ml/simulating-and-running-ml-workloads.html#downloading-results*](https://www.apheris.com/docs/data-science-and-ml/simulating-and-running-ml-workloads.html#downloading-results)
10. [*https://www.apheris.com/docs/gateway/monitoring-compute-gateways.html*](https://www.apheris.com/docs/gateway/monitoring-compute-gateways.html)

| **Integration with Other Tools**  **(1)** | R / BioConductor and Python Integration | Apheris provides direct integration with Python and enables workloads in R. Because Apheris is Docker-based, any custom analytic workload can be run. |
| --- | --- | --- |
|  | Integration with Clinical Trial Management Systems (CTMS) | We do not provide integration with Clinical Trial Systems. |
|  | Integration with Electronic Health Records (EHR) | We do not provide seamless EHR integration, although it is certainly possible to work with EHR records through our software. |

References:

1. <https://www.apheris.com/docs/how-to/python-api-reference.html>

# Support for Semantic Integration

Does Apheris support semantic integration through the use of terminologies, ontologies, and common data models?

Terminologies and Ontologies: Can Apheris integrate with standard medical terminologies and ontologies such as ICD, SNOMED CT, LOINC, and others. This ensures consistent data representation and facilitates interoperability.?

- - Apheris and the Compute Gateway are data type, data model, and data standard agnostic. Following common data standards simplifies the work in Apheris in a multi-site compute use case. If standards differ between sites, Apheris provides the ability to run data harmonization workloads in different ways and persist these harmonized datasets for efficient analytics & ML workflows. Several common data models and terminologies are supported.

Common Data Models (CDMs): Can Apheris work with various common data models like the Observational Medical Outcomes Partnership (OMOP) CDM, enabling data standardization and easier data sharing across institutions.?

- Cf. Above

Ontology Management: Does the platform include tools for ontology management, allowing users to customize and extend the ontologies as needed to fit their specific research requirements​?

- Apheris doesn’t include tools for managing ontologies
