# Supplementary material for: Selecting medical research data platforms for translational biomedical research: a five-tier overview and requirement-weighted assessment framework
Source: Front Digit Health. 2026 Jun 17;8:1814015. doi: 10.3389/fdgth.2026.1814015 (PMC13319098; doi:10.3389/fdgth.2026.1814015)
Supplement: Supplementary file 6 [file Supplementaryfile6.docx]

*Here comes the “empty” form to add your information. All my comments are labeled in blue. They can be removed. Would be good if you chose another color for your input.*

***INSIGHT platform***

***Deployment and Usage****:*

*< please fill-in all relevant information on YOUR PLATFORM with respect to deployment and usage in academic and commercial installations >*

***References:***

1. please add references for installations of < YOUR PLATFORM >
2. in particular please refer to scientific publications describing these installations

**INSIGHT components**

*à HERE GOES A CARTOON WITH THE MAJOR FEATURES / THE ARCHITECTURE OF YOUR PLATFORM*


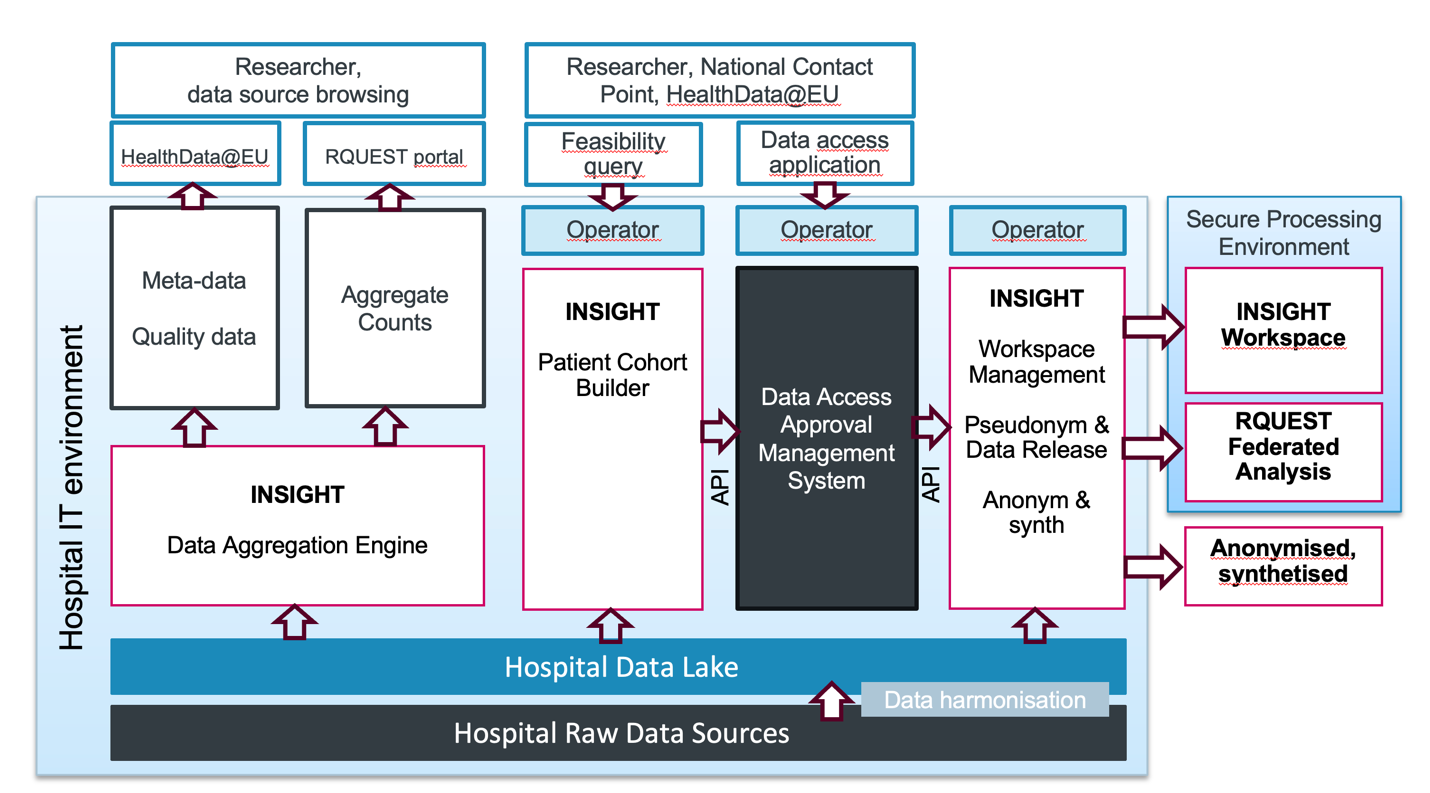


*Figure 1: Simplified scenario for EHDS regulated hospital. Unidentified researcher can browse pre-aggregated datasets but for feasibility queries and data access applications researcher must be identified and reason for asking must be provided. Pseudonymised data can only be released to Secure Processing Environment (SPE) defined by EHDS. According to the latest EHDS specification ‘Operator’ is independent governmental authority (Health Data Access Body) i.e all availability queries and data access applications must be approved by authority.*

***Reference:***

[*If*](https://community.i2b2.org/wiki/display/BUN/Analysis+of+Populations%3A+Install+Guide?preview=/41746456/41746457/worddavf4910d141b05e4133d5e3adb0cc77a8b.png) *the platform architecture has been published, please add a reference here*

***Matrix INSIGHT features***

| *Criteria* | *Details* |
| --- | --- |
| ***Security and Privacy*** | ***Data Encryption:*** *BC Platforms ensures data encryption both in transit and at rest across all supported deployment models (cloud, on-premises, and hybrid), safeguarding sensitive information. Access Controls: Role-based access control (RBAC) and comprehensive user management features provide granular permissions for controlling data access.*  ***Data Masking & Anonymization:*** *The platform offers multiple layers of data protection, including ways to integrate pseudonymization and anonymization techniques, to protect personally identifiable information (PII) and support compliance with data privacy regulations. Integrated logging and monitoring mechanisms ensure that security events are traceable and auditable.* |
| ***Compliance and Regulatory Adherence*** | ***Global Compliance:*** *BC Platforms adheres to a range of international compliance standards, including ISO 27001, GDPR, and HIPAA. Our architecture and processes ensure compliance with local and international regulations, including restrictions on data transfer and sovereignty.*  ***Audits and Certifications:*** *The platform undergoes regular internal and external audits and third-party penetration testing to validate compliance and security. Compliance support is available for CFR Part 11 validated data release workflows, ensuring regulatory adherence throughout the data lifecycle.* |
| **Interoperability and Extensibility** | ***Standards Support:*** *BC Platforms supports major interoperability standards such as HL7, FHIR, OMOP CDM, and DICOM. This ensures seamless data exchange between clinical, research, and lab systems.*  ***Modular and Extensible Architecture:*** *The platform’s microservice-based architecture allows for easy addition of new data modules and tools, ensuring the system adapts to evolving research and clinical needs. API Integration: A rich set of APIs enables integration with external applications, custom workflows, and in-house analytical tools, making it ideal for complex multi-system environments.* |
| ***Data Quality and Integrity*** | ***Data Validation and Provenance:*** *The platform includes mechanisms for data validation, integrity checks, and quality control. Provenance tracking allows users to trace data origin, transformations, and usage, ensuring the trustworthiness and reproducibility of data.*  ***Audit Trails and Versioning:*** *Comprehensive audit trails and versioning capabilities enable tracking of all data changes, access events, and system activities, supporting transparency and compliance. Data governance features ensure that data accuracy and integrity are maintained throughout its lifecycle.* |
| ***Usability and Accessibility*** | ***User Interface:*** *BC Platforms offers an intuitive and user-friendly interface that caters to both technical and non-technical users. Researchers can interact with the platform through drag-and-drop tools, pre-configured analysis templates, and visualization options.*  ***Advanced SQL and Script-Based Queries:*** *For experienced users, advanced SQL query tools and customizable scripts are available. Accessibility and Training: Comprehensive documentation and training resources support users of all skill levels, ensuring effective use of the platform’s capabilities.* |
| ***Scalability and Performance*** | ***Horizontal and Vertical Scalability:*** *The platform is designed to scale both horizontally and vertically based on data volume and analysis complexity, making it suitable for large-scale multiomic and clinical datasets.*  ***Performance Optimization:*** *The platform includes built-in performance monitoring and optimization capabilities, ensuring high performance and responsiveness even under heavy data loads. Leveraging cloud-native technologies, it can scale dynamically to meet growing demands and ensure optimal resource utilization.* |
| ***Collaboration and Sharing Capabilities*** | **Federated and Centralized Research Models:** The platform supports both centralized data storage and federated data access models, enabling collaborative research across multiple institutions while preserving data privacy and compliance.  **Collaboration Tools:** Shared workspaces, project-specific data access, and real-time collaboration tools facilitate team-based research. Fine-grained permissions allow control over data access and sharing.  **Data Sovereignty Compliance:** Federated capabilities enable data to remain within its original location while still allowing research teams to perform cross-site analysis and discovery. |
| ***Cost and Sustainability*** | ***Flexible Deployment Options:*** *BC Platforms offers multiple deployment options—cloud, on-premises, and hybrid—allowing organizations to select a model that best fits their budget and infrastructure.*  ***Cost-Effective Resource Utilization:*** *The platform’s modular architecture and optimized storage options reduce overall operational costs. Regular updates and enhancements ensure long-term sustainability, while customers have the flexibility to scale their usage based on project requirements without additional overhead. Sustainability is supported through continuous R&D investment and close collaboration with industry leaders.* |
| ***Ethical Considerations*** | *Mechanisms for tracking patient consent and ensuring compliance with ethical standards for data use and sharing. Integrated consent management tools enable automated control of data access based on consent rules.*  ***Ethical Review and Governance:*** *Built-in tools support compliance with ethical review processes, ensuring data is accessed and shared in line with ethical requirements. These features help organizations manage sensitive data responsibly, reducing the risk of non-compliance and supporting ethical research practices.* |
| ***Innovation and Adaptability*** | ***Support for Emerging Data Types:*** *BC Platforms continuously adapts to support new and emerging data types, such as multiomic data and imaging data, ensuring the platform remains relevant as research evolves.*  ***Future-Proof Design:*** *The platform’s modular and flexible architecture allows for rapid integration of new technologies, making it easy to incorporate innovations such as AI and machine learning. It also enables research institutions to adapt quickly to changes in regulatory requirements, ensuring compliance and alignment with industry best practices.* |

***References***

*1 your references for relevant literature go here*

***Matrix INSIGHT common challenges***

| **Category** | **Description** |
| --- | --- |
| **Federated Queries Challenges** | **Challenge:** Ensuring consistent data alignment and terminology when conducting federated queries across multiple nodes.  **BC Platforms Solution:** BC Platforms supports standardized ontologies (e.g., OMOP CDM) and provides tools for data harmonization, ensuring consistent federated queries across diverse sites while maintaining compliance. |
| **Patient Privacy and Data Protection** | **Challenge:** Managing data privacy and ensuring compliance across multiple jurisdictions and cross-border research environments.  **BC Platforms Solution:** BC Platforms adheres to privacy-by-design principles, implementing data anonymization and pseudonymization. The platform also supports federated research models, enabling data access without transferring sensitive patient data, ensuring compliance with frameworks like GDPR and HIPAA. |
| **Organizational Policies** | **Challenge:** Adapting to complex organizational policies and diverse access requirements can be difficult in a standardized solution.  **BC Platforms Solution:** BC Platforms uses configurable role-based access controls and policy management to adapt to the unique requirements of each organization, supporting flexible and compliant research environments. |
| **Data Transformation requirements** | **Challenge:** Extensive local knowledge is needed to transform and prepare data for centralized repositories, making ingestion challenging.  **BC Platforms Solution:** BC Platforms provides advanced data ingestion and transformation tools with automated mapping and validation capabilities, simplifying the ingestion of diverse data types such as multi-omics and clinical data. |
| **Installation and Maintenance** | **Challenge:** Complex installation and ongoing maintenance processes hinder deployment scalability.  **BC Platforms Solution:** BC Platforms’ containerized architecture and Kubernetes orchestration simplify deployment and maintenance across cloud, hybrid, and on-premises environments, supporting auto-scaling and reducing downtime. |
| **Secure Deployment** | **Challenge:** Securing deployment environments can be complex, particularly for hybrid and cloud-based infrastructures.  **BC Platforms Solution:** BC Platforms follows industry best practices for cloud-native deployments, including support for encryption, network segmentation, and automated monitoring, ensuring a secure deployment across diverse infrastructures. |
| **Understanding User Queries** | **Challenge:** Supporting diverse user skill levels in querying and exploring data can be challenging.  **BC Platforms Solution:** BC Platforms offers a multi-layered query system with tools like the Cohort Builder for non-technical users and advanced SQL editors for technical users, making it accessible for varying expertise levels. |
| **Informatics and User Experience** | **Challenge:** Complex proprietary platforms may have steep learning curves and limited usability for researchers with varying technical skills.  **BC Platforms Solution:** BC Platforms offers persona-based user interfaces that cater to different user groups, from non-technical researchers to data experts. The platform includes intuitive dashboards, drag-and-drop cohort building, and advanced query capabilities, enhancing usability and enabling efficient data exploration and analysis. |
| **Complexity of INSIGHT Software** | **Challenge:** Complex software implementations for a broad range of operations can lead to difficulties in testing and deploying new configurations.  **BC Platforms Solution:** BC Platforms’ modular microservices-based architecture supports easy customization and adaptation to specific use cases, reducing complexity in testing and configuration. |
| **Incremental Updating Limitations** | **Challenge:** Incremental updates can lead to database fragmentation and require significant maintenance efforts.  **BC Platforms Solution:** Incremental data upload is supported, also including WGS level NGS data. SQL database performs house-keeping operations automatically. |
| **Standardized Vocabularies and Flexibility** | **Challenge:** Limited flexibility in adopting diverse vocabularies for specialized research contexts.  **BC Platforms Solution:** BC Platforms supports multiple vocabularies and ontologies, including OMOP CDM, HPO, and custom structures, enabling flexibility in adapting to specialized research contexts and ensuring comprehensive data representation. |

***References :***

1. please add references here or directly in the fields of the matrix

***Data Modalities Supported by INSIGHT***

INSIGHT supports for different options for Data Modalities: 1) pre-installed Data Modality modules e.g. for OMOP CDM, 2) eCRF editor for creating any structure, including numeric, text, date, multiple choice and file (BLOB) fields and 3) command line APIs for creating any set of datasets (SQL tables) and populating them. Using option 3) we have implemented workflow to automatically ingest new version of UK Biobank based on Data Dictionary and original data file. Using option 2) we have implemented database of FASTQ files (data type BLOB), including all meta-data including sampleID and date.

| **Category** | **Data Modality** | **Description** |
| --- | --- | --- |
| **Clinical Data** | Electronic Health Records (EHRs) | OMOP CDM pre-installable module |
|  | Hospital Administrative Data | Using eCRF editor or API |
| **Genomic Data** | Genomic Sequences | FASTQ, BAM, gVCF, VCF. (g)VCF genotypes, VCF INFO and VCF FORMAT fields are stored to BC Tiling structure (5k subjects * 5 Mbp tiles) facilitating parallelized data analysis. Tiles are compressed BLOBs to optimize data storage costs. |
|  | Genotype Data | Microsatellites, SNPs, imputed data with dosages, copy number variations (CNVs). |
|  | Gene Expression Data | Any data which can be presented as VCF format. |
| **Annotation databases** | Public databases | Common annotation databases such as GnomAD, ClinVar are pre-installed. |
|  | Private databases | System supports installing any private or commercial annotation databases |
|  | Updating option | Service for keeping list of annotation databases up-to-date is offered as an additional service |
| **Imaging Data** | Radiology Images | File repository. DICOM viewers supported / integrated. |
|  | Pathology Images | File repository. DICOM viewers supported / integrated. |
| **Phenotypic Data** | Disease Phenotypes | Using eCRF editor or API |
|  | Clinical Outcomes | Using eCRF editor or API |
| **Medication Data** | Prescription Records | Using eCRF editor or API |
|  | Medication Adherence / Compliance | Using eCRF editor or API |
| **Laboratory Data** | Lab Test Results | Using eCRF editor or API |
| **Survey Data** | Questionnaires and Surveys | Using eCRF editor or API |
|  | Patient-Reported Outcomes | Using eCRF editor or API |
| **Biomarker Data** | Proteomics | Using eCRF editor or API or stored as VCF files |
|  | Metabolomics | Using eCRF editor or API or stored as VCF files |
| **Environmental Data** | Lifestyle Factors | Using eCRF editor or API |
|  | Environmental Exposures | Using eCRF editor or API |
| **Socioeconomic Data** | Social Determinants of Health | Using eCRF editor or API |
| **Family History Data** | Genetic Risk Factors | Pedigrees in LINKAGE format. Visualization using MADELINE |
| **Longitudinal Data** | Time-Series Data | Using eCRF editor or API (primary key: SubjectID, Timestamp) |
| **Behavioral Data** | Behavioral Assessments | Using eCRF editor or API |
|  | Transcriptomics | Using eCRF editor or API or stored as VCF files |
| **Pathway Data** | Biological Pathways | Using eCRF editor or API |
|  | Interaction Networks | Using eCRF editor or API |

***References :***

1. please provide references to relevant publications / documentation here

**Built-in Workflows and Analysis Tools**

**Workflow**

| **Feature** | **Description** |
| --- | --- |
| Workspace management | Admin users can create workspaces only containing approved data. Also genomic data can be controlled in subject and variant level. Different pseudonymization keys are automatically used for every workspace. Off-line workspaces supported (eg for EHDS SPEs). APIs for direct communication with Data Access Approval system |
| Federated Cohort Discovery | Allows researchers to query multiple distributed data sources for patient and sample availability across organizations without moving data. The federated cohort discovery results are viewable in dedicated workspaces linked to each research study, supporting collaborative exploration while maintaining data privacy and compliance. |
| Workspace Cohort Discovery | Enables users to create patient and sample cohorts within a single local dataset using clinical, omics, and metadata attributes. Integrated workspaces provide a collaborative environment for each research study, allowing users to save cohort definitions, share results, and perform downstream analyses within a secure research workspace. |
| Data Integration and Management | Facilitates integration of diverse data types (clinical, genomic, proteomic, and imaging) into a unified platform. Integrated workspaces support seamless data management for each study, providing tools for custom data structure definition, metadata management, and real-time data capture from external systems such as LIMS and EHRs. |
| Ontology Management | Supports managing and using internal and external ontologies (e.g., ICD-10, MedDRA, OMOP) for categorizing data. Integrated workspaces enable ontology-based exploration and analysis, ensuring consistent annotation and harmonization of research datasets, with shared access for research teams working on the same study. |
| Data Extraction and Transformation | Provides automated pipelines for extracting, transforming, and validating data from various sources into formats compatible with the platform’s data models. Integrated workspaces centralize transformation pipelines, allowing researchers to validate and transform datasets collaboratively and access transformed data within their study workspaces. |
| Security and Privacy Management | Implements advanced security measures such as role-based access control, data anonymization, and pseudonymization. Each research study has a dedicated workspace with secure access controls and privacy settings, ensuring compliance with data privacy regulations like GDPR and HIPAA. |
| Multi-Omic Data Analysis | Offers built-in analysis tools for multi-omics research, including genomic, transcriptomic, and proteomic data. Analysis results are saved and shared within integrated workspaces for each study, enabling collaborative review and further analysis, while maintaining study-specific access controls. |
| Custom Workflow and Analysis Pipeline Creation | Supports the creation and management of custom workflows and analysis pipelines using languages like R and Python. Integrated workspaces enable researchers to design, implement, and productize new workflows in containerized environments specific to each research study, ensuring reproducibility and scalability. |
| Data Release and Versioning | Manages controlled data release processes, ensuring data integrity and traceability. Integrated workspaces allow researchers to apply version control and manage data releases specific to each study, maintaining transparency and history of data updates and disclosures for compliance and collaboration. |

**References:**

1. references go here

**Analysis Tools**

| Query Interface | Offers a graphical interface for building complex SQL queries, allowing users to create, modify, and run SQL queries or build queries visually with drag-and-drop components for more flexible data exploration. |
| --- | --- |
| Timeline Viewer | Displays comprehensive subject-specific data view, enabling users to visualize and analyze data over time for individual subjects, making it ideal for longitudinal studies and patient journey reviews. |
| Statistics and Analytics | Provides basic statistical analysis (counts, distributions) through a graphical interface, while advanced statistical modeling is supported through integrated Jupyter notebooks and Nextflow workflows. |
| Plugin Framework | Enables integration of custom tools using Docker containers. Pre-built integrations with Jupyter and Nextflow allow users to develop and run custom workflows for a wide range of analysis needs. |
| Natural Language Processing (NLP) | Supports third-party integration with AI-based NLP tools for extracting structured information from unstructured text, enabling semantic search and advanced text analysis for clinical notes and research documents. |
| Genomic Data Analysis | Unique BC Tiling structure facilitates cost-effective data storage as Objects, and high-performance distributed analysis of >100K whole genomes (WGS). Genomic data can be accessed as VCF, PLINK binary, PLINK text or R dataframe formats. Commonly used workflows such as GWAS and PheWAS are pre-installed. Commonly used bioinformatics tools pre-installed (and accessible from Nextflow scripts), research can bring own tools. |
| Temporal Querying | Facilitates temporal queries through APIs, enabling users to analyze and explore time-based patterns and relationships between different data points, such as treatment events or clinical outcomes. |
| Data Visualization | Includes built-in tools for common visualizations like histograms and scatter plots. |
| Export and Reporting | Supports exporting data in various formats like CSV, Excel, and VCF. Automated reporting options allow easy sharing and export of data based on project needs. |

***References***

*1.*references go here

| **Integration with Other Tools** | R / BioConductor and Python Integration | Using Nextflow and Jupyter notebooks. |
| --- | --- | --- |
|  | Integration with Clinical Trial Management Systems (CTMS) | Using API’s or HL7. |
|  | Integration with Electronic Health Records (EHR) | Using HL7 or customer ETL tool. Both reading and writing. |

References:

1. references go here

**Support for Semantic Integration**

1. **Terminologies and Ontologies**: INSIGHT can integrate with standard medical terminologies and ontologies such as OMOP, ICD, Read, ATC and others. This ensures consistent data representation and facilitates interoperability.
2. **Common Data Models (CDMs)**: INSIGHT supports with OMOP CDM enabling data standardization and federated data analysis across institutions. Also, various custom structures as UK Biobank data model can be created and ingested using APIs.
3. **Ontology Management**: INSIGHT supports different ontologies and code mappings across different OMOP CDM supported ontologies.

**References** :

1. References go here
