# Supplementary material for: Selecting medical research data platforms for translational biomedical research: a five-tier overview and requirement-weighted assessment framework
Source: Front Digit Health. 2026 Jun 17;8:1814015. doi: 10.3389/fdgth.2026.1814015 (PMC13319098; doi:10.3389/fdgth.2026.1814015)
Supplement: Supplementary file 7 [file Supplementaryfile7.docx]

***DigiMed Secure Cloud Platform***

***Deployment and Usage****:*

The DigiMed Secure Cloud is a secure and flexible platform built on OpenStack technologies, specifically designed for future Bavarian healthcare systems. It offers a scalable Infrastructure as a Service (IaaS) to the research community in Bavaria, enabling users to perform store and process data securely. The DigiMed Secure Cloud allows our partners to migrate their on-premises infrastructure and data silos to a central, secure, compliant, and audited platform where collaboration is possible.

The cloud ensures comprehensive data protection through confidential computing (end-to-end data encryption). Data at rest is cryptographically secured via the storage system. Data in use is encrypted using Trusted Execution Environments: AMD SEV-SNP processors. Data in transit between nodes is also encrypted through secure transport protocols. Additionally, the cloud supports federated authentication, strengthened by Shibboleth [2] and OpenID connect [3], to ensure seamless and secure access control.

We developed the DigiMed Secure Cloud's technical infrastructure in parallel with a comprehensive legal framework, which was established prior to the technical implementation. This dual approach has enabled us to create a secure and compliant cloud environment that meets both technical and legal requirements for sensitive data and workflows.

***References:***

1. Openstack Osism, available at <https://osism.tech/docs/>
2. Shibboleth, available at <https://www.shibboleth.net/>
3. https://openid.net/

**DigiMed Secure Cloud components**

The secure features of the DigiMed Secure Cloud are powered by both hardware and software components. The hardware incorporates a compute node cluster and a storage located in two separate network. The software stack performs two main functions: resource provision and threat defense.


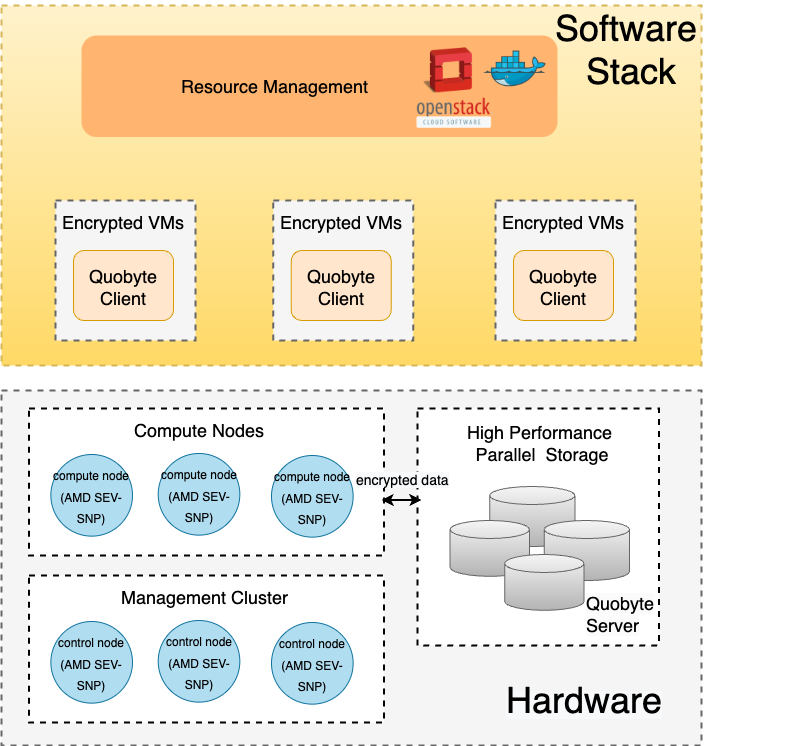


*Fig1. Architecture of DigiMed Secure Cloud.*

***Reference:***

1. N. Zhou, F. Dufour, V. Bode, P. Zinterhof, N. J. Hammer and D. Kranzlmüller, "Towards Confidential Computing: A Secure Cloud Architecture for Big Data Analytics and AI," 2023 IEEE 16th International Conference on Cloud Computing (CLOUD), Chicago, IL, USA, 2023, pp. 293-295, doi: 10.1109/CLOUD60044.2023.00042. 2
2. F. Dufour, N. Zhou, V. Bode, P. Zinterhof, N. J. Hammer, D. Kranzlmüller, “Towards Confidential Computing: A Cloud Architecture for Big Data Analytics and AI in Biomedical Research (poster),” in ISC, Hamburg, Germany, May 2023.
3. N. Zhou, F. Dufour, V. Bode, P. Zinterhof, N. J. Hammer, D. Kranzlmüller, “ DigiMed Cloud: A Highly-Secure Cloud for Big Data Analytics and AI in Biomedical Research (poster),” in Hipeac, Munich, Germany, Jan 2024.

***Matrix DigiMed Secure Cloud features***

| *Criteria* | *Details* |
| --- | --- |
| ***Security and Privacy*** | - Confidential computing: Once in the cloud, data is end-to-end-encrypted using AMD’s Secure Encrypted Virtualization (SEV) feature - Collection of anonymized/pseudo-nymized data only (pseudonymisation concept by [1]) - Storage access granted organization-wise by default. - Authentication and authorization continuously required, e.g., VPN, SSH, OpenStack Keystone, shibboleth and OpenID. |
| ***Compliance and Regulatory Adherence*** | - GDPR/DSGVO - ISO/IEC 27001-Certification (ISMS) (in progress) - C5 Criteria Catalog [2] (in progress) |
| **Interoperability and Extensibility** | - DigiMed current purpose is for secondary use of medical data for research purposes only. We do not integrate EHR data at this moment, only clinical and multi-omic |
| ***Data Quality and Integrity*** | ***Not applicable.*** |
| ***Usability and Accessibility*** | - In addition to the user documentation [3], we provide cloud usage trainings (workflow migration and best practices) as well as awareness and security training. Security and usage training are mandatory for user onboarding. - Initially access is limited to DigiMed community, to date researchers upon approval. |
| ***Scalability and Performance*** | - Example are:   - Clinical knowledge graph [4] (GB).   - German Heart Centre Data Lake (TB).   - Helmholtz sequencing data (PB). - Scaling perspective horizon 2027: 10PB storage, 3PB backups/archives, 5 GPU nodes, 20 CPU nodes - Storage performance is monitored by the storage system, system performances are monitored via checkmk. |
| ***Collaboration and Sharing Capabilities*** | - Users are provided with the ability to share compute and storage resources. Granular control is provided at the organization, project, group, and user level. - cloud is delivered as IaaS. Users deploy workspaces on their own. |
| ***Cost and Sustainability*** | - The DigiMed Cloud services are free for DigiMed project partners - Sustainability depends by German Bavarian State Ministry for Health, Care, and Prevention(Bayerisches Staatsministerium für Gesundheit, Pflege und Prävention)   - current funding ends in 2027 and extended funding is planned |
| ***Ethical Considerations*** | - Consent is managed by scientific partners. This part is not managed by DigiMed Secure Cloud directly. However, a consortium of scientists within WP7 of the DigiMed project has been continuously working on this topic [5]. |
| ***Innovation and Adaptability*** | - Providing the DigiMed Secure Cloud as an IaaS allows to experiment with different components while providing users with a stable experience. - New technologies such as confidential GPUs (GPUs with TEEs to support confidential computing) and Hardware Security Module (HSM) are to be integrated in phase two. - New technologies and stacks are tested on a testbed before being ported to the DigiMed Secure Cloud - New regulatory requirements can be easily adapted due to the delivery model that DigiMed Cloud offers. |

***References***

*1* <https://bitcare.de>

*2.* <https://www.bsi.bund.de/EN/Themen/Unternehmen-und-Organisationen/Informationen-und-Empfehlungen/Empfehlungen-nach-Angriffszielen/Cloud-Computing/Kriterienkatalog-C5/kriterienkatalog-c5_node.html>

*3.https://digimed.pages.gitlab.lrz.de/user-documentation/*

*4.* [*https://github.com/MannLabs/CKG*](https://github.com/MannLabs/CKG)

*5.* https://www.digimed-bayern.de/en/work-packages/wp-7elsi.html

***Matrix DigiMed Secure Cloud common challenges and mitigation***

| **Category** | **Description** |
| --- | --- |
| **Federated Queries Challenges** | Not applicable. |
| **Patient Privacy and Data Protection** | Data is managed on the institute level. Data by default is never shared between different institutes unless otherwise agreed with each other. Data is encrypted by default unless otherwise disabled by users themselves. Institutes are responsible for redacting patient data who revoke their consents. Data anonymization and pseudonymization are performed before patient data is uploaded to the cloud. |
| **Organizational Policies** | Not applicable. |
| **Data Transformation requirements** | Data stay intact on the Cloud where users are responsible for data transformation. |
| **Installation and Maintenance** | Deployment and installation are automatic, which can be easily triggered by Ansible scripts. One challenge lies in introduce of new components to the cloud, which may require significant work in re-scripting and generation of new container images. |
| **Secure Deployment** | Challenges:   - Onboard institutes that are outside the VPN Munich Scientific Network - Keep track for all the inventories of software and hardware, especially user installation. - Lack of blueprint and good practices for secure deployment at the design phase   Mitigation:  Dependency track is performed using *dependencytrack* (https://dependencytrack.org) with the database located locally on the DigiMed secure cloud. |
| **Understanding User Queries** | The DigiMed Secure Cloud is offered as IaaS. DigiMed cloud provisions with flexible environment customization. Users are responsible for installation of the necessary software to enable query function. Strict data separation is enforced otherwise. |
| **Informatics and User Experience** | Tools to perform data analytics are installed by users. A popular demand can be accessed and made available in the VM base image offering to all users or selected users. Users are managed in a hierarchy fashion. Each organization elects a couple of master users that are directly managed by LRZ and the master users control the rest within the scope of their organizations. |
| **Complexity of DigiMed Secure Cloud Software** | Not applicable, DigiMed offers IaaS cloud directly. |
| **Incremental Updating Limitations** | Data is encrypted at all time. The DigiMed Secure Cloud does not alter data form or perform data analysis on user data. Within the scope of DigiMed Secure Cloud security concept, users are directly responsible for de-identification of their data. Exposure of data to public is disabled by default. Users can optionally choose to expose the data of their own affiliation to any third party, however, must be responsible for the risks that may incur. |
| **Standardized Vocabularies and Flexibility** | Basic VM images are offered by DigiMed Cloud. Data on storage is only accessible via Munich scientific network and is not visible to people outside their organizations. Users are complied with the security concepts of DigiMed. Non-compliance to the agreed standards within the collaborative learning may lead to exclusion of a participant. |

- ***does your community organize data challenges / platform challenges? Is there any benchmarking available?***

*Benchmarking will be available via a bachelor thesis titled "Cloud Performance in Healthcare: A Benchmarking and Analysis Study of the DigiMed Bayern Secure Cloud" in March 2025.*

References :

1. F. Dufour, “Towards the Medicine of the Future in Bavaria and Germany, One Heartbeat at the Time With Confidential Computing,” in Open Confidential Computing Conference (OC3), Online, 2023 (conference talk, [link](https://dufour.xyz/x/oc3)).
2. <https://digimed.pages.gitlab.lrz.de/user-documentation/Acceptable_Use_Policy/>
3. V. Pfeil, “Confidential Computing Via Hardware Trusted Execution Environments by an Openstack HPC Capable Cloud,” Bachelor Thesis, University of the Bundeswehr Munich, Munich, Germany, 2024.

***Data Modalities Supported by DIGIMED Secure Cloud***

Usually, clinical research data platforms are designed to integrate and manage a wide range of data modalities to support biomedical research. The primary data modalities used so far in DIGIMED Secure Cloud include:

This is not applicable to our platform. This would need to be addressed by our scientific partners who are actively using the cloud for their research.

| **Category** | **Data Modality** | **Description** |
| --- | --- | --- |
| **Clinical Data** | Electronic Health Records (EHRs) | Structured data (please specify) and unstructured data (please add information on indexing / information extraction possible). |
|  | Hospital Administrative Data | Admissions, discharges, transfers, billing codes, and insurance information. |
| **Genomic Data** | Genomic Sequences | Whole genome, exome sequencing, targeted sequencing (please specify and provide references). |
|  | Genotype Data | Single nucleotide polymorphisms (SNPs), copy number variations (CNVs). Please specify and provide references. |
|  | Gene Expression Data | What types of transcriptomics data? |
| **Imaging Data** | Radiology Images | MRI, CT, X-ray, ultrasound. Please specify and provide references if possible |
|  | Pathology Images | Digital pathology slides, histology images. Please specify and provide references if possible |
| **Phenotypic Data** | Disease Phenotypes | Disease characteristics, symptom severity, progression. Use of HPO or other controlled vocabularies for annotation? Please provide references if possible. |
|  | Clinical Outcomes | Treatment responses, survival rates, recurrence. Please specify and provide references if possible. |
| **Medication Data** | Prescription Records | (Co-)Medication names, dosages, administration routes, duration. Please add references if possible. |
|  | Medication Adherence / Compliance | e.g. Refill records, patient self-reports. |
| **Laboratory Data** | Lab Test Results | Blood tests, urine tests, microbiological cultures, biochemical assays. Biomarker measurements. Please provide references if possible. |
| **Survey Data** | Questionnaires and Surveys | Patient health questionnaires, lifestyle surveys, mental health assessments. Please provide references if possible. |
|  | Patient-Reported Outcomes | Pain scales, quality of life measures, functional status. Please provide references if possible. |
| **Biomarker Data** | Proteomics | Protein expression, protein-protein interactions, post-translational modifications. Mass-Spec, MALDI whatsoever. Blood and urine proteomics? References ?? |
|  | Metabolomics | Metabolite profiles, metabolic pathways, lipidomics. |
| **Environmental Data** | Lifestyle Factors | Diet, physical activity, workout schemata, smoking, alcohol consumption, substance (ab)use. |
|  | Environmental Exposures | Air quality, water quality, exposure to toxins, occupational hazards. |
| **Socioeconomic Data** | Social Determinants of Health | Education, income, employment status, housing, neighborhood characteristics. |
| **Family History Data** | Genetic Risk Factors | Family history of diseases, pedigree analysis. Risk alleles, tumor gene panels etc. |
| **Longitudinal Data** | Time-Series Data | Repeated measures over time, disease progression, treatment responses over time. Please provide information on how you organize information in time (from time stamp to longitudinal representation of patients). |
| **Behavioral Data** | Behavioral Assessments | Cognitive tests, psychological assessments, behavioral interventions. Nutrition coaching? Please specify and provide references, if possible. |
|  | Transcriptomics | mRNA levels, non-coding RNAs, alternative splicing events. |
| **Pathway Data** | Biological Pathways | Signaling pathways, metabolic pathways. Mechanism graphs. Pathophysiology graphs (disease maps) ? |
|  | Interaction Networks | Protein-protein interaction networks, gene regulatory networks. Co-expression networks ? |

***References :***

1. please provide references to relevant publications / documentation here

**Built-in Workflows and Analysis Tools**

Does DigiMed Secure Cloud contain built-in workflows and analysis tools that facilitate clinical and translational research?

Environment customization can be flexibly performed by users.

**Workflow**

| **Feature** | **Description** |
| --- | --- |
| Patient Cohort Discovery | Not applicable. |
| Data Integration and Management | Not applicable. |
| Ontology Management | Not applicable. |
| Data Extraction and Transformation | Not applicable. |
| Security and Privacy Management | Data security and privacy are enforced by tenant separation, data encryption and federated access control. |

**References:**

1. references go here

**Analysis Tools**

Not directly addressed by DigiMed Secure Cloud. This point needs to be otherwise addressed by our scientific partners.

| Query Interface | Main interface for creating queries to identify patient cohorts based on various clinical and demographic criteria. Please specify |
| --- | --- |
| Timeline Viewer | Visualize individual patient timelines, displaying events such as diagnoses, treatments, and lab results over time. Please specify for  DIGIMED Secure Cloud |
| Statistics and Analytics | Basic statistical tools to analyze query results, including counts, distributions, and summary statistics. |
| Plugin Framework | Integrate external analysis tools and custom plugins to extend the platform's capabilities. Please provide references, examples and documentation of plugin architecture. |
| Natural Language Processing (NLP) | Does DIGIMED Secure Cloud comprise already integrated NLP services? Are they open source? |
| Genomic Data Analysis | Integrate and analyze genomic data alongside clinical data, often requiring additional modules or plugins. |
| Temporal Querying | Perform queries that consider the temporal sequence of events, such as identifying patients who had a particular treatment before a specific diagnosis. |
| Data Visualization | Basic tools for visualizing data distributions and query results, extendable with additional plugins for advanced visualization. |
| Export and Reporting | Does DIGIMED Secure Cloud allow for export of query results for further analysis or reporting purposes in formats compatible with other statistical and data analysis software. |

***References***

*1.*references go here

| **Integration with Other Tools** | R / BioConductor and Python Integration | Use R and Python scripts for advanced statistical analysis and machine learning workflows. Please specify and provide references … |
| --- | --- | --- |
|  | Integration with Clinical Trial Management Systems (CTMS) | Is DIGIMED Secure Cloud integrated with CTMS for managing clinical trial data and workflows?. |
|  | Integration with Electronic Health Records (EHR) | Does DIGIMED Secure Cloud allow for seamless integration with EHR systems to pull in clinical data for analysis? |

References:

1. references go here

**Support for Semantic Integration**

Not applicable. The DigiMed Secure Cloud is offered as IaaS.

Does DIGIMED Secure Cloud support semantic integration through the use of terminologies, ontologies, and common data models? Such as:

1. **Terminologies and Ontologies**: Can DIGIMED Secure Cloud integrate with standard medical terminologies and ontologies such as ICD, SNOMED CT, LOINC, and others. This ensures consistent data representation and facilitates interoperability.?
2. **Common Data Models (CDMs)**: Can DIGIMED Secure Cloud work with various common data models like the Observational Medical Outcomes Partnership (OMOP) CDM, enabling data standardization and easier data sharing across institutions.?
3. **Ontology Management**: Does the platform include tools for ontology management, allowing users to customize and extend the ontologies as needed to fit their specific research requirements​?

**References** :

1. References go here
