# Supplementary material for: Selecting medical research data platforms for translational biomedical research: a five-tier overview and requirement-weighted assessment framework
Source: Front Digit Health. 2026 Jun 17;8:1814015. doi: 10.3389/fdgth.2026.1814015 (PMC13319098; doi:10.3389/fdgth.2026.1814015)
Supplement: Supplementary file 8 [file Supplementaryfile8.docx]

**Overview**

DNAnexus is a cloud-based platform designed for managing, analyzing, and sharing genomic and biomedical data. DNAnexus platform is independently certified as compliant to relevant research and clinical regulations (including ISO 27001, HIPAA, CLIA, CAP and GCP).  It provides a comprehensive environment for data-driven research in genomics, facilitating collaborations, and ensuring data security and compliance.( 18,[9](file:///D:/Martin%27s%20Pojectwork/platforms/9), 17 )

**Matrix table for the** **DNAnexus platform’ features**

| **Dimension** | **Description** |
| --- | --- |
| **Security and Privacy(1,2,19)** | provides strong cybersecurity - Data encryption at rest and in transit - Regular security audits and vulnerability assessments - Compliance with industry standards (e.g., HIPAA, GDPR) |
| **Compliance and Regulatory Adherence(2,3)** | - Support for regulatory standards such as HIPAA, GDPR, CLIA, and GxP - Audit trails and compliance reporting - Data residency and sovereignty options |
| **Interoperability and Extensibility(4,5,6,7)** | - APIs and SDKs for integration with external systems - Support for standard data formats (e.g., FASTQ, BAM, VCF) - Custom tool and pipeline integration using Docker |
| **Data Quality and Integrity(8,9,20)** | - Quality control tools for data validation - Detailed logging of data processing steps, easy integration and customization efficient data analysis |
| **Usability and Accessibility(10,9,20)** | User-friendly-Intuitive web interface- Comprehensive documentation and tutorials - Accessibility features to support diverse user needs |
| **Scalability and Performance(11,12,13,18)** | - Elastic scaling to handle varying workloads - High-performance computing resources - Efficient resource allocation and management |
| **Collaboration and Sharing Capabilities(12,13,14,17,18)** | - Secure data sharing with fine-grained access controls - Collaborative project workspaces - Tools for real-time collaboration and communication- Cloud-based big data solutions can facilitate collaboration between teams and organizations, enabling them to work together more efficiently. |
| **Cost (12)** | Pay-as-you-go pricing model - cost-efficient |
| **Ethical Considerations(15)** | Policies to ensure ethical data use - Support for patient consent management |
| **Innovation and Adaptability(16)** | - Regular updates with new features and tools - Support for cutting-edge research methodologies - Flexible platform to adapt to evolving research needs |

***References***

1. [***https://link.springer.com/article/10.1186/1471-2105-15-30***](https://link.springer.com/article/10.1186/1471-2105-15-30)
2. [***https://documentation.dnanexus.com/faqs/legal-and-compliance***](https://documentation.dnanexus.com/faqs/legal-and-compliance)
3. [***https://documentation.dnanexus.com/admin/audit-trail***](https://documentation.dnanexus.com/admin/audit-trail)
4. [***https://documentation.dnanexus.com/***](https://documentation.dnanexus.com/)
5. [***https://documentation.dnanexus.com/developer/api***](https://documentation.dnanexus.com/developer/api)
6. [***https://documentation.dnanexus.com/user/running-apps-and-workflows/tools-list***](https://documentation.dnanexus.com/user/running-apps-and-workflows/tools-list)
7. [***https://dnanexus.gitbook.io/uk-biobank-rap/working-on-the-research-analysis-platform/bringing-your-app-or-workflow-to-the-research-analysis-platform***](https://dnanexus.gitbook.io/uk-biobank-rap/working-on-the-research-analysis-platform/bringing-your-app-or-workflow-to-the-research-analysis-platform)
8. [***https://documentation.dnanexus.com/admin/gxp***](https://documentation.dnanexus.com/admin/gxp)
9. ***https://www.dnanexus.com/***
10. [***https://bioinformatics.ccr.cancer.gov/gau/dnanexus-biowulf-setup/***](https://bioinformatics.ccr.cancer.gov/gau/dnanexus-biowulf-setup/)
11. [***https://link.springer.com/article/10.1186/1471-2105-15-30***](https://link.springer.com/article/10.1186/1471-2105-15-30)
12. [***https://journals.plos.org/plosone/article?id=10.1371/journal.pone.0129277***](https://journals.plos.org/plosone/article?id=10.1371/journal.pone.0129277)
13. [***https://aws.amazon.com/solutions/case-studies/dnanexus/***](https://aws.amazon.com/solutions/case-studies/dnanexus/)
14. [***https://www.dnanexus.com/platform-collaborate***](https://www.dnanexus.com/platform-collaborate)
15. [***https://www.dnanexus.com/terms/acceptable-use-policy***](https://www.dnanexus.com/terms/acceptable-use-policy)
16. [***https://documentation.dnanexus.com/release-notes***](https://documentation.dnanexus.com/release-notes)
17. [***https://aacrjournals.org/cancerdiscovery/article/11/5/1082/666431/St-Jude-Cloud-A-Pediatric-Cancer-Genomic-Data***](https://aacrjournals.org/cancerdiscovery/article/11/5/1082/666431/St-Jude-Cloud-A-Pediatric-Cancer-Genomic-Data)
18. [***https://www.biorxiv.org/content/10.1101/343970V1.full***](https://www.biorxiv.org/content/10.1101/343970V1.full)
19. [***https://pubs.acs.org/doi/full/10.1021/acs.jcim.2c00255***](https://pubs.acs.org/doi/full/10.1021/acs.jcim.2c00255)
20. https://www.sciencedirect.com/science/article/pii/S2772442523000576

**DNAnexus Architecture (1)**

***1.https://www.dnanexus.com/platform-collaborate***


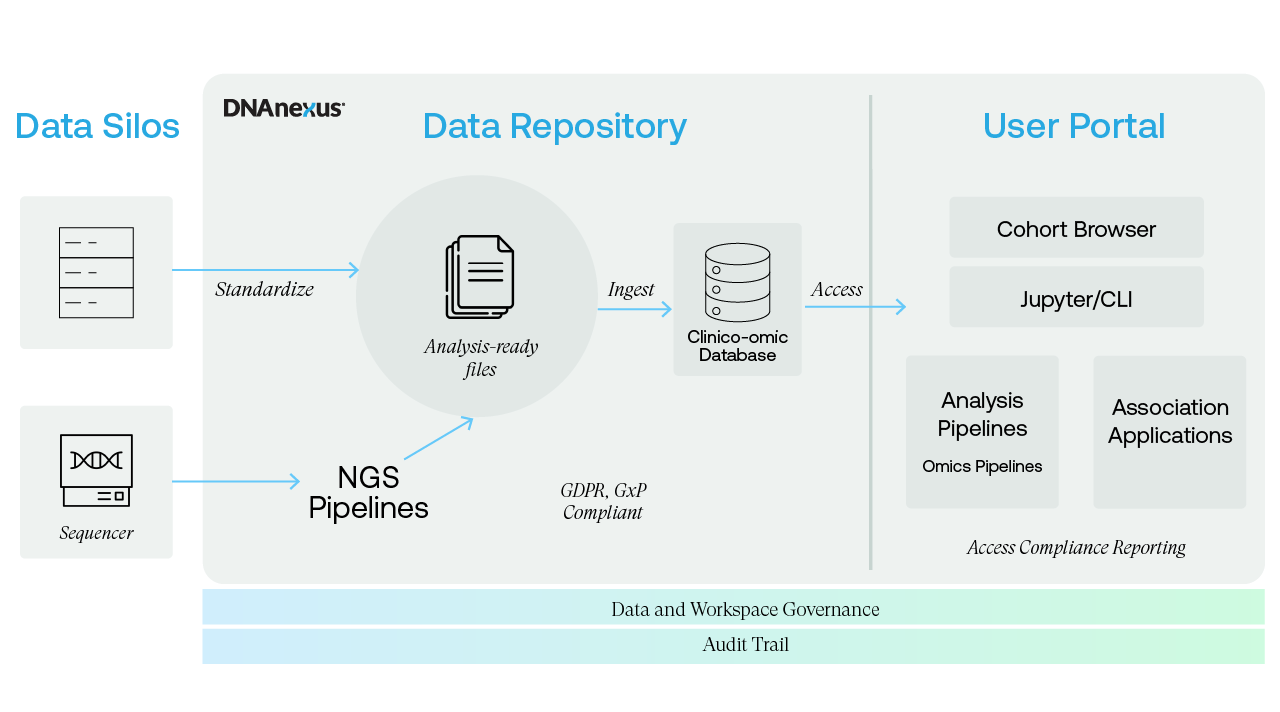


**DNAnexus Common Challenges**

| **Challenge** | **Description** | **Example/Context** |
| --- | --- | --- |
| **On-Demand Instances Availability(1)** | On-demand instances provide uninterrupted service but may not always be available. | Users may experience interruptions in their workflow if instances are not available when needed. |
| **Security Issues(2)** | Many cloud servers are insufficiently secured because security experts are not involved in their design. Virtualization introduces new security challenges, as existing security systems do not work properly with virtual network devices. | The lack of physical network devices makes it harder to apply traditional security measures effectively. |
| **Reduced Control(3)** | Reduced control over the distribution of computation and resources. | Users have limited control over how resources are allocated and managed in the cloud environment. |
| **Data Transfer Time(3)** | Large amounts of time needed to transfer large data to and from the cloud. | Transferring genomic data sets to the cloud can be time-consuming, affecting the overall efficiency of the analysis process. |
| **Data Transfer Problems(3)** | Easy access to resources but data transfer problems remain. | Despite easy access to computational resources, transferring large data sets can still be problematic due to bandwidth limitations. |
| **Network Bandwidth Issues(3)** | Problems with network bandwidth make the transfer of large data difficult. | Limited bandwidth can slow down the transfer of large genomic data sets, causing delays in processing and analysis. |
| **Data Integration(4)** | Integrating and managing data from various sources can be challenging and time-consuming. | A healthcare organization must ensure that patient data from multiple sources, such as EHR, lab reports, and wearable devices, is integrated and managed effectively. |
| **Skill Shortage(4)** | There is a shortage of skilled professionals who can design, implement, and maintain cloud-based big data solutions. | A healthcare organization may need to hire outside experts or train existing staff to develop and maintain a cloud-based big data solution. |
| **Latency Issues(4)** | Cloud-based big data solutions may experience latency issues due to data processing and transmission over the internet. | Ensuring that patient data is processed and analyzed in a timely manner can be challenging due to latency in cloud-based systems. |
| **Data Portability(4)** | Moving data between different cloud providers or on-premises systems can be challenging due to differences in data formats and structures. | A healthcare organization must ensure that patient data stored in the cloud can be easily migrated between different cloud providers or on-premises systems. |

**References**

1. <https://journals.plos.org/plosone/article?id=10.1371/journal.pone.0129277>
2. <https://link.springer.com/article/10.1007/s13258-015-0280-7>
3. <https://citeseerx.ist.psu.edu/document?repid=rep1&type=pdf&doi=df267c7084b0da0dd418661dc0130a1e49c0b3c0>

1. <https://www.sciencedirect.com/science/article/pii/S2772442523000576>

**DNAnexus data modality**

| **Data Modality** | **Description** | **Examples** |
| --- | --- | --- |
| **Genomic Data(1)** | Analysis of DNA sequences. | Whole Genome Sequencing (WGS), Whole Exome Sequencing (WES), Targeted Sequencing |
| **Transcriptomic Data(2)** | Analysis of RNA sequences to study gene expression. | RNA Sequencing (RNA-Seq), Single-cell RNA-Seq (scRNA-Seq) |
| **Epigenomic Data(3­)** | Study of chemical modifications on DNA and histone proteins. | ChIP-Seq, ATAC-Seq, DNA Methylation Sequencing |
| **Proteomic Data(4)** | Large-scale study of proteins, their structures, and functions. | Mass Spectrometry (MS), Protein Arrays |
| **Metabolomic Data(4)** | Comprehensive analysis of metabolites in a biological sample. | NMR Spectroscopy, Mass Spectrometry-based Metabolomics |
| **Clinical Data(5)** | Data related to patient health records and clinical trials. | Electronic Health Records (EHR), Clinical Trial Data |
| **Phenotypic Data(4)** | Observable traits and characteristics of organisms. | Imaging Data (MRI, CT scans), Wearable Device Data |

**Reference**

1. <https://citeseerx.ist.psu.edu/document?repid=rep1&type=pdf&doi=df267c7084b0da0dd418661dc0130a1e49c0b3c0>
2. <https://www.sciencedirect.com/science/article/pii/S0006497119708258>
3. <https://www.nature.com/articles/ng.3968>
4. <https://www.dnanexus.com/press/dnanexus-streamlines-access-analysis-for-large-scale-proteomics-data>
5. <https://www.sciencedirect.com/science/article/pii/S215335392200757X>

**DNAnexus Common Tools**

| **Category** | **Workflow/Tool Name** | **Description** | **Use Case** |
| --- | --- | --- | --- |
| **Sequencing** | **BWA** | Burrows-Wheeler Aligner for mapping low-divergent sequences against a large reference genome | Sequence alignment |
|  | **FASTQ-to-BAM** | Converts raw FASTQ files to aligned BAM files | Data preprocessing |
| **Variant Calling** | **GATK** | Genome Analysis Toolkit for variant discovery in high-throughput sequencing data | Variant calling, germline/somatic variants |
|  | **DeepVariant** | A deep learning-based variant caller developed by Google | High-accuracy variant calling |
| **RNA-Seq** | **STAR** | Spliced Transcripts Alignment to a Reference for RNA sequencing reads | RNA-Seq alignment |
|  | **Kallisto** | Quantifies abundances of transcripts from RNA-Seq data | RNA-Seq quantification |
| **Single-cell** | **Cell Ranger** | Analyzes single-cell RNA sequencing data | Single-cell RNA-Seq analysis |

**References**

1. https://documentation.dnanexus.com/user/running-apps-and-workflows/tools-list

***Note: for further tools*** : <https://documentation.dnanexus.com/user/running-apps-and-workflows/tools-list>

**support for semantic integration**

| **feature** | **Description** |
| --- | --- |
| **Terminologies** | *There is no evidence on the Worldwide Web,* only this address from *the platform's webpage*  ´´*https://docs.omics.ai/products/workbench/terminology´´* users can fetch standardized terms from ***external terminology*** servers and apply them to their data within DNAnexus(**ChatGPT**) |
| **Ontologies** | DNAnexus does not provide built-in ontologies, it can integrate with external ontologies and controlled vocabularies. Users can reference these ***external sources*** to annotate and organize their data semantically. (**ChatGPT**) and *it seems that there not any support for that according to this statement from DNAnexus platform.(2)* |
| **Common Data Models (CDMs)(3)** | The platform allows for the annotation of data with metadata, which can include semantic information about the data. This metadata can be used to integrate data from different sources based on shared attributes |
| **Metadata Management** | *there isn't a specific academic reference solely for DNAnexus regarding metadata annotation with standardized vocabularies, but ChatGPT says* |
| **Data Harmonization(2)** | *it seems that there not any support for that according to this statement from DNAnexus platform.(2)* |

**References :**

1. <https://documentation.dnanexus.com/user/spark/vcf-preprocessing>
2. <https://documentation.dnanexus.com/developer/ingesting-data>
3. https://documentation.dnanexus.com/developer/workflows/workflow-metadata
