# Supplementary material for: Selecting medical research data platforms for translational biomedical research: a five-tier overview and requirement-weighted assessment framework
Source: Front Digit Health. 2026 Jun 17;8:1814015. doi: 10.3389/fdgth.2026.1814015 (PMC13319098; doi:10.3389/fdgth.2026.1814015)
Supplement: Supplementary file 9 [file Supplementaryfile9.docx]

*Here comes the “empty” form to add your information. All my comments are labeled in blue. They can be removed. Would be good if you chose another color for your input.*

***HiGHmed HIVE Suite***

***Deployment and Usage****:*

Rooted in the NUM (network university medicine) funding for medical informatics in Germany, during the covid-19 pandemic, a core set of key components related to a central openEHR-based clinical data respository (CDR) and connecting infrastructure to the MII (medical informatics initiative) have been introduced to the community. Specifically, an openEHR-compatible instance “EHRbase” was co-authored by the Medizinische Hochschule Hannover, Vitagroup and HiGHmed.
Now addressing the specific needs of long-term clinical data integration centers, short-term project data stores and secondary use (specifically AI training), HiGHmed is professionalizing and extending the existing tools towards a cloud-based, industry-grade service.

***References:***

https://openehr.org/static/files/resources/openEHR_vendor_independent_platform.pdf

<https://www.gesundheitsforschung-bmbf.de/de/codex-bundelt-daten-fur-die-covid-19-forschung-12743.php>

*https://ebooks.iospress.nl/doi/10.3233/SHTI240808*

**HIVE suite product components**


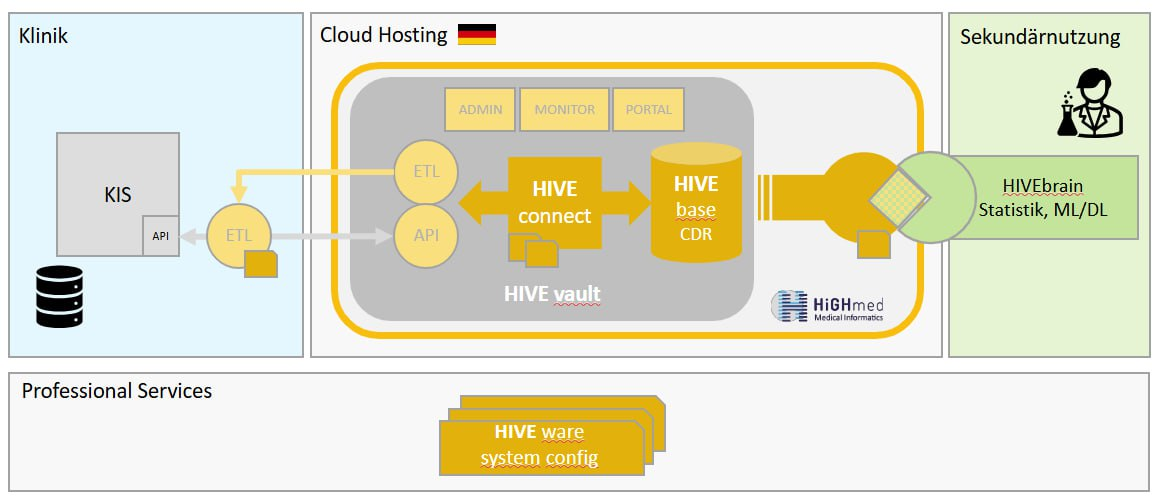


- **HIVEbase: openEHR-compatible EHRbase instance**
- **HIVEconnect: no-code configurable FHIR/openEHR mapper**
- **HIVE vault: contains HIVEbase + connect + misc. facilitating components that make up a complete cloud-based data integration center service.**
- **HIVEbrain: privacy-perserving secure compute environment**
- **HIVEware: a collection of standard system configurations (KIS-ETL, mappings, etc)**

***Reference:***

n/a in Arbeit …

***Matrix “HIVE Suite” features***

| *Criteria* | *Details* |
| --- | --- |
| ***Security and Privacy*** | *Deployment of organization-specific mandant instances, keycloak for ID management and authentication. FHIR/openEHR endpoints all SSL encrypted + certificates.* |
| ***Compliance and Regulatory Adherence*** | *GDPR, BSI IT-Grundschutz* |
| **Interoperability and Extensibility** | ***Standards Support****: FHIR MII-Kerndatensatz compatibility, openEHR templates according to publicly accessible resources i.e. ckm.openehr.org, output channels as FHIR, openEHR and OMOP.*  ***Extensibility****: KIS integration planned (step-wise) starting with Dedalus ORBIS, ETL-framework built-in to facilitate customer-side development + extensions* |
| ***Data Quality and Integrity*** | ***Data Validation****: native feature in openEHR, thanks to the strong semantic typing of medical records. Validation is performed on all data types according to criteria specified in the object primitives or archetypes (i.e. range, date, units of measurements, patterns etc)* ***Audit Trails****: Yes, in/output of all HIVESuite components is logged, indexed and made available via a GRAFANA dashboard.*  ***Data Provenance****: currently only KIS-type information. We will be working next on highly resolved timeseries data from for example PDMS system. PACS is not directly included, but images can be referenced. Genomic data is included in openEHR models, but we are currently not putting a focus on it.* |
| ***Usability and Accessibility*** | ***User Interface****:* ***Training and Support:****.*  *Extensive openEHR training available in the openehr.org community. Specific to our system, we will plan for: i) video training, ii) support-center (chat/call)*  ***Accessibility: n/a*** |
| ***Scalability and Performance*** | ***Handling Large Datasets:*** *not yet, we are working on “scaling” – so far non of the KIS-based datasets is real big-date. This may change however with genomics.*  ***Performance Metrics: Yes, part of the monitoring and accessible in GRAFANA dashboard*** |
| ***Collaboration and Sharing Capabilities*** | Yes, we allow inter-clinics communication between i) 1:1 openEHR (HIVEbase) instances, or 1:N (hub-spoke model), ii) connection to any MII/NUM-compatible data integration center via DSF (data sharing framework) adapters, iii) in perspective: represent an EHDS/FHIR-compatible data node in an EU-collaborative setting |
| ***Cost and Sustainability*** | ***Cost-Effectiveness:*** *yes, but cannot disclose directly.*  ***Sustainability:*** *openEHR increasingly popular in Europe (UK, Spain, NL, Denmark, Finland …), in Germany so far only by a minority of clinics (Charité, Hannover, Vivantes Kliniken, etc), growing ☺* |
| ***Ethical Considerations*** | ***Informed Consent:*** *consent can be annotated as object in openEHR. Among the care-facing (web)applications we are planning for, a consent management form facing patients is on the list (but not yet implemented)*  ***Ethical Review:*** *n/a* |
| ***Innovation and Adaptability*** | ***New Technologies:*** *well, openEHR for data persistence is “new” to many still sticking to the FHIR world for both data transfer and persistence. A co-existance is ideal and actually wanted by HL7/openEHR joining forces with FHIR putting focus on transfer and openEHR on EHR-level persistance*  ***Flexibility:*** *as a central “adjustment screw”, openEHR allows to incorporate new data models and capitalize on a huge community of internationally-reviewed models which are localization-independent but can be localized (labelling) very easily with native openEHR capabilities. Second, cloud deployment allows us to easily scale-up storage/bandwidth as needed and adapt to needs (university clinics vs. pop-up “temporary” project strore)* |

***References***

*n/a*

***Matrix “HIVE Suite” common challenges***

| **Category** | **Description** |
| --- | --- |
| **Federated Queries Challenges** | HIVEbase (or HIVEvault) are local, centralized data repository with internal ID-space for patients. Most of the applications engineered on top of the CDR reside with the clinics, thus same ID space. If connectivity features (for example MII/NUM via DSF) are to be used, records can be pseudonymized (and receive a project-namespace pseudonym) using fTTP (federated trusted third party). Currently Uni Greifswald is providing such fTTP, but we intend to offer one on our own. |
| **Patient Privacy and Data Protection** | Single-organization mandant instances running on high-end CSP in Germany |
| **Organizational Policies** | n/a. with regard to authorization we have role-based auth with users + groups. |
| **Data Transformation requirements** | Yes, definitively: Input from KIS systems. Orchestration via Apache Airflow. Output FHIR/openEHR/OMOP via ETL adapters. |
| **Installation and Maintenance** | HIVE suite is cloud based, containerized (K8) setup, that we manage and can replicate at short notice. Images containing ALL components are integration-tested, thus including concurrent, validated software updates of its components |
| **Secure Deployment** | Secured within realm of CSP. In/output connections all TLS SSL, certificate protected. Just IT-standard nowadays. |
| **Understanding User Queries** | Two types: i) research queries (anything that can be described using openEHR AQL language – similar to SQL), ii) (web)applications built on top of HIVE which are designed to execute one (or few) domain-specific queries, transform data into view and depict the view to the user side. This is more an easy-access querying in the clinical routine. |
| **Informatics and User Experience** | See previous answer. (i) open exposure for trained medical/IT staff, (ii) anyone can do. |
| **Complexity of < YOUR PLATFORM > Software** | See previous box, last topic “flexibility”. On the IT-side the system is actually very complex (many of the hospitals trying to do this on their own with limited staff, do fail miserably) but it is assembled using state-of-the-art development processes and deployment tools (CI/CD pipeline). So we are able to manage complexity easily. |
| **Incremental Updating Limitations** | In contrast to FHIR specs (which are quick to implement, but contain a residual “fuzziness” that can kill your dataspace with model evolution) the openEHR world is very well thought, mature and robust (backward compatible) with regard to model updates. We have seen A LOT of FHIR-only apps dying from model erosion over time (and lack of resources to keep pace with adapting business logic) while this is almost no problem with openEHR |
| **Standardized Vocabularies and Flexibility** | Terminology service being used in ETL processes for validation purpose. Using standards like SNOMED, ICD and others. |

- ***does your community organize data challenges / platform challenges? Is there any benchmarking available?***
- ***This has been the case in the preceding NUM/MII project days, but those “Hackathons” have often failed to provide the required level of evidence due to multiple (academic) partners having time and resource (and often apparent “motivational”) constraints to collaborate on those tests. Now, having all components under HiGHmed’s fingertips, we are running tests/benchmarking as part of CI/CD pipeline (QA).***
- ***Specifically for openEHR-based modeling, there are challenges (collaborations) around to (internationally) model new data domains. We do not (yet) actively propose such challenges on our own.***

***References :***

1. n/a

***Data Modalities Supported by < YOUR PLATFORM >***

Usually, clinical research data platforms are designed to integrate and manage a wide range of data modalities to support biomedical research. The primary data modalities used so far in

< YOUR PLATFORM > include:

| **Category** | **Data Modality** | **Description** |
| --- | --- | --- |
| **Clinical Data** | Electronic Health Records (EHRs) | Yes, core capability of openEHR HIVEbase |
|  | Hospital Administrative Data | Yes, we (can) keep personal/identifyable data in HIVEvault. Not directly in the EHRbase instance, but adjacent to that in a separate store. |
| **Genomic Data** | Genomic Sequences | No |
|  | Genotype Data | Yes, as part of openEHR modelled genome diagnostic |
|  | Gene Expression Data | Yes, same as Genotype Data |
| **Imaging Data** | Radiology Images | No, images can be referenced but not analysed/shown. |
|  | Pathology Images | No |
| **Phenotypic Data** | Disease Phenotypes | Yes, as part of openEHR’s very precisely modeled (disease-specific) obervations. |
|  | Clinical Outcomes | Yes, this and “Yes” records below: there exist semantically highly resolved openEHR archetypes/templates to describe the domains. |
| **Medication Data** | Prescription Records | Yes |
|  | Medication Adherence / Compliance | Yes |
| **Laboratory Data** | Lab Test Results | Yes |
| **Survey Data** | Questionnaires and Surveys | Yes |
|  | Patient-Reported Outcomes | Yes |
| **Biomarker Data** | Proteomics | Yes |
|  | Metabolomics | No sure .. |
| **Environmental Data** | Lifestyle Factors | Yes |
|  | Environmental Exposures | Yes |
| **Socioeconomic Data** | Social Determinants of Health | Yes |
| **Family History Data** | Genetic Risk Factors | Yes |
| **Longitudinal Data** | Time-Series Data | No (not yet). This is not a domain for openEHR but more specifically a dedicated timeseries database of which data series are referenced in openEHR. |
| **Behavioral Data** | Behavioral Assessments | Yes |
|  | Transcriptomics | Yes (as part of genome diagnostics) |
| **Pathway Data** | Biological Pathways | No |
|  | Interaction Networks | No |

***References :***

1. please provide references to relevant publications / documentation here

**Built-in Workflows and Analysis Tools**

Does < YOUR PLATFORM > contain built-in workflows and analysis tools that facilitate clinical and translational research?

**Workflow**

| **Feature** | **Description** |
| --- | --- |
| Patient Cohort Discovery | Yes, we have cohort-explorer GUI to build (Boolean) queries. |
| Data Integration and Management | Native feature of openEHR, which allows to use existing archetypes and primitives to assemble new templates which cover new domains or join existing one. |
| Ontology Management | No. we delegate this to the sources catering ontologies to our terminology service |
| Data Extraction and Transformation | Any script-based ETL process (ideally in Python, or via py wrapper) implementing the interfaces (status, process control) required by Apache-Airflow which orchestrates a whole set of (subordinate) ETL processes. |
| Security and Privacy Management | See “Features” above, section “Security & Privacy” |

**References:**

1. n/a

**Analysis Tools**

| Query Interface | Yes, “cohort explorer” web UI |
| --- | --- |
| Timeline Viewer | No, not yet, this would be an “App” engineered on top of HIVEbase |
| Statistics and Analytics | Most basically, openEHR AQL queries return JSON, which can be grabbed into Python and treated by any stat library available. |
| Plugin Framework | openEHR HIVE base templates (models) are no-code plug-ins.  The same holds for HIVEconnect mappings which are no-code plug-in. |
| Natural Language Processing (NLP) | No |
| Genomic Data Analysis | No, not yet, this would be an “App” engineered on top of HIVEbase |
| Temporal Querying | Yes, easily doable with openEHR AQL. It is one of the key features of openEHR (in contrast to FHIR stores) that data is organized as a patient centric EHR, easily queryable in the temporal dimension. |
| Data Visualization | No, not yet, this would be an “App” engineered on top of HIVEbase |
| Export and Reporting | See answer abobe (Statistics & Analysis). IN ADDITION, on the SCE (secure compute environment) side (which is still in concept/prototype phase), we anticipate providing privacy-shielded data to Tensorflow/Keras as a Toolbox of choice for AI training |

***References***

*n/a*

| **Integration with Other Tools** | R / BioConductor and Python Integration | See answer above (Statistics & Analysis). |
| --- | --- | --- |
|  | Integration with Clinical Trial Management Systems (CTMS) | No |
|  | Integration with Electronic Health Records (EHR) | HIVEbase (as the core of everything) in HIVE suite *IS* an EHR ! |

References:

1. n/a

**Support for Semantic Integration**

Does < YOUR PLATFORM > support semantic integration through the use of terminologies, ontologies, and common data models? Such as:

1. **Terminologies and Ontologies**: Can < YOUR PLATFORM > integrate with standard medical terminologies and ontologies such as ICD, SNOMED CT, LOINC, and others. This ensures consistent data representation and facilitates interoperability.?
2. **Common Data Models (CDMs)**: Can < YOUR PLATFORM > work with various common data models like the Observational Medical Outcomes Partnership (OMOP) CDM, enabling data standardization and easier data sharing across institutions.?
3. **Ontology Management**: Does the platform include tools for ontology management, allowing users to customize and extend the ontologies as needed to fit their specific research requirements​?

I’m feeling that this has been answered already. Yes, openEHR standard (as the basis for HIVE suite) has extremely strong focus on CDMs through its community-driven modeling efforts (see: ckm.openehr.org). In the models, terminology requirements and restrictions can be incorporated. With regard to OMOP, we provide an openEHR (HIVEbase) export facility towards the OMOP datamodel, allowing users in the OMOP community to make use of the existing analysis toolbox.References go here
