# Supplementary material for: Selecting medical research data platforms for translational biomedical research: a five-tier overview and requirement-weighted assessment framework
Source: Front Digit Health. 2026 Jun 17;8:1814015. doi: 10.3389/fdgth.2026.1814015 (PMC13319098; doi:10.3389/fdgth.2026.1814015)
Supplement: Supplementary file 10 [file Supplementaryfile10.docx]

**HONIC components – Health Data-as-a-Service research platform**

***Short Summary:***

Honic operates a health data research platform, co-developed with the relevant data privacy authority, patient organizations and other stakeholders.

Health care providers (“data providers”) share, often on a daily basis, their care data / RWD / Versorgungsdaten with Honic. Bundesdruckerei (Federal printer of Germany) acts as an external trust center and generates a pseudonym across all provider silos.

Honic has established a comprehensive data privacy architecture that enables the linkage of pseudonymized health data across silos without the necessity of patient consent.

Linked, anonymized data is then accessible for researchers via remote access in a secure processing environment on a separate part of the platform.

***Reference:***

[*If*](https://community.i2b2.org/wiki/display/BUN/Analysis+of+Populations%3A+Install+Guide?preview=/41746456/41746457/worddavf4910d141b05e4133d5e3adb0cc77a8b.png) *the platform architecture has been published, please add a reference here*

***Matrix HONIC features***

| *Criteria* | *Details* |
| --- | --- |
| ***Security and Privacy*** | *Entire platform is governed by security-by-design & privacy-by-design*  *Data on platform is only processed in pseudonymized form*  *Data cannot be accessed by Honic without provider’s consent*  *Data is stored in encrypted provider silos on platform, not in a data lake*  *Researchers have only access to the specific data they requested*  *Data is anonymized after linkage, prior to remote-access for researchers*  *Anonymization is done following the ‘Five Saves’ Framework^1^* |
| ***Compliance and Regulatory Adherence*** | *Platform architecture is co-developed with relevant data privacy authority (LfDI) ^2^*  *Comprehensive compliance architecture includes external trust center (Bundesdruckerei), external compliance board (patient & science representatives have veto rights) etc.* |
| **Interoperability and Extensibility** | ***Standards Support:*** *HL7, FHIR, LOINC, SNOMED CT etc. – supporting all international standard data formats – and grateful for all well documented data*  *But: So far not working with image data*  ***Extensibility****: does HONIC support the adding of new or special standards? Yes*  *Does it allow for connectors to Clinical Information Systems? Was not a requirement yet, but happy to look into it* |
| ***Data Quality and Integrity*** | ***Data Validation****: automated workflows for data quality assessments? Yes*  ***Audit Trails****: audit trails to track data access and modifications?*  *Was not a requirement yet, but happy to look into it*  *Data Provenance: tracking the origin and any changes made to the data ? Yes*  Can your system link heterogeneous data (e.g. genomics data and EHR-derived information)? Yes, we can link data across sectors / silos. And we have a data privacy authority approved architecture that allows the linkage even without patient consent |
| ***Usability and Accessibility*** | ***User Interface****:* ***Training and Support:*** *Does the platform offer training resources and user support?*  *UX is at the core of our company, so the platform should be self explanatory. We didn’t have any customers yet who needed additional guidance*  ***Accessibility:*** *any comments? See above* |
| ***Scalability and Performance*** | ***Handling Large Datasets:*** *can you provide an example for a really complex, big data set?*  *We have real world data from >10 million patients on our platform, coming from labs, outpatient care centers, practice information systems and many other sources. To make this data queryable, link it across silos, anonymize it and prepare it for research is what we do for living.*  ***Performance Metrics:*** *Does the platform monitor performance? (under load)?*  *Yes, we have explored and will migrate almost fully to the Apache tech stack (most notably Apache Iceberg), where performance is greatly mastered* |
| ***Collaboration and Sharing Capabilities*** | Does < YOUR PLATFORM > support federated queries across multiple institutions?  No, not yet. But we are eager to work on these, once we have a sponsor who supports the development /application  ***Collaboration Tools:***  *features like shared workspaces and real-time data sharing.*  *Data remains on the platform, remote access is provided to individual, qualified researchers on a project basis. So no, no real-time data sharing.*  *Researchers can bring their own analysis tools, which we can implement on a container-basis into our research environment. Else, we provide the standard tools, such as R.*  ***Permissions Management:*** *< YOUR PLATFORM > offers fine-grained permissions management to control data access and sharing settings?*  *Yes, regarding access. No, regarding data sharing.* |
| ***Cost and Sustainability*** | ***Cost-Effectiveness:*** *cost of ownership / operation known ?*  *Depends on scope of project: 1. accessing research data for statistical analysis (dashboards) or 2. for specific research projects (project specific data package).*  ***Sustainability:*** *active community adoption ? Flagship projects with strong durable partners ?*  *The platform is operated by a company whose business model is data as a service, thus to provide access to real world data for medical research. The platform is an essential part of the business.* |
| ***Ethical Considerations*** | ***Informed Consent:*** *monitoring usage / compliance with informed consent provided by patients?*  *Thanks to our unique data privacy architecture, we can operate without informed consent from the patient.*  *.* ***Ethical Review:*** *Has the platform undergone an assessment of ethical compliance?*  *The platform was co-developed with the data privacy authority and Germany’s largest patient organization, BAG Selbsthilfe. An external compliance board with veto rights governs the access to data.* |
| ***Innovation and Adaptability*** | ***New Technologies:*** *< Honic > prepared to incorporate new technologies and methodologies as they emerge?*  *Yes, we are an agile software company, constantly iterate and try out new technologies.*  ***Flexibility:*** *Is the platform flexible and can it be adapted to new research needs and changes in regulatory requirements?*  *Yes* |

***References***

1. [*https://ukdataservice.ac.uk/help/secure-lab/what-is-the-five-safes-framework/*](https://ukdataservice.ac.uk/help/secure-lab/what-is-the-five-safes-framework/)
2. *https://www.honic.eu/documents/25/220905_Honic_LFDI_final.pdf*

***Matrix Honic common challenges***

| **Category** | **Description** |
| --- | --- |
| **Federated Queries Challenges** | Challenges include proper accounting for same-patient data across multiple nodes, imputation of missing data points, and aggregating similar data referred to using different ontologies.  Not applicable yet |
| **Patient Privacy and Data Protection** | Ensuring consistent data protection throughout the platform  At the core of what we do. Platform is data privacy by design & security by design. |
| **Organizational Policies** | does < Honic > address the complex organizational policies inherent in hospital operations?  No, we focus so far only on outpatient care data. Hospital data will follow in 2025 |
| **Data Transformation requirements** | Requirements for ETL ? Global schemata / common data models? Mapping: who is doing that?  We have developed our own FHIR-based common data model and map all incoming data onto it ourselves to safeguard quality. |
| **Installation and Maintenance** | Ease of installation, deployment in clouds (??), maintenance, operations ?  Build on Kubernetes, hosted in the Stackit-cloud but designed cloud agnostic. Platform can be migrated to another cloud or used in a multi-cloud approach |
| **Secure Deployment** | Deploying < Honic > in a secure network: challenges? Cloud deployability ? References for secure deployment?  We operate the platform on our own |
| **Understanding User Queries** | How do clinical and translational researchers perform queries in < YOUR PLATFORM > ? Use cases? Published examples ? Application scenarios ?  Researchers get remote access to our research environment / secure processing environment, where they can analyse the data via R or other pre-qualified analysis tools.  We will start to build our own automatic query tool (“Cohort Explorer”) starting in 2025, to automate and ease feasibility studies. |
| **Informatics and User Experience** | Is the user “shielded” from the informatics core, or how does the platform orchestrate the collaboration between computer science and medical researcher?  Yes, only remote access to the research environment, no access whatsoever to the core platform  How direct can a clinical researcher address a retrieval or analytics challenge?  We store the path to the specific research project, but we destroy the specific data package after the research is completed. |
| **Complexity of < YOUR PLATFORM > Software** | How complex is your software? How easily can it be adapted to new environments / new application scenarios?  We deployed mainly open source software, mainly from Apache. So comparatively easy to adapt it, plus very active open source community. |
| **Incremental Updating Limitations** | de-identification issues?  Anonymization is a multi-dimensional challenge. We build on the Five Save framework (see above) to minimize de-identification. Already at project application stage, external compliance board checks if access to specific data is needed, or e.g. statistical analysis would also do the trick.  By controlling the entire ETL process from data ingest over data normalization, quality improvement, linkage & anonymization, in a very structured, well-documented approach, we try to minimize known data quality challenges.  , exposure of provisional data?, potential database fragmentation? How does < YOUR PLATFORM > handle these aspects ? |
| **Standardized Vocabularies and Flexibility** | How does < YOUR PLATFORM > enable adoption of controlled vocabularies and other standards (e.g. metadata) ?  Wherever possible, we use international data standards like Snomed CT, and document every step. Regarding Metadata, we currently look into DCAT AP and are in close contact with BfArM and other KOLs. |

- ***does your community organize data challenges / platform challenges? Is there any benchmarking available?***

***References :***

1. please add references here or directly in the fields of the matrix

***Data Modalities Supported by < YOUR PLATFORM >***

Usually, clinical research data platforms are designed to integrate and manage a wide range of data modalities to support biomedical research. The primary data modalities used so far in

< YOUR PLATFORM > include:

| **Category** | **Data Modality** | **Description** |
| --- | --- | --- |
| **Clinical Data** | Electronic Health Records (EHRs) | Structured data (please specify) and unstructured data (please add information on indexing / information extraction possible).  We can access all relevant health data directly from care providers. If helpful, we provide you with our data catalogs, summarizing all data that we have per sector (Lab, outpatient care etc). |
|  | Hospital Administrative Data | Admissions, discharges, transfers, billing codes, and insurance information.  No hospital data yet, coming in 2025 |
| **Genomic Data** | Genomic Sequences | Whole genome, exome sequencing, targeted sequencing (please specify and provide references).  No genomic data yet, coming in 2026 |
|  | Genotype Data | Single nucleotide polymorphisms (SNPs), copy number variations (CNVs). Please specify and provide references. |
|  | Gene Expression Data | What types of transcriptomics data? |
| **Imaging Data** | Radiology Images | MRI, CT, X-ray, ultrasound. Please specify and provide references if possible  No image data yet |
|  | Pathology Images | Digital pathology slides, histology images. Please specify and provide references if possible  No image data yet |
| **Phenotypic Data** | Disease Phenotypes | Disease characteristics, symptom severity, progression. Use of HPO or other controlled vocabularies for annotation? Please provide references if possible.  We can access all relevant health data directly from care providers. If helpful, we provide you with our data catalogs, summarizing all data that we have per sector (Lab, outpatient care etc). |
|  | Clinical Outcomes | Treatment responses, survival rates, recurrence. Please specify and provide references if possible. |
| **Medication Data** | Prescription Records | (Co-)Medication names, dosages, administration routes, duration. Please add references if possible.  So far, only data from prescriber (e.g. GPs), not from the pharmacy (distributer). Pharmacy data is top priority, will be on platform latest 2026 |
|  | Medication Adherence / Compliance | e.g. Refill records, patient self-reports. |
| **Laboratory Data** | Lab Test Results | Blood tests, urine tests, microbiological cultures, biochemical assays. Biomarker measurements. Please provide references if possible.  Extensive lab data. Can provide you with lab catalog, if helpful. Currently onboarding Germany’s 2^nd^ largest lab chain. |
| **Survey Data** | Questionnaires and Surveys | Patient health questionnaires, lifestyle surveys, mental health assessments. Please provide references if possible.  Not yet |
|  | Patient-Reported Outcomes | Pain scales, quality of life measures, functional status. Please provide references if possible.  Not yet |
| **Biomarker Data** | Proteomics | Protein expression, protein-protein interactions, post-translational modifications. Mass-Spec, MALDI whatsoever. Blood and urine proteomics? References ??  Not yet |
|  | Metabolomics | Metabolite profiles, metabolic pathways, lipidomics. |
| **Environmental Data** | Lifestyle Factors | Diet, physical activity, workout schemata, smoking, alcohol consumption, substance (ab)use.  Not yet, if not documented in e.g. GP EHR |
|  | Environmental Exposures | Air quality, water quality, exposure to toxins, occupational hazards.  Not yet, but if data is public, rather easy to add and link to patient data |
| **Socioeconomic Data** | Social Determinants of Health | Education, income, employment status, housing, neighborhood characteristics.  Not yet, but interested to add |
| **Family History Data** | Genetic Risk Factors | Family history of diseases, pedigree analysis. Risk alleles, tumor gene panels etc.  Not yet, if not documented in e.g. GP EHR |
| **Longitudinal Data** | Time-Series Data | Repeated measures over time, disease progression, treatment responses over time. Please provide information on how you organize information in time (from time stamp to longitudinal representation of patients).  Yes, we can link data per patient longitudinal, also across silos (e.g. link data from one patient across GP, labs & specialists) |
| **Behavioral Data** | Behavioral Assessments | Cognitive tests, psychological assessments, behavioral interventions. Nutrition coaching? Please specify and provide references, if possible.  Not yet |
|  | Transcriptomics | mRNA levels, non-coding RNAs, alternative splicing events.  Not yet |
| **Pathway Data** | Biological Pathways | Signaling pathways, metabolic pathways. Mechanism graphs. Pathophysiology graphs (disease maps) ?  Not yet |
|  | Interaction Networks | Protein-protein interaction networks, gene regulatory networks. Co-expression networks ?  Not yet |

***References :***

1. please provide references to relevant publications / documentation here

**Built-in Workflows and Analysis Tools**

Does < YOUR PLATFORM > contain built-in workflows and analysis tools that facilitate clinical and translational research?

**Workflow**

| **Feature** | **Description** |
| --- | --- |
| Patient Cohort Discovery | Create and manage patient cohorts based on criteria such as demographics, diagnoses, medications, procedures, and lab results. Based on study data catalogues and mappings? See for instance [https://adata.scai.fraunhofer.de](https://adata.scai.fraunhofer.de/) as an example for such an indication-wide study discovery engine.  We are working on a fully automated cohort explorer tool, will be ready end of 2025 |
| Data Integration and Management | Integrate heterogeneous data sources into a common data model (CDM), including clinical, genomic, and other research data. Does < YOUR PLATFORM > support the usage and mapping to CDMs?  Yes, our CDM grows constantly by adding new data sources |
| Ontology Management | Create and manage ontologies for organizing and categorizing (meta-)data, making it easier to search and analyze. Does < YOUR PLATFORM > comprise an ontology store / lookup service?  Yes |
| Data Extraction and Transformation | What ETL does < YOUR PLATFORM > support? References ?  We have our own ETL process, happy to walk through, if helpful |
| Security and Privacy Management | Ensure data security and patient privacy with mechanisms like role-based access control and data de-identification.  Yes, all of it |

**References:**

1. references go here

**Analysis Tools**

| Query Interface | Main interface for creating queries to identify patient cohorts based on various clinical and demographic criteria. Please specify  So far manual work by our team, will be automated with the Cohort Explorer (in development) |
| --- | --- |
| Timeline Viewer | Visualize individual patient timelines, displaying events such as diagnoses, treatments, and lab results over time. Please specify for  < YOUR PLATFORM >  All data can be accessed via statistical tools such as R, we do not provide specific interfaces, if not requested/paid for |
| Statistics and Analytics | Basic statistical tools to analyze query results, including counts, distributions, and summary statistics.  See above |
| Plugin Framework | Integrate external analysis tools and custom plugins to extend the platform's capabilities. Please provide references, examples and documentation of plugin architecture.  Yes, we can integrate any external tool in a containerized form, but we need to check the tool first to safeguard our platform & data |
| Natural Language Processing (NLP) | Does < YOUR PLATFORM > comprise already integrated NLP services? Are they open source?  Not yet, but we need to safeguard that a NLP does not misuse our data (exports, de-identifies etc.) |
| Genomic Data Analysis | Integrate and analyze genomic data alongside clinical data, often requiring additional modules or plugins.  Not yet |
| Temporal Querying | Perform queries that consider the temporal sequence of events, such as identifying patients who had a particular treatment before a specific diagnosis.  Yes, by linking data across silos in a longitudinal approach, you can build such temporal sequences |
| Data Visualization | Basic tools for visualizing data distributions and query results, extendable with additional plugins for advanced visualization.  See above |
| Export and Reporting | Does < YOUR PLATFORM > allow for export of query results for further analysis or reporting purposes in formats compatible with other statistical and data analysis software.  Yes, see above. But: no data leaves our platform, so all data analysis have to be done on our platform |

***References***

*1.*references go here

| **Integration with Other Tools** | R / BioConductor and Python Integration | Use R and Python scripts for advanced statistical analysis and machine learning workflows. Please specify and provide references …  Yes, R and Python are our two default tools already integrated in our research environment |
| --- | --- | --- |
|  | Integration with Clinical Trial Management Systems (CTMS) | Is < YOUR PLATFORM > integrated with CTMS for managing clinical trial data and workflows?.  Not yet |
|  | Integration with Electronic Health Records (EHR) | Does < YOUR PLATFORM > allow for seamless integration with EHR systems to pull in clinical data for analysis?  Define seamless in the German context Medatixx is e.g. one of our strategic partners and we can access their full data stream from >2.400 doctors |

References:

1. references go here

**Support for Semantic Integration**

Does < YOUR PLATFORM > support semantic integration through the use of terminologies, ontologies, and common data models? Such as:

1. **Terminologies and Ontologies**: Can < YOUR PLATFORM > integrate with standard medical terminologies and ontologies such as ICD, SNOMED CT, LOINC, and others. This ensures consistent data representation and facilitates interoperability.?

Yes, see above. We are very happy about and support all international standard data formats like Snomed, Loinc etc.

1. **Common Data Models (CDMs)**: Can < YOUR PLATFORM > work with various common data models like the Observational Medical Outcomes Partnership (OMOP) CDM, enabling data standardization and easier data sharing across institutions.?

We have developed our own, FHIR-based and well documented CDM. From our CDM, we can easily transform the data into OMOP or other formats

1. **Ontology Management**: Does the platform include tools for ontology management, allowing users to customize and extend the ontologies as needed to fit their specific research requirements​?

Not yet, we have our own well documented ontology, would need to understand requirements beyond that.

**References** :

1. References go here
