# Supplementary material for: Selecting medical research data platforms for translational biomedical research: a five-tier overview and requirement-weighted assessment framework
Source: Front Digit Health. 2026 Jun 17;8:1814015. doi: 10.3389/fdgth.2026.1814015 (PMC13319098; doi:10.3389/fdgth.2026.1814015)
Supplement: Supplementary file 11 [file Supplementaryfile11.docx]

***I2B2 platform***

***Deployment and Usage****:*

*i2b2 is a scalable informatics framework designed by Harvard Medical School with NIH funding, and it's utilized by over 250 research institutions worldwide. It is particularly noted for transforming patient-oriented clinical data to facilitate clinical research. At the Mayo Clinic, i2b2 has been used extensively since 2016 for feasibility analysis, study design, cohort identification, patient recruitment, and population health research. It has supported over 600 researchers in these tasks​ ​.*

***References:***

1. [*https://www.mayo.edu/research/centers-programs/center-clinical-translational-science/resources/i2b2-informatics-for-integrating-biology-and-the-bedside*](https://www.mayo.edu/research/centers-programs/center-clinical-translational-science/resources/i2b2-informatics-for-integrating-biology-and-the-bedside)
2. [*https://www.i2b2.org/about/intro.html*](https://www.i2b2.org/about/intro.html)
3. [*https://www.frontiersin.org/journals/artificial intelligence/articles/10.3389/frai.2021.769582/full*](https://www.frontiersin.org/journals/artificial%20intelligence/articles/10.3389/frai.2021.769582/full)

**I2B2 component**


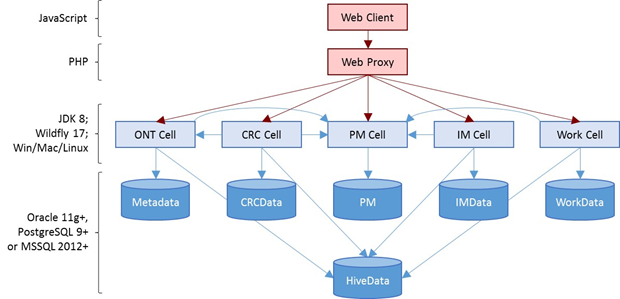


***Reference:***

[*https://community.i2b2.org/wiki/display/BUN/Analysis+of+Populations%3A+Install+Guide?preview=/41746456/41746457/worddavf4910d141b05e4133d5e3adb0cc77a8b.png*](https://community.i2b2.org/wiki/display/BUN/Analysis+of+Populations%3A+Install+Guide?preview=/41746456/41746457/worddavf4910d141b05e4133d5e3adb0cc77a8b.png)

***Matrix table for the i2b2 platform’ features***

| *Criteria* | *Details* |
| --- | --- |
| ***Security and Privacy(1)*** | *Data Encryption: i2b2 ensures encryption of data both in transit and at rest, safeguarding sensitive information. Access Controls: It employs robust access control mechanisms to restrict data access to authorized personnel only. Anonymization: The platform provides features for anonymizing and de-identifying patient data to protect patient privacy.* |
| ***Compliance and Regulatory Adherence(2,3,4,5,6)*** | *HIPAA Compliance: i2b2 is compliant with HIPAA regulations, ensuring the privacy and security of health information. GDPR Compliance: For data from the EU, i2b2 ensures GDPR compliance, protecting the privacy and personal data of individuals. Local Regulations: i2b2 adheres to other relevant local and international regulations, such as the CCPA for data from California.* |
| **Interoperability and Extensibility(7,8,9)** | *Standards Support: i2b2 supports major interoperability standards like HL7, FHIR (Fast Healthcare Interoperability Resources), and DICOM, ensuring seamless data exchange. EHR Integration: The platform integrates with various Electronic Health Record (EHR) systems, facilitating the exchange and management of clinical data.*  ***Extensibility****: The platform’s modular design allows for the addition of new modules and functionalities as needed, making it adaptable to evolving research requirements​* |
| ***Data Quality and Integrity(10,11)*** | *Data Validation: i2b2 implements mechanisms for data validation, ensuring the accuracy and consistency of data. Audit Trails: The platform maintains comprehensive audit trails to track data access and modifications. Data Provenance: i2b2 ensures data provenance, tracking the origin and any changes made to the data.* i2b2 supports the integration of data from various sources, including EHRs, genomic data, and patient-reported outcomes. This capability is crucial for comprehensive clinical and translational research |
| ***Usability and Accessibility(12,13)*** | *User Interface: Training and Support: The platform offers extensive training resources and user support to help users effectively utilize the platform. Accessibility: i2b2 complies with accessibility standards, ensuring that all users, regardless of their abilities, can effectively use the platform* |
| ***Scalability and Performance(14,15)*** | *Handling Large Datasets: i2b2 is capable of efficiently handling large volumes of data, making it suitable for large-scale research and clinical applications. Performance Metrics: The platform monitors and maintains high performance in terms of speed and responsiveness, even under high load conditions.* |
| ***Collaboration and Sharing Capabilities(16,17,18,19)*** | i2b2 supports federated queries across multiple institutions, enabling collaborative research on a larger scale. This feature is particularly valuable for large-scale genomic studies and other projects requiring extensive datasets from multiple sources. *Collaboration Tools:*  *i2b2 facilitates collaboration among researchers with features like shared workspaces and real-time data sharing. Permissions Management: The platform offers fine-grained permissions management to control data access and sharing settings.* |
| ***Cost and Sustainability(20,21)*** | *Cost-Effectiveness: i2b2 offers good value for money, providing a comprehensive set of features at a reasonable cost. Sustainability: The platform ensures long-term sustainability with continuous vendor support and active community adoption.* |
| ***Ethical Considerations(22)*** | *Informed Consent: i2b2 includes mechanisms to ensure that data sharing complies with the informed consent provided by patients. Ethical Review: The platform has processes for ethical review and oversight of data sharing activities, ensuring compliance with ethical standards.* |
| ***Innovation and Adaptability(23,24)*** | *New Technologies: i2b2 is designed to incorporate new technologies and methodologies as they emerge, keeping the platform current and innovative. Flexibility: The platform is flexible and can adapt to new research needs and changes in regulatory requirements.* |

***References***

*1.*[*https://doi.org/10.1136/amiajnl-2012-001042*](https://doi.org/10.1136/amiajnl-2012-001042)

2.[*https://ctsi.wakehealth.edu/service/data-and-design/data-extraction*](https://ctsi.wakehealth.edu/service/data-and-design/data-extraction)

3.[*https://www.ncbi.nlm.nih.gov/pmc/articles/PMC3000779/*](https://www.ncbi.nlm.nih.gov/pmc/articles/PMC3000779/)

4.[*https://ebooks.iospress.nl/doi/10.3233/SHTI220554*](https://ebooks.iospress.nl/doi/10.3233/SHTI220554)

5.[*https://www.sciencedirect.com/science/article/pii/B9780128228845000167*](https://www.sciencedirect.com/science/article/pii/B9780128228845000167)

*6.https://opus.bibliothek.uni-augsburg.de/opus4/frontdoor/deliver/index/docId/39540/file/ME17-02-0022.pdf*

***7.*** [*https://academic.oup.com/jamia/article/20/1/172/2909248?login=false*](https://academic.oup.com/jamia/article/20/1/172/2909248?login=false)

8.[*https://www.ncbi.nlm.nih.gov/pmc/articles/PMC6371332/*](https://www.ncbi.nlm.nih.gov/pmc/articles/PMC6371332/)

*9.https://academic.oup.com/jamia/article/24/2/398/2631471?login=false*

*10.* [*https://www.ncbi.nlm.nih.gov/pmc/articles/PMC3041580/*](https://www.ncbi.nlm.nih.gov/pmc/articles/PMC3041580/)

*11.https://www.ncbi.nlm.nih.gov/pmc/articles/PMC9285160/*

***12.*** [*https://www.sciencedirect.com/science/article/pii/S1532046418301369*](https://www.sciencedirect.com/science/article/pii/S1532046418301369)

*13.https://academic.oup.com/jamia/article/18/Supplement_1/i103/796270*

*14.* [*https://academic.oup.com/jamia/article/24/2/398/2631471*](https://academic.oup.com/jamia/article/24/2/398/2631471)

*15.https://www.sciencedirect.com/science/article/pii/S1532046418301369*

16.[*https://community.i2b2.org/wiki/*](https://community.i2b2.org/wiki/)

17.[*https://catalyst.harvard.edu/wp-content/uploads/2021/03/SHRINE-Natter_et_al_2012.pdf*](https://catalyst.harvard.edu/wp-content/uploads/2021/03/SHRINE-Natter_et_al_2012.pdf)

18.[*https://academic.oup.com/jamia/article/24/2/398/2631471?login=false*](https://academic.oup.com/jamia/article/24/2/398/2631471?login=false)

*19.https://www.ncbi.nlm.nih.gov/pmc/articles/PMC3000779/*

*20.* [*https://www.ncbi.nlm.nih.gov/pmc/articles/PMC3631913/*](https://www.ncbi.nlm.nih.gov/pmc/articles/PMC3631913/)

*21.*[*https://community.i2b2.org/wiki/*](https://community.i2b2.org/wiki/)

***22.*** [*https://www.ncbi.nlm.nih.gov/pmc/articles/PMC3241166/*](https://www.ncbi.nlm.nih.gov/pmc/articles/PMC3241166/)

*23. https://community.i2b2.org/wiki/display/RM/Upgrade+i2b2*

24.[*https://www.i2b2.org/software/*](https://www.i2b2.org/software/)

***Matrix table for the i2b2 platform’ common challenges***

| **Category** | **Description** |
| --- | --- |
| **Federated Queries Challenges(1)** | Challenges include proper accounting for same-patient data across multiple nodes, imputation of missing data points, and aggregating similar data referred to using different ontologies. |
| **Patient Privacy and Data Protection(2)** | Ensuring consistent data protection throughout the platform despite the adaptability of i2b2 to local site patient privacy requirements. |
| **Organizational Policies(3)** | i2b2 core implementation does not address the complex organizational policies inherent in hospital operations. |
| **Data Transformation for i2b2 CRC(4)** | Local knowledge and resources are required for data transformations when placing data into the i2b2 CRC, posing significant challenges. |
| **Installation and Maintenance(5)** | i2b2 is challenging to install and maintain, including patching, upgrading, data modeling, and ontology mapping, which hinders wider adoption. |
| **Secure Deployment(6)** | Deploying i2b2 in a secure network is challenging. AWS cloud infrastructure offers a solution for easier maintenance and scalability. |
| **Understanding User Queries (7)** | Limited literature on how clinical and translational researchers perform i2b2 queries, with studies mainly focused on specific use cases or enterprise-wide usage at select sites. |
| **Informatics and User Experience(8)** | Understanding the usage of i2b2 by researchers with varying informatics expertise can help informatics professionals improve service delivery, measure effects, and describe the value of i2b2. |
| **Complexity of i2b2 Software(9)** | i2b2 is complex due to its generic implementation for a wide range of operations. It is developed by domain experts using an agile approach, making it challenging to test and install due to the variety of use cases. |
| **Incremental Updating Limitations(10)** | Challenges include de-identification issues, exposure of provisional data, significant initial effort, and potential database fragmentation over time, which may result in performance degradation and require downtime to address. |
| **Standardized Vocabularies and Flexibility(11)** | Limited use of standardized vocabularies and low flexibility in adapting to the diverse representations of cancer concepts and their values across different sites. |

- ***over 100 publications in research journals or conferences were enabled by i2b2 challenges (REF***[***www.i2b2.org/NLP/DataSets/Publications.php***](http://www.i2b2.org/NLP/DataSets/Publications.php)***)***

***References :***

1. [*https://academic.oup.com/jamia/article/20/1/172/2909248*](https://academic.oup.com/jamia/article/20/1/172/2909248)
2. [*https://academic.oup.com/jamia/article/18/Supplement_1/i103/796270*](https://academic.oup.com/jamia/article/18/Supplement_1/i103/796270)
3. [*https://academic.oup.com/jamia/article/18/Supplement_1/i103/796270*](https://academic.oup.com/jamia/article/18/Supplement_1/i103/796270)*)*
4. [*https://academic.oup.com/jamia/article/17/2/124/2909101*](https://academic.oup.com/jamia/article/17/2/124/2909101)*)*
5. [*https://www.proquest.com/openview/900e3899582bbe38101d6049755e8156/1?pq-origsite=gscholar&cbl=18750&diss=y*](https://www.proquest.com/openview/900e3899582bbe38101d6049755e8156/1?pq-origsite=gscholar&cbl=18750&diss=y)*)*
6. [*https://www.proquest.com/openview/900e3899582bbe38101d6049755e8156/1?pq-origsite=gscholar&cbl=18750&diss=y*](https://www.proquest.com/openview/900e3899582bbe38101d6049755e8156/1?pq-origsite=gscholar&cbl=18750&diss=y)*)*
7. [*https://www.ncbi.nlm.nih.gov/pmc/articles/PMC7233105/*](https://www.ncbi.nlm.nih.gov/pmc/articles/PMC7233105/)
8. [*https://www.ncbi.nlm.nih.gov/pmc/articles/PMC7233105/*](https://www.ncbi.nlm.nih.gov/pmc/articles/PMC7233105/)
9. [*https://journals.sagepub.com/doi/full/10.1177/1178222618777749*](https://journals.sagepub.com/doi/full/10.1177/1178222618777749)
10. [*https://www.ncbi.nlm.nih.gov/pmc/articles/PMC5977612*](https://www.ncbi.nlm.nih.gov/pmc/articles/PMC5977612)
11. https://www.sciencedirect.com/science/article/pii/S1532046423002265)

***Data Modalities Supported by i2b2***

The i2b2 platform is designed to integrate and manage a wide range of data modalities to support biomedical research. The primary data modalities involved in i2b2 platforms include.

| **Category** | **Data Modality** | **Description** |
| --- | --- | --- |
| **Clinical Data(1)** | Electronic Health Records (EHRs) | Structured data (ICD codes, medications, lab results) and unstructured data (clinical notes). |
|  | Hospital Administrative Data | Admissions, discharges, transfers, billing codes, and insurance information. |
| **Genomic Data(2)** | Genomic Sequences | Whole genome, exome sequencing, targeted sequencing. |
|  | Genotype Data | Single nucleotide polymorphisms (SNPs), copy number variations (CNVs). |
|  | Gene Expression Data | Microarray, RNA-seq, qPCR. |
| **Imaging Data(3)** | Radiology Images | MRI, CT, X-ray, ultrasound. |
|  | Pathology Images | Digital pathology slides, histology images. |
| **Phenotypic Data(1)** | Disease Phenotypes | Disease characteristics, symptom severity, progression. |
|  | Clinical Outcomes | Treatment responses, survival rates, recurrence. |
| **Medication Data(4)** | Prescription Records | Medication names, dosages, administration routes, duration. |
|  | Medication Adherence | Refill records, patient self-reports. |
| **Laboratory Data(5)** | Lab Test Results | Blood tests, urine tests, microbiological cultures, biochemical assays. |
| **Survey Data(6)** | Questionnaires and Surveys | Patient health questionnaires, lifestyle surveys, mental health assessments. |
|  | Patient-Reported Outcomes | Pain scales, quality of life measures, functional status. |
| **Biomarker Data(7)** | Proteomics | Protein expression, protein-protein interactions, post-translational modifications. |
|  | Metabolomics | Metabolite profiles, metabolic pathways, lipidomics. |
| **Environmental Data(8)** | Lifestyle Factors | Diet, physical activity, smoking, alcohol consumption, substance use. |
|  | Environmental Exposures | Air quality, water quality, exposure to toxins, occupational hazards. |
| **Socioeconomic Data(9)** | Social Determinants of Health | Education, income, employment status, housing, neighborhood characteristics. |
| **Family History Data(10)** | Genetic Risk Factors | Family history of diseases, pedigree analysis. |
| **Longitudinal Data(11)** | Time-Series Data | Repeated measures over time, disease progression, treatment responses over time. |
| **Behavioral Data(12)** | Behavioral Assessments | Cognitive tests, psychological assessments, behavioral interventions. |
|  | Transcriptomics | mRNA levels, non-coding RNAs, alternative splicing events. |
| **Pathway Data(13)** | Biological Pathways | Signaling pathways, metabolic pathways. |
|  | Interaction Networks | Protein-protein interaction networks, gene regulatory networks. |

***References :***

1. <https://www.sciencedirect.com/science/article/pii/S1532046416300843>
2. <https://journals.plos.org/plosone/article?id=10.1371/journal.pone.0172187>
3. <https://www.thieme-connect.com/products/ejournals/html/10.1055/s-0038-1651497>
4. <https://www.ncbi.nlm.nih.gov/pmc/articles/PMC4765563/>
5. <https://academic.oup.com/jamia/article/24/2/398/2631471>
6. <https://www.thieme-connect.com/products/ejournals/abstract/10.4338/ACI-2014-11-RA-0106>
7. <https://www.sciencedirect.com/science/article/pii/S1532046423002265>
8. <https://www.atsjournals.org/doi/full/10.34197/ats-scholar.2023-0028OC>
9. <https://core.ac.uk/download/pdf/28950404.pdf>
10. <https://medinform.jmir.org/2021/4/e24020/>
11. <https://medinform.jmir.org/2020/7/e15918/>
12. <https://link.springer.com/article/10.1186/s12911-020-01239-2>
13. https://www.annalsofoncology.org/article/S0923-7534(20)34213-7/fulltext

**Built-in Workflows and Analysis Tools**

i2b2 provides several built-in workflows and analysis tools that facilitate clinical and translational research:

**Workflow**

| **Feature** | **Description** |
| --- | --- |
| Patient Cohort Discovery(1,2) | Create and manage patient cohorts based on criteria such as demographics, diagnoses, medications, procedures, and lab results. |
| Data Integration and Management(3) | Integrate heterogeneous data sources into a common data model, including clinical, genomic, and other research data. |
| Ontology Management(4) | Create and manage ontologies for organizing and categorizing data, making it easier to search and analyze. |
| Data Extraction and Transformation(5) | Extract data from various sources and transform it into a format compatible with the i2b2 data model. |
| Security and Privacy Management(6) | Ensure data security and patient privacy with mechanisms like role-based access control and data de-identification. |

**References:**

1. <https://www.ncbi.nlm.nih.gov/pmc/articles/PMC4371505/>
2. <https://link.springer.com/chapter/10.1007/978-3-319-57741-8_8>
3. <https://link.springer.com/chapter/10.1007/978-3-319-57741-8_8>
4. <https://link.springer.com/chapter/10.1007/978-3-319-57741-8_8>
5. <https://journals.plos.org/plosone/article?id=10.1371/journal.pone.0212463>
6. <https://www.thieme-connect.com/products/ejournals/abstract/10.4338/ACI-2010-09-CR-0051>

**Analysis Tools**

| Query Tool(1) | Main interface for creating queries to identify patient cohorts based on various clinical and demographic criteria. |
| --- | --- |
| Timeline Viewer(2) | Visualize individual patient timelines, displaying events such as diagnoses, treatments, and lab results over time. |
| Statistics and Analytics(1) | Basic statistical tools to analyze query results, including counts, distributions, and summary statistics. |
| Plugin Framework(3) | Integrate external analysis tools and custom plugins to extend the platform's capabilities. |
| Natural Language Processing (NLP)(4) | Extract structured data from unstructured clinical notes, , enhancing the breadth of data that can be analyzed |
| Genomic Data Analysis(5) | Integrate and analyze genomic data alongside clinical data, often requiring additional modules or plugins. |
| Temporal Querying(6, 7) | Perform queries that consider the temporal sequence of events, such as identifying patients who had a particular treatment before a specific diagnosis. |
| Data Visualization(8,9) | Basic tools for visualizing data distributions and query results, extendable with additional plugins for advanced visualization. |
| Export and Reporting(10) | Export query results for further analysis or reporting purposes in formats compatible with other statistical and data analysis software. |

***References***

*1.*[*https://journals.plos.org/plosone/article?id=10.1371/journal.pone.0172187*](https://journals.plos.org/plosone/article?id=10.1371/journal.pone.0172187)

*2.* [*https://link.springer.com/chapter/10.1007/978-3-319-57741-8_8*](https://link.springer.com/chapter/10.1007/978-3-319-57741-8_8)

*3.* [*https://www.ncbi.nlm.nih.gov/pmc/articles/PMC5333310/*](https://www.ncbi.nlm.nih.gov/pmc/articles/PMC5333310/)

*4.* [*https://www.thieme-connect.com/products/ejournals/abstract/10.4338/ACI-2014-11-RA-0106*](https://www.thieme-connect.com/products/ejournals/abstract/10.4338/ACI-2014-11-RA-0106)

*5.* [*https://www.sciencedirect.com/science/article/pii/S1877050914003573*](https://www.sciencedirect.com/science/article/pii/S1877050914003573)

*6.* [*https://onlinelibrary.wiley.com/doi/full/10.1155/2019/5640685*](https://onlinelibrary.wiley.com/doi/full/10.1155/2019/5640685)

*7.* [*https://academic.oup.com/jamia/article/17/2/124/2909101*](https://academic.oup.com/jamia/article/17/2/124/2909101)

*8.https://web.archive.org/web/20200321111622id_/http://telemedicina.unifesp.br/pub/AMIA/2007%20AMIA%20Proceedings/data/papers/posters/AMIA-0463-S2007.pdf*

*9.* [*https://www.ncbi.nlm.nih.gov/pmc/articles/PMC4371505/*](https://www.ncbi.nlm.nih.gov/pmc/articles/PMC4371505/)

*10. https://community.i2b2.org/wiki/display/webclient/Analysis+Tools*

| **Integration with Other Tools** | R and Python Integration(1,2) | Use R and Python scripts for advanced statistical analysis and machine learning workflows. |
| --- | --- | --- |
|  | Integration with Clinical Trial Management Systems (CTMS) | Integrate with CTMS for managing clinical trial data and workflows. |
|  | Integration with Electronic Health Records (EHR)(3) | Seamless integration with EHR systems to pull in clinical data for analysis. |

References:

1. <https://academic.oup.com/jamia/article/18/3/314/700370>
2. <https://academic.oup.com/bioinformatics/article/38/20/4833/6687125>
3. <https://academic.oup.com/jamia/article/23/5/909/2379861>

**Support for Semantic Integration**

i2b2 supports semantic integration through the use of terminologies, ontologies, and common data models:

1. **Terminologies and Ontologies**: i2b2 can integrate with standard medical terminologies and ontologies such as ICD, SNOMED CT, LOINC, and others. This ensures consistent data representation and facilitates interoperability.(1,2)
2. **Common Data Models (CDMs)**: i2b2 can work with various common data models like the Observational Medical Outcomes Partnership (OMOP) CDM, enabling data standardization and easier data sharing across institutions. (3,4)
3. **Ontology Management**: The platform includes tools for ontology management, allowing users to customize and extend the ontologies as needed to fit their specific research requirements​.

**References** :

- 1. <https://www.ncbi.nlm.nih.gov/pmc/articles/PMC7233105/>
  2. <https://ebooks.iospress.nl/doi/10.3233/SHTI220460>
  3. https://journals.plos.org/plosone/article?id=10.1371/journal.pone.0212463
  4. https://www.sciencedirect.com/science/article/pii/S1532046423002265

*Here comes the “empty” form to add your information. All my comments are labeled in blue. They can be removed. Would be good if you chose another color for your input.*

1. References go here
