# Supplementary material for: Selecting medical research data platforms for translational biomedical research: a five-tier overview and requirement-weighted assessment framework
Source: Front Digit Health. 2026 Jun 17;8:1814015. doi: 10.3389/fdgth.2026.1814015 (PMC13319098; doi:10.3389/fdgth.2026.1814015)
Supplement: Supplementary file 12 [file Supplementaryfile12.docx]

Prof. Martin Hofmann-Apitius

Department of Bioinformatics

Fraunhofer Institute for Algorithms and Scientific Computing (SCAI)

7/12/24

Dear Colleagues

Our team at Fraunhofer Institute for Algorithms and Scientific Computing (SCAI) is currently working on a systematic overview on medical data sharing platforms, virtual research environments for translational medical research and federated learning platforms.

Our goal is a systematic assessment of platforms with respect to such diverse aspects like:

| *Criteria* |
| --- |
| *Security and Privacy* |
| *Compliance and Regulatory Adherence* |
| Interoperability and Extensibility |
| *Data Quality and Integrity* |
| *Usability and Accessibility* |
| *Scalability and Performance* |
| *Collaboration and Sharing Capabilities* |
| *Cost and Sustainability* |
| *Ethical Considerations* |

We also look into data modalities and examples for successful application of these platforms. Goal of the entire exercise is a rather complete overview on medical research data platforms (we do not consider this a trivial task, as the landscape of tools and systems is super complex).

We contacted you, because you are the PI / CEO/CSO behind such a platform. We want to ask you, whether you want to be part of this systematic assessment. In that case, we ask you to fill-in the attached matrix. We provide an example for I2B2 that reflects our own data collection effort for this particular platform.

I would be really grateful if you could provide us with your input for your platform. We will acknowledge each contributor to this overview and we promise to share the manuscript of our review BEFORE we submit it so that you can see what we will be writing.

Our aim is to bring some order into the rather complex world of medical research data sharing and learning platforms. We believe that this systematic assessment is of great value for the entire research community. It will also be quite helpful for the industry, as we are doing a large part of your job when it comes to generating competitor matrices …

Thank you for your time and effort

Looking forward to hearing from you

Prof. Martin Hofmann-Apitius, PhD

Professor for Applied Life Science Informatics

University of Bonn

Head of the Department of Bioinformatics

Fraunhofer Institute for Algorithms and Scientific Computing (SCAI)

*Hi Phil,*

*In blue: instructions from Martin/Marc*

*In black: my answers, please check if you agree.*

*When with red background: I wasn’t sure if that was appropriate.*

***<IDERHA > platform***

***Deployment and Usage****:*

*<*IDERHA (Integration of Heterogeneous Data and Evidence towards Regulatory and HTA Acceptance) is a European public-private partnership launched in April 2023 and offers an open, disease agnostic, federated data platform which enables connectivity, access, use and reuse of digital health data among academic and industry partners to maximize data value for medical research and ultimately patient care. To pave the way towards regulatory acceptance of real-world evidence, IDERHA develops consensus policy recommendations for regulators and HTA decision-making bodies. As IDERHA is part of the [Europe Beating Cancer Plan](https://commission.europa.eu/strategy-and-policy/priorities-2019-2024/promoting-our-european-way-life/european-health-union/cancer-plan-europe_en), it uses artificial intelligence and machine learning to improve early detection of e.g. lung cancer and the quality of life for patients. IDERHA enables a data-driven approach for clinical co-decision making and personalized disease management. *>*

***References:***

1. Gyrard A, Gribbon P, Hussein R, Abedian S, Bonmati LM, Cabornero GL, Manias G, Danciu G, Dalmiani S, Autexier S, van Nuland R, Jendrossek M, Avramidis I, Alvarez EG. Synergies Among Health Data Projects with Cancer Use Cases Based on Health Standards. Stud Health Technol Inform. 2024 Aug 22;316:1292-1296. doi: 10.3233/SHTI240649. PMID: 39176618.
2. [*https://www.iderha.org/about*](https://www.iderha.org/about)
3. [*https://www.iderha.org/outreach/corporate-slide-deck*](https://www.iderha.org/outreach/corporate-slide-deck)
4. [*https://www.iderha.org/outreach/public-deliverables*](https://www.iderha.org/outreach/public-deliverables)
5. *https://www.linkedin.com/company/94277941*

**< IDERHA > components**


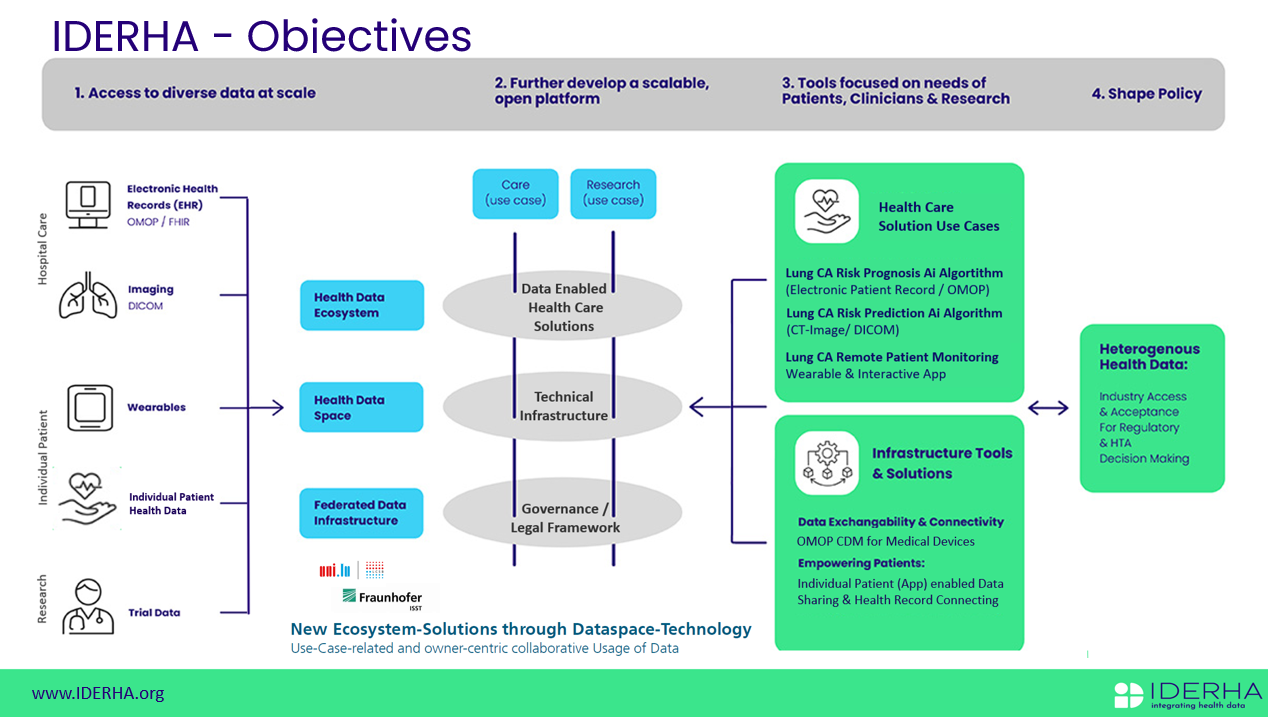


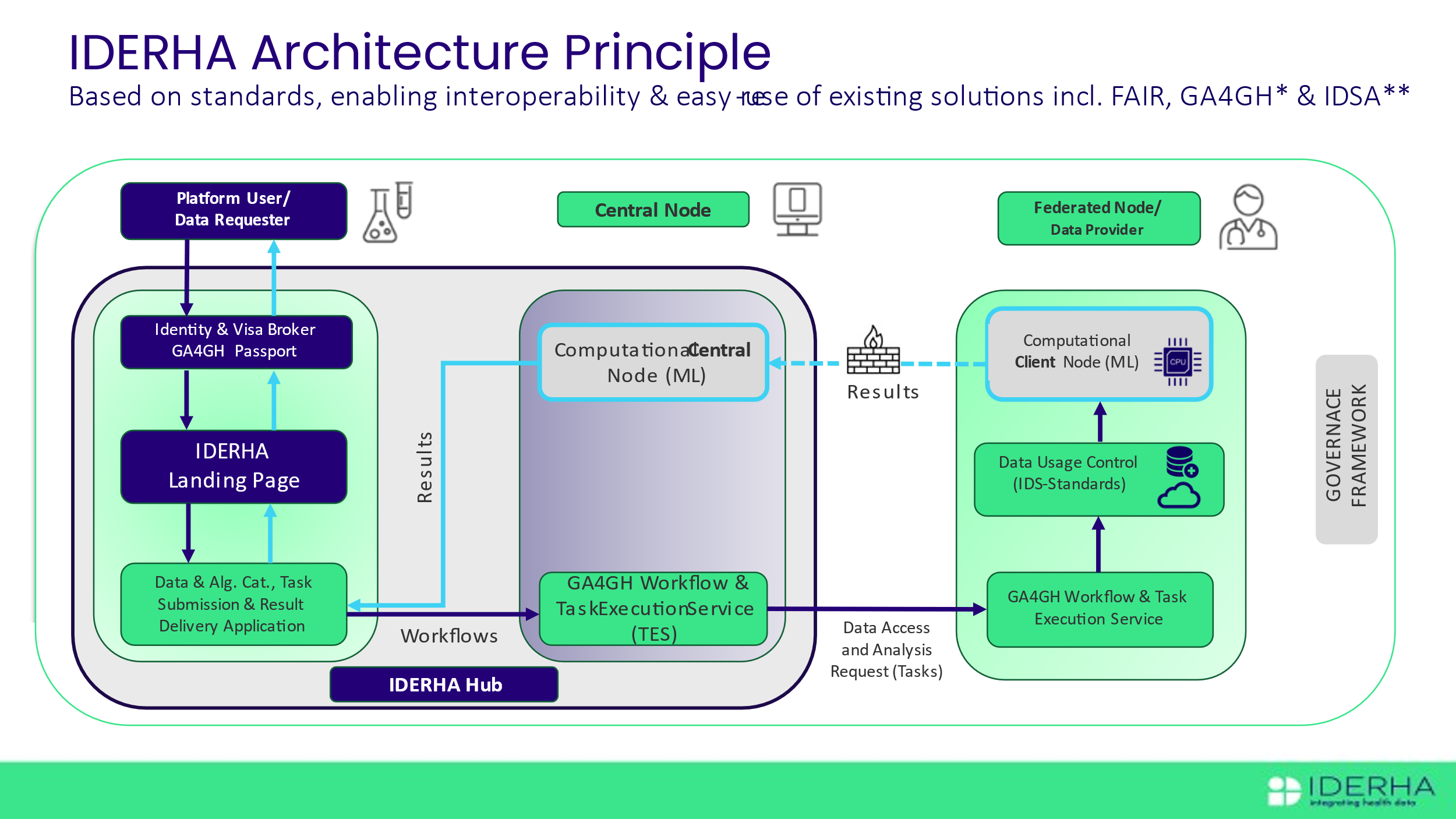


***Reference:***

1. Boutsma, E. (2024, Oktober 15). IDERHA Corporate Slide Deck. Zenodo. <https://doi.org/10.5281/zenodo.13934884>

***Matrix < IDERHA > features***

| *Criteria* | *Details* |
| --- | --- |
| ***Security and Privacy*** | *Please add information on Data Encryption; Access Control (Authorization; Authentification); Anonymization Options. Please add references wherever possible*  General aspects: Data always remains at the site of the data holder site, not in a data lake. Researchers access data through algorithm deployment at the federated node level and only aggregated data is returned to the central node. Data holders/providers remain in full control of the data access and processing, while the central components of the IDERHA platform are only responsible for the orchestration and brokering of existing, approved grants and analyses. IDERHA only brokers externally signed and verifiable information for identity and access.  Through OpenID Connect (User authentication and authorisation (SSO, MFA)), IDERHA will obtain signed ID tokens for the user of such providers and broker them through the central node alongside any analysis or access requests to the federated nodes, where they may be verified for authenticity directly with the issuing provider. Potentially malicious access attempts with modified or self-issued ID tokens will not be able to pass this check. After the validation of the token, it is now up to the data provider to decide on whether they accept this information or initiate further identity verification processes before they authorise the access. The platform follows security-by-design and privacy-by-design principles and e.g. the Five Safes for trusted research environments (Safe Data, Safe Projects, Safe People, Safe Settings, Safe Outputs) framework.   - Choice of identity providers still an ongoing discussion (e.g. LifeScience RI, eduGAIN, eIDAS, d-trust..)   Centralised trusted research environment (TRE): Source databases are handled by data custodian and should be encrypted at rest. Data is made accessible for analysis under data minimization principles, but is not shared directly with researcher. Only approved algorithms are stored in the TRE and available for remote execution. New algorithms may be submitted to the Algorithm Review committee and approved through manual review process.  Federated TRE: traffic between components in different networks is encrypted. Traffic between components inside the same network is isolated through virtualization (Docker/ Kubernetes) and can also be encrypted if needed. Algorithms (container images) are fetched from a strictly controlled non-public registry. Source database in federated node sits in a restricted subnet (only egress through EDC).  IDERHA supports flexible on-premise or cloud (AWS, Azure..) deployments. Computational workloads (e.g. AI models) are run after a GA4GH workflow and task execution service on the computational client node of the IDERHA federated node.  IDERHA platform software is installed on a virtual machine or physical server behind the data custodian’s firewall, whether on-prem in a data center or on a Virtual Private Cloud (VPC). The client can securely access local datasets and has access to GPUs/CPUs in order to securely run computational workloads (e.g., AI-model training) without moving sensitive data outside the firewall. |
| ***Compliance and Regulatory Adherence*** | *Please add information on aspects such as HIPAA Compliance; GDPR Compliance (Europe); Local Regulations (that may apply). Please add references wherever possible*  IDERHA complies with GDPR and local national requirements.  Under consideration of Art. 26 of the General Data Protection Regulation (the “GDPR”), the IDERHA parties acknowledge their roles as Joint Controllers of the Personal Data processed in the context of IDERHA and therefore concluded a joint controller agreement. A joint data management plan further details the data flows and data processing. This legal governance framework assists each data controller, especially the clinical sites, to conduct data protection impact assessments. In addition, a code of conduct to implement information security measures. In IDERHA, a special focus is given to the ethical development of AI. |
| **Interoperability and Extensibility** | ***Standards Support:*** *things like HL7, FHIR, and DICOM. Please comment on EHR Integration:* ***Extensibility****: does < IDERHA > support the adding of new or special standards? Does it allow for connectors to Clinical Information Systems?*  Open-source tools and components are used for the IDERHA platform, therefore allowing for a wide applicability and allowing for scalability into other disease areas apart from lung cancer.  IDERHA supports the integration and use of electronic health records in OMOP and FHIR format, imaging data in DICOM, individual patient data from wearables and research data from clinical trials for secondary research purposes. |
| ***Data Quality and Integrity*** | ***Data Validation****: automated workflows for data quality assessments?* ***Audit Trails****: audit trails to track data access and modifications? Data Provenance: tracking the origin and any changes made to the data ?.* Can your system link heterogeneous data (e.g. genomics data and EHR-derived information)?  The IDERHA data quality framework covers dataset-specific and lifecycle-based (from primary source to data transformation, secondary use) dimensions, with a particular focus on data quality for AI training. The IDERHA quality framework builds on the work of the QUANTUM consortia (https://quantumproject.eu/ ) which is establishing a quality label for data sets  IDERHA uses a unified approach to auditing across the platform and standardization of the logs across all services, which would also enable requested features, such as transparency reporting. IDERHA uses platform and workflow monitoring for obtaining information about service availability, processing status, request status, etc. |
| ***Usability and Accessibility*** | ***User Interface****:* ***Training and Support:*** *Does the platform offer training resources and user support?.* ***Accessibility:*** *any comments ?*  To support the process to make the platform should be self-explanatory (UX), IDERHA provides an IDERHA guidebook to support platform operators, data holders (clinical sites) and users (e.g.. algorithm developers) in the onboarding process and the usage of the IDERHA platform. 1:1 onboarding for data holders is foreseen. In addition, IDERHA develops learning materials related to skills on standards and FAIR principles to support the implementation of selected standards and methods.  IDERHA provides a dash board for data access requests and approvals. |
| ***Scalability and Performance*** | ***Handling Large Datasets:*** *can you provide an example for a really complex, big data set?.* ***Performance Metrics:*** *Does the platform monitor performance? (under load)??*  The IDERHA architecture permits existing platforms to integrate and connect with new data sources in the health data spaces. Open-source tools and components are used for the IDERHA platform, therefore allowing for a wide applicability and allowing for scalability into other disease areas apart from lung cancer.  With regards to the federated learning: Once an algorithm is approved, it can potentially be executed on numerous federated nodes if data access permissions are in place. |
| ***Collaboration and Sharing Capabilities*** | Does < IDERHA > support federated queries across multiple institutions?. ***Collaboration Tools:***  *features like shared workspaces and real-time data sharing.* ***Permissions Management:*** *< IDERHA > offers fine-grained permissions management to control data access and sharing settings?.*  Data Sovereignty: Data remains local and under custodian control, processed only within custodian firewalls = Data never leaves the data holder site. = ensuring data privacy.  Federated analysis: Algorithms are executed on distributed/ federated nodes according to data access permissions. |
| ***Cost and Sustainability*** | ***Cost-Effectiveness:*** *cost of ownership / operation known ?.* ***Sustainability:*** *active community adoption ? Flagship projects with strong durable partners ?.*  IDERHA platform development is funded by the EU-IHI programme. Operational fees will apply for the platform usage to maintain the IDERHA central node with its metadata catalogue and connections to federated nodes. Potential funding could also be coming from upcoming EU or national projects to further adapt the platform to extended use cases. |
| ***Ethical Considerations*** | ***Informed Consent:*** *monitoring usage / compliance with informed consent provided by patients?.* ***Ethical Review:*** *Has the platform undergone an assessment of ethical compliance?.*  Compliance with ethical standards is ensured through local data provider ethics committees and the IDERHA ethics committee for oversight of data sharing activities. Informed consent is the legal basis for personal data processing undertaken in most data made accessible through IDERHA. Data holders have pre-existing consent that covers IDERHA purposes and use. Analysed data in IDERHA is fully anonymized and uses OMOP and DiCOM standards. |
| ***Innovation and Adaptability*** | ***New Technologies:*** *< IDERHA > prepared to incorporate new technologies and methodologies as they emerge?.* ***Flexibility:*** *Is the platform flexible and can it be adapted to new research needs and changes in regulatory requirements?.*  IDERHA is built on tools and standards that have been developed by various previous initiatives, for examples from partners in the ELIXIR community, and IDERHA is constantly monitoring community efforts and new regulations (e.g. EHDS, AI Act) in the field, in addition to pro-actively engaging with platform users to adapt the platform to user requirements and use cases.  Our approach also provides a certain robustness to changing requirements and plans, as we are not building a monolithic platform, but instead deploy many small, specialized services, which can be easily replaced by other components if needed. And finally, the choice of using known standards is an important step to enable interoperability and allows for the development of reusable client software, which will be important for researchers to connect their specialised local tooling (e.g., for model training monitoring). |

***References***

1. Boenert, E., Herzinger, S., Ćwiek-Kupczyńska, H., Scheider, S., Geller, D., Kamal Mallick, M., & Satagopam, V. (2024). Data and analysis platform architecture (D1.1). Zenodo. <https://doi.org/10.5281/zenodo.13919268>
2. Alper P, Dĕd V, Herzinger S, Grouès V, Peter S, Lebioda J, Ebermann L, Popleteeva M, Barry ND, Welter D, Ghosh S, Becker R, Schneider R, Gu W, Trefois C, Satagopam V. DS-PACK: Tool assembly for the end-to-end support of controlled access human data sharing. Sci Data. 2024 May 15;11(1):501. doi: 10.1038/s41597-024-03326-9. PMID: 38750048; PMCID: PMC11096168.
3. “Home | LifeScience RI.” Accessed: Mar. 24, 2024. [Online]. Available: https://lifescience-ri.eu/home.html
4. “eduGAIN – enabling worldwide access.” Accessed: Apr. 06, 2024. [Online]. Available: https://edugain.org/
5. “eIDAS Regulation | Shaping Europe’s digital future.” Accessed: Apr. 06, 2024. [Online]. Available: https://digital-strategy.ec.europa.eu/en/policies/eidas-regulation

***Matrix <IDERHA > common challenges***

| **Category** | **Description** |
| --- | --- |
| **Federated Queries Challenges** | Challenges include proper accounting for same-patient data across multiple nodes, imputation of missing data points, and aggregating similar data referred to using different ontologies.  The IDERHA infrastructure is being designed based on the principle of interoperability and should be generic enough to support different FML solutions in the  future, the first focus will be on one centralised FML framework of choice: NVIDIA FLARE.  NVIDIA FLARE (NVFlare) is an open source software deployment kit for federated learning that has been integrated in the IDERHA platform. |
| **Patient Privacy and Data Protection** | Ensuring consistent data protection throughout the platform  IDERHA developed a data protection guidance framework as an overarching information governance assessment providing a data protection impact assessment (DPIA) framework designed to review at a higher level the data protection risks associated with the initiative across all partners and their local jurisdictions. |
| **Organizational Policies** | does < IDERHA > address the complex organizational policies inherent in hospital operations?.  IDERHA data holders define usage policies which will be displayed in the metadata catalogue and implemented in data access workflows. The Eclipse Dataspace Components (EDC) will be the clearing house for access control. EDC uses svisas to control data processing at the IDERHA federated nodes. |
| **Data Transformation requirements** | Requirements for ETL ? Global schemata / common data models? Mapping: who is doing that?  IDERHA generally does not get involved in clinical data mapping activities to a data model, but provides guidelines and can help to connect to experts in that field (e.g. ITTM, AIDAVA, Data Steward from SCAI). |
| **Installation and Maintenance** | Ease of installation, deployment in clouds (??), maintenance, operations ?  For the connection to the IDERHA central node (including the IDERHA landing page and the data and algorithm catalogue, the submission and result delivery application, the computational central node and the Ga4GH Workflow and Task Execution Service), the IDERHA federated node installation packages can be installed on-premise or in the cloud. |
| **Secure Deployment** | Deploying < IDERHA > in a secure network: challenges? Cloud deployability ? References for secure deployment?  IDERHA components can be deployed in a cloud- or on-premise setting. |
| **Understanding User Queries** | How do clinical and translational researchers perform queries in < IDERHA > ? Use cases? Published examples ? Application scenarios ?  IDERHA users (e.g. algorithm developers) can submit their developed algorithms to IDERHA. The IDERHA algorithm evaluation committee then evaluates the algorithm. After approval and after the data user has successfully applied for data access (incl ethical approvals and application of data minimization principles) at specific data provider sites (federated nodes), the algorithm can be executed on the federated infrastructure data. |
| **Informatics and User Experience** | Is the user “shielded” from the informatics core, or how does the platform orchestrate the collaboration between computer science and medical researcher? How direct can a clinical researcher address a retrieval or analytics challenge?  A clinical researcher can browse the metadata catalogue after user authentication and authorization to identify suitable datasets for research studies. Once identified, the clinical researcher can apply for access and send her/his algorithm to the IDERHA algorithm evaluation committee for approval. |
| **Complexity of < IDERHA > Software** | How complex is your software? How easily can it be adapted to new environments / new application scenarios?  IDERHA is built on existing software and tool components, partially open-access. Components are constantly further developed by the ELIXIR community. |
| **Incremental Updating Limitations** | de-identification issues?, exposure of provisional data?, potential database fragmentation? How does < IDERHA > handle these aspects ?  IDERHA integrates solutions such as federated learning to prevent direct data exposure, ontology-based data integration (DCAT..) to maintain data consistency.  To further maintain data consistency and avoid inconsistent or fragmented datasets (e.g. different versions of data coexisting, making e.g. analysis difficult), IDERHA uses standardized data models (e.g. OMOP). |
| **Standardized Vocabularies and Flexibility** | How does < IDERHA > enable adoption of controlled vocabularies and other standards (e.g. metadata) ?  By aligning ourselves with major communities like GA4GH, ELIXIR, and IDS we discovered various (microservice) standards. Being part of these communities, IDERHA partners will be able to contribute to further development of existing standards and service implementations through the use cases that are driven by the IDERHA project requirements. |

- ***does your community organize data challenges / platform challenges? Is there any benchmarking available?***

***References :***

1. NVIDIA FLARE,” NVIDIA Developer. Accessed: Mar. 24, 2024. [Online]. Available:

https://developer.nvidia.com/fl

***Data Modalities Supported by < IDERHA >***

Usually, clinical research data platforms are designed to integrate and manage a wide range of data modalities to support biomedical research. The primary data modalities used so far in

< IDERHA > include:

| **Category** | **Data Modality** | **Description** |
| --- | --- | --- |
| **Clinical Data** | Electronic Health Records (EHRs) | Structured data (please specify) and unstructured data (please add information on indexing / information extraction possible).  Structured data from patient EHRs, including clinical notes, diagnoses, medications, and lab results. |
|  | Hospital Administrative Data | Admissions, discharges, transfers, billing codes, and insurance information. |
| **Genomic Data** | Genomic Sequences | Whole genome, exome sequencing, targeted sequencing (please specify and provide references). |
|  | Genotype Data | Single nucleotide polymorphisms (SNPs), copy number variations (CNVs). Please specify and provide references. |
|  | Gene Expression Data | What types of transcriptomics data? |
| **Imaging Data** | Radiology Images | MRI, CT, X-ray, ultrasound. Please specify and provide references if possible  Medical imaging data such as MRI, CT, X-ray, and ultrasound images in DICOM format |
|  | Pathology Images | Digital pathology slides, histology images. Please specify and provide references if possible |
| **Phenotypic Data** | Disease Phenotypes | Disease characteristics, symptom severity, progression. Use of HPO or other controlled vocabularies for annotation? Please provide references if possible. |
|  | Clinical Outcomes | Treatment responses, survival rates, recurrence. Please specify and provide references if possible. |
| **Medication Data** | Prescription Records | (Co-)Medication names, dosages, administration routes, duration. Please add references if possible.  Structured data from patient EHRs, including clinical notes, diagnoses, medications, and lab results. |
|  | Medication Adherence / Compliance | e.g. Refill records, patient self-reports. |
| **Laboratory Data** | Lab Test Results | Blood tests, urine tests, microbiological cultures, biochemical assays. Biomarker measurements. Please provide references if possible.  Clinical data including demography, medical history, medical treatment (immunotherapy, chemotherapy, concomitant medication), physical examination, ECOG assessment, lab values, adverse events, tumor assessments, Quality of Life Questionnaires |
| **Survey Data** | Questionnaires and Surveys | Patient health questionnaires, lifestyle surveys, mental health assessments. Please provide references if possible.  Quality of Life Questionnaires |
|  | Patient-Reported Outcomes | Pain scales, quality of life measures, functional status. Please provide references if possible.  Short daily questions to assess adverse events between clinic visits, patients’ well-being, and their overall healthcare experience including their perceptions about the impact of the treatment, patient satisfaction and general patient information |
| **Biomarker Data** | Proteomics | Protein expression, protein-protein interactions, post-translational modifications. Mass-Spec, MALDI whatsoever. Blood and urine proteomics? References ?? |
|  | Metabolomics | Metabolite profiles, metabolic pathways, lipidomics. |
| **Environmental Data** | Lifestyle Factors | Diet, physical activity, workout schemata, smoking, alcohol consumption, substance (ab)use. |
|  | Environmental Exposures | Air quality, water quality, exposure to toxins, occupational hazards.  Open data on environmental exposure available from European Environment Agency |
| **Socioeconomic Data** | Social Determinants of Health | Education, income, employment status, housing, neighborhood characteristics. |
| **Family History Data** | Genetic Risk Factors | Family history of diseases, pedigree analysis. Risk alleles, tumor gene panels etc. |
| **Longitudinal Data** | Time-Series Data | Repeated measures over time, disease progression, treatment responses over time. Please provide information on how you organize information in time (from time stamp to longitudinal representation of patients). |
| **Behavioral Data** | Behavioral Assessments | Cognitive tests, psychological assessments, behavioral interventions. Nutrition coaching? Please specify and provide references, if possible. |
|  | Transcriptomics | mRNA levels, non-coding RNAs, alternative splicing events. |
| **Pathway Data** | Biological Pathways | Signaling pathways, metabolic pathways. Mechanism graphs. Pathophysiology graphs (disease maps) ? |
|  | Interaction Networks | Protein-protein interaction networks, gene regulatory networks. Co-expression networks ? |

***References :***

1. Boutsma, E. (2024, Oktober 15). IDERHA Corporate Slide Deck. Zenodo. <https://doi.org/10.5281/zenodo.13934884>

**Built-in Workflows and Analysis Tools**

Does < IDERHA > contain built-in workflows and analysis tools that facilitate clinical and translational research?

IDERHA builds on a modular, flexible and scalable infrastructure, which works with independent but standardized components. It is based on the following key design principles: privacy and security by design (data providers control data access and analysis, the platform orchestrates data access and analysis requests, only pre-approved algorithms can be executed, data protection at-rest and in-motion), FAIR data principles (user facing catalogs and linking services, advanced user authentication and data governance framework, connect-ability of external tools and platforms using established standards, data versioning, licensing and clear documentation for data use), and data space principles (policy-based data governance, trust framework for sovereign and secure data sharing and processing, data sovereignty as the capability of entities to exercise all rights on their data via platform functions).

The following infrastructure components related to cloud computing and API standards are part of IDERHA: Task Execution Service (consistent task execution across network), Task Execution Service Gateway (coordination of task execution services, central routing of tasks to the federated nodes), workflow execution service (enabling complex workflows, support of parallel task executions with centralised result aggregation), Tool Registry Service (abstraction of algorithmic resources for task execution service, definition of and cataloguing of tools and workflows, access to approved computational resources) and a dataset registry service (abstraction of data storage, mapping of resource IDs to URLs, identification of datasets via IDs to facilitate references in the system, complementation of Tool Registry Service in cataloging workflows.

IDERHA offers a metadata catalogue that automates the synchronization of metadata from different data holder sites, enables advanced search and filtering capabilities for efficient clinical data discovery, and interface for initiation and tracking of data access requests, and functionalities for transferring documents with respect to data holders governance restrictions.

Users can provide algorithms to IDERHA. If applicable, an algorithm evaluation committee evaluates the algorithm and might add it to the approved platform algorithms. Once approved, federated learning is executed from the central node to different local data holders (federated nodes). After the analysis, the updated model parameters (e.g. neural network weights) are sent back to the computational central node and the results are displayed to the platform user. IDERHA develops AI/ML personalized malignancy risk prediction using CT scans based on structured EHR data and CT scans and malignancy diagnostic of low dose CT images.

**Workflow**

| **Feature** | **Description** |
| --- | --- |
| Patient Cohort Discovery | Create and manage patient cohorts based on criteria such as demographics, diagnoses, medications, procedures, and lab results. Based on study data catalogues and mappings? See for instance <https://adata.scai.fraunhofer.de> as an example for such an indication-wide study discovery engine. |
| Data Integration and Management | Integrate heterogeneous data sources into a common data model (CDM), including clinical, genomic, and other research data. Does < IDERHA > support the usage and mapping to CDMs?  IDERHA decided to adopt the OMOP-CDM and Observational Health Data Sciences and Informatics (OHDSI) vocabularies for clinical data exchange. IDERHA supports the usage of common data models such as OMOP, but only provides guidelines and support, but does not get involved in the mapping activities.  Further work is needed on mapping data in OMOP (FHIR, CDISC..). There are still challenges, particularly in standardizing areas like environmental exposure, medical devices, cancer data, and patient-reported experience measures/ patient-reported outcome measures (PREMs/ PROMS). |
| Ontology Management | Create and manage ontologies for organizing and categorizing (meta-)data, making it easier to search and analyze. Does < IDERHA > comprise an ontology store / lookup service?  IDERHA is planning to use Health DCAT-AP- as Resource Description Framework vocabulary for the description of datasets and services in the IDERHA metadata catalogue. |
| Data Extraction and Transformation | What ETL does < IDERHA > support? References ?  IDERHA builds on the OMOP CDM that was developed by OHDSI (Observational Health data Sciences and Informatics Intiative) Each table of the OMOP CDM is represented with a high-level description and ETL conventions that should be followed. |
| Security and Privacy Management | Ensure data security and patient privacy with mechanisms like role-based access control and data de-identification.  IDERHA architecture built based on security and privacy-by-design principles (see above). |

**References:**

1. references go here

**Analysis Tools**

| Query Interface | Main interface for creating queries to identify patient cohorts based on various clinical and demographic criteria. Please specify  IDERHA provides a landing page/user interface which manages the user authentication and authorization, will display the metadata catalogue enabling high-level of data and algorithm, and enable task submission and result delivery. Pre-approved algorithms presented to IDERHA will be executed in a federated way on federated notes. |
| --- | --- |
| Timeline Viewer | Visualize individual patient timelines, displaying events such as diagnoses, treatments, and lab results over time. Please specify for  < IDERHA > |
| Statistics and Analytics | Basic statistical tools to analyze query results, including counts, distributions, and summary statistics.  Basic tools such as R and SAS are used within IDERHA |
| Plugin Framework | Integrate external analysis tools and custom plugins to extend the platform's capabilities. Please provide references, examples and documentation of plugin architecture. |
| Natural Language Processing (NLP) | Does < IDERHA > comprise already integrated NLP services? Are they open source? |
| Genomic Data Analysis | Integrate and analyze genomic data alongside clinical data, often requiring additional modules or plugins. |
| Temporal Querying | Perform queries that consider the temporal sequence of events, such as identifying patients who had a particular treatment before a specific diagnosis. |
| Data Visualization | Basic tools for visualizing data distributions and query results, extendable with additional plugins for advanced visualization. |
| Export and Reporting | Does < IDERHA > allow for export of query results for further analysis or reporting purposes in formats compatible with other statistical and data analysis software. |

***References***

*1.*references go here

| **Integration with Other Tools** | R / BioConductor and Python Integration | Use R and Python scripts for advanced statistical analysis and machine learning workflows. Please specify and provide references … |
| --- | --- | --- |
|  | Integration with Clinical Trial Management Systems (CTMS) | Is < IDERHA > integrated with CTMS for managing clinical trial data and workflows?.  IDERHA is suitable for patient cohort discoverability through the IDERHA metadata catalogue in the IDERHA central node. |
|  | Integration with Electronic Health Records (EHR) | Does < IDERHA > allow for seamless integration with EHR systems to pull in clinical data for analysis?  IDERHA can work with EHR data that is located in the federated node. The hospital data is analysed in this secure processing environment and is never transferred to the IDERHA central node. |

References:

1. references go here

**Support for Semantic Integration**

Does < IDERHA > support semantic integration through the use of terminologies, ontologies, and common data models? Such as:

1. **Terminologies and Ontologies**: Can < IDERHA > integrate with standard medical terminologies and ontologies such as ICD, SNOMED CT, LOINC, and others. This ensures consistent data representation and facilitates interoperability.?

IDERHA is planning to work with HealthDVAT-AP.

IDERHA decided to adopt the OMOP-CDM and Observational Health Data Sciences and Informatics (OHDSI) vocabularies for clinical data exchange. Maintained as open-source resources, the OHDSI vocabularies promote transparency in their generation processes. OHDSI employs OMOP vocabularies to standardize health data across multiple domains. These key domains include Conditions (e.g., SNOMED CT, ICD-10), Drugs (e.g., RxNorm, ATC), Procedures (e.g., CPT, HCPCS), Measurements (e.g., LOINC for laboratory results), Observations (e.g., SNOMED CT for patient-reported outcomes), and Devices (e.g., FDA device classifications, SNOMED CT for devices). Each domain uses these terminologies to provide consistency and interoperability, enabling large-scale observational research and improving screening/prediction of healthcare outcomes through a standardized vocabulary framework. OHDSI has developed a searchable database called Athena. Which serves as a resource for researchers seeking to identify and map codes to their corresponding OMOP equivalents. This tool facilitates the standardized use of medical terminology within the OMOP-CDM by providing researchers with a comprehensive repository of standardized vocabularies. It enables researchers to search for specific conditions, such as gestational diabetes, and retrieve relevant information, including the Domain ID, Concept IDs for related terms, and a visual representation of hierarchical relationships. This repository and hierarchy helps researchers to explore the hierarchical structure of concepts, identifying ancestors (e.g., diabetes mellitus during pregnancy) and descendants (e.g., postpartum gestational diabetes).

1. **Common Data Models (CDMs)**: Can < IDERHA > work with various common data models like the Observational Medical Outcomes Partnership (OMOP) CDM, enabling data standardization and easier data sharing across institutions.?

IDERHA works with OMOP.

1. **Ontology Management**: Does the platform include tools for ontology management, allowing users to customize and extend the ontologies as needed to fit their specific research requirements​?

Ontology customization might be enabled in the algorithm submission and deployment part.

**References:**

1. References go here
