# Supplementary material for: Selecting medical research data platforms for translational biomedical research: a five-tier overview and requirement-weighted assessment framework
Source: Front Digit Health. 2026 Jun 17;8:1814015. doi: 10.3389/fdgth.2026.1814015 (PMC13319098; doi:10.3389/fdgth.2026.1814015)
Supplement: Supplementary file 13 [file Supplementaryfile13.docx]

Indoc Systems – Pilot Data Management and Analysis Platform

*Here comes the “empty” form to add your information. All my comments are labeled in blue. They can be removed. Would be good if you chose another color for your input.*

***Deployment and Usage****:*

Pilot is a data infrastructure solution for secure management, analysis, and sharing of complex medical research data. It powers data coordinating centres, translational research programs, and trusted research environments for big science initiatives, hospitals, research networks, and health systems. Pilot supports ingestion, harmonization, and integration of a broad range of data types and sources including clinical assessments, patient-reported outcomes, electronic medical records, wearables, and various forms of genomic and imaging modalities. The Pilot architecture delivers scalability and extensibility in cloud, on-premises, or hybrid deployments.

Reference deployments include the data coordinating centre for the BD^2^ translational research program in bipolar disorder; the Brain-CODE neuroinformatics platform supporting several large-scale deep phenotyping research networks; the Ontario Health Data Platform (OHDP), a trusted research environment holding health system and administrative data for over 15 million residents; the Public Health Analytics Environment that enables infectious disease surveillance and reporting of pathogenic genomic variants; the GDPR-compliant Virtual Research Environment (VRE) deployed at the Charité Hospital Berlin; and the Health Data Cloud, an extension of the Human Brain Project/EBRAINS research data infrastructure for the management and sharing of sensitive data.

*
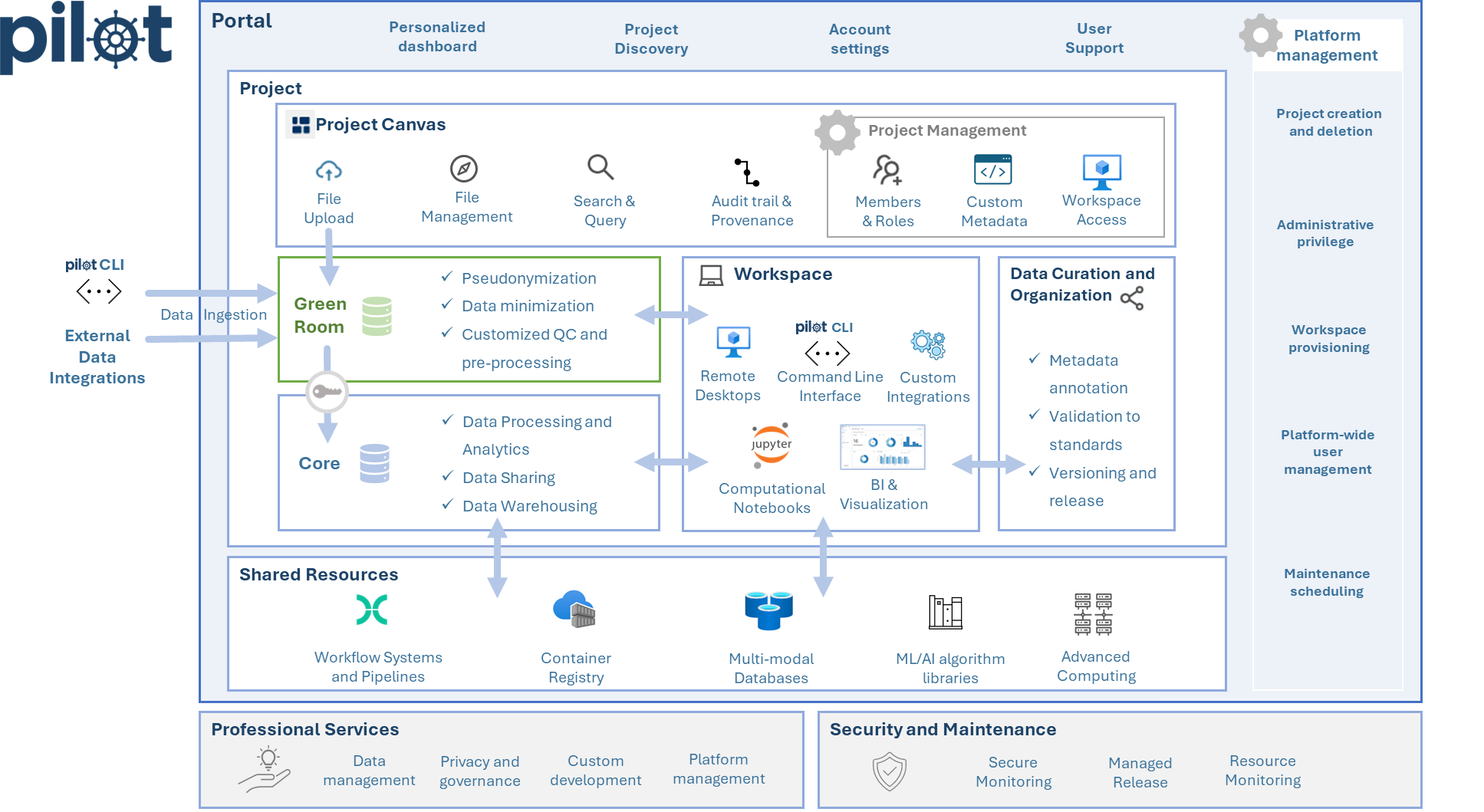
*

***References:***

1. S. Pollentier, A. Vaccarino, Data integration and sharing – easier said than done? From Brain-CODE to a Pilot ecosystem solution, Neuroscience Applied, Volume 1, Supplement 2, 2022, 100365, ISSN 2772-4085, <https://doi.org/10.1016/j.nsa.2022.100365>.
2. Yung C, Dharsee M, Dong F*,* Evans K, Gilbert Evans S, Gee T, Javadi M, MacPhee C, Pollentier S, Shahnazari S*.* Pilot: Making research data FAIR. *F1000Research* 2022, **11**:1397; <https://doi.org/10.7490/f1000research.1119237.1>.
3. Breakthrough Discoveries for thriving with Bipolar Disorder (BD^2^): <https://www.bipolardiscoveries.org/>;
4. Inside BD²’s Integrated Network: Coordinating Clinical & Biological Data from 4,000 Participants to Rapidly Improve Lives. <https://www.bipolardiscoveries.org/2024/09/inside-bd2s-integrated-network-coordinating-clinical-and-biological-data-from-4000-participants-to-rapidly-improve-lives/>
5. Anthony L. Vaccarino, Moyez Dharsee, Stephen Strother, Don Aldridge, Stephen R. Arnott, Brendan Behan, Costas Dafnas, Fan Dong, Kenneth Edgecombe, Rachad El-Badrawi, Khaled El-Emam, Tom Gee, Susan G. Evans, Mojib Javadi, Francis Jeanson, Shannon Lefaivre, Kristen Lutz, F. Chris MacPhee, Jordan Mikkelsen, Tom Mikkelsen, Nicholas Mirotchnick, Tanya Schmah, Christa M. Studzinski, Donald T. Stuss, Elizabeth Theriault, Kenneth R. Evans. Brain-CODE: A Secure Neuroinformatics Platform for Management, Federation, Sharing and Analysis of Multi-Dimensional Neuroscience Data. Front. Neuroinform., 23 May 2018. <https://doi.org/10.3389/fninf.2018.00028>. PMID: 29875648
6. Rotenberg David J., Chang Qing, Potapova Natalia, Wang Andy, Hon Marcia, Sanches Marcos, Bogetic Nikola, Frias Nathan, Liu Tommy, Behan Brendan, El-Badrawi Rachad, Strother Stephen C., Evans Susan G., Mikkelsen Jordan, Gee Tom, Dong Fan, Arnott Stephen R., Laing Shuai, Dharsee Moyez, Vaccarino Anthony L., Javadi Mojib, Evans Kenneth R., Jankowicz Damian. The CAMH Neuroinformatics Platform: A Hospital-Focused Brain-CODE Implementation. Frontiers in Neuroinformatics. 2018. <https://doi.org/10.3389/fninf.2018.00077>
7. Brain-CODE: <https://www.braincode.ca>
8. Ontario Health Data Platform (OHDP): <https://ohdp.ca>
9. Public Health Analytics Environment (PHAE): [https://phae.indocpilot.io/](https://phae.indocpilot.io/login); <https://www.publichealthontario.ca/en/Data-and-Analysis/Infectious-Disease/COVID-19-Data-Surveillance>
10. Charité VRE: <https://vre.charite.de/>
11. EBRAINS Health Data Cloud: <https://www.healthdatacloud.eu/>
12. eBRAIN-Health: [https://ebrain-health.eu](https://ebrain-health.eu/) ; <https://cordis.europa.eu/project/id/101058516>
13. Lefaivre S, Behan B, Vaccarino A, Evans K, Dharsee M, Gee T, Dafnas C, Mikkelsen T, Theriault E. Big Data Needs Big Governance: Best Practices From Brain-CODE, the Ontario-Brain Institute's Neuroinformatics Platform. Front Genet. 2019 Mar 29;10:191. doi: 10.3389/fgene.2019.00191. PMID: 30984233; PMCID: PMC6450217.
14. Brendan Behan, Francis Jeanson, Heena Cheema, Derek Eng, Fatema Khimji, Anthony L. Vaccarino, Tom Gee, Susan G. Evans, F. Chris MacPhee, Fan Dong, Shahab Shahnazari, Alana Sparks, Emily Martens, Ryan Elliott, Bianca Lasalandra, Stephen R. Arnott, Stephen Strother, Mojib Javadi, Moyez Dharsee, Kenneth R. Evans, Kirk Nylen, Tom Mikkelsen. Reporting from the Trenches: Operating a Data Access Committee for international neuroscience data sharing. GA4GH 11^th^ Plenary, 20-22 September 2023.
15. Kyle Weber, Justina Zych, Brendan Behan, Francis Jeanson, Heena Cheema, Fatema Khimji, Kirk Nylen, Mojib Javadi, Alana Sparks, Ryan Elliott, Emily Martens, Stephen Arnott, Christina Yung, Anthony Vaccarino, Tom Mikkelsen. Brick by Brick: Building a Neuroinformatics-Centered Data Quality Framework. INCF Neufoinformatics Assembly, 23-25 September 2024.
16. Behan Brendan, Jeanson Francis, Cheema Heena, Eng Derek, Khimji Fatema, Vaccarino Anthony L., Gee Tom, Evans Susan G., MacPhee F. Chris, Dong Fan, Shahnazari Shahab, Sparks Alana, Martens Emily, Lasalandra Bianca, Arnott Stephen R., Strother Stephen C., Javadi Mojib, Dharsee Moyez, Evans Kenneth R., Nylen Kirk, Mikkelsen Tom. FAIR in action: Brain-CODE - A neuroscience data sharing platform to accelerate brain research. Frontiers in Neuroinformatics, 2023. DOI=10.3389/fninf.2023.1158378.
17. Behan, B., Sparks, A., Cheema, H., Naska, S., Jeanson, F., Basque, S., Ma, C., Chai-Onn, J., Javadi, M., Ho, M. and Mikkelsen, T. (2024) “Reporting on the establishment of a Privacy Preserving Record Linkage to Facilitate an Ongoing Crosswalk Between Research and Health Administrative Databases”, *International Journal of Population Data Science*, 9(5). doi: 10.23889/ijpds.v9i5.2760.

***Features***

| *Criteria* | *Details* |
| --- | --- |
| ***Security and Privacy (1)*** | The design, development and operation of Pilot are guided by Privacy by Design principles and a multi-layered, privacy-first approach to security to provide end-to-end protection across the data lifecycle. Specific measures include encryption at rest and in flight, multi-factor authentication, zone-based data isolation, role-based access control, extensive logging, continuous threat monitoring, vulnerability scanning and testing, verified backup and recovery protocols, regular threat and risk assessments, comprehensive data governance frameworks and policies, and security training and awareness programs.  Data stored on the platform are segregated into separate projects, each with its own unique membership and customizable roles and permissions. Within a project, the *Green Room* is an isolated zone for landing sensitive data and applying pseudonymization, validation, and data minimization prior to downstream processing and sharing. |
| ***Compliance and Regulatory Adherence (2, 3, 4, 5)*** | Indoc holds a SOC 2 Type II report validating the effectiveness of security and privacy controls for cloud deployments of Pilot. Specific deployments have undergone successful audits for compliance with GDPR and other regional privacy legislation, as well as numerous privacy impact assessments and threat risk assessments. Examples include:   - A customized instance of Pilot deployed at the [Charité](https://www.bihealth.org/en/translation/network/digital-medicine/bihcharite-virtual-research-environment) University Hospital in Berlin, Germany underwent an independent GDPR service readiness audit and was deemed suitable for providing data processing services in compliance with GDPR. - The [Ontario Health Data Platform](https://ohdp.ca/) (OHDP) underwent external TRAs and TVAs by the Ontario Government in compliance with PHIPA, PIPEDA and applicable regulations. - [Brain-CODE](https://www.braincode.ca/) has undergone multiple external PIAs, TRAs, and TVAs throughout its life cycle as the system grew and adapted to an ever-expanding range of programs and data sources. |
| **Interoperability and Extensibility (6, 7)** | Interoperability and integration with external systems is supported, including EHRs, PACS, LIS/LIMS, clinical data management systems. Data exchange (e.g., HL7 FHIR, DICOM) is facilitated by the Pilot CLI and API endpoints. |
| ***Data Quality and Integrity (8, 9, 10, 11)*** | Data validation and quality assessments can be performed using platform-managed pipelines. Brain-CODE, for instance, includes an extensive set of centrally orchestrated pipelines for assessing the integrity, standards compliance, and quality of imaging datasets. Similar pipelines have been deployed for assessing health system data (OHDP), and clinical assessment data (BD^2^).  All data files landing and moving within the platform are automatically tracked and indexed in the Pilot Data Catalogue. All subsequent data operations performed by users through the web portal or Command Line Interface (CLI), and operations performed though platform-managed pipelines, are also tracked and captured within annotated provenance and lineage records, generating a comprehensive audit trail at the file level across the data lifecycle. Furthermore, individual data files can be combined into a platform-managed *dataset* that can be structured, annotated, and versioned, providing an additional level of tracking at the dataset level. Through the web portal, users can visualize file lineage graphs and search the audit trail.  Data warehouse functionality can be used to combine heterogeneous structured data. In a typical workflow, data are extracted from an external data source and uploaded to a project Green Room through the web portal or CLI; ETL pipelines managed by the platform are automatically triggered to reshape and ingest the data into a pre-configured data warehouse; authorized users, pipelines, or applications can query the data warehouse and consume the data. In some Pilot deployments, integration of data from multiple sources requires a linkage process that matches different identifiers corresponding to the same subject. The matching logic can be a simple table lookup, or employ more complex matching algorithms, such as homomorphic encryption or non-deterministic methods (Behan et al., 2024). |
| ***Usability and Accessibility*** | Extensive documentation, user guides, knowledge bases, and step-by-step guides are available to users directly on the platform. Users can also submit support tickets.  Pilot is designed to align with the core principles of Web Content Accessibility Guidelines (WCAG). The UI is built on the Ant Design library, grounded in the React UI framework, and the design team places a strong emphasis on adhering to accessibility design standards. **I**nformation and UI components are presented in a perceptible manner. Text alternatives are provided for non-text content, comprehensive captions, and adaptable content to accommodate diverse user needs. Users can seamlessly interact with and navigate Pilot through keyboard accessibility and intuitive design. Sufficient time is provided for users to read and use content; content that may cause discomfort is avoided, and navigability and accessibility are prioritized. Pilot has been tested and used by multiple end users, ensuring that information and the operation of the UI are presented in an intuitive manner. The UI has been developed to be interpreted consistently across various user agents while conforming to evolving coding and technical standards and appropriate labeling of elements. |
| ***Scalability and Performance (1, 2, 3)*** | Pilot has been deployed in multiple large-scale programs. OHDP, for example, manages data from 15 million patients from many sources within the provincial health system. PHAE holds whole genome data and currently processes and manages over 50 TB of data and hundreds of millions of files. Similarly, large-scale master observational studies such as those supported by Brain-CODE similarly manage over 40 TB of data, spanning hundreds of millions of files, including processing of imaging data from thousands of participants.  Continuous monitoring of resource utilization and performance is conducted through the collection and analysis of metrics from VMs and containers in real time, such as CPU, memory, disk, network, and application metrics. This monitoring helps to identify bottlenecks, errors, or anomalies that affect platform performance, and adjust resource allocation accordingly. The platform can support increases in concurrent users and data volumes by scaling horizontally onto additional computing resources. This ensures consistent performance for all users even when there is a burst of compute-intensive activities. For example, large concurrent uploads will minimally affect other users exploring and analyzing data on the platform. |
| ***Collaboration and Sharing Capabilities (1, 6, 13)*** | Pilot provides researchers with multiple collaboration and sharing features across the data lifecycle. The project Green Room offers a common space for initial landing and staging of data uploaded by researchers or extracted from institutional or external data sources. At this stage, researchers can request their data to be copied to the project Core zone, and project administrators can review, verify, act on and communicate the outcomes of these requests directly from the web portal. Pilot Workspace tools provide additional collaboration opportunities. Researchers can use built-in business intelligence tools to represent their data visually and share reports and dashboards with other project members. Jupyter notebooks enable researchers to share their analyses and visualizations. Workspace containers, remote desktops, and VMs provide a computing environment for researchers to collaboratively analyze, visualize and share data.  Through role-based access control (RBAC), each Pilot user is associated with a role and associated permissions that determine the data and features the user can access. In addition to built-in roles, project administrators can define custom roles for fine-grained data access. In addition, Pilot employs a data zone architecture where each zone has its own data access rules. For example, users with the Collaborator role in a project can only upload data and download their own data from the project Green Room but are unable to access data from other users or zones. |
| ***Cost and Sustainability (1, 5, 14)*** | Cloud deployments generally offer cost efficiencies through shared engineering and infrastructure, and on-demand scaling. On-premises deployment can also be sustainable for clients wishing to leverage institutional infrastructure. |
| ***Ethical Considerations (12)*** | Pilot can be deployed with the ability to conduct extensive tracking of informed consent to appropriately control secondary use of data in compliance with research participant consent. For example, Brain-CODE includes a participant registry application used by study coordinators to capture fine-grained data sharing permissions granted across all informed consent form versions completed by study participants, and to capture data sharing approval instructions provided by institutional ethics review boards. This information is used in downstream applications to control which participant data can be included within open or controlled data releases (Lefaivre et al., 2019). |
| ***Innovation and Adaptability*** | The software architecture provides scalability, resiliency, and portability on a variety of computing environments including cloud, on-premises and hybrid deployments. The platform can integrate/federate with virtualization systems, identity providers, external databases and repositories, and tools for data capture, business intelligence, and analysis. |

***References***

1. Vaccarino AL, Dharsee M, Strother S, Aldridge D, Arnott SR, Behan B, Dafnas C, Dong F, Edgecombe K, El-Badrawi R, El-Emam K, Gee T, Evans SG, Javadi M, Jeanson F, Lefaivre S, Lutz K, MacPhee FC, Mikkelsen J, Mikkelsen T, Mirotchnick N, Schmah T, Studzinski CM, Stuss DT, Theriault E, Evans KR. Brain-CODE: A Secure Neuroinformatics Platform for Management, Federation, Sharing and Analysis of Multi-Dimensional Neuroscience Data. Front Neuroinform. 2018 May 23;12:28. doi: 10.3389/fninf.2018.00028. PMID: 29875648; PMCID: PMC5974337.
2. Ontario Health Data Platform (OHDP): <https://ohdp.ca>
3. Public Health Analytics Environment (PHAE): [https://phae.indocpilot.io/](https://phae.indocpilot.io/login); <https://www.publichealthontario.ca/en/Data-and-Analysis/Infectious-Disease/COVID-19-Data-Surveillance>
4. Charité VRE: <https://vre.charite.de/>
5. HDC: <https://www.healthdatacloud.eu/>
6. Behan B, Jeanson F, Cheema H, Eng D, Khimji F, Vaccarino AL, Gee T, Evans SG, MacPhee FC, Dong F, Shahnazari S, Sparks A, Martens E, Lasalandra B, Arnott SR, Strother SC, Javadi M, Dharsee M, Evans KR, Nylen K, Mikkelsen T. FAIR in action: Brain-CODE - A neuroscience data sharing platform to accelerate brain research. Front Neuroinform. 2023 May 18;17:1158378. doi: 10.3389/fninf.2023.1158378. PMID: 37274750; PMCID: PMC10233014.
7. Behan, B., Gee, T., Evans, S. G., Dharsee, M., Evans, K., Azimaee, M., Ross, K., Cartagena, R., Victor, C., Green-Noble, L., Mikkelsen, T. and Nylen, K. (2020) “Using A Privacy Preserving Record Linkage to Facilitate an Ongoing Crosswalk Between Research and Health Administrative Databases”, *International Journal of Population Data Science*, 5(5). doi: 10.23889/ijpds.v5i5.1630.
8. An overview of the quality assurance and quality control of magnetic resonance imaging data for the Ontario Neurodegenerative Disease Research Initiative (ONDRI): pipeline … CJM Scott, SR Arnott, A Chemparathy, F Dong, I Solovey, T Gee, ... ioRxiv, 2020.01. 10.896415
9. Theyers AE, Zamyadi M, O'Reilly M, et al. Multisite Comparison of MRI Defacing Software Across Multiple Cohorts. Front Psychiatry. 2021;12:617997. Published 2021 Feb 24. doi:10.3389/fpsyt.2021.617997
10. Liang S, Beaton D, Arnott SR, et al. Magnetic Resonance Imaging Sequence Identification Using a Metadata Learning Approach. Front Neuroinform. 2021;15:622951. Published 2021 Nov 17. doi:10.3389/fninf.2021.622951
11. Kayvanrad A, Arnott SR, Churchill N, Hassel S, Chemparathy A, Dong F, Zamyadi M, Gee T, Bartha R, Black SE, Lawrence-Dewar JM, Scott CJM, Symons S, Davis AD, Hall GB, Harris J, Lobaugh NJ, MacQueen G, Woo C, Strother S; ONDRI Founding Investigators; CAN-BIND Investigators. Resting state fMRI scanner instabilities revealed by longitudinal phantom scans in a multi-center study. Neuroimage. 2021 Aug 15;237:118197. doi: 10.1016/j.neuroimage.2021.118197. Epub 2021 May 21. PMID: 34029737.
12. Lefaivre S, Behan B, Vaccarino A, Evans K, Dharsee M, Gee T, Dafnas C, Mikkelsen T, Theriault E. Big Data Needs Big Governance: Best Practices From Brain-CODE, the Ontario-Brain Institute's Neuroinformatics Platform. Front Genet. 2019 Mar 29;10:191. doi: 10.3389/fgene.2019.00191. PMID: 30984233; PMCID: PMC6450217.
13. S. Pollentier, A. Vaccarino, Data integration and sharing – easier said than done? From brain-CODE to a pilot ecosystem solution, Neuroscience Applied, Volume 1, Supplement 2, 2022, 100365, ISSN 2772-4085, <https://doi.org/10.1016/j.nsa.2022.100365>.
14. BD2 – Breakthrough Discoveries for thriving with Bipolar Disorder. <https://www.bipolardiscoveries.org/>

***Matrix table for Pilot common challenges***

| **Category** | **Description** |
| --- | --- |
| **Federated Queries Challenges (1, 2, 3)** | Participant data are typically collected in disparate and disconnected systems, and integration of these data therefore requires a matching and de-duplication process to link same-patient records across systems. This typically involves the use of sensitive personal identifiers, such as a hospital record number or health insurance plan number, to generate and assign a pseudonymized global identifier to matched records, while securely maintaining the mapping to the personal identifier. Pilot deployments ensure that this sensitive information is stored and processed in a secure environment with restricted access (i.e., within isolated Green Rooms), and include pipelines that implement privacy-preserving methods such homomorphic encryption (Behan et al., 2019) to enable de-duplication, and linkage with health system data and data from other sources.  Federation or integration of data from multiple institutions, data sources, and modalities is accomplished in deployments such as Brain-CODE and BD^2^ through the deployment of data ingestion and ETL pipelines coupled with a central data warehouse with a common data model. This integration relies on effective data validation, standardization and harmonization processes implemented across the data lifecycle. |
| **Patient Privacy and Data Protection (4)** | Pilot is built with defense-in-depth to prevent and mitigate incidents or malicious activity. This is implemented via overlapping layers of administrative, technical, and physical protection. All data on the platform can only be accessed and used for their intended purposes and by authorized users. Isolation between tenants and projects is strictly enforced. Even within a project, there are multiple data zones of isolation so that only project members with the necessary permissions can access protected data. Role-based access control provides consistent authorization across all platform components. All user interactions with the platform take place via the Pilot web application or the command line interface, which mediate communication with underlying storage structures and services. Data is always encrypted both at rest and in flight. Single sign-on (SSO) is enabled across all platform components, complete with multi-factor authentication (MFA). |
| **Organizational Policies** | Pilot is designed with the appropriate flexibility to manage the full range of Privacy, Security, and Organizational requirements associated with various types of medical and health research. These requirements can be very different depending on the use case. Health System requirements are associated with government security needs, while international cohort studies need to address regulatory requirements across jurisdictions. Pilot was built with this in mind and has been implemented in multiple countries, multiple municipal, state, and provincial jurisdictions, within hospitals, and in both on premise and cloud implementations.  For each of these use cases Indoc worked closely with the users and appropriate privacy and security officials to ensure the appropriate policies and procedures were in place for the specific application, with generally applied policies and procedures utilized wherever possible. |
| **Data Transformation requirements (2, 5, 6, 7)** | Common data models within a centralized data warehouse is generally deployed (depending on the use case requirements), with standard data ingestion/ETL pipelines. This is particularly important in large scale master observational trial designs such as used by ONDRI, CAN-BIND, POND, BD^2^ and others. Other models have been widely deployed for mapping within health system data sets. In many cases Indoc conducts the mapping as part of their support service to research programs, while in other cases the programs themselves manage these sorts of activities. Another common form of transformation is the conversion of DICOM imaging data to standardized formats (e.g., to NIfTI, BIDS) that facilitate quality assessment, data processing and integration. |
| **Installation and Maintenance** | Data platforms can be complex to install, maintain, and develop, particularly if the requirements of a given institution or research group are idiosyncratic, as they often are. In simple use cases where the organization has strong technical and IT teams and resources, and where the requirements are clearly defined and heavily supported by the organization, the installation and deployment will obviously be much easier. In cases where there is minimal support from the organization, where the needs are highly idiosyncratic, and where the requirements are only loosely defined, the installation and deployment will be much more complex. That said, Pilot has been successfully deployed in a great many different jurisdictions, situational complexities, and with the full range of support needs. |
| **Secure Deployment** | Multi-factor authentication (MFA) is supported to verify user identity. It can be enabled at a per-user level and is compatible with time-based one-time password (TOTP) authenticators. Administrator access to back-end infrastructure and systems is facilitated via controlled interfaces. These interfaces are based on the underlying deployment method (cloud environment, on-premises infrastructure, or hybrid configuration) and may include a dedicated virtual private network (VPN), RBAC hypervisor controls, and cloud-hypervisor controls such as Azure Bastion.  User access activities are centrally aggregated into a centralized logging system which is integrated with a Security Information and Event Management (SIEM) solution. Dataset and schema events such as creation, updates, downloads, releases, and deletions are centralized. In combination with streaming event data from Kafka, this permits a high degree of security intelligence and business analytics.  Network security is implemented throughout the Pilot deployment environment. Web-facing applications are protected via firewall, load balancing, and proxy appliances against external threats. All internal calls are routed via an API gateway to orchestrate microservices and to control API traffic. Network segregation occurs at a granular level to isolate all containers via network security groups and namespaces.  Backup and restoration services leverage the underlying deployment infrastructure to mitigate data loss and meet recovery point objectives. Pilot may be further integrated with distributed on-premises, hybrid, or cloud infrastructure to provide high availability services and minimize recovery time objectives. |
| **Understanding User Queries (2)** | Pilot incorporates data warehousing and dashboarding capabilities through integration of the PostgreSQL relational database and business intelligence (BI) solutions, such as Apache Superset. Extraction-transformation-loading (ETL) pipelines can be deployed and automated in Pilot to ingest source data into the database. Users can then create dashboards to visualize the data and can also query the database directly using BI tools, SQL interfaces, or other query tools of their choice. These dashboards and queries can retrieve data in real-time, reflecting the actual data in the database at the time of retrieval. Similar dashboards are available to researchers who wish to explore the contents of open and controlled data releases, and to request access to the data. |
| **Informatics and User Experience** | Pilot is designed to support a broad range of user personas. A variety of built-in tools are available in the web portal, including computational notebooks, query and dashboarding tools, and containers and remote desktop where additional tools can be installed. Users can perform advanced tasks using the Pilot CLI within notebooks and remote desktops, such as uploading data files, accessing project data, annotating data lineage information, and storing derived data. |
| **Complexity of Software** | Pilot’s architecture provides scalability, resiliency, and portability on a variety of computing environments including cloud, on-premises and hybrid deployments. Platform components are developed, deployed, and scaled using containerization and centralized orchestration. The complexity of this architecture is opaque to the end user.  The platform can integrate or federate with third party solutions, including virtualization systems, identity providers, external databases and repositories, and tools for data capture, business intelligence, and analysis. The platform can also be extended with platform-orchestrated pipelines to perform data-driven processing and analysis. It is designed to support a broad range of application scenarios and is not limited to specific data types or research areas. |
| **Incremental Updating Limitations (2)** | Pilot incorporates data integration functionality to aggregate and integrate heterogeneous structured data. In most deployments, automated ETL pipelines are triggered on a daily schedule to fully refresh the data warehouse with up-to-date data. This update method usually addresses most use cases, but some deployments have implemented incremental updates instead of a full refresh. Each approach has its advantages and limitations, and the platform does not impose a particular method, so that the choice is guided by each specific use case. |
| **Standardized Vocabularies and Flexibility (8)** | At a high level, Pilot implements and promotes the FAIR principles of making data findable, accessible, interoperable, and reusable. Pilot supports annotation of research data with rich metadata, adopts ontologies and standardized schemas where applicable, and enables the data to be searchable and retrievable for downstream research, irrespective of the source. Users can attach tags and file attributes to data files and folders. Tags are descriptive keywords or labels that facilitate file management, workflow automation, organization, discovery, and collaboration on the platform. File attributes are a collection of pre-configured key-value pairs that can be used to encourage or enforce standardized annotation of data during the data upload process. Pilot also supports the concept of a platform-managed dataset, which is a set of files combined into a desired folder structure that can be annotated and versioned.  A dataset can be easily annotated with keyword tags which can serve to make the data more findable and discoverable. Datasets also support more complex and structured annotation through standard or custom metadata schemas. For example, the openMINDS schema developed by the Human Brain Project for the annotation of neuroimaging data, or the Data Tagging Suite (DATS) schema for the annotation of scientific datasets, have been configured in some deployments. There is also built-in support for common data structures, such as the Brain Imaging Data Structure (BIDS) for neuroscience data, which includes schema validation, and automated extraction and indexing of metadata. |

***References***

1. Behan, B., Gee, T., Evans, S. G., Dharsee, M., Evans, K., Azimaee, M., Ross, K., Cartagena, R., Victor, C., Green-Noble, L., Mikkelsen, T. and Nylen, K. (2020) “Using A Privacy Preserving Record Linkage to Facilitate an Ongoing Crosswalk Between Research and Health Administrative Databases”, *International Journal of Population Data Science*, 5(5). doi: 10.23889/ijpds.v5i5.1630.
2. Anthony L. Vaccarino, Moyez Dharsee, Stephen Strother, Don Aldridge, Stephen R. Arnott, Brendan Behan, Costas Dafnas, Fan Dong, Kenneth Edgecombe, Rachad El-Badrawi, Khaled El-Emam, Tom Gee, Susan G. Evans, Mojib Javadi, Francis Jeanson, Shannon Lefaivre, Kristen Lutz, F. Chris MacPhee, Jordan Mikkelsen, Tom Mikkelsen, Nicholas Mirotchnick, Tanya Schmah, Christa M. Studzinski, Donald T. Stuss, Elizabeth Theriault, Kenneth R. Evans. Brain-CODE: A Secure Neuroinformatics Platform for Management, Federation, Sharing and Analysis of Multi-Dimensional Neuroscience Data. Front. Neuroinform., 23 May 2018 | https://doi.org/10.3389/fninf.2018.00028. PMID: 29875648
3. Vaccarino AL, Beaton D, Black SE, et al. Common Data Elements to Facilitate Sharing and Re-use of Participant-Level Data: Assessment of Psychiatric Comorbidity Across Brain Disorders. Front Psychiatry. 2022;13:816465. Published 2022 Feb 7. doi:10.3389/fpsyt.2022.816465
4. Lefaivre S, Behan B, Vaccarino A, Evans K, Dharsee M, Gee T, Dafnas C, Mikkelsen T, Theriault E. Big Data Needs Big Governance: Best Practices From Brain-CODE, the Ontario-Brain Institute's Neuroinformatics Platform. Front Genet. 2019 Mar 29;10:191. doi: 10.3389/fgene.2019.00191. PMID: 30984233; PMCID: PMC6450217.
5. An overview of the quality assurance and quality control of magnetic resonance imaging data for the Ontario Neurodegenerative Disease Research Initiative (ONDRI): pipeline … CJM Scott, SR Arnott, A Chemparathy, F Dong, I Solovey, T Gee, ... ioRxiv, 2020.01. 10.896415
6. Kayvanrad A, Arnott SR, Churchill N, Hassel S, Chemparathy A, Dong F, Zamyadi M, Gee T, Bartha R, Black SE, Lawrence-Dewar JM, Scott CJM, Symons S, Davis AD, Hall GB, Harris J, Lobaugh NJ, MacQueen G, Woo C, Strother S; ONDRI Founding Investigators; CAN-BIND Investigators. Resting state fMRI scanner instabilities revealed by longitudinal phantom scans in a multi-center study. Neuroimage. 2021 Aug 15;237:118197. doi: 10.1016/j.neuroimage.2021.118197. Epub 2021 May 21. PMID: 34029737.
7. Farzan F, Atluri S, Frehlich M, Dhami P, Kleffner K, Price R, Lam RW, Frey BN, Milev R, Ravindran A, McAndrews MP, Wong W, Blumberger D, Daskalakis ZJ, Vila-Rodriguez F, Alonso E, Brenner CA, Liotti M, Dharsee M, Arnott SR, Evans KR, Rotzinger S, Kennedy SH. Standardization of electroencephalography for multi-site, multi-platform and multi-investigator studies: insights from the canadian biomarker integration network in depression. Sci Rep. 2017 Aug 7;7(1):7473. doi: 10.1038/s41598-017-07613-x. PMID: 28785082; PMCID: PMC5547036.
8. Yung C, Dharsee M, Dong F*,* Evans K, Gilbert Evans S, Gee T, Javadi M, MacPhee C, Pollentier S, Shahnazari S*.* Pilot: Making research data FAIR. *F1000Research* 2022, **11**:1397; <https://doi.org/10.7490/f1000research.1119237.1>.

***Data Modalities Supported by Pilot***

Usually, clinical research data platforms are designed to integrate and manage a wide range of data modalities to support biomedical research. The primary data modalities used so far in

Pilot include:

| **Category** | **Data Modality** | **Description** |
| --- | --- | --- |
| **Clinical Data** | Electronic Health Records (EHRs) | Structured data (demographics, event/treatment metadata, clinical assessments, clinical labs) and unstructured data (e.g., waveform data). |
|  | Hospital Administrative Data | Our platforms are being used to manage the full range of administrative data, including ambulatory care, health insurance claims, hospital discharge data, mental health services, drug benefits claims, triage data, laboratory tests, vaccine administration, public health cases, prescription for narcotics, etc. Within the OHDP platform we manage data from 15 million patients. |
| **Genomic Data (1)** | Genomic Sequences (1) | Our platforms are used to manage most types of genomic data. Data are derived from:   - Clinical testing (e.g. within the PHAE public health platform we manage whole genomes of SARS-CoV2 to support variant surveillance reporting for the Province of Ontario. - Clinical genetics, as within the Virtual Laboratory Network - Whole genome and exome sequencing conducted within large scale academic research projects |
|  | Genotype Data (2) | Our platforms have been used to support research based on SNP, CNV, DNA methylation |
|  | Gene Expression Data (2, 3, 4, 5, 29) | All forms of gene expression data are supported, including Microarray, RNA-seq, mRNA, miRNA. |
| **Imaging Data** | Radiology Images (2, 6, 7, 8, 9) | Many of the large-scale research programs that Pilot supports incorporate one or more types of imaging, including MRI, fMRI, DWI, MEG, EEG. We routinely are involved in curation of these data, including defacing of brain images. |
|  | Pathology Images (32) | All supported in Pilot. |
| **Phenotypic Data** | Disease Phenotypes (10, 11, 12, 13, 14) | Most of the large research programs we support are deep phenotyping cohort projects. Numerous systems are supported, including ICD, WHO, and others. |
|  | Clinical Outcomes (15, 16, 17, 18, 19) | Again, most of the programs on Pilot conduct extensive clinical outcomes testing and these data are captured and or managed on the platform. Some examples referenced below. |
| **Medication Data** | Prescription Records (2, 30) | Routinely managed as part of clinical trials, public health projects, and cohort studies. Some examples are referenced above. |
|  | Medication Adherence / Compliance | As above, routine part of medical research programs. |
| **Laboratory Data** | Lab Test Results (2, 30) | As above, all deep phenotyping projects include measures of these types and are currently managed on Pilot. Laboratory test data from health system data sets are also managed on Pilot (e.g. OHDP) |
| **Survey Data** | Questionnaires and Surveys (14, 15, 20) | All supported on Pilot. |
|  | Patient-Reported Outcomes (2, 12, 21, 32, 33) | All supported on Pilot. |
| **Biomarker Data** | Proteomics (2, 22, 23, 24, 25) | All supported on Pilot. |
|  | Metabolomics | All supported on Pilot. |
| **Environmental Data** | Lifestyle Factors (2, 32, 33) | All supported on Pilot. |
|  | Environmental Exposures | All supported on Pilot. |
| **Socioeconomic Data** | Social Determinants of Health (2, 30, 32, 33) | All supported on Pilot. |
| **Family History Data** | Genetic Risk Factors (2, 32, 33) | All supported on Pilot. |
| **Longitudinal Data** | Time-Series Data (2, 32, 33) | All supported on Pilot. |
| **Behavioral Data** | Behavioral Assessments (2, 11, 12, 13, 26, 30, 32, 33) | All supported on Pilot. |
|  | Transcriptomics (2, 3, 4, 5) | All supported in Pilot (same as Gene Expression Data row above) |
| **Pathway Data** | Biological Pathways (23, 27, 34) | Pilot deployments have supported the storage and analysis of biological pathway datasets. |
|  | Interaction Networks (23, 28, 34) | Pilot deployments have supported the storage and analysis of Interaction network datasets. |

***References***

1. SARS-CoV-2 Genomic Surveillance in Ontario reporting from Piot-based PHAE platform: <https://www.publichealthontario.ca/en/Data-and-Analysis/Infectious-Disease/COVID-19-Data-Surveillance>
2. Lam RW, Milev R, Rotzinger S, Andreazza AC, Blier P, Brenner C, Daskalakis ZJ, Dharsee M, Downar J, Evans KR, Farzan F, Foster JA, Frey BN, Geraci J, Giacobbe P, Feilotter HE, Hall GB, Harkness KL, Hassel S, Ismail Z, Leri F, Liotti M, MacQueen GM, McAndrews MP, Minuzzi L, Müller DJ, Parikh SV, Placenza FM, Quilty LC, Ravindran AV, Salomons TV, Soares CN, Strother SC, Turecki G, Vaccarino AL, Vila-Rodriguez F, Kennedy SH; CAN-BIND Investigator Team. Discovering biomarkers for antidepressant response: protocol from the Canadian biomarker integration network in depression (CAN-BIND) and clinical characteristics of the first patient cohort. BMC Psychiatry. 2016 Apr 16;16:105. doi:10.1186/s12888-016-0785-x. PubMed PMID: 27084692; PubMed Central PMCID: PMC4833905.
3. Kim HK, Tyryshkin K, Elmi N, Dharsee M, Evans KR, Good J, Javadi M, McCormack S, Vaccarino AL, Zhang X, Andreazza AC, Feilotter H. Plasma microRNA expression levels and their targeted pathways in patients with major depressive disorder who are responsive to duloxetine treatment. J Psychiatr Res. 2019 Mar;110:38-44. doi:10.1016/j.jpsychires.2018.12.007. Epub 2018 Dec 8. PubMed PMID: 30580082.
4. Juan Pablo Lopez, Laura M. Fiori , Cristiana Cruceanu, Rixing Lin, Benoit Labonte, Hannah M. Cates, Elizabeth A. Heller, Vincent Vialou, Stacy M. Ku, Christophe Gerald, Ming-Hu Han, Jane Foster, Benicio N. Frey, Claudio N. Soares, Daniel J. Müller, Faranak Farzan, Francesco Leri, Glenda M. MacQueen, Harriet Feilotter, Kathrin Tyryshkin, Kenneth R. Evans, Peter Giacobbe, Pierre Blier, Raymond W. Lam, Roumen Milev, Sagar V. Parikh, Susan Rotzinger, Steven C. Strother, Cathryn M. Lewis, Katherine J. Aitchison, Gayle M. Wittenberg, Naguib Mechawar, Eric J. Nestler, Rudolf Uher, Sidney H. Kennedy & Gustavo Turecki. MicroRNAs 146a/b-5 and 425-3p and 24-3p are markers of antidepressant response and regulate MAPK/Wnt-system genes. Nature Communications. 2017 May 22;8:15497. doi: 10.1038/ncomms15497. PMID: 28530238 DOI: [10.1038/ncomms15497](https://doi.org/10.1038/ncomms15497)
5. Khella HW, Butz H, Ding Q, Rotondo F, Evans KR, Kupchak P, Dharsee M, Latif A, Pasic MD, Lianidou E, Bjarnason GA, Yousef GM. miR-221/222 Are Involved in Response to Sunitinib Treatment in Metastatic Renal Cell Carcinoma. Mol Ther. 2015 Nov;23(11):1748-58. doi: 10.1038/mt.2015.129. Epub 2015 Jul 23. PubMed PMID: 26201448; PubMed Central PMCID: PMC4817948.
6. Theyers AE, Zamyadi M, O'Reilly M, et al. Multisite Comparison of MRI Defacing Software Across Multiple Cohorts. Front Psychiatry. 2021;12:617997. Published 2021 Feb 24. doi:10.3389/fpsyt.2021.617997
7. Liang S, Beaton D, Arnott SR, et al. Magnetic Resonance Imaging Sequence Identification Using a Metadata Learning Approach. Front Neuroinform. 2021;15:622951. Published 2021 Nov 17. doi:10.3389/fninf.2021.622951
8. An overview of the quality assurance and quality control of magnetic resonance imaging data for the Ontario Neurodegenerative Disease Research Initiative (ONDRI): pipeline … CJM Scott, SR Arnott, A Chemparathy, F Dong, I Solovey, T Gee, ... ioRxiv, 2020.01. 10.896415
9. Faranak Farzan, Sravya Atluri, Matthew Frehlich, Paul Dhami, Killian Kleffner, Rae Price, Raymond W. Lam, Benicio N. Frey, Roumen Milev, Arun Ravindran, Mary Pat McAndrews, Willy Wong, Daniel Blumberger, Zafiris J Daskalakis, Fidel Vila-Rodriguez, Esther Alonso, Colleen A. Brenner, Mario Liotti, Moyez Dharsee, Stephen R. Arnott, Kenneth R. Evans, Susan Rotzinger, Sidney H. Kennedy. Standardization of Electroencephalography for Multi-Site, Multi-Platform and Multi-Investigator Studies: Insights from the Canadian Biomarker Integration Network in Depression. Scientific Reports. 2017 Aug 7;7(1):7473. doi: 10.1038/s41598-017-07613-x.
10. Vaccarino AL, Black SE, Gilbert Evans S, et al. Rasch analyses of the Quick Inventory of Depressive Symptomatology Self-Report in neurodegenerative and major depressive disorders. Front Psychiatry. 2023;14:1154519. Published 2023 Jun 2. doi:10.3389/fpsyt.2023.1154519
11. McPhee PG, Vaccarino AL, Naska S, et al. Harmonizing data on correlates of sleep in children within and across neurodevelopmental disorders: lessons learned from an Ontario Brain Institute cross-program collaboration. Front Neuroinform. 2024;18:1385526. Published 2024 May 17. doi:10.3389/fninf.2024.1385526
12. McPhee, P. G., Georgiades, S., Andrade, A., Corkum, P. V., Vaccarino, A. L., Cheema, H., et al. (2023). Sleep, internalizing symptoms, and health-related quality of life in children with neurodevelopmental disorders: a cross-sectional analysis of cohort data from three research programs in Canada. Front. Sleep 2:1224610. doi: 10.3389/frsle.2023.1224610
13. Vaccarino AL, Beaton D, Black SE, et al. Common Data Elements to Facilitate Sharing and Re-use of Participant-Level Data: Assessment of Psychiatric Comorbidity Across Brain Disorders. Front Psychiatry. 2022;13:816465. Published 2022 Feb 7. doi:10.3389/fpsyt.2022.816465
14. Sparks A, Gilbert Evans S, Javadi M, et al. Assessment of anxiety in children with neurodevelopment disorders: Rasch analysis of the Spence Children's Anxiety Scale. Front Psychiatry. 2024;15:1240357. Published 2024 Apr 29. doi:10.3389/fpsyt.2024.1240357
15. Vaccarino AL, Black SE, Gilbert Evans S, et al. Rasch analyses of the Quick Inventory of Depressive Symptomatology Self-Report in neurodegenerative and major depressive disorders. Front Psychiatry. 2023;14:1154519. Published 2023 Jun 2. doi:10.3389/fpsyt.2023.1154519
16. Muscedere J, Afilalo J, Araujo de Carvalho I, Cesari M, Clegg A, Eriksen HE, Evans KR, Heckman G, Hirdes JP, Kim PM, Laffon B, Lynn J, Martin F, Prorok JC, Rockwood K, Rodrigues Mañas L, Rolfson D, Shaw G, Shea B, Sinha S, Theou O, Tugwell P, Valdiglesias V, Vellas B, Veronese N, Wallace LMK, Williamson PR. Moving Towards Common Data Elements and Core Outcome Measures in Frailty Research. J Frailty Aging. 2020;9(1):14-22. doi: 10.14283/jfa.2019.43. PMID: 32150209.
17. Kennedy SH, Lam RW, Rotzinger S, Milev RV, Blier P, Downar J, Evans KR, Farzan F, Foster JA, Frey BN, Giacobbe P, Hall GB, Harkness KL, Hassel S, Ismail Z, Leri F, McInerney S, MacQueen GM, Minuzzi L, Müller DJ, Parikh SV, Placenza FM, Quilty LC, Ravindran AV, Sassi RB, Soares CN, Strother SC, Turecki G, Vaccarino AL, Vila-Rodriguez F, Yu J, Uher R; CAN-BIND Investigator Team. Symptomatic and Functional Outcomes and Early Prediction of Response to Escitalopram Monotherapy and Sequential Adjunctive Aripiprazole Therapy in Patients With Major Depressive Disorder: A CAN-BIND-1 Report. J Clin Psychiatry. 2019 Feb 5;80(2). pii:18m12202. doi: 10.4088/JCP.18m12202. PubMed PMID: 30840787.
18. Lam RW, Milev R, Rotzinger S, Andreazza AC, Blier P, Brenner C, Daskalakis ZJ, Dharsee M, Downar J, Evans KR, Farzan F, Foster JA, Frey BN, Geraci J, Giacobbe P, Feilotter HE, Hall GB, Harkness KL, Hassel S, Ismail Z, Leri F, Liotti M, MacQueen GM, McAndrews MP, Minuzzi L, Müller DJ, Parikh SV, Placenza FM, Quilty LC, Ravindran AV, Salomons TV, Soares CN, Strother SC, Turecki G, Vaccarino AL, Vila-Rodriguez F, Kennedy SH; CAN-BIND Investigator Team. Discovering biomarkers for antidepressant response: protocol from the Canadian biomarker integration network in depression (CAN-BIND) and clinical characteristics of the first patient cohort. BMC Psychiatry. 2016 Apr 16;16:105. doi:10.1186/s12888-016-0785-x. PubMed PMID: 27084692; PubMed Central PMCID: PMC4833905.
19. Kennedy SH, Downar J, Evans KR, Feilotter H, Lam RW, MacQueen GM, Milev R, Parikh SV, Rotzinger S, Soares C. The Canadian Biomarker Integration Network in Depression (CAN-BIND): advances in response prediction. Curr Pharm Des. 2012;18(36):5976-89. PubMed PMID: 22681173.
20. Vaccarino AL, Kalali AH, Blier P, Gilbert Evans S, Engelhardt N, Foster JA, Frey BN, Greist JH, Kobak KA, Lam RW, MacQueen G, Milev R, Müller DJ, Parikh SV, Placenza FM, Rizvi SJ, Rotzinger S, Sheehan DV, Sills T, Soares CN, Turecki G, Uher R, Williams JBW, Kennedy SH, Evans KR. THE DEPRESSION INVENTORY DEVELOPMENT SCALE: Assessment of Psychometric Properties Using Classical and Modern Measurement Theory in a CAN-BIND Trial. Innov Clin Neurosci. 2020 Jul 1;17(7-9):30-40. PMID: 33520402; PMCID: PMC7839654.
21. Vaccarino AL, Evans SG, Javadi M, et al. Quality of life across neurodegenerative and major depressive disorders, European College of Neuropsychopharmacology, Milan. Italy, 2024
22. Yang Y, Chen Y, Saha MN, Chen J, Evans K, Qiu L, Reece D, Chen GA, Chang H. Targeting phospho-MARCKS overcomes drug-resistance and induces antitumor activity in preclinical models of multiple myeloma. Leukemia. 2015 Mar;29(3):715-26. doi: 10.1038/leu.2014.255. Epub 2014 Sep 2. PubMed PMID: 25179733.
23. Cawthorn TR, Moreno JC, Dharsee M, Tran-Thanh D, Ackloo S, Zhu PH, Sardana G, Chen J, Kupchak P, Jacks LM, Miller NA, Youngson BJ, Iakovlev V, Guidos CJ, Vallis KA, Evans KR, McCready D, Leong WL, Done SJ. Proteomic analyses reveal high expression of decorin and endoplasmin (HSP90B1) are associated with breast cancer metastasis and decreased survival. PLoS One. 2012;7(2):e30992. doi:10.1371/journal.pone.0030992. Epub 2012 Feb 20. PubMed PMID: 22363530; PubMed Central PMCID: PMC3282708.
24. Yang Y, Chen Y, Saha MN, Chen J, Evans K, Qiu L, Reece D, Chen GA, Chang H. Targeting phospho-MARCKS overcomes drug-resistance and induces antitumor activity in preclinical models of multiple myeloma. Leukemia. 2015 Mar;29(3):715-26. doi: 10.1038/leu.2014.255. Epub 2014 Sep 2. PubMed PMID: 25179733.
25. Merali Z, Gao MM, Bowes T, Chen J, Evans K, Kassner A. Neuroproteome Changes after Ischemia/Reperfusion Injury and Tissue Plasminogen Activator Administration in Rats: A Quantitative iTRAQ Proteomics Study. Wang X, ed.PLoS ONE. 2014;9(5):e98706. doi:10.1371/journal.pone.0098706.
26. Vaccarino AL, Sills T, Anderson KE, Borowsky B, Craufurd D, Giuliano J, Goodman L, Guttman M, Kupchak P, Ho AK, Paulsen JS, C Stout J, van Kammen DP, Evans K. Assessment of cognitive symptoms in prodromal and early huntington disease. PLoS Curr. 2011 Oct 25;3:RRN1250. PubMed PMID: 22120841; PubMed Central PMCID: PMC3201666.
27. Koti M, Gooding RJ, Nuin P, Haslehurst A, Crane C, Weberpals J, Childs T, Bryson P, Dharsee M, Evans K, Feilotter HE, Park PC, Squire JA. Identification of the IGF1/PI3K/NF κB/ERK gene signalling networks associated with chemotherapy resistance and treatment response in high-grade serous epithelial ovarian cancer. BMC Cancer. 2013 Nov 16;13:549. doi: 10.1186/1471-2407-13-549. PMID: 24237932; PMCID: PMC3840597.
28. Geraci J, Dharsee M, Nuin P, Haslehurst A, Koti M, Feilotter HE, Evans K. Exploring high dimensional data with Butterfly: a novel classification algorithm based on discrete dynamical systems. Bioinformatics. 2014 Mar 1;30(5):712-8. doi: 10.1093/bioinformatics/btt602. Epub 2013 Oct 21. PMID: 24149051.
29. Lichner Z, Fendler A, Saleh C, Nasser AN, Boles D, Al-Haddad S, Kupchak P, Dharsee M, Nuin PS, Evans KR, Jung K, Stephan C, Fleshner NE, Yousef GM. MicroRNA signature helps distinguish early from late biochemical failure in prostate cancer. Clin Chem. 2013 Nov;59(11):1595-603. doi: 10.1373/clinchem.2013.205450. Epub 2013 Aug 19. PMID: 23958847.
30. Anthony L. Vaccarino, Moyez Dharsee, Stephen Strother, Don Aldridge, Stephen R. Arnott, Brendan Behan, Costas Dafnas, Fan Dong, Kenneth Edgecombe, Rachad El-Badrawi, Khaled El-Emam, Tom Gee, Susan G. Evans, Mojib Javadi, Francis Jeanson, Shannon Lefaivre, Kristen Lutz, F. Chris MacPhee, Jordan Mikkelsen, Tom Mikkelsen, Nicholas Mirotchnick, Tanya Schmah, Christa M. Studzinski, Donald T. Stuss, Elizabeth Theriault, Kenneth R. Evans. Brain-CODE: A Secure Neuroinformatics Platform for Management, Federation, Sharing and Analysis of Multi-Dimensional Neuroscience Data. Front. Neuroinform., 23 May 2018 | https://doi.org/10.3389/fninf.2018.00028. PMID: 29875648
31. Charité VRE: <https://vre.charite.de/>
32. Farhan SM, Bartha R, Black SE, Corbett D, Finger E, Freedman M, Greenberg B, Grimes DA, Hegele RA, Hudson C, Kleinstiver PW, Lang AE, Masellis M, McIlroy WE, McLaughlin PM, Montero-Odasso M, Munoz DG, Munoz DP, Strother S, Swartz RH, Symons S, Tartaglia MC, Zinman L; ONDRI Investigators; Strong MJ. The Ontario Neurodegenerative Disease Research Initiative (ONDRI). Can J Neurol Sci. 2017 Mar;44(2):196-202. doi: 10.1017/cjn.2016.415. Epub 2016 Dec 22. PMID: 28003035.
33. Fehlings, D.L., Zarrei, M., Engchuan, W. *et al.* Comprehensive whole-genome sequence analyses provide insights into the genomic architecture of cerebral palsy. *Nat Genet* **56**, 585–594 (2024). <https://doi.org/10.1038/s41588-024-01686-x>
34. Rogers RS, Dharsee M, Ackloo S, Sivak JM, Flanagan JG. Proteomics analyses of human optic nerve head astrocytes following biomechanical strain. Mol Cell Proteomics. 2012 Feb;11(2):M111.012302. doi: 10.1074/mcp.M111.012302. Epub 2011 Nov 29. PMID: 22126795; PMCID: PMC3277762.

**Built-in Workflows and Analysis Tools**

**Workflow**

| **Feature** | **Description** |
| --- | --- |
| Patient Cohort Discovery (1, 2) | Data extracted from clinical sources can be ingested and integrated into a platform-managed data warehouse, enabling query and visualization. Cohort discovery dashboards accessible from the web portal can be implemented to visually discover and shape patient cohorts across research studies and generate data sets based on demographic, clinical, and other endpoints of interest. The same dashboard functionality is also used for visualizing data contained in open and controlled data releases, allowing researchers to generate and request access to subsets of data that meet specific cohort criteria. |
| Data Integration and Management (1, 3, 4) | Pilot includes the capability to deploy data import and ETL pipelines that can ingest data from multi-modal sources into a platform-managed data warehouse configured with a common data model, enabling researchers to perform queries and analyses on the integrated data. Examples include OHDP, Brain-CODE, and the BD^2^ data platform. |
| Ontology Management (5, 6, 7, 8, 9) | Pilot includes the ability to capture file-level and dataset-level metadata annotations based on built-in (e.g., DATS) or custom schemas encoded in a machine-interpretable format (JSON LD). Annotations can incorporate unique identifiers and can be indexed to easily retrieve data on the web portal or using APIs. Datasets encoded with standard ontology-based schemas (e.g., OMOP) or formatted with standard data structures (e.g., BIDS) are also supported, and data validators can be incorporated to verify compliance. Ontology stores or lookup services can also be incorporated as required.  Knowledge graph solutions can also be integrated with Pilot to enable advanced annotation and knowledge representation. For instance, the Pilot-based Health Data Cloud was integrated with the EBRAINS knowledge graph based on the openMINDS data model. |
| Data Extraction and Transformation (1, 3, 4, 8) | Any ETL workflow can be implemented and automated. Pilot integrates a generalized workflow management system that enables the definition and automation of ETL and other types of pipelines. These pipelines can be automatically triggered based on specified rules and events. ETL pipelines have been implemented in Pilot for various purposes, including ingestion of structured data (clinical, imaging, genomics, laboratory, wearables, mobile, etc.) into a central data warehouse, conversion of data formats (e.g., from DICOM to BIDS), quality control and validation, and others. Pilot deployments that implement ETL pipelines include BD^2^, Brain-CODE, Charité VRE, and OHDP. |
| Security and Privacy Management (1, 3, 4, 6, 10, 11) | Pilot incorporates several features that support data protection and privacy:   - Role-based access control, with the ability to define custom roles at the project level, allowing fine-grained control over the data and features available to users. - Zone architecture, including an isolated Green Room zone for data landing and staging, allowing authorized users to pseudonymize, validate, and curate data before they are shared with other members of a project in a separate Core zone. - De-identification pipelines, which can be configured and automated on data landing in the Green Room. Examples use cases include privacy-preserving pseudonymization of sensitive identifiers and defacing of full-face MR scans. - Privacy-protective ingress and egress: Deployments with highly sensitive data require privacy-protective and layered ingress and egress procedures and technology. This ensures that only approved files can be uploaded, and only approved research results can leave the platform, safeguarding the confidentiality and integrity of the data throughout the process, and provides comprehensive audit of all files on and off the platform, in addition to the proactive controls. - Data visitation isolation: Deployments with highly sensitive data include tailorable degrees of security and isolation, including fully secured “Data Visitation” mechanisms whereby all movement of files on or off the system are strictly limited or completely prevented, enabling the processing of fully identified data in secured and isolated workspaces. Data may only be visited (seen, processed, and analyzed) in the workspaces, but not copied or moved from the platform. - Privacy-preserving data linkage: Pilot deployments include the use of homomorphic cryptographic linkage and tokenization through deterministic and probabilistic methods, and the generation and maintenance of pseudonymized globally unique identifiers (GUIDs), to ensure data minimization and facilitate secure linkage. - Additional features and measures include encryption at rest and in flight, multi-factor authentication, extensive logging, continuous threat monitoring, vulnerability scanning and testing, verified backup and recovery protocols, regular threat and risk assessments, comprehensive data governance frameworks and policies, and security training and awareness programs. |

**References:**

1. Anthony L. Vaccarino, Moyez Dharsee, Stephen Strother, Don Aldridge, Stephen R. Arnott, Brendan Behan, Costas Dafnas, Fan Dong, Kenneth Edgecombe, Rachad El-Badrawi, Khaled El-Emam, Tom Gee, Susan G. Evans, Mojib Javadi, Francis Jeanson, Shannon Lefaivre, Kristen Lutz, F. Chris MacPhee, Jordan Mikkelsen, Tom Mikkelsen, Nicholas Mirotchnick, Tanya Schmah, Christa M. Studzinski, Donald T. Stuss, Elizabeth Theriault, Kenneth R. Evans. Brain-CODE: A Secure Neuroinformatics Platform for Management, Federation, Sharing and Analysis of Multi-Dimensional Neuroscience Data. Front. Neuroinform., 23 May 2018 | https://doi.org/10.3389/fninf.2018.00028. PMID: 29875648
2. Brain-CODE data releases: <https://www.braincode.ca/content/data-releases>
3. Breakthrough Discoveries for thriving with Bipolar Disorder (BD^2^): <https://www.bipolardiscoveries.org/>;
4. Ontario Health Data Platform (OHDP): <https://ohdp.ca>
5. Data Tagging Suite; <https://datatagsuite.github.io/docs/html/index.html>
6. EBRAINS Health Data Cloud: <https://www.healthdatacloud.eu/>
7. openMINDS data model: <https://ebrains.eu/news/new-openminds-metadata-models/>
8. Charité VRE: <https://vre.charite.de/>
9. Blue Brain Nexus: <https://bluebrainnexus.io>
10. Behan, B., Gee, T., Evans, S. G., Dharsee, M., Evans, K., Azimaee, M., Ross, K., Cartagena, R., Victor, C., Green-Noble, L., Mikkelsen, T. and Nylen, K. (2020) “Using A Privacy Preserving Record Linkage to Facilitate an Ongoing Crosswalk Between Research and Health Administrative Databases”, *International Journal of Population Data Science*, 5(5). doi: 10.23889/ijpds.v5i5.1630.
11. Lefaivre S, Behan B, Vaccarino A, Evans K, Dharsee M, Gee T, Dafnas C, Mikkelsen T, Theriault E. Big Data Needs Big Governance: Best Practices From Brain-CODE, the Ontario-Brain Institute's Neuroinformatics Platform. Front Genet. 2019 Mar 29;10:191. doi: 10.3389/fgene.2019.00191. PMID: 30984233; PMCID: PMC6450217.

**Analysis Tools**

| Query Interface (1, 2) | Data extracted from clinical sources can be harmonized and integrated within a platform-managed data warehouse with a common data model, thus enabling query and visualization using interactive dashboards on the web portal. |
| --- | --- |
| Timeline Viewer (1, 2) | Pilot-based platforms include data warehousing and interactive dashboard functionality which have been customized for a variety of purposes, including cohort discovery, data access requests, and participant-level timelines and other longitudinal data. |
| Statistics and Analytics (1, 3, 4) | Pilot includes built-in Jupyter notebooks that enable users to conduct basic statistical analyses. Pipelines can also be deployed to automate these analyses on uploaded data, or on data ingested in a central data warehouse. Users can also conduct these analyses using their preferred software tools within workspace containers or remote desktops that can be accessed directly from the web portal. |
| Plugin Framework (1, 4, 5) | Pilot’s architecture, command line interface, pipeline functionality, and API-first design enable the platform to be readily extended with new functionality and third-party integrations. |
| Natural Language Processing (NLP) | Pilot can readily incorporate NLP functionality as a native service, automated workflows, a web application, or a persistent container-based application. |
| Genomic Data Analysis (1) | Pilot has been used extensively to store, process, integrate and analyze genomic data, and other molecular data types. |
| Temporal Querying (1, 2) | Supported generically with built-in analytics tools (e.g., Apache Superset) to query and visualize event-based data. |
| Data Visualization (1, 2) | Built-in tools or third-party integrations support query and visualization, including customizable, interactive dashboards. Jupyter notebooks are also available for custom visualizations. Additional tools (e.g., specific to imaging or genomics data) can be deployed within containers and remote desktops. |
| Export and Reporting (1, 6) | Users can export data and generate reports using built-in tools or third-party integrations. Workflows can be deployed to convert data formats (e.g., imaging data from DICOM to NiFTi/BIDS format) and transmit reports data to external systems. |

***References***

1. Anthony L. Vaccarino, Moyez Dharsee, Stephen Strother, Don Aldridge, Stephen R. Arnott, Brendan Behan, Costas Dafnas, Fan Dong, Kenneth Edgecombe, Rachad El-Badrawi, Khaled El-Emam, Tom Gee, Susan G. Evans, Mojib Javadi, Francis Jeanson, Shannon Lefaivre, Kristen Lutz, F. Chris MacPhee, Jordan Mikkelsen, Tom Mikkelsen, Nicholas Mirotchnick, Tanya Schmah, Christa M. Studzinski, Donald T. Stuss, Elizabeth Theriault, Kenneth R. Evans. Brain-CODE: A Secure Neuroinformatics Platform for Management, Federation, Sharing and Analysis of Multi-Dimensional Neuroscience Data. Front. Neuroinform., 23 May 2018 | https://doi.org/10.3389/fninf.2018.00028. PMID: 29875648
2. Brain-CODE data releases: <https://www.braincode.ca/content/data-releases>
3. Breakthrough Discoveries for thriving with Bipolar Disorder (BD^2^): <https://www.bipolardiscoveries.org/>;
4. EBRAINS Health Data Cloud: <https://www.healthdatacloud.eu/>
5. Charité VRE: <https://vre.charite.de/vre/pages/resources>
6. <https://www.publichealthontario.ca/en/Data-and-Analysis/Infectious-Disease/COVID-19-Data-Surveillance>

| **Integration with Other Tools** | R / BioConductor and Python Integration (1, 4) | Pilot offers built-in workspace tools integrated with single sign-on and paired with Pilot’s command line interface to offer an array of analysis capabilities. Pilot includes JupyterHub to provide personal notebooks preconfigured with a data science environment including popular tools like Python and R. Users can also access remote desktops or terminals to install programing tools and libraries. |
| --- | --- | --- |
|  | Integration with Clinical Trial Management Systems (CTMS) (1, 3) | Pilot supports interfaces or workflows that integrate with clinical trial management systems. |
|  | Integration with Electronic Health Records (EHR) (2) | Pilot can integrate data extracted from hospital EHR systems. Imported data are typically loaded into a research data warehouse and integrated with other data types and modalities. |
|  | Integration with External Databases (5, 6, 7, 8) | Integration with external databases is supported. |
|  | Integration with REDCap for Research Capture in a Clinical Workflow (1, 9) | Indoc has extensive experience with the operation and integration of the REDCap electronic data capture software. Data are ingested using the REDCap API, validated with automated QC workflows, transformed to a common data model, and typically loaded it into a central database that end users can query to analyze multi-modal datasets. |

References:

1. Anthony L. Vaccarino, Moyez Dharsee, Stephen Strother, Don Aldridge, Stephen R. Arnott, Brendan Behan, Costas Dafnas, Fan Dong, Kenneth Edgecombe, Rachad El-Badrawi, Khaled El-Emam, Tom Gee, Susan G. Evans, Mojib Javadi, Francis Jeanson, Shannon Lefaivre, Kristen Lutz, F. Chris MacPhee, Jordan Mikkelsen, Tom Mikkelsen, Nicholas Mirotchnick, Tanya Schmah, Christa M. Studzinski, Donald T. Stuss, Elizabeth Theriault, Kenneth R. Evans. Brain-CODE: A Secure Neuroinformatics Platform for Management, Federation, Sharing and Analysis of Multi-Dimensional Neuroscience Data. Front. Neuroinform., 23 May 2018 | https://doi.org/10.3389/fninf.2018.00028. PMID: 29875648.
2. Brain-CODE data releases: <https://www.braincode.ca/content/data-releases>
3. Breakthrough Discoveries for thriving with Bipolar Disorder (BD^2^): <https://www.bipolardiscoveries.org/>
4. EBRAINS Health Data Cloud: <https://www.healthdatacloud.eu/>
5. <https://www.publichealthontario.ca/en/Data-and-Analysis/Infectious-Disease/COVID-19-Data-Surveillance>
6. Southwell A, Bronskill S, Gee T, Behan B, Evans SG, Mikkelsen T, Theriault E, Nylen K, Lefaivre S, Chong N, Azimaee M, Tusevljak N, Lee D, Swartz RH. Validating a novel deterministic privacy-preserving record linkage between administrative & clinical data: applications in stroke research. Int J Popul Data Sci. 2022 Nov 22;7(4):1755. doi: 10.23889/ijpds.v7i4.1755. PMID: 37152407; PMCID: PMC10161965.
7. Gee, T., Behan, B., Lefaivre, S., Azimaee, M., Dharsee, M., El Emam, K., Yang, J., Vaccarino, A., Evans, K., Victor, J. C. and Theriault, E. (2018) “Designing and Implementing a Privacy Preserving Record Linkage Protocol”, *International Journal of Population Data Science*, 3(4). doi: 10.23889/ijpds.v3i4.831.
8. Behan, B., Gee, T., Evans, S. G., Dharsee, M., Evans, K., Azimaee, M., Ross, K., Cartagena, R., Victor, C., Green-Noble, L., Mikkelsen, T. and Nylen, K. (2020) “Using A Privacy Preserving Record Linkage to Facilitate an Ongoing Crosswalk Between Research and Health Administrative Databases”, *International Journal of Population Data Science*, 5(5). doi: 10.23889/ijpds.v5i5.1630*.*
9. Charité VRE: <https://vre.charite.de/vre/pages/resources>

**Support for Semantic Integration**

Ontologies, terminologies and common data models are supported and implemented as per the requirements of the research programs that use the platform.

Pilot environments support standards-based harmonization and interoperability through the deployment of data warehouse and data federation designs based on the OMOP common data model (CDM), enabling import and export of OMOP datasets and conversion to and from OMOP of standard classifications and terminologies including ATC, ICD-10, LOINC, SNOMED-CT, MeDRA, and others.

Pilot allows users to attach tags and file attributes to data files and folders. Tags are descriptive keywords or labels that facilitate file management, workflow automation, organization, discovery, and collaboration on the platform. File attributes are a collection of pre-configured key-value pairs that can be used to encourage or enforce standardized annotation of data during the data upload process.

Using platform-managed datasets, research datasets can be organized, annotated, and versioned. A dataset can be tagged with keywords and annotated using standard or custom schemas. For example, the openMINDS schema developed by the Human Brain Project/EBRAINS for the annotation of neuroimaging data, or the Data Tagging Suite (DATS) schema for the annotation of scientific datasets, have been configured in some deployments. Custom support for scientific data structures (e.g., Brain Imaging Data Structure, BIDS) includes schema validation and automated metadata extraction and indexing.

These capabilities can be foundational for effective semantic integration and search based on pre-trained models and indexing schemes. Pilot has also been integrated with specific knowledge graph solutions (e.g., EBRAINS Knowledge Graph, Blue Brain Nexus).

1. References go here
