# Supplementary material for: Selecting medical research data platforms for translational biomedical research: a five-tier overview and requirement-weighted assessment framework
Source: Front Digit Health. 2026 Jun 17;8:1814015. doi: 10.3389/fdgth.2026.1814015 (PMC13319098; doi:10.3389/fdgth.2026.1814015)
Supplement: Supplementary file 14 [file Supplementaryfile14.docx]

*Here comes the “empty” form to add your information. All my comments are labeled in blue. They can be removed. Would be good if you chose another color for your input.*

***IQVIA Health Data Research Platform (HDRP)***

***Deployment and Usage****:*

**HDRP (Health Data Research Platform)** is an innovative, patient-centric research platform designed to maximize the utility of clinical data for secondary use. The solution is built upon three core pillars: clinical data integration, biobanking, and comprehensive support for clinical studies—from feasibility analysis and design to execution and evaluation.

HDRP is a highly integrative system, seamlessly interfacing with a wide range of healthcare solutions, including hospital information systems, laboratory systems, and specialized documentation systems. It supports a variety of common data communication standards such as HL7.v2, HL7-FHIR, and XML. Developed according to FAIR data principles, HDRP ensures the highest security standards, enabling the harmonization and aggregation of all clinical data, which is presented in the patient context.

In biobanking, HDRP offers extensive integration with various laboratory automation solutions, including liquid handling platforms, quantification devices, and automated storage systems. The built-in workflow engine (jBPM) allows the mapping and execution of interdisciplinary processes according to standard operating procedures (SOPs).

HDRP can be deployed as a standalone installation, in a networked environment with multi-master synchronization, or in conjunction with a trustee for full pseudonymization or anonymization. It combines the functionalities of a medical data warehouse, a study management system, a Clinical Trial Management System (CTMS), and an Electronic Data Capture (EDC) system into a single, powerful platform.

Typically, HDRP is installed on premises within the client’s IT infrastructure, and behind their firewalls. In these cases, the clients operate the system and own all data collected within. Some use cases demand deploying HDRP in the cloud. The solution is provider agnostic if system requirements are met.

Currently, IQVIA mainly supports clients from the University Hospital space with HDRP technology. This includes deployments at 32 of a total of 36 German University hospitals. HDRP has also been deployed across 10+ countries in Europe and North America. IQVIA has recently also been supporting more of its Pharma clients with HDRP-EDC (Electronic Data Capture) capabilities in several NIS (non-interventional studies) for data collection and analytics.

***References:***

[Bridging the gap: Leveraging telemedicine and IT infrastructure to connect outpatient oncology practices with specialized expert teams in the management of rare tumors.](https://pubmed.ncbi.nlm.nih.gov/39398892/)

Kasprzak J, Goering T, Berger-Thürmel K, Kratzer V, Prompinit W, Wichert SP, Leutner S, Langermann N, von Bergwelt-Baildon M, Heinemann V, Algül H, Zünkeler M, Nasseh D; TARGET Group *.Digit Health. 2024 Oct 9;10:20552076241272709. doi: 10.1177/20552076241272709. eCollection 2024 Jan-Dec.PMID: 39398892

[Linked Data Applications Through Ontology Based Data Access in Clinical Research.](https://pubmed.ncbi.nlm.nih.gov/28423769/)

Kock-Schoppenhauer AK, Kamann C, Ulrich H, Duhm-Harbeck P, Ingenerf J.Stud Health Technol Inform. 2017;235:131-135.PMID: 28423769

***The HDRP is a custom off the shelf (COTS) product. There are more than 250 installations of the application globally.***

***32 of a total of 36 German University Hospitals make use of HDRP modules. The largest German epidemiological study, the National Cohort, with 200.000 healthy volunteers, and a total collection of more than 30 million biological samples, relies on the IQVIA Biobanking Solution, which is a cornerstone module of the HDRP.***

***Research consortia have also deployed HDRP components as patient registries and for various other secondary usages of research data.***

***HDRP has also been deployed for more than 15 retrospective non interventional studies, as the core EDC (Electronic Data Capture) component. In some cases, HDRP is being used for tech enabled data collection, supporting E2E (EMR to EDC) data pipelines.***


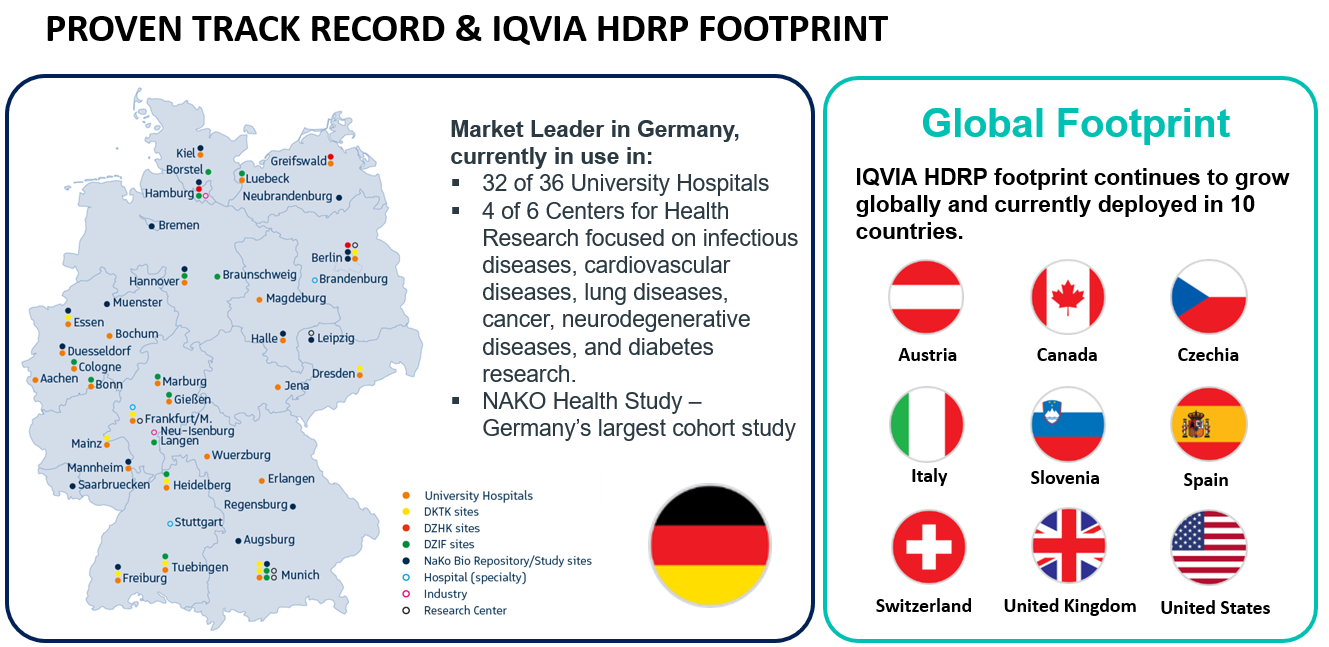


1. please add references for installations of < YOUR PLATFORM >
2. in particular please refer to scientific publications describing these installations

**HDRP components**

1. For the use case of HDRP in a local (e.g., University Hospital), networked environment

*
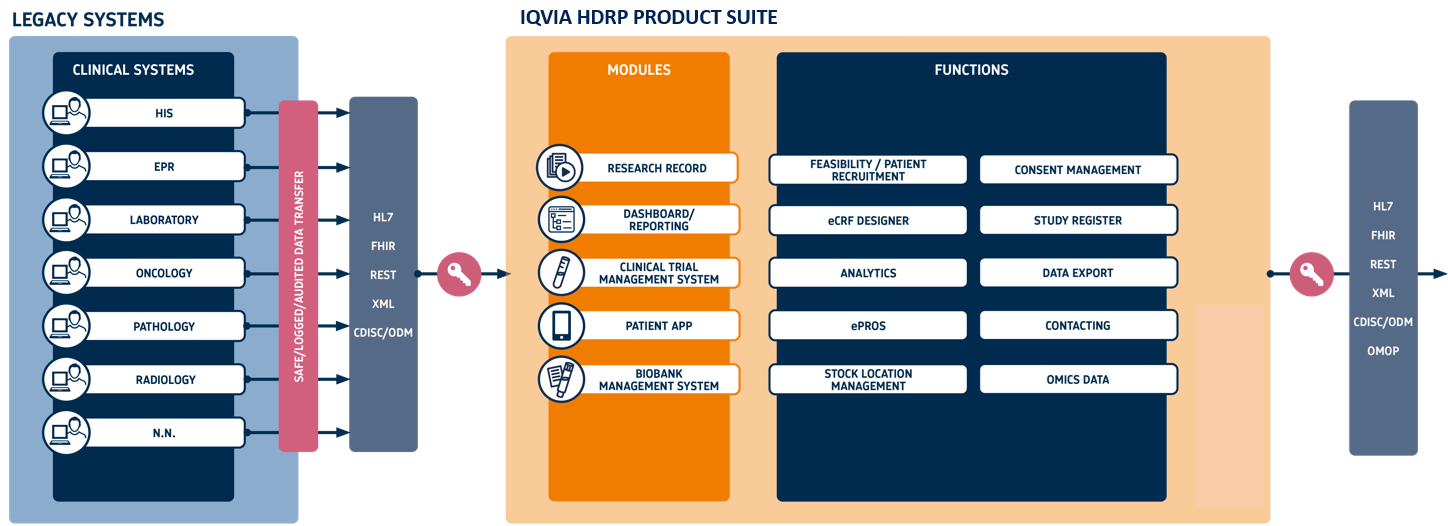
*

1. For the use case of HDRP used in a federated setup (e.g., es EDC for multi centric studies)

*
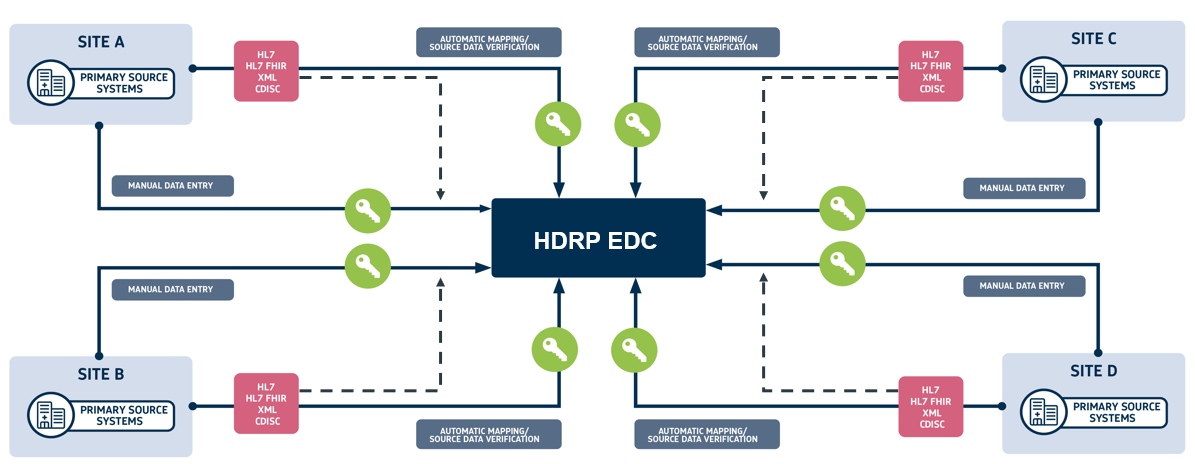
*

1. Microservices Architecture


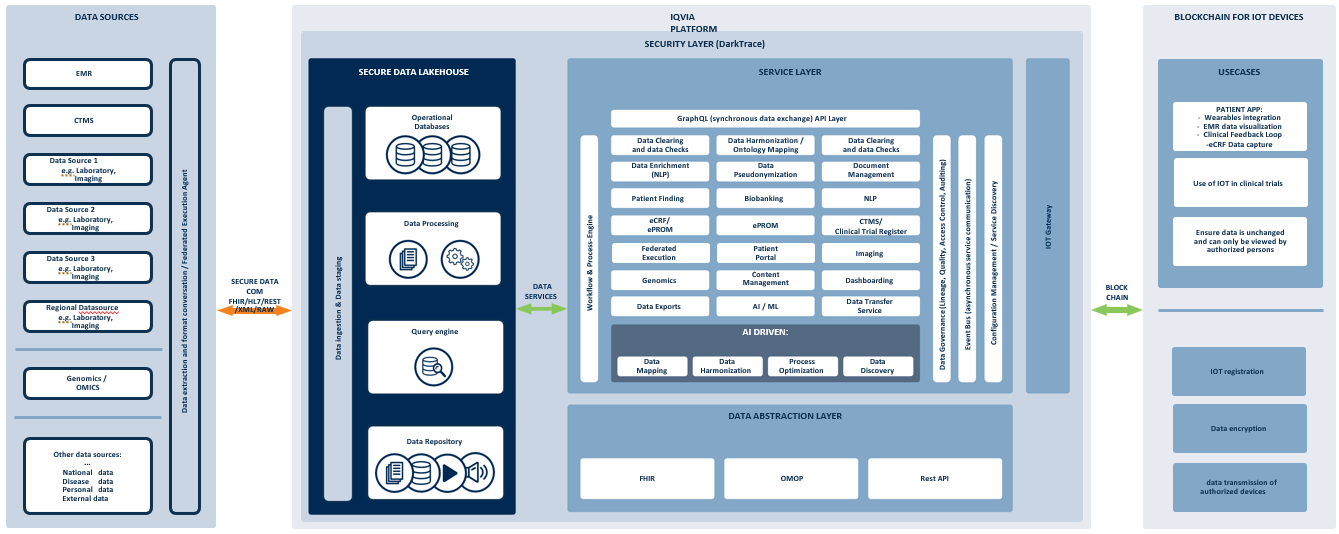


***Reference:***

[*If*](https://community.i2b2.org/wiki/display/BUN/Analysis+of+Populations%3A+Install+Guide?preview=/41746456/41746457/worddavf4910d141b05e4133d5e3adb0cc77a8b.png) *the platform architecture has been published, please add a reference here*

***Matrix HDRP features***

| *Criteria* | *Details* |
| --- | --- |
| ***Security and Privacy*** | Data access & persistence is managed through Hibernate and jOOQ, which offer fine-grained control over how data is fetched and persisted, optimizing performance. Security is central to HDRP’s design, with access control managed via OAuth 2.0 protocols, LDAPS, or SAML 2.0 compliant security providers, ensuring that only authorized users can access sensitive data. Two-factor authentication is available as an option as well, utilizing the HDRP authentication service. Data security is ensured by security through design, one of the core requirements built into HDRP as mandated by the QMS (ISO13485/9001)  HDRP is equipped with pseudonymization mechanisms, i.e. all PHI (Personal Health Information), can be scrambled according to user access rights. HDRP can also be setup in conjunction with a third-party trustee for full anonymization of all data held in the platform.  User access rights can be managed in granular detail, providing the client with full control over how individual users may access the system, and what information as well as functionality is available to them. Password complexity is configurable. Automatic log out times after intervals of inactivity can be set. A full audit trail of all inserts, updates, and deletes is written into a separate database which can be used for comprehensive audit trail reporting. Every access to individual patient/research records is logged, as well as all queries run in the system including any export of data following those queries. |
| ***Compliance and Regulatory Adherence*** | IQVIA has an available GxP assessment (GxP is used as a term to combine GCP [good clinical practice], GLP [good laboratory practice], GMP [good manufacturing practice], and GAMP [good automated manufacturing practice]. It also covers the cGxP aspects (c for “current”) from FDA’s 21 CFR chapter).  The GAMP 5 guideline was integrated into the internal Quality Management System and internal development is aligned according to defined procedure, including required documentation acc. GAMP 5 and 21 CFR 820.  KAIROS Quality Management is responsible for performing and documenting the Part 11 assessment for every released version of CentraXX.  Compliant with GDPR and apply the following standards to processes and procedures: ISO 27789, ISO13485, ISO9001, ISO 11633, ISO 30111, ISO 14971, ISO 20387, IEC 81001-5-1, IEC 81001, and IEC 62366.  KAIROS’ technical realization team responsible for implementations and deployments have completed the necessary ISO 27001 training. We can fulfill ISO 27001 requirements with server's setup in the EU, Dublin L3 Enclave. |
| **Interoperability and Extensibility** | Integration is a fundamental aspect of HDRP, with the platform using HL7-HAPI libraries for processing HL7v2 messages and HAPI-FHIR libraries for its FHIR interface. These technologies ensure seamless communication and data exchange with other healthcare systems, facilitating interoperability and data sharing. HDRP also includes a REST-based XML API for the build of custom data ingestion/integration (e.g., for legacy data imports).  HDRP also features a sandboxed Groovy engine that allows for custom script execution. This engine supports custom configurations and behaviors, such as data ingestion, data export, and data verification and validation. The Groovy scripts can feed into the internal rules engine, providing a high level of customization for HDRP processes.  To support complex, stateful workflows, HDRP includes an embedded jBPM engine. This engine allows for the automation of business processes, including decision gateways, event notifications, and other commonly used business logic features. |
| ***Data Quality and Integrity*** | Prior to any data ingestion, all data is validated according to out-of-the-box as well as customizable business rules. Simple plausibility checks are always performed (e.g., date formats, reference ranges, mandatory data points, etc.). More complex rules can be customized into entry forms through the User Interface forms designer and via Groovy Scripts (see above). |
| ***Usability and Accessibility*** | HDRP offers a user-friendly web-based front-end. The system comes with a context-sensitive user handbook.  Each project is staffed with IQVIA HDRP delivery managers that assist the clients with user training and client-tailored documentation (e.g., screencasts, handbooks, work instructions).  Web Content Accessibility Guidelines (WCAG) were consolidated during development of all frontend views in the application. |
| ***Scalability and Performance*** | One example is the use of HDRP by a large University Hospital for harmonizing and aggregating legacy EMR record data of the last 20 years into a larger repository. This resulted in loading approximately 20 million individual records with several observations/forms per record (incl. diagnoses, treatments, biospecimen data and lab results). Studies can be created through the front end in the same system, with the option of associating inclusion and exclusion criteria. HDRP continuously runs queries across the existing data, notifying personnel about the records that meet a configurable threshold of the search parameters. Performance of the system is constantly being monitored and results are made available through a Micrometer interface, access able through a REST interface. |
| ***Collaboration and Sharing Capabilities*** | HDRP is used in projects that allow federated queries. In one case study, HDRP is used for site data harmonization, aggregation, and conversion into OMOP standard. This dataset can then be used by third-party tool for federated queries and delivery of aggregated/de-identified record counts to the querying party after approval of the query by the site delivering the record count.  The ‘repository connect module’ allows to connect an external FHIR store to HDRP, to be searched in parallel. Data can then on demand automatically be transferred from the external data store to HDRP if needed.  The ‘study recruitment network’ is another capability leveraging distributed query handling among connected sites for feasibility queries.  Build on the FAIR data principles, HDRP offers a variety of integration and pipeline use cases and interfaces to become part of a federated data pipeline.  HDRP is used widely by academia in collaborative approaches. One example is by sites partnering in a hub and spokes model, delivering select data through proprietary synchronization tools from local sites into a central solution.  Another option selected by clients is opening access to their local solutions to partners through customized workflows, managing precisely what these partners may do and see in the application.  Fine-grained access rights can be given on an individual user basis with out-of-the-box functionality, allowing setup of temporary access for collaborators, with de-identified/limited views only. |
| ***Cost and Sustainability*** | HDRP can be offered at an annual subscription rate, or with perpetual licenses, which can be more cost-efficient to the client in the long run.  HDRP is currently broadly used and supported by IQVIA as a strategic product in its tech-enablement efforts for its several prime and partner sites across the globe. IQVIA offers annual Maintenance and Support contracts for its clients, with ongoing support in managing and extending the solution, as well as collaboration across partners. |
| ***Ethical Considerations*** | Informed Consent can be managed in multiple ways. At a minimum, HDRP can be used to manage structured information about consent provided by patients within the patient record. This tool can be used for broad consent, or where needed, to map out tiered, detailed consent forms (e.g., for components to individually track permission to recontact, to carry out DNA analysis, to reuse data for other research projects, etc.).  If consent is captured paper-based, a scan of the actually signed document can be uploaded and linked to the patient record consent.  The HDRP mobile Patient App can also be used for patients to submit consent to the platform electronically, with a signature via Sign pad, as well as biometrics-confirmation (e.g., fingerprint) during submission.  Several clients have undergone Ethical review processes regarding their use of HDRP. This process is almost always required by the sites prior to go-live with the tools. IQVIA supports these clients with all necessary documentation. |
| ***Innovation and Adaptability*** | IQVIA continues to build out innovative technologies into HDRP. Examples include the recent addition of an OMOP converter, integration of a Natural Language Processing (NLP) Workflow for the structuring of pathology reports PDF, extension of existing Study Management capabilities of the IQVIA Trial Manager with study budgeting capabilities, and much more.  HDRP was designed as a modular application. This allows clients to start small and scale up by adding additional functionality as the need arises. This requires license key activation only, without the need of code development.  The tools come with various configuration functionality by the clients themselves through user interface designers. As it is impossible to foresee all changes required for data collection in research, the tool allows easy changes/configurations to be made without the potential bottleneck caused by requiring programmers to make those changes. All the while, the tool is equipped with standard APIs to allow interoperability despite user interface changes made by clients for customized data collection. |

***References***

*1 your references for relevant literature go here*

***Matrix HDRP common challenges***

| **Category** | **Description** |
| --- | --- |
| **Federated Queries Challenges** | Bringing together relevant datasets into a common data model to allow for federated queries is not free of challenges. Firstly, data harmonization is difficult, as data will often be using different ontologies. Next, often most of the data does not even yet exist in structured format but is rather currently “hidden” in unstructured notes.  HDRP tools come with various tools and ideas to help mitigate. |
| **Patient Privacy and Data Protection** | HDRP tools make sure patient privacy is consistently maintained while allowing for sensible and meaningful research on consented unidentifiable datasets. |
| **Organizational Policies** | HDRP has been developed in close collaboration with our clients for the past 14 plus years. Most of our clients come from the hospital space, hence much of the functionality revolves around hospital-specific use cases (e.g., separation into organization units, and departments for multi-client capability). |
| **Data Transformation requirements** | HDRP is equipped with several APIs that allow immediate data ingestion from sources that adhere to standards (e.g., HL7 v2, FHIR profiles supported by HDRP, CDISC-ODM). IQVIA supports clients in customizing these technologies for a tailor-made ingestion pipeline, either with service days to build and maintain the data path, or by offering training on how to manage the technology independently.  In addition to the standards mentioned above, the REST based XML-API can be used for bespoke ETL pipelines. |
| **Installation and Maintenance** | HDRP can be deployed via MSI installer package. Clients can opt to self-manage their deployment processes or acquire services by IQVIA’S technical realization team (TR) for everything related to the deployment. Cloud-based, as well as on-prem installations, are possible.  Cloud native deployments in Azure, utilizing AKS is possible as well.  IQVIA offers annual Maintenance and Support contracts. These manage Service Level Agreements (SLAs) as well as access to feature updates (made available roughly quarterly) and bugfixes (made available roughly bi-weekly, or in urgent cases as soon as possible). Clients also receive access to a telephone hotline and an online ticket system. |
| **Secure Deployment** | HDRP offers flexible deployment options, either as a traditional application or in a cloud-native, container-based environment. It supports on-premises installations as well as cloud-native service integration into Kubernetes or Docker-based environments, such as Azure Kubernetes Services (AKS). This allows organizations to leverage cloud-native database instance services, offering scalability and flexibility to meet evolving needs. |
| **Understanding User Queries** | HDRP comes with an out-of-the-box query interface that allows clinicians to query all structured clinical and translational research data their user has access to. The query allows using AND/OR operators, as well as various qualifiers (ranges, greater than, smaller than, equals, does not equal, etc.). Data points can be bracketed in searches. All searches can be saved and shared across users, or user groups. Patient and or sample result tables can be created, columns can be configured, and the outputs can be exported (via CSV or PDF), should the user be allowed to perform the export.  For all queries that require more logic, or computations and visualizations of the data, HDRP offers an integrated Reporting Engine. This allows the manual or automatic execution of report templates built with Business Intelligence Reporting Tools (BIRT), incl. export via CSV; xlsx, PDF, etc. (e.g., for the push of weekly inventories to management).  IQVIA also offers the HDRP dashboard. This tool allows for real-time data visualization of a connected HDRP platform. |
| **Informatics and User Experience** | The user is provided access to the front-end only, with a user-friendly web interface.  IQVIA continues to improve its application by converting client-provided feature requests (e.g., based on tickets in the hotline system, or following client survey provided after annual user days).  IQVIA provides continued support to its client base, for the delivery of client specific requests in the form of tailor-made reports (e.g., for data retrieval), or the development of client-specific workflows that orchestrate client-specific business processes in orchestrated documentation flows in the application. |
| **Complexity of < YOUR PLATFORM > Software** | Depending on the use case, the software can be quite complex, when a range of functionality is intended to be made available to a user. User options can be limited through user rights that can be granted on an individual user level, i.e. if a user is only permitted to run queries, this will be the only option visible to a user, making the system much easier to adapt for a new user.  Navigation in the system is intuitive, and the context-sensitive handbook can always be used by the user for guidance.  Custom-built workflows can be developed, for more client-tailored documentation flows in the system.  IQVIA’s HDRP has been slightly adapted in recent years for a wide range of use cases. These include, but are not limited to, use by Life Sciences companies for tracking of inventory and documenting the production of therapeutics, a hospital survey system after patient discharge, a standalone PROM system, EDC-tool for single multi-centric studies with EMR to EDC capabilities, consent and contact management via Patient App). |
| **Incremental Updating Limitations** | HDRP has been set up in several different architectures to assist our clients on these aspects. This includes synchronization options between separate HDRP instances, as well as including third-party ID management/trustee systems.  The HDRP database structure is divided into separate databases that allow storing identifying data on physically separate storage from the research relevant data. |
| **Standardized Vocabularies and Flexibility** | Where possible, HDRP ties in standard ontologies and catalogues into the application. This includes, ICD-10 and OPS catalgues, LOINC and SNOMED terminology via the HDRP Meta Data Repository, the SPREC catalogue for biospecimen handling, and any other client specific custom catalog that can be uploaded into the application and used for single or multi selector fields in data entry forms. |

***References :***

1. please add references here or directly in the fields of the matrix

***Data Modalities Supported by HDRP***

Usually, clinical research data platforms are designed to integrate and manage a wide range of data modalities to support biomedical research. The primary data modalities used so far in

HDRP include:

| **Category** | **Data Modality** | **Description** |
| --- | --- | --- |
| **Clinical Data** | Electronic Health Records (EHRs) | HDRP is a patient-centric solution. All clinical information is stored in patient/research records under unique identifiers that allow for longitudinal data collection. Data includes structured information on several relevant data points, structured into various tabs in the patient records, which include tabs on consent, diagnoses, therapies, biospecimen data, lab result data, tumor data, and more. Unstructured data can be uploaded to or linked from the record (e.g., DICOM images, physician’s notes, images, lab result PDFs, etc.). |
|  | Hospital Administrative Data | Admissions, discharges, transfers, billing codes, and insurance information can be captured in the patient records as well. Many of our clients make use of our HL7 Listener, and the supported ADT message type for loading this data from their EMR system into HDRP: |
| **Genomic Data** | Genomic Sequences | Result data from whole genome/exome sequencing runs can be loaded into HDRP in a structured format, as well as loaded and linked (e.g., as VCF files). Raw data typically is not duplicated into client systems but result data can be linked to the corresponding specimen delivered to the labs for WGS. |
|  | Genotype Data | Clients can build their own profiles for capturing various genotype datasets (incl. but not limited to Single nucleotide polymorphisms (SNPs), copy number variations (CNVs). The profiles created through the user interface can be used for data ingestion via the mentioned APIs. |
|  | Gene Expression Data | Clients can build their own profiles for capturing various transcriptomics datasets. |
| **Imaging Data** | Radiology Images | Images can either be loaded directly into the system, or they can be linked to from the corresponding patient/research record. Depending on the size of the image, we would recommend linking to the images rather than duplicating. |
|  | Pathology Images | Images can either be loaded directly into the system, or they can be linked to from the corresponding patient/research record. Depending on the size of the image, we would recommend linking to the images rather than duplicating. |
| **Phenotypic Data** | Disease Phenotypes | Clients can build their own profiles for capturing various disease phenotype datasets (incl. the option to integrate controlled vocabulary/catalogs for annotation). |
|  | Clinical Outcomes | Clients can build their own profiles for capturing datasets on treatment responses and survival rates. Reporting and dashboarding functionality allow for data analytics. |
| **Medication Data** | Prescription Records | One of the tabs in the system is for capturing information related to patient medication. This includes information on the name, the PZN, the filler order number, dose strength, quantity, scheme, ordinance release form, active ingredient, active ingredient group, application method, dose form, application medium, Is dose, target dose, deviation target dose, medication arrangement, prescribed by, prescribed on, and more. |
|  | Medication Adherence / Compliance | Patient questionnaires can be configured in the system, and data entry can be made available to patients through use of Patient App or via web application. |
| **Laboratory Data** | Lab Test Results | Clients can build their own profiles for capturing data on blood tests, urine tests, microbiological cultures, biochemical assays. Biomarker measurements, etc. All forms created in HDRP can be used for manual data entry as well as for data ingestion through API. |
| **Survey Data** | Questionnaires and Surveys | Patient health questionnaires can be configured in the system, and data entry can be made available to patients through use of Patient App or via web application. |
|  | Patient-Reported Outcomes | HDRP includes a PROM module. |
| **Biomarker Data** | Proteomics | Clients can build their own profiles for capturing data on protein expression, protein-protein interactions, post-translational modifications, Mass-Spec result data, etc., All forms created in HDRP can be used for manual data entry as well as for data ingestion through API. |
|  | Metabolomics | Clients can build their own profiles for capturing data on metabolite profiles, metabolic pathways, lipidomics, etc. All forms created in HDRP can be used for manual data entry as well as for data ingestion through API. |
| **Environmental Data** | Lifestyle Factors | Clients can build their own profiles for capturing data on diet, physical activity, workout schemata, smoking, alcohol consumption, substance (ab)use, etc. All forms created in HDRP can be used for manual data entry as well as for data ingestion through API. |
|  | Environmental Exposures | Clients can build their own profiles for capturing data on air quality, water quality, exposure to toxins, occupational hazards, etc. All forms created in HDRP can be used for manual data entry as well as for data ingestion through API. |
| **Socioeconomic Data** | Social Determinants of Health | Clients can build their own profiles for capturing data on education, income, employment status, housing, neighborhood characteristics, etc. All forms created in HDRP can be used for manual data entry as well as for data ingestion through API. |
| **Family History Data** | Genetic Risk Factors | Clients can build their own profiles for capturing data on family history of diseases, pedigree analysis. Risk alleles, tumor gene panels, etc. All forms created in HDRP can be used for manual data entry as well as for data ingestion through API. |
| **Longitudinal Data** | Time-Series Data | HDRP includes a Measurement Findings tab in the patient/research record. This allows presenting Measurement series over time. With an integrated monitoring tool, users can define ranges and select from individual values or entire panels to show the development of certain values (e.g., blood values) over time. For more detailed requirements of how to visualize and compute datasets, IQVIA recommends use of its HDRP dashboarding tools, or a customized design of tailormade reports via BIRT tools (see above) for manual or automated extraction of time series data. |
| **Behavioral Data** | Behavioral Assessments | Clients can build their own profiles for capturing data on cognitive tests, psychological assessments, behavioral interventions. Nutrition coaching, etc. All forms created in HDRP can be used for manual data entry as well as for data ingestion through API. |
|  | Transcriptomics | Clients can build their own profiles for capturing data on mRNA levels, non-coding RNAs, alternative splicing events., etc. All forms created in HDRP can be used for manual data entry as well as for data ingestion through API. |
| **Pathway Data** | Biological Pathways | Clients can build their own profiles for capturing data on Signaling pathways, metabolic pathways., etc. All forms created in HDRP can be used for manual data entry as well as for data ingestion through API. |
|  | Interaction Networks | Interaction networks are not supported or shown directly within HDRP, but Web-links to external networks are possible. |

***References :***

1. please provide references to relevant publications / documentation here

**Built-in Workflows and Analysis Tools**

Does HDRP contain built-in workflows and analysis tools that facilitate clinical and translational research?

**Workflow**

| **Feature** | **Description** |
| --- | --- |
| Patient Cohort Discovery | HDRP includes out-of-the-box functionality for designing structured queries that can be run manually or automatically for identifying patient cohorts that match certain criteria. All relevant structured patient data (incl. diagnoses, demographics, medications, procedures, lab results can be used in the query). |
| Data Integration and Management | HDRP can function as a clinical data warehouse and can be used to integrate relevant clinical data from multiple sources into a common data model (CDM). |
| Ontology Management | IQVIA offers the HDRP MDR (Meta Data Repository), which can be used in conjunction with the HDRP system for ontology management. |
| Data Extraction and Transformation | IQVIA offers a range of APIs for data ingestion.  Typically, we would deploy our FHIR interface, or our REST based XML API for ETL jobs. A MIRTH connect server can be used for managing multiple source system feeds. |
| Security and Privacy Management | Access is managed with role-based access that can be given by the system administrators via the user interface. Patients and users are grouped into 1-n organization units in the system. Only where there is overlap in the patient’s organization units with those of the user, will the user be able to search and find the patient’s record. All data can be visualized in a pseudonymized form for users based on access rights. |

**References:**

1. references go here

**Analysis Tools**

| Query Interface | HDRP comes with an out-of-the-box query interface that allows clinicians to query all structured clinical and translational research data their user has access to. The query allows using AND/OR operators, as well as various qualifiers (ranges, greater than, smaller than, equals, does not equal, etc.). Data points can be bracketed in searches. All searches can be saved and shared across users, or user groups. Patient and or sample result tables can be created, columns can be configured, and the outputs can be exported (via CSV or PDF), should the user be allowed to perform the export.  For all queries that require more logic, or computations and visualizations of the data, HDRP offers an integrated Reporting Engine. This allows the manual or automatic execution of report templates built with Business Intelligence Reporting Tools (BIRT), incl. export via CSV; xlsx, PDF, etc. (e.g., for the push of weekly inventories to management).  IQVIA also offers the HDRP dashboard. This tool allows for real-time data visualization of a connected HDRP platform. |
| --- | --- |
| Timeline Viewer | Every patient record comes with the tab “Time course”. This allows creating and editing a longitudinal overview of all relevant patient data on a timeline, with the option to select and deselect several event types such as diagnoses, biospecimen, lab result findings, etc. |
| Statistics and Analytics | IQVIA offers the HDRP dashboard, and an integrated reporting engine for statistics and analytics on all HDRP data. |
| Plugin Framework | It is possible to use third party statistics tools and access all data from HDRP’s relational database (for which MSSQL, ORACLE, or PostgreSQL can be used).  While the integrated jBPM engine allows for dynamic integration of external tools and provides and receives data, it is not a defined plugin architecture, but follows our external Workflow API, which can be used for the integration with external tools.  Through the interoperability layer, HDRP offers direct integration through HL7-FHIR and HL7v2.x.  Also there is a REST API which can be used to integrate external tools and applications. |
| Natural Language Processing (NLP) | IQVIA has collaborated with Averbis (third party) to establish customized NLP pipelines (for the ingestion of medication, and diagnostic data from physician’s release notes in PDFs). HDRP provides a complete NLP workspace for supervised and un-supervised NLP. |
| Genomic Data Analysis | HDRP does not support genomic data analysis within the solution but can integrate structured results from other analytical solutions.  Therefore, VCF and FHIR-VCF can be imported directly as structured information and can be used for cohort building or detailed result sets to biospecimen |
| Temporal Querying | These types of queries cannot be built with the query interface. Where more logic is needed for queries, IQVIA uses the integrated reporting engine for these types of queries. |
| Data Visualization | IQVIA offers its HDRP Dashboard for this. Reports via report engine can also be used to create time-stamped reports and data visualization. |
| Export and Reporting | Data can be exported in various formats and allow interoperability, i.e., export in a structured format for reuse by other systems (e.g., statistical tools). |

***References***

*1.*references go here

| **Integration with Other Tools** | R / BioConductor and Python Integration | HDRP is integrated with R-Shiny to generate several analytical visualizations and dashboards. |
| --- | --- | --- |
|  | Integration with Clinical Trial Management Systems (CTMS) | One of the core modules of HDRP is the CTMS (Clinical Trial Management System), which comes with functionality including a Study Register, a Study budgeting module, an EDC tool for actual clinical documentation into configurable eCRFs, study inclusion and exclusion criteria management, randomization into study arms, configurable export of eCRF data, SAE management, and more. |
|  | Integration with Electronic Health Records (EHR) | A core piece of HDRP is its API structure that allows for data ingestion. For most clients, the primary system data is pulled from the EMR system. Typically, the HDRP HL7 Listener will receive access to a comm server and is able to ingest EMR records via several HL7 message types. HDRP can also ingest data from several FHIR profiles, and the REST based XML API can also be used for EMR source data ingestion. |

References:

1. references go here

**Support for Semantic Integration**

HDRP supports semantic integration through the use of terminologies, ontologies, and common data models, such as:

1. **Terminologies and Ontologies**: HDRP can integrate with standard medical terminologies and ontologies such as ICD, SNOMED CT, LOINC, and others. This ensures consistent data representation and facilitates interoperability.
2. **Common Data Models (CDMs)**: HDRP is equipped with an OMOP converter, enabling data standardization and easier data sharing across institutions.?
3. **Ontology Management**: IQVIA offers the HDRP Meta Data Repository, which allows users to customize and extend the ontologies as needed to fit their specific research requirements

**References** :

1. References go here
