# Supplementary material for: Selecting medical research data platforms for translational biomedical research: a five-tier overview and requirement-weighted assessment framework
Source: Front Digit Health. 2026 Jun 17;8:1814015. doi: 10.3389/fdgth.2026.1814015 (PMC13319098; doi:10.3389/fdgth.2026.1814015)
Supplement: Supplementary file 15 [file Supplementaryfile15.docx]

***NVIDIA FLARE***

***Deployment and Usage****:*

[*NVIDIA FLARE*](https://nvidia.github.io/NVFlare/) *(NVIDIA Federated Learning Application Runtime Environment) is a domain-agnostic, open-source, extensible Python SDK that allows researchers and data scientists to adapt existing ML/DL workflows to a federated paradigm. It enables platform developers to build a secure, privacy-preserving offering for a distributed multi-party collaboration.*

*NVIDIA is widely used by both academia and industries for various purposes including: medical imaging (such as radiology, digital pathology), cancer studies, financial services (fraud detection, anti-money laundry (AML), autonomous vehicle, transportation fleet management, medical device application (NVIDIA Holoscan), Scientific computing, drug discovery (on-going FL+ Web lab), energy study etc.*

*Several startups are also powered by FLARE, such as Rhino Health, Apheris.*

*The FLIP project of the UK AI centre is powered by FLARE,* [*FLIP*](https://aicentre.co.uk/platforms%22%20/l%20%22view2) *allows AI researchers to develop clinical applications on NHS patient data without the information ever leaving the hospital network. FLIP is being deployed in at least 5 NHS trusts serving over 10 million patients in early 2023. The first projects using FLIP include research into AI that can diagnose the severity of strokes from head CT scans, an algorithm that can help radiographers detect and diagnose prostate cancers, and software that uses AI to reduce the time of cardiac MRI scans.*

*You can find more real-world FL showcases with NVIDIA FLARE in the NVFLARE DAY 2024 talks.*

***References:***

[***https://arxiv.org/abs/2210.13291***](https://arxiv.org/abs/2210.13291)

[***https://github.com/NVIDIA/NVFLARE***](https://github.com/NVIDIA/NVFLARE)

[***https://nvidia.github.io/NVFlare/flareDay/***](https://nvidia.github.io/NVFlare/flareDay/)

[***https://www.aicentre.co.uk/news-and-events/news/flip-and-aide-released-as-open-source-platforms***](https://www.aicentre.co.uk/news-and-events/news/flip-and-aide-released-as-open-source-platforms)

***NVIDIA FLARE Components***

***FLARE architecture: federated component engine***

***
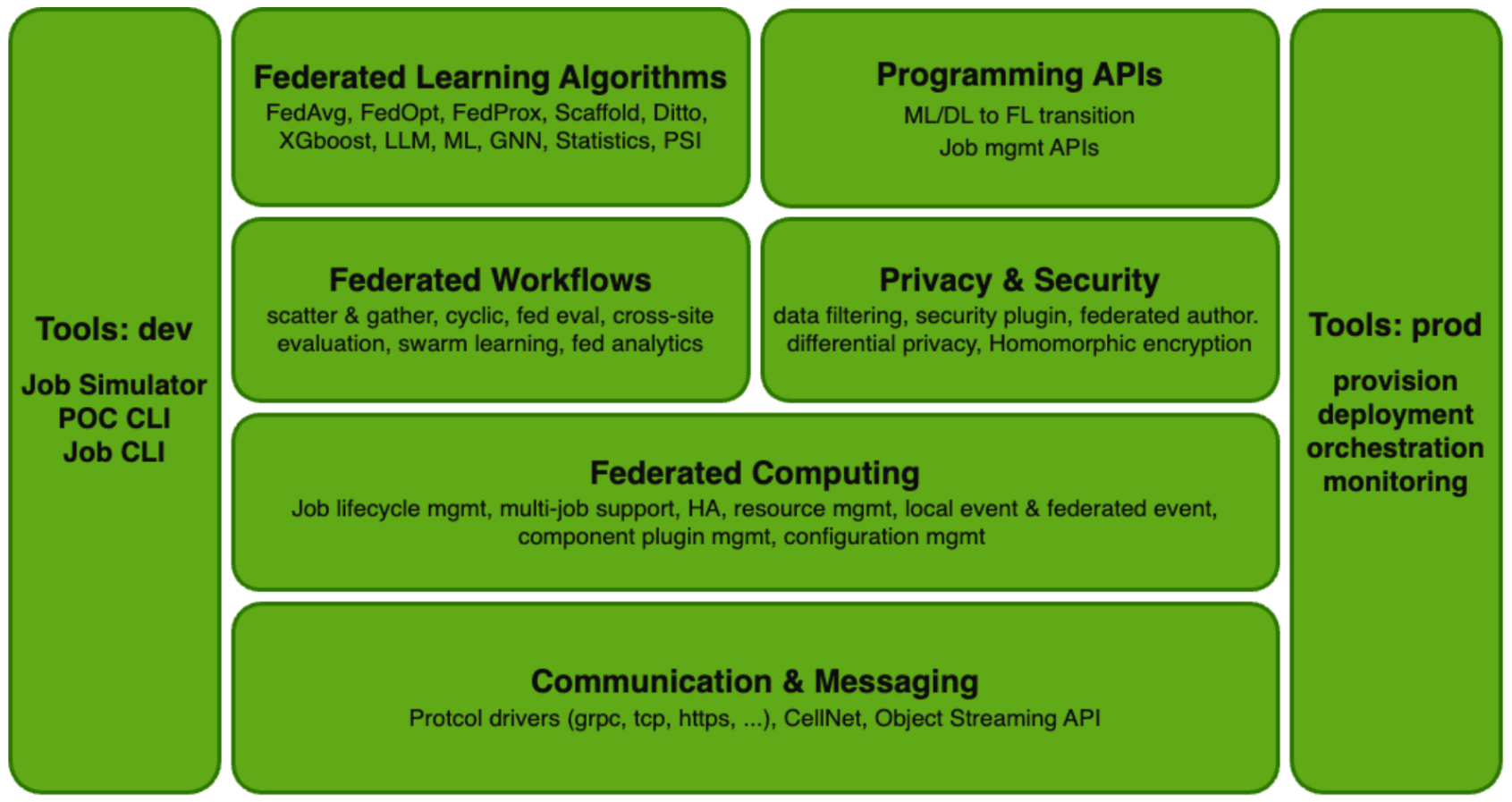
***

***
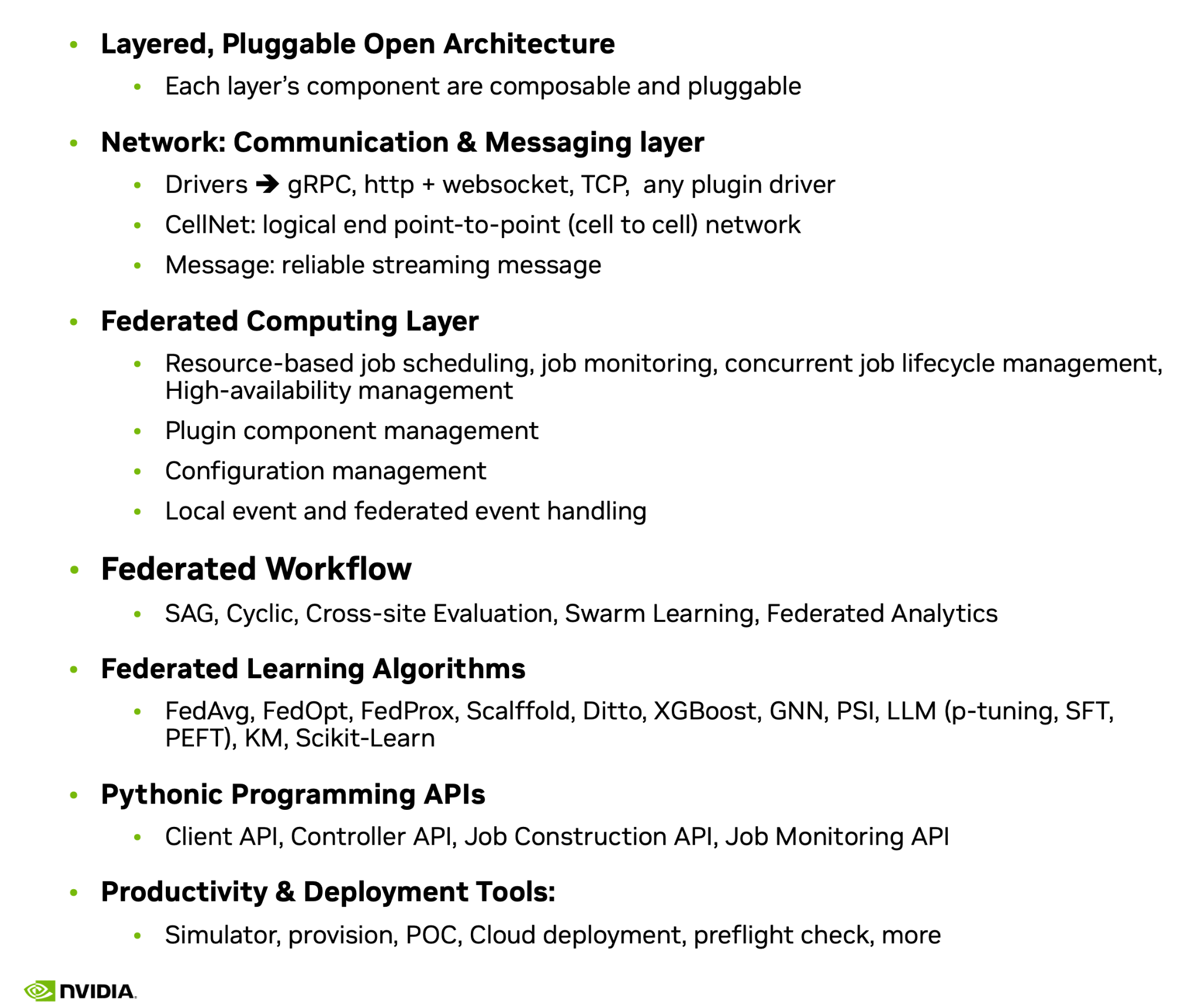
***

***FL Server and Client Interactions***

***
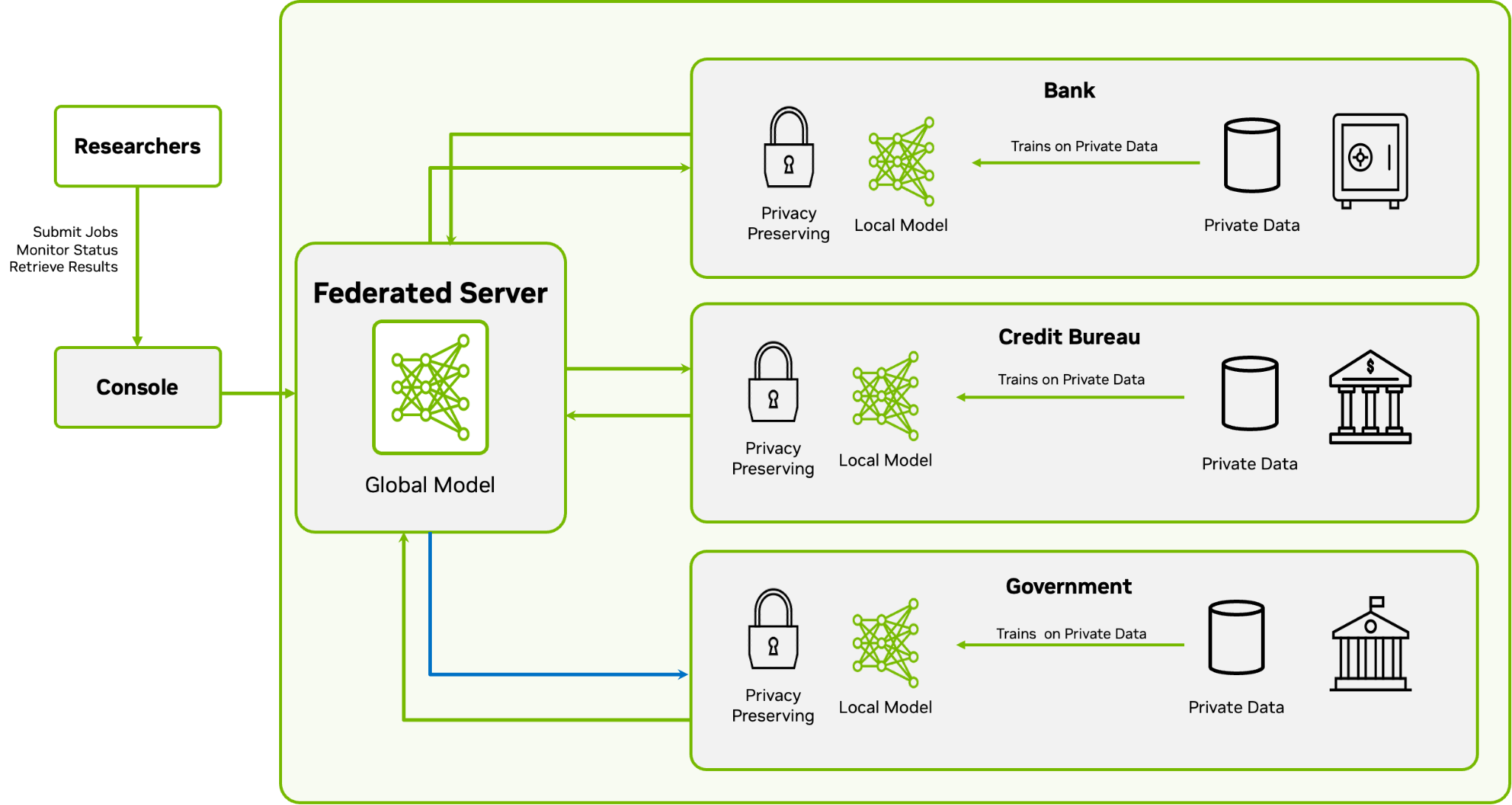
***

***
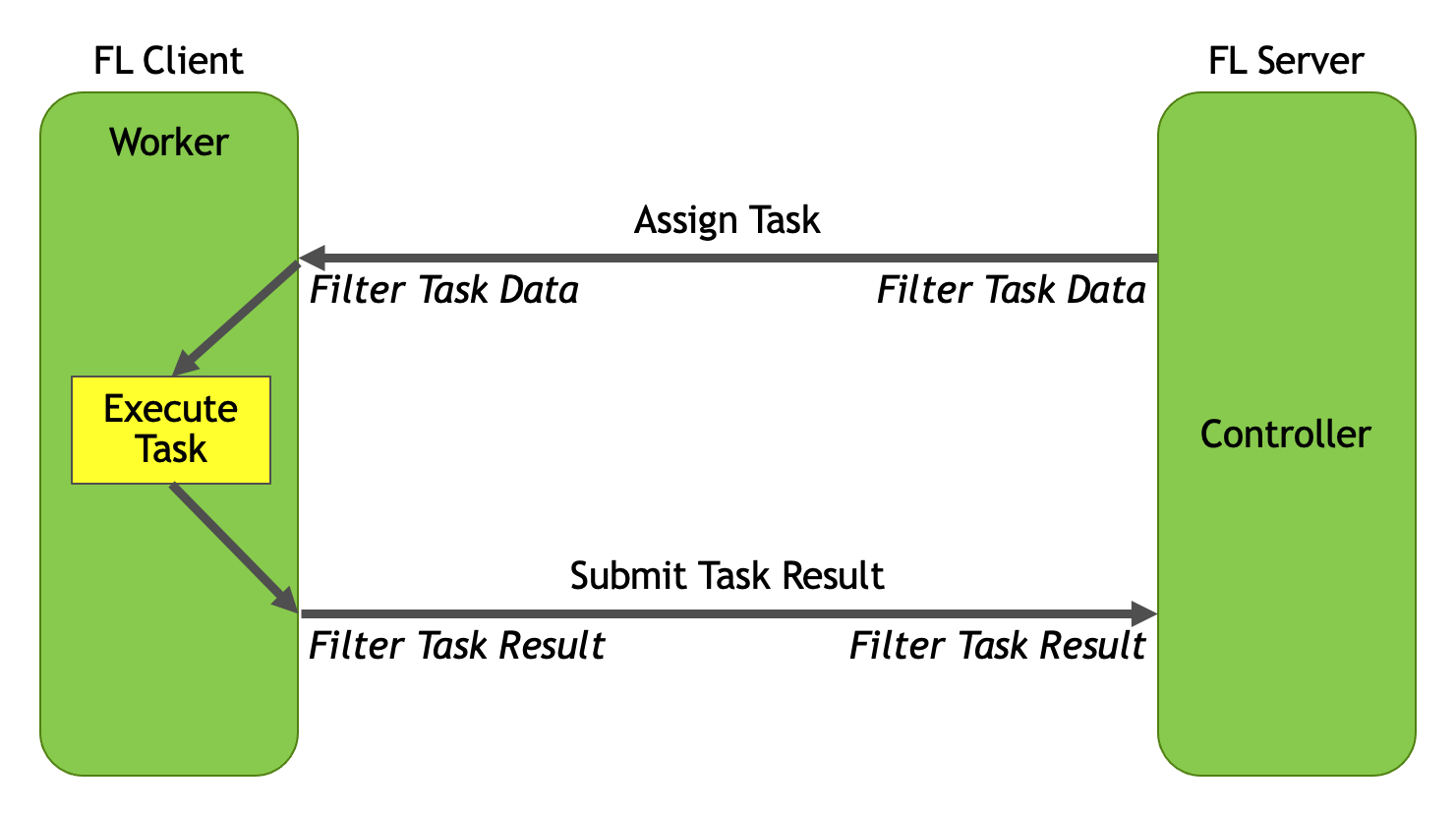
***

***FLARE Board UI for software distribution***

***
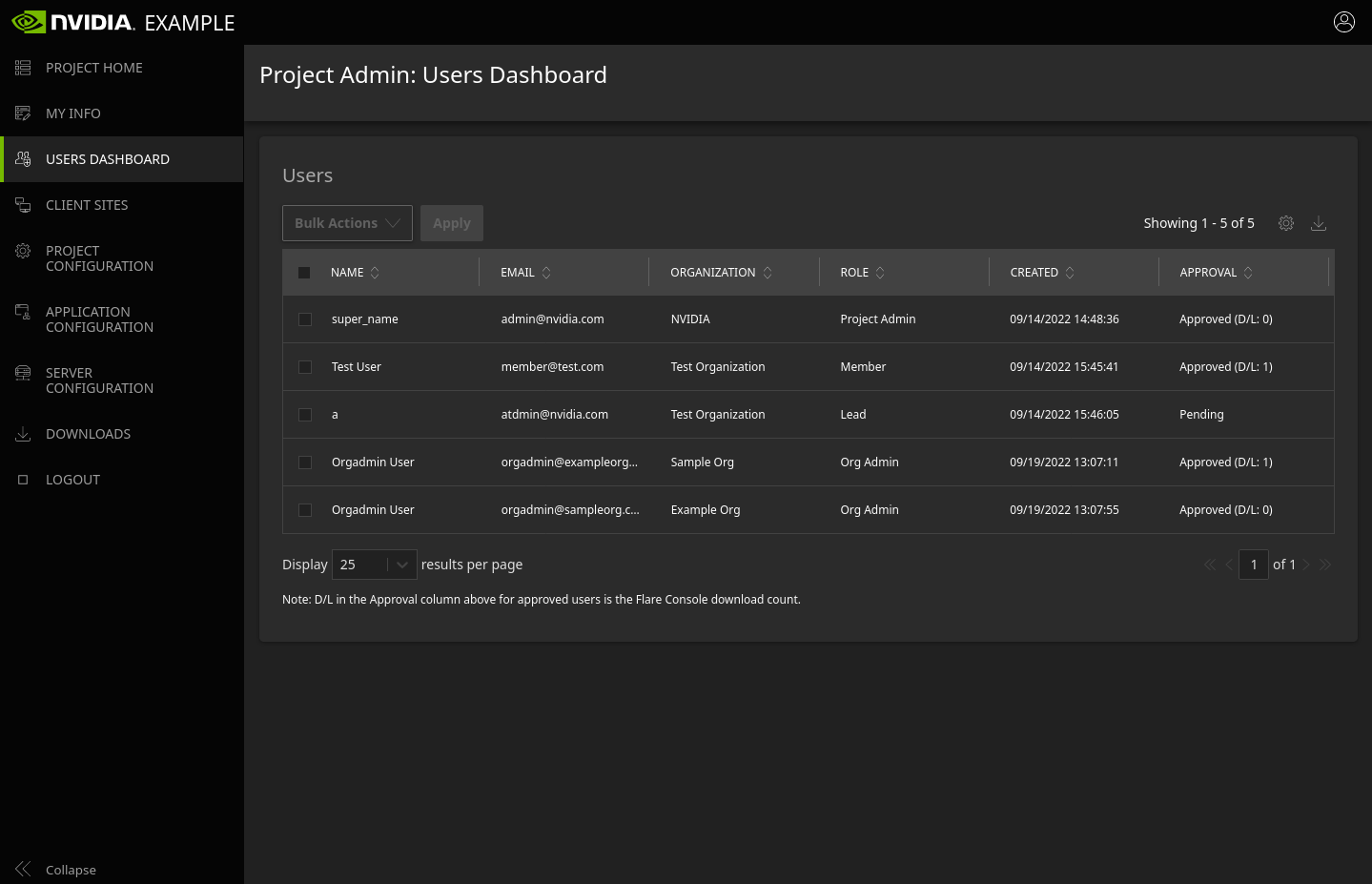
***

***References:***

[***https://nvflare.readthedocs.io/en/main/flare_overview.html***](https://nvflare.readthedocs.io/en/main/flare_overview.html)

[***https://nvflare.readthedocs.io/en/main/programming_guide.html***](https://nvflare.readthedocs.io/en/main/programming_guide.html)

[***https://arxiv.org/abs/2210.13291***](https://arxiv.org/abs/2210.13291)

[***https://nvflare.readthedocs.io/en/main/user_guide/dashboard_ui.html***](https://nvflare.readthedocs.io/en/main/user_guide/dashboard_ui.html)

***Matrix table for the NVIDIA FLARE features***

***Notes;***

*NVFLARE is a generic federated compute & Federated learning platform, which is not specific for the healthcare lifecycle industry. Our design philosophy is different from the medical specific platform. The platform is not specifically tailored to particular applications, instead, through NVFLARE’s plugin-component mechanism, we are able to handle all specific needs, except these special purposes will not be part of core NVFLARE. Therefore some of the questions domain specific here will be addressed via plugin, but not provided by FLARE*

*NVFLARE is a Federated Learning and computing Platform, but it is* ***not a federated data platform****. The many capabilities associated with data platforms such as data governance, data lineage, and data quality assurance, data ingestion and transformation from different formats are not the responsibility of the NVIDIA FLARE.*

| ***Criteria*** | ***Details*** |
| --- | --- |
| ***Security and Privacy*** | *FLARE offers* ***enterprise level security*** *and make sure its ready for real-world production needs*  ***General security***  *client /server connection authorization: FLARE leverage PKI mechanism to authenticate the connection between client & server*  *The TLS encrypt the message during transit*  *User Roles: we support 4 roles: Project Admin, Org Admin, Lead Researcher and Member, each role has different privileges at project level, site-level and job level*  [*https://nvflare.readthedocs.io/en/main/user_guide/security/identity_security.html#roles*](https://nvflare.readthedocs.io/en/main/user_guide/security/identity_security.html#roles)  ***Additional Security Control***  *We provide* ***federated authentication and authorization*** *mechanisms which can be integrated with local,* ***site-specific*** *authentication mechanisms. The authorization is job-level site-specific,which means the same user can be authenticated by hospital 1, but maybe rejected by hospital-2 for the one job. But both can be allowed for the next job.*  ***Data Privacy Control***  *FLARE provides a* ***filter*** *mechanism to filter the data in/out the client or server component. This mechanism can be used to apply differential encryption or homeomorphic encryption privacy enhancing technologies. The same filter is used for support site-specific privacy policies. NVFLARE provide an example how to perform local-site-specific privacy policy*  *FLARE also supports Differential Privacy (DP), Homomorphic encryption support. You can find examples in github repo for Deep learning as well as Secure Federated XGBoost examples.*  *FLARE supports peer-to-peer messaging (example, in swarm learning) via client-side controller. In such cases, secure message communication is used, where the peer-to-peer message is only decrypted by sending and receiving parties.*  ***Confidential Computing***  *FLARE supports end-to-end confidential computing which allows alternative ways to provide secure aggregation and model theft protection*  *Data encryption and decryption is specific to the data; processing is not the core of the FLARE, if such capability is desired, the plugin component can be written for the desired encryption algorithm. One can place component at beginning of FLARE workflow*  *De-identifacation is usually the part of the data-pipeline process where the data cleaning, normalization and other ETL activity. This can be coordinated by FLARE, but it is not core to the FLARE. Similarly one can add an extra component for this, or extra step for de-id. FLARE does show an Federated ETL for feature engineering, pre-process steps.*  *Usually the De-identification process may involve different tools depending on the customer's technologies and tools: one can use python, Apache Spark, Ray framework etc. different tools, or Spark Rapid for large data volume. Whatever tool we provide is not going to fit the needs for different use cases.* |
| ***Compliance & Regulatory Adherence*** | ***These regulatory rules are region specific, industry specific and domain specifics.***  *As the general Federated learning/compute we can’t not ensure how to enforce and comply with such regulations. We can only provide the tools and technologies to help clients to comply with such regulartions, but the framework itself can’t do that.*  *For example, for HIPPA compliance, FLARE can’t guarantee HIPPA compliance, If the data scientist uses FLARE but training code sends user information to another site, we can’t prevent that. The company can add a privacy filter in the privacy policy to catch such cases. But someone in the company needs to enforce such practice.*  *FLARE provides a way for FL users to train models without moving data, this helps industries to comply with EU GDPR, AI ACT, California’s CCPA, China’s Privacy law.*  *In fact, FlARE is used for cross-border training for autonomous vehicles for such compliance.* |
| ***Interoperability and extendibility*** | *FLARE is highly extendable and pluggable. Every layer’s components can be replaced with new components. This can be from lower messaging layer such as network protocols to higher layers. One uses gRPC, TCP, HTTP or any other protocols, one can also replace FedAvg implementation with once’s own fed avg.*  *We are easy to adapt, we have integration with Monai, Holoscan, other HPC training facilities*  *But out of the box, FLARE doesn’t have components for healthcare specific Data loaders for specific formats. Those should be considered optional plugin components* |
| ***Data quality and integrity*** | ***Data quality, data lineage & Audit tail, data governance***  *These are functions of the data platform, not federated learning platform. FLARE is not a data platform, thus does not support these features*  *On the other hand, FLARE supports other features related data quality and integrity*  *We can access the data quality based on their constitution to the model: contribution estimation; we are working to verify the data/code/model integrities at run time* |
| ***Usability and Accessibility*** | *FLARE offers extensive documentation and training materials.*  [*https://nvidia.github.io/NVFlare/*](https://nvidia.github.io/NVFlare/) |
| ***Scalability and performance*** | ***Scalability*** *comes from different angles:*  *Scale number of sites/devices*  *Scale of handing data size ( usually for data platform for ETL or feature engineering)*  *Scale of transfor large data volume to remote site (LLM training)*  *NVFLARE scales well in all dimensions. We can support a large number of cross-silo sites. We are working to support scale cross-edge devices*  *There is no limitation for NVFLARE to handle large data size even though FLARE is not data platform*  *FLARE offers object streaming and can stream LLM (hundreds of GB) over internet from one country to another*  ***Performance:***  *FLARE performance is robust, able to scale to large number of clients and load* |
| ***Collaborative & Sharing Capability*** | ***Federated Query:***  *We view the federated query as a data platform feature where data privacy is not of concern.*  *From a technical point of view, FLARE can easily support federated query, as FLARE is a federated compute platform: the query command can be issued from controller and distributed to each client executor and controller aggregate the result to user.*  *But we don’t support federated query. The key promise of Federated learning is to bring the training/evaluation/compute to data, but keep the data local.*  *A federated query is kind of breaking this promise unless there is sufficient security quarantee.*  *Adding the permission as to who can view the query results will not change the facts that the raw data is potentially leaked.*  *One exception for this could be Federated Query under Confidential Computing protected Clean room solution, in such conditions, the Clean room is TEE of confidential computing with access lockdown, user can not view the results. We will support this kind of use case.*  *On the other hand, we support federated statistics with additional privacy filters. This allows one to get a sense of the data without looking at the data.*  ***Collaborated Tools***  *Data sharing or collaborative workspace are also data platform features, not private privacy guarantees. This is the same as the above mentioned clean room solution.*  ***Permission management***  *Since we don’t view above FL platform features, we don’t have the permission management for above features.*  *But we do have extensive permission and security control in general as discussed above* |
| ***Cost and Sustainability*** | ***Cost-effective:*** *NVIDIA FLARE is free of charge*  ***Sustainability:*** *NVIDIA FLARE is maintained by the NVIDIA Federated learning team with contributions from the FL community. It powers many ISVs as well as many global service integrators. All major CSPs vendors has integration with NVFLARE* |
| ***Ethic*** | ***NA***  *FLARE doesn’t keep any private or public datasets.* |
| ***Innovation & Adaptability*** | ***Innovation:*** *FLARE is constantly innovating, taking the latest work from all industries and customer feedback. Here are a few latest developments*   - *Confidential Federated AI – end-to-end confidential federated learning, supporting AI workload for both in-cloud on-prem – cutting edge development leveraging confidential computing* - *unlimited LLM streaming – streaming any size LLM ( event it is bigger than GPU memory)* - *Secure Fed XGBoost with cuda acceleration* - *5-min to FL – easy FL Client API turns any DL code into FL client training code with a few lines of code changes.* - *CellNet – point-to-point (cell-to-cell) network that can connect any two logical or physical end-point. support client-server, peer-to-peer connections.*   ***Adaptability:***   - *Easy to adapt to any workflow with FLARE custom plugin component mechanism* - *One example to show-case such capability*   - *Fed RAG, – use NVFLARE as an multi-agent RAG framework for coordinated RAG retrieval* |

***References*** [***https://github.com/NVIDIA/NVFlare/tree/main/examples/advanced/federated-policies***](https://github.com/NVIDIA/NVFlare/tree/main/examples/advanced/federated-policies)

[***https://github.com/NVIDIA/NVFlare/tree/main/examples/advanced/custom_authentication***](https://github.com/NVIDIA/NVFlare/tree/main/examples/advanced/custom_authentication)

[***https://github.com/NVIDIA/NVFlare/tree/main/research/fed-ce***](https://github.com/NVIDIA/NVFlare/tree/main/research/fed-ce)

[***https://nvflare.readthedocs.io/en/main/real_world_fl/notes_on_large_models.html#notes-on-large-models***](https://nvflare.readthedocs.io/en/main/real_world_fl/notes_on_large_models.html#notes-on-large-models)

[***https://github.com/NVIDIA/NVFlare/tree/main/examples/advanced/federated-statistics***](https://github.com/NVIDIA/NVFlare/tree/main/examples/advanced/federated-statistics)

[***https://developer.download.nvidia.com/assets/Clara/flare/NVFLARE_DAY_2024_Part_10_Deloitte.mp4***](https://developer.download.nvidia.com/assets/Clara/flare/NVFLARE_DAY_2024_Part_10_Deloitte.mp4)

***Matrix table for the NVIDIA FLARE Common Challenges***

| ***Criteria*** | ***Details*** |
| --- | --- |
| ***Federated Query*** | ***NA***  *We provide federated statistics and hierarchical fed stats, which reports min/max/std/var/mean/histogram for numerical features* |
| ***Patient privacy and data protection*** | *NVFLARE offers strong data privacy protection via different techniques. DP, HE, Confidential computing. Plus a set of security measures, local policies* |
| ***organizational policies*** | *NVFALRE supports the local organization specific policies including authentication, authorization and other policies* |
| ***Data transformation*** | *NVFLARE will not have this challenge as NVFLARE is not trying to do the data transformation for the customers. If needed, NVFLARE will help coordinate the data transformation leveraging the site-specific favored tools* |
| ***Installation and Maintenance*** | *To set up the FL system, the challenges are mostly on business and legal site rather than the installation side, these involves process to establish consortium, model legal right, data contributions etc*  *FLARE offers both CLI way and Dashboard UI to distribute the setup software and also includes pre-flight check to make setup easier.*  *ISV vendors consistently update the new FLARE release to keep up the new features.* |
| ***Secure Deployment*** | *NVFLARE requires network communication, besides needed ports. FLARE can operate in an airgap env. Where all dependencies can be pre-installed.*  *NVFLARE can be easily installed ( one line CLI command) in both Azure and AWS* |
| ***Understanding User Query*** | *NA* |
| ***Informatics and User Experience*** | *The question is kind related to the data platform, query data but at the same time want to shield users from seeing critical data.*  *From a federated learning perspective, we don’t let users access the raw data, as any raw data retrieval can be information leak. The risk is dependent on use cases and industries. In healthcare, leak an CT image of anonymous user, may be not as serious as leaking an revenue number from financial institutions*  *What we provide is Federated Statistics. Even in such cases, we don’t report statistics if the sample size is too small ( users can define what small means). We don’t reveal real individual site’s max/min values. A series of privacy filters will be used to enforce such calculation* |
| ***Complexity & Adaptability*** | ***Complexity***  *NVFLARE is a modular design, the overall system is not complex. The complexity comes with the concept FLARE introduced, which is not familiar to the data scientist/researcher. As they have to get familiar with new terminologies and concepts. There was a learning curve prior to FLARE 2.4.0*  *This has started to change since FLARE 2.4.0, we introduced higher-level structures which convert FLModel, which literally contains weight, optimizer, metrics and metadata fields, all familiar to the data scientists. We then make the new Client API which can turn any ML/DL code to FL code with few lines of code changes, no need for new class or structure changes. For pytorch-lightning DL code, we just need 4 lines of code changes (including import)*  *FLARE 2.5.0 further improves this with a job API which allows writing python code end-to-end, client, server, configuration generation and simulator run with 15-30 lines of code.*  *We are continuing to improve the API to make all researchers to easily learn FALRE.*  [*https://nvidia.github.io/NVFlare/*](https://nvidia.github.io/NVFlare/)  ***Adaptability :***  *FLARE is federated compute at core, with a robust plugin system, Local and Federated Event messaging system, cell-to-cell communication network, therefore it is fairly easily adapted to any workload*  *Here are examples*   - *Use FLARE to perform Private Set Intersection (PSI) needed for vertical learning* - *Split learning* - *Peer-to-Peer Swarm learning* - *Federated ETL with feature engineering and pre-process* - *Federated RAG* - *Federated Learning with HPC, interact with 3rd party system with Client Agent*   *Here are few different type of applications*   - *Federated Scilkit-learn: Logistics regression, Random forest, XGBost* - *Kaplan-merer survival analysis* - *LLM tuning, p-tuning, SFT, PEFT (Lora)* - *Drug discovery: FLARE + BioNemo* - *FLARE + Keycloak to illustrate federated authentication*   [*https://github.com/NVIDIA/NVFlare/tree/main/examples*](https://github.com/NVIDIA/NVFlare/tree/main/examples)  [*https://github.com/NVIDIA/NVFlare/tree/main/research*](https://github.com/NVIDIA/NVFlare/tree/main/research) |
| ***Incremental update limitations*** | *Some customers with older version of NVFARE may have difficulty to upgrade due to the architecture changes in 2.2*  *But the majority of the customers are in the new versions.* |
| ***Standardized vocabularies and Flexibility*** | *Private FLARE namespace in the variables should avoid this problem* |

***Reference:***

*All points made above can be found in FLARE documentation, examples, and publications*

***Data Modalities Supported by NVFLARE***

*NVIDIA FLARE doesn’t support this:*

- *FLARE is not a data platform*
- *FLARE is not medical specific*

***Built-in Workflows and Analysis Tools***

***Workflow***

*The examples displayed in the survey show medical specific data pipeline workflows, which does not apply to FLARE. FLARE can certainly implement these workflows, but they are not core to FLARE.*

*Instead, I will add few common workflow patterns that are generic to all FL*

| ***Feature*** | ***Description*** |
| --- | --- |
| ***Scatter & Gather*** | *Similar to MPI scatter and gather* |
| ***Cyclic weight transfer*** | *round-robin* |
| ***Split learning*** | *Split the model layers training between server and client*  [*https://github.com/NVIDIA/NVFlare/tree/main/examples/advanced/vertical_federated_learning/cifar10-splitnn*](https://github.com/NVIDIA/NVFlare/tree/main/examples/advanced/vertical_federated_learning/cifar10-splitnn) |
| ***Swarm learning*** | *scatter and gather among all clients with random selected client as aggregator node for each round*  [*https://github.com/NVIDIA/NVFlare/tree/main/examples/advanced/swarm_learning*](https://github.com/NVIDIA/NVFlare/tree/main/examples/advanced/swarm_learning) |
|  |  |

***FLARE customers have built some of these workflow to their needs for example***

***Patient Cohort Discovery –*** [*https://www.oasysnow.com/about*](https://www.oasysnow.com/about)

***Data integration and Management*** *–* [*https://www.apheris.com/*](https://www.apheris.com/)

***Ontology management*** *– metadata management – No*

***Security & Access Management*** *– Yes NVFLARE has strong support for security and privacy as discussed above.*

*Role-based on PKI authentication, customizable, federated local-site specific authorization and authorization at job level, customizable site-specific local privacy policy, secure messaging, homomorphic encryption support, Differential difference support. Unique end-to-end confidential Federated AI features (coming soon)*

***Data Extraction and Transformation –*** *FLARE is capable of performing this task either by instructing data ETL (extraction transformation and load) at client side directly, or integrating with other ETL tools such as Spark or Ray. Here is an example of FLARE performing federated feature enrichment and pre-process before training for Credit card fraud detection example.* [*https://github.com/NVIDIA/NVFlare/tree/main/examples/advanced/finance-end-to-end*](https://github.com/NVIDIA/NVFlare/tree/main/examples/advanced/finance-end-to-end)

***Analysis Tools***

*Most analytics tools are not applicable for the FL framework. But I will just list few might be relavant*

- *plugable – every layer is plugable*
- [*Statistics & Analysis*](https://github.com/NVIDIA/NVFlare/tree/main/examples/advanced/federated-statistics)
  - *Federated Statistics, with notebook visualization*
  - *Hierarchical federated statistics, notebook visualization*
    - *web UI visualization in development*
- *NLP – not sure what you mean by NLP service, NLP training yes, see example in repo. RAG application – yes, see Deloitte’s talk ( code is not open source yet)*

[*https://github.com/NVIDIA/NVFlare/tree/main/examples/advanced/nlp-ner*](https://github.com/NVIDIA/NVFlare/tree/main/examples/advanced/nlp-ner)

[*https://github.com/NVIDIA/NVFlare/tree/main/examples/advanced/llm_hf*](https://github.com/NVIDIA/NVFlare/tree/main/examples/advanced/llm_hf)

[*https://github.com/NVIDIA/NVFlare/tree/main/integration/nemo/examples*](https://github.com/NVIDIA/NVFlare/tree/main/integration/nemo/examples)

- *Genomic Data Analysis – no, Genomic Model training – yes. See* [*example*](https://github.com/NVIDIA/NVFlare/tree/main/examples/advanced/bionemo)
- *Integration with tools – Python yes, others – No.*

***Support for Semantic Integration***

*No, these are domain specific.*

*We couldn’t mind work with some organization(s) to leverage FLARE and added domain specific workflows and specific functions needed by medical community*
